# New quinoline and isatin derivatives as apoptotic VEGFR-2 inhibitors: design, synthesis, anti-proliferative activity, docking, ADMET, toxicity, and MD simulation studies

**DOI:** 10.1080/14756366.2022.2110869

**Published:** 2022-08-16

**Authors:** Eslam B. Elkaeed, Mohammed S. Taghour, Hazem A. Mahdy, Wagdy M. Eldehna, Nehal M. El-Deeb, Ahmed M. Kenawy, Bshra A. Alsfouk, Mohammed A. Dahab, Ahmed M. Metwaly, Ibrahim H. Eissa, Mohamed A. El-Zahabi

**Affiliations:** a Department of Pharmaceutical Sciences, College of Pharmacy, AlMaarefa University, Riyadh, 13713, Saudi Arabia; b Pharmaceutical Medicinal Chemistry & Drug Design Department, Faculty of Pharmacy (Boys), Al-Azhar University, Cairo, Egypt; c Department of Pharmaceutical Chemistry, Faculty of Pharmacy, Kafrelsheikh University, Kafrelsheikh, Egypt; d Biopharmaceutical Products Research Department, Genetic Engineering and Biotechnology Research Institute, City of Scientific Research and Technological Applications (SRTA-City), Alexandria, Egypt; e Pharmaceutical and Fermentation Industries Development Center, City of Scientific Research and Technological Applications (SRTA city), Alexandria, Egypt; f Nucleic Acids Research Department, Genetic Engineering and Biotechnology Research Institute. City of Scientific Research and Technological Applications (SRTA-City), Alexandria, Egypt; g Department of Pharmaceutical Sciences, College of Pharmacy, Princess Nourah bint Abdulrahman University, Riyadh, Saudi Arabia; h Pharmacognosy and Medicinal Plants Department, Faculty of Pharmacy (Boys), Al-Azhar University, Cairo, Egypt

**Keywords:** VEGFR-2, anticancer, isatin derivatives, quinoline derivatives, apoptosis, gene expression, cell cycle analysis, docking, MD simulations

## Abstract

New quinoline and isatin derivatives having the main characteristics of VEGFR-2 inhibitors was synthesised. The antiproliferative effects of these compounds were estimated against A549, Caco-2, HepG2, and MDA-MB-231. Compounds **13** and **14** showed comparable activities with doxorubicin against the Caco-2 cells. These compounds strongly inhibited VEGFR-2 kinase activity. The cytotoxic activities were evaluated against Vero cells. Compound **7** showed the highest value of safety and selectivity. Cell migration assay displayed the ability of compound **7** to prevent healing and migration abilities in the cancer cells. Furthermore, compound **7** induced apoptosis in Caco-2 through the expressive down-regulation of the apoptotic genes, Bcl2, Bcl-xl, and Survivin, and the upregulation of the TGF gene. Molecular docking against VEGFR-2 emerged the interactions of the synthesised compounds in a similar way to sorafenib. Additionally, seven molecular dynamics simulations studies were applied and confirmed the stability of compound **13** in the active pocket of VEGFR-2 over 100 ns.

## 1. Introduction

The WHO estimated the number of global deaths because of cancer to be more than ten million humans in 2020. Among them, 935,000 people died because of colon and rectum cancer^1^. Colon cancer was described by the NHS as one of the four most common cancer types^2^. It was estimated that from 2007 to 2016 both incidence and mortality of colorectal cancer increased in countries that have medium and high Human Development Index as well as in the younger people^3^. The global number of new cases diagnosed with colorectal cancer was 1,096,601 in 2018^4^.

Apoptosis originated from a Latin word that means “to fall off” and scientifically can be defined as programmed cell death. In the early stages of growth, apoptosis is the mechanism that the body uses to get rid of unwanted cells such as the soft tissues between the fingers of the growing hand^5^. Apoptosis is the main mechanism utilised by the human body to eliminate damaged cells. Apoptosis plays a crucial role in the process of cancer prevention and treatment. The blockage of apoptosis in a cell resulted in its uncontrolled division and subsequently its development to be malignant^6^. In order to survive and expand, malignant cells utilise various strategies to modulate the apoptotic signals inhibiting apoptosis at both protein and genetic levels^7^.

Vascular Endothelial Growth Factor (VEGF) family exhibited strong antiapoptotic activities in addition to its effect as angiogenesis promoters^8–10^. VEGF is described as the strongest pro-angiogenic protein. VEGF potentiates the proliferation as well as the tube formation of endothelial cells^11^. Also, VEGF induces endothelial nitric oxide synthase causing vasodilatation^12^. VEGF exhibits its effect via binding with certain receptors on the cell surface. These receptors are the tyrosine kinase receptors including VEGF receptor-1 (VEGFR-1) besides VEGFR-2^13^. The interaction of VEGF to the receptor’s extracellular domain results in the activation of a cascade of downstream enzymes. VEGFR-2 was identified as the major key receptor that mediates the pro-angiogenic activities of VEGF^14^.

The utilisation of computers (*in silico*) in the fields of drug design and discovery appeared as a relevant approach that can be employed in the discovery of active and safe candidates. Computational chemistry has the privilege of limiting time, efforts, and costs in addition to saving animal lives^15–17^. Various *in silico* methods were employed successfully in drug design, discovery, DFT, ADMET, and toxicity of new drugs^18^.

Our teamwork employed the *in silico* drug design approach to discover various novel VEGFR-2 inhibitors. The designed candidates were synthesised and examined against the VEGFR-2 enzyme. These candidates were belong to various chemical classes such as quinazoline^19^, quinoxaline-2 (1*H*)-one^20^, and thieno[2,3-*d*]pyrimidine^21^.

Based on our attempts to develop potent anti-VEGFR-2 inhibitors, two novel sets of quinoline-thiazolidine-2,4-dione and isatin-thiazolidine-2,4-dione hybrids were produced through the modification of some reported inhibitors of VEGFR-2. The targeted candidates were designed to maintain the key pharmacophoric characteristics of inhibitors of VEGFR-2, and they were tested to demonstrate their cytotoxic activities against human malignant cell lines as well as their inhibitory activities against the VEGFR-2 protein.

### 1.1. Rationale

VEGFR-2 inhibitors have four key pharmacophoric features, according to prior publications. (i) A hetero aromatic ring structure capable of engaging Cys917 at the hinge region^22^. (ii) A spacer moiety capable to be directed in the spacer region of the active site^23^. (iii) A pharmacophore moiety (e.g. amide or urea) that can bind to Glu883 and Asp1044 at the DFG motif region. (iv) A hydrophobic group resides in the allosteric pocket of the VEGFR-2 binding site^24^.

**Figure 1.**
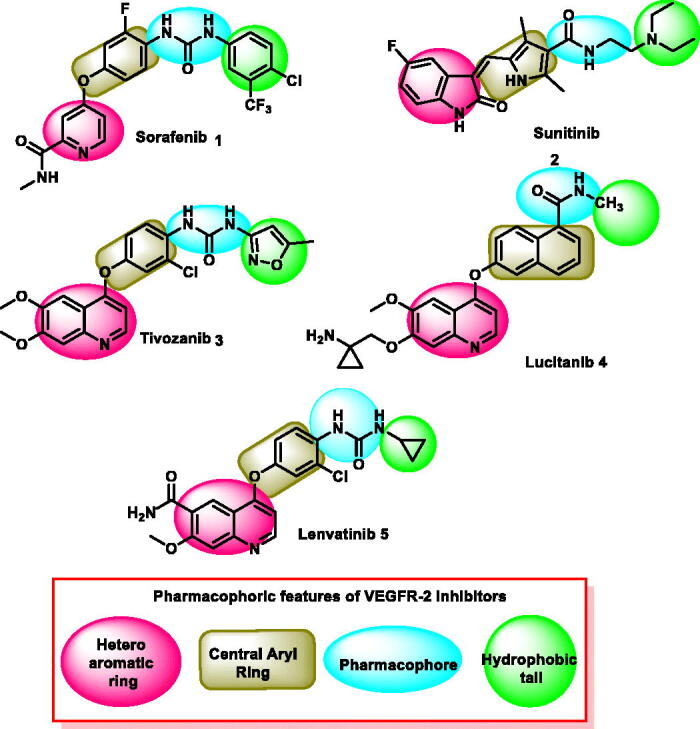
Reported VEGFR-2 inhibitors and their essential inhibitory charachterstics.

Quinoline, isatin, and thiazolidine-2,4-dione are three scaffolds that have great interest in the field of drug synthesis and discovery. These scaffolds were observed in many reported anticancer agents, especially VEGFR-2 inhibitors. Three FDA VEGFR-2 inhibitors (lenvatinib, **2**, tivozanib, **3**, and lucitanib, **4**) comprise the quinoline moiety as a hetero aromatic system. Another FDA VEGFR-2 (sunitinib, **5**) comprises the isatin moiety. In addition, sunitinib, **5**, comprises the 2,4-dimethyl-1*H*-pyrrole moiety as a linker (Figure 1).

**Figure 2.**
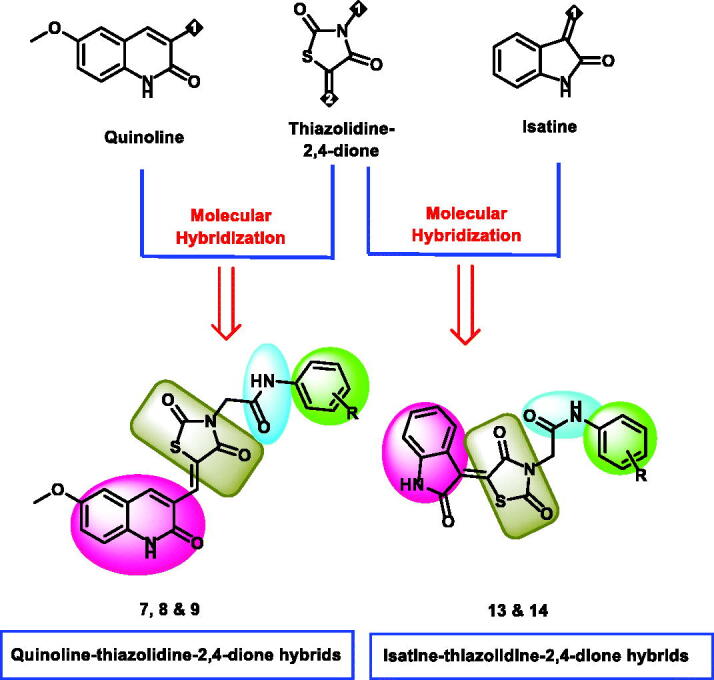
The strategy of molecular design.

Utilising ligand-based drug design, especially the molecular hybridisation strategy that entails the connection of two or more groups with significant biological capabilities^25^, Two series of VEGFR-2 were design new hybrids of quinoline-thiazolidine-2,4-dione (compounds **7**, **8**, and **9**) and isatin-thiazolidine-2,4-dione(compounds **13** and **14**). As shown in Figure 2, the heteroaromatic system was designed to be quinoline or isatin moieties. The liker group was the thiazolidine-2,4-dione moiety as a ring equivalent for 2,4-dimethyl-1*H*-pyrrole of sunitinib with increased the advantage of being a good centre for hydrogen bonding interactions and enhancement of water solubility of the synthesised compounds. The pharmacophore moiety was kept to be an amide group in all the designed compounds. The terminal hydrophobic moiety was kept to be different substituted aromatic structures.

## 2. Results and discussion

### 2.1. Chemistry

**Scheme 1.**
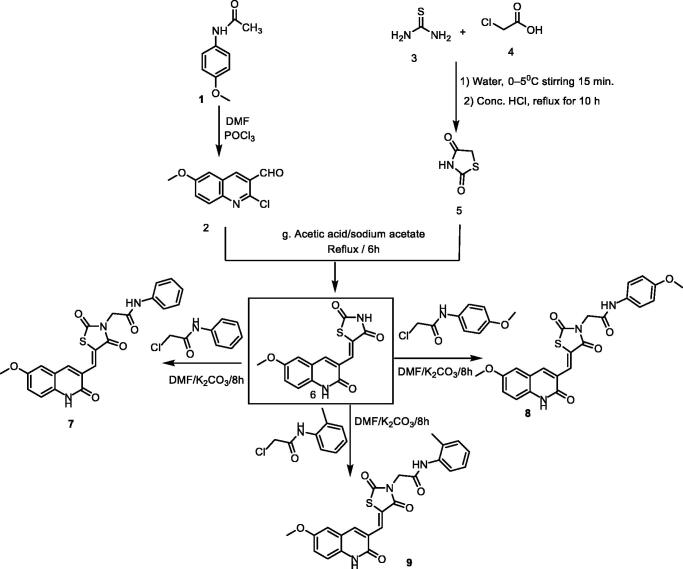
Synthetic pathways of compounds **7**, **8**, and **9**.

**Scheme 2.**
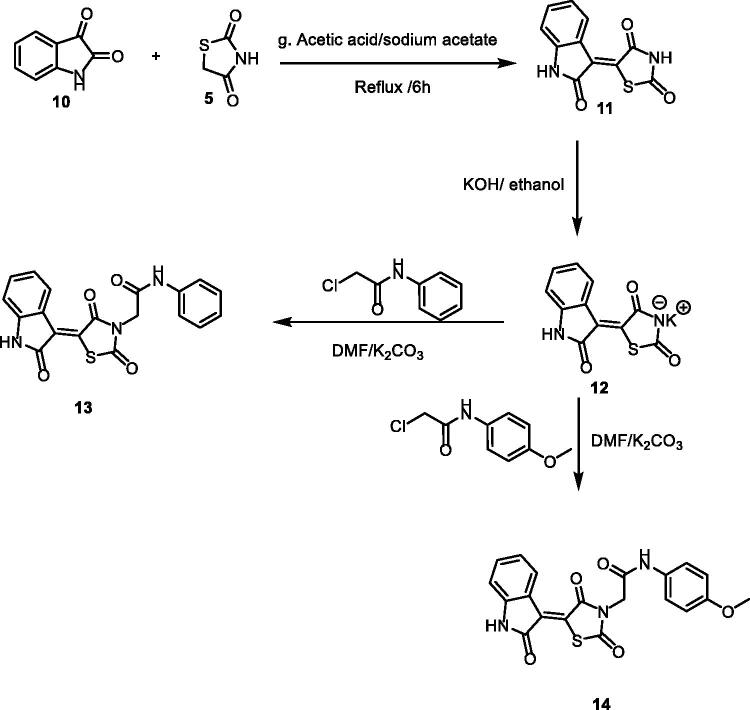
Synthetic pathways of compounds **13** and **14**.

The synthetic pathways adopted to obtain the target compounds are presented in Schemes 1 and 2. Firstly, the synthesis of the key starting compound **2** (2-chloro-6-methoxyquinoline-3-carbaldehyde) (Scheme 1) was achieved through chlorination, formylation, and cyclisation of *N-*(4-methoxyphenyl)acetamide **1** using DMF/POCl_3_ to give 2-chloro-6-methoxyquinoline-3-carbaldehyde **2**, according to the reported procedure^26^. On the other hand, refluxing the thiourea **3** with 2-chloroacetic acid **4** in water contains 4 N HCl, afforded thiazolidine-2, 4-dione **5**^27^. The condensation of compound **5** with 2-chloro-6-methoxyquinoline-3-carbaldehyde **2** in glacial acetic acid/sodium acetate mixture in accordance with the Knoevenagel condensation^28^, furnished the final benzylidine product **6**. Treatment of compound **6** with 2-chloroacetamide derivatives in refluxing DMF using anhydrous K_2_CO_3_ as base and KI as a nucleophilic catalyst to afford the target derivatives **7**, **8**, and **9**.

^1^H NMR spectra **7**, **8**, and **9** showed the appearance of aliphatic protons of the methylenes as shielded singlet signals at 4.49 − 4.55 ppm, and singlet signals around *δ* 3.50 ppm of the methoxy group. In addition, the benzylidene methine protons exhibited singlet signals in the range of *δ* 7.98 − 7.99 ppm. This methine was also detected in the ^13^C NMR spectra at *δ* of 142.0 ppm. Moreover, their ^1^H NMR spectra revealed the presence of two NH protons at *δ* ranges of 10.24 − 10.43 ppm and 12.15 − 12.16 ppm. In addition, ^13^C NMR showed the presence of a methylene carbon in the *δ* range of 46.73–56.03 ppm. Two amide carbonyls were displayed in the ^13^C NMR spectrum at the *δ* range of 166.1–160.5 ppm.

Synthesis of compound **11** (Scheme 2) was achieved via refluxing of thiazolidine-2,4-dione **4** with isatin **10** in glacial acetic acid and anhydrous sodium acetate. Consequent treatment of **11** with alcoholic potassium hydroxide provided the corresponding salt **12**. Heating of **12** with 2-chloroacetamide derivatives in dry DMF afforded the target compounds **13** and **14**. ^1^H NMR spectra data showed shielded singlet signals of the methylene protons (aliphatic) at the *δ* range of 4.55 − 4.59 ppm. In addition to2NH protons at the *δ* ranges of 10.38 − 10.49 ppm and 11.31 − 11.34 ppm.

### 2.2. Biological evaluation

#### 2.2.1. *In-vitro* anticancer effects

To assess the antiproliferative effects of the targeted candidates, an MTT assay^29–31^ was performed against four cancer cell lines: lung carcinoma epithelial (A549), colon cancer (Caco-2), hepatocellular cancer (HepG2), and breast cancer (MDA-MB-231). The results were listed in Table 1 as IC_50_ values.

**Table 1.** *In vitro* anti-proliferative activities.

Compounds	Anti-proliferative activity (IC_50_ µM)^a^			
	A549	Caco-2	HepG-2	MDA-MB231
**7**	159 ± 14	93.5 ± 0.71	150 ± 7.07	122.5 ± 7.01
**8**	196 ± 70	189.5 ± 9.11	134 ± 1.41	130 ± 5.60
**9**	51 ± 4.20	167 ± 4.20	145 ± 3.50	188 ± 7.01
**13**	49.5 ± 0.70	9.3 ± 0.421	149 ± 9.80	28 ± 0.50
**14**	54 ± 1.40	5.7 ± 0.07	149 ± 7.01	9 ± 0.51
**Doxorubicin**	7 ± 0.22	8.2 ± 0.21	2.8 ± 0.07	9 ± 0.77

^a^The results were the mean of three replicates.

The results revealed that Caco-2 cells are the most sensitive cell line against the targeted candidates. In descending pattern, compounds **14**, **13**, and **7** are the most active candidates against Caco-2 cells with IC_50_ values of 5.7, 9.3, and 93.5 µM, respectively. Interestingly, compounds **13** and **14** showed comparable activity with that of doxorubicin against Caco-2 cells (IC_50_ = 8.2 µM). Compounds **13** and **14** are 0.88 and 1.44 times as active as doxorubicin. In addition, compound **14** was the most active member against MDA-MB231 cells showing an equal IC_50_ value (9 µM) to that of doxorubicin.

From the results of cytotoxicity against the four cell lines, it can be deduced that isatin derivatives (**13** and **14**) are more cytotoxic than quinoline derivatives (**7**, **8**, and **9**) against three cell lines (A549, Caco-2, and MDA-MB-231). Furthermore, by comparing the cytotoxicity of the tested compounds against the Caco-2 cell line, we can reach available structure-activity relationships regarding the hydrophobic tail. It was found that the phenyl ring is more advantageous as a hydrophobic tail than *p*-methoxyphenyl moiety, and the latter is more beneficial for activity than *o*-tolyl moiety.

#### 2.2.2. VEGFR-2 inhibition

As the main target in this work is the design and synthesis of promising VEGFR-2 inhibitors, we subjected the synthesised compounds to *in vitro* VEGFR-2 inhibitory assay to assess the ability of these compounds to obstacle the kinase activity of VEGFR-2. The results were summarised in Table 2 as IC_50_ values in a nanomolar unit.

**Table 2.** VEGFR-2 inhibitory assay for the targeted candidates and sorafenib.

Compounds	VEGFR-2 inhibitory activity IC_50_ (nM)^a^
**7**	137.40
**8**	187.00
**9**	98.53
**13**	69.11
**14**	85.89
**Sorafenib**	53.65

^a^The results were the mean of three replicates.

The results revealed that the isatin derivatives (compounds **13** and **14**) are the most active members exhibiting strong IC_50_ values of 69.11 and 85.89 nM, respectively. Compounds **13** and **14** were 0.78 and 0.70 times as active as sorafenib (IC_50_ = 53.65 nM). Additionally, compound **9** showed moderate VEGFR-2 inhibitory activity with an IC_50_ value of 98.53 nM (0.54 times of sorafenib). On the other hand, compounds **7** and **8** showed weak activities with IC_50_ values of 137.40 and 187.00 nM, respectively.

#### 2.2.3. Cytotoxicity against normal cell lines

The cytotoxic activities of the synthesised against normal cells were evaluated against the Vero cell line utilising an MTT assay. The results were summarised in Table 3.

**Table 3.** Cytotoxicity of the targeted candidates against the Vero cell line

Compounds	Cytotoxicity (IC_50_ µM)
**7**	440 ± 14.10
**8**	150 ± 14.10
**9**	196 ± 2.80
**13**	26.5 ± 1.71
**14**	30 ± 1.35
**Doxorubicin**	25 ± 1.41

The results disclosed that the quinoline derivatives (compounds **7, 8,** and **9**) have very low cytotoxicity against Vero cells with IC_50_ values of 440, 150, and 196 µM, respectively. Although the isatin derivatives (compounds **13** and **14**) expressed higher cytotoxicity against the normal cells with IC_50_ values of 26.5 and 30 µM, respectively, the obtained results were safer than doxorubicin which showed an IC_50_ value of 25 µM. These results indicated the higher safety of quinoline derivatives over the isatin.

#### 2.2.4. Selectivity index (SI)

For further evaluation of the toxicity of the synthesised compounds, the selectivity index (SI) of these compounds was calculated. SI is the ratio of the IC_50_ value on normal cells to the IC_50_ value on cancer cells^32^. A compound with SI lower than 1 is considered to be toxic^33^^,^^34^.

**Figure 3.**
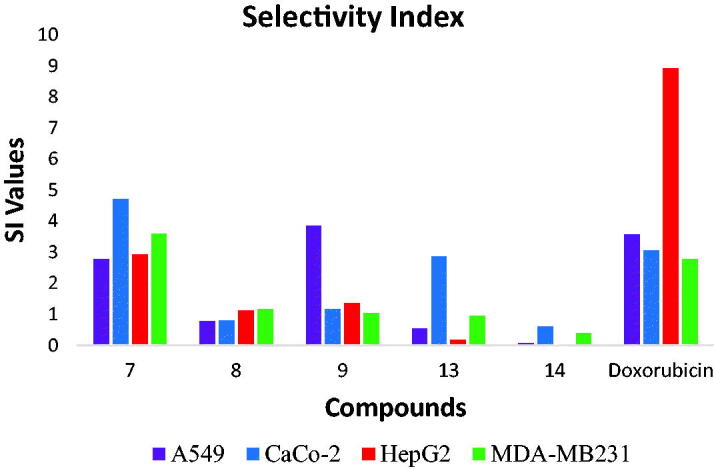
Selectivity indices of the synthesised compounds.

From the results of SI presented in Table 4, it can be observed that the SI of quinoline derivatives (**7** and **9**) are higher than 1 in the examined cell lines. Also, compound **8** revealed safe results against HepG2 and MDA-MB231 cell lines. On the other hand, the isatin derivatives showed SI values lower than 1, indicating their lower selectivity against normal cells (Figure 3). Accordingly, compound **7** of the highest selectivity index was selected for further biological analysis.

**Table 4.** Selectivity index of the synthesised compounds.

Compounds	A549	Caco-2	HepG2	MDA-MB231
**7**	2.77	4.71	2.93	3.59
**8**	0.77	0.79	1.12	1.15
**9**	3.84	1.17	1.35	1.04
**13**	0.54	2.85	0.18	0.95
**14**	0.06	0.61	0.02	0.39
**Doxorubicin**	3.57	3.05	8.93	2.78

#### 2.2.5. Wound healing assay (migration assay)

**Figure 4.**
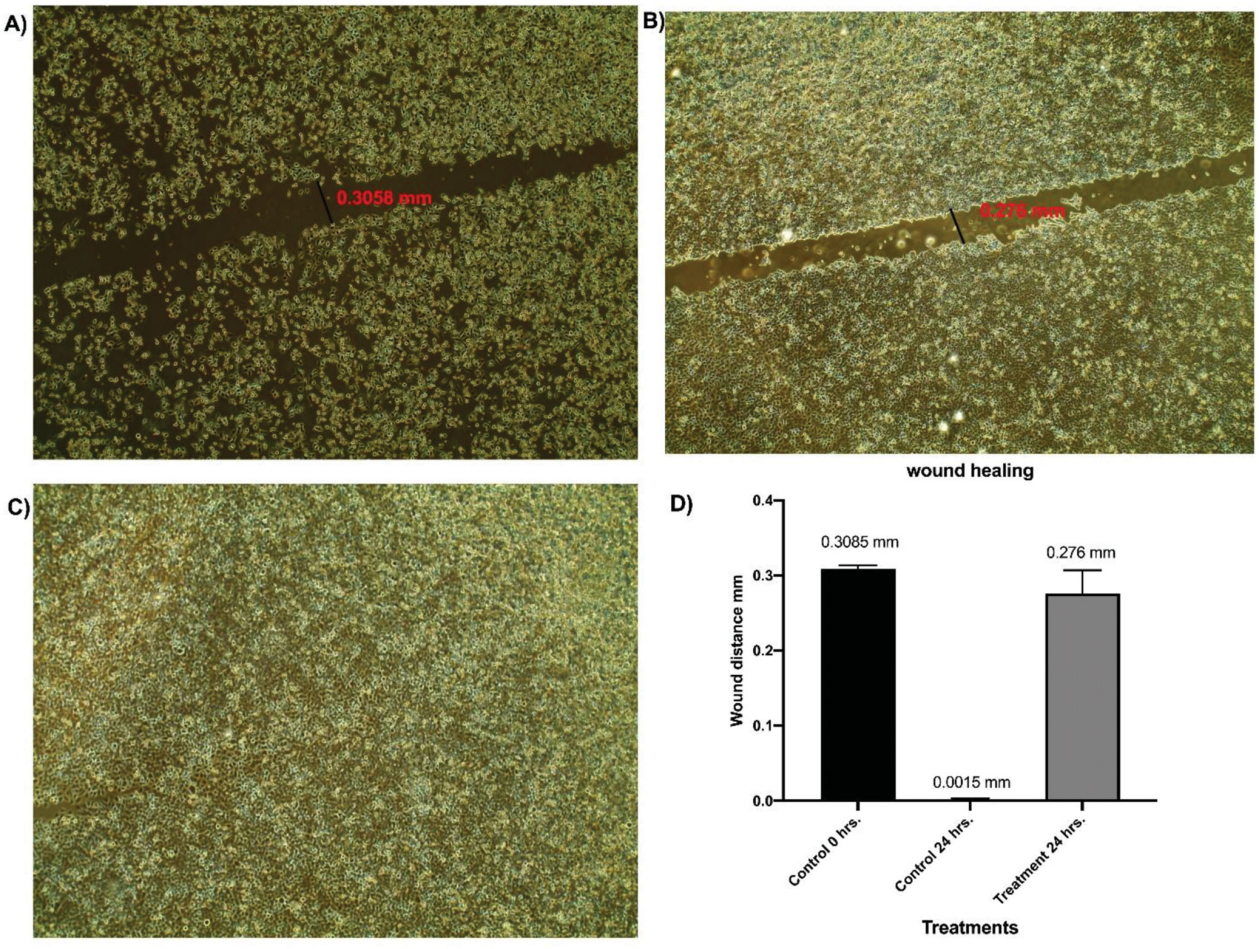
Effect of compound **7** on wound healing of Caco-2 cells at a concentration of 90 µM. (A) The treated cells with a diameter of 0.3058 mm. (B) the control cells with a diameter of 0.276 mm. (C) The treated cells after 24 h showing complete closure of wound. (D) Diagram of the wound healing test. Determination of apoptotic and anti-apoptotic gene expression.

*In-vitro* scratch assay^35^ was performed for compound **7** as it was the safest compound exhibiting the highest selectivity index. In this test, Caco-2 cells were allowed to grow then, a wound was formed on the cell layer. Next, the cells were incubated with the sub IC_50_ dose of compound **7**. The results of wound healing were compared to the untreated cell line. Figure 4 illustrates the degree of wound healing caused by compound **7** compared to the control cells.

From Figure 4(A) (the treated cells), it can be noticed that the diameter of the wound is equal to 0.3058 mm. on the other hand, Figure 4(B) (the control cells) showed a diameter of 0.276 mm. The wound was completely closed within 24 h as appeared in Figure 4(C). Such findings indicate the ability of compound 7 to prevent wound healing in the cancer population at a low concentration.

Apoptosis is an important mechanism for fighting the tumour. The apoptosis process comprises many gene families such as p53, caspases, and Bcl-2. The apoptosis mechanism is controlled by the balance between the pro-apoptotic and anti-apoptotic mediators. The Bcl-2 family (Bcl2 and Bcl-xl) is a well-known example of anti-apoptotic mediators^36^. Moreover, Survivin is an example of the overexpressed pro-survival protein in various cancer cells. Furthermore, the transforming growth factor (TGF) is an example of a pro-apoptotic mediator that suppresses and controls proliferation of malignant cells in its early stages^37^.

**Figure 5.**
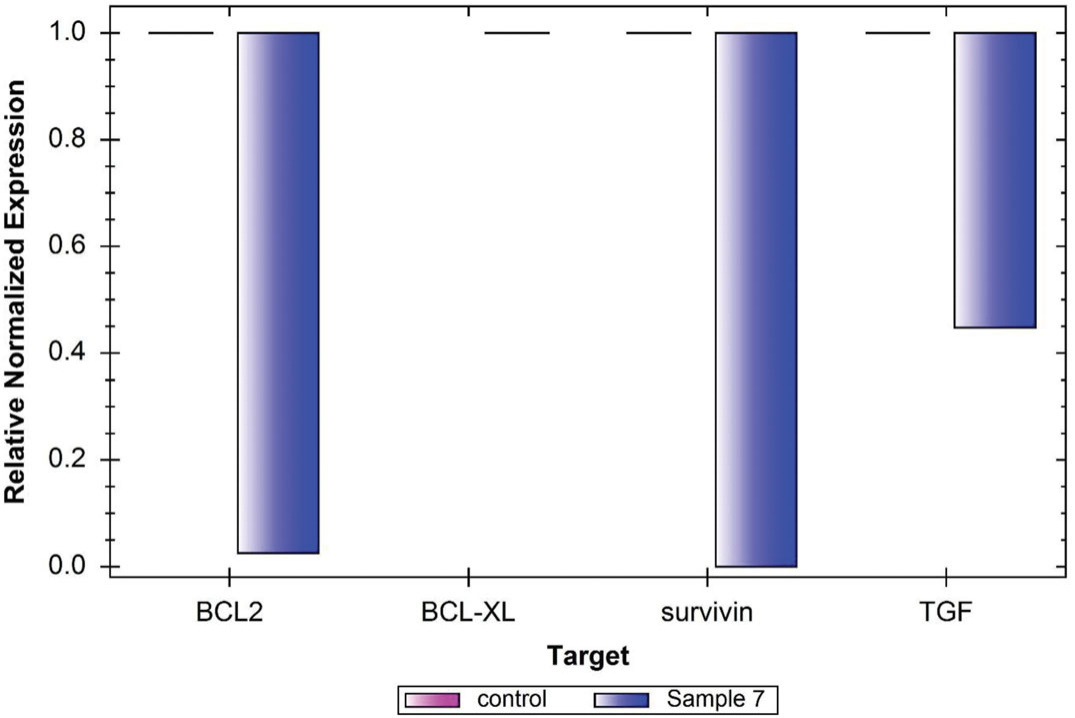
Relative expression of BCL2, BCLXL, Survivin, and TGF levels in Caco-2 cell line after treatment with 90 µM of compound **7** for 24 h showing an expressive down-regulation potential on the Bcl2, Bcl-xl, and Survivin apoptic genes as well as an upregulation potential on the TGF gene.

RT-qPCR technique was applied to assess the expression levels of Bcl2, Bcl-xl, Survivin, and TGF in Caco-2 cells after treatment with compound **7** for 24 h. As shown in Figure 5, compound **7** exhibited an expressive down-regulating potentialities against of Bcl2, Bcl-xl, and Survivin genes. On the other hand, such a compound produced an upregulation effect of the TGF gene. Taking these results into consideration, it can be concluded that compound **7** can induce apoptosis in Caco-2.

#### 2.2.7. Cell cycle analysis

**Figure 6.**
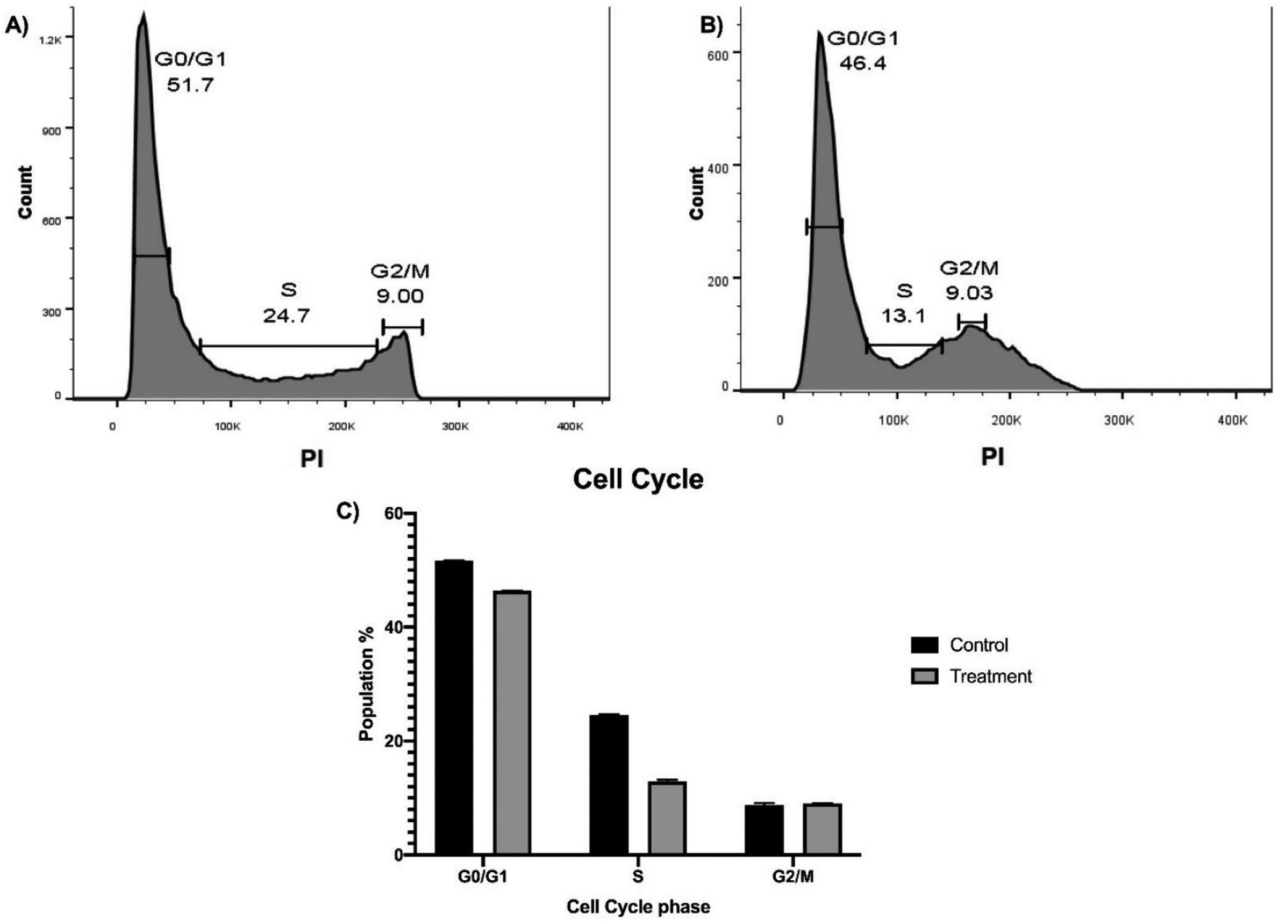
Flow cytometric cell cycle analysis of Caco-2 before (A) and after (B) a 24 h treatment with a 90 µM compound **7**. (A) the cell cycle of the untreated cells showed 51.7 and 24.7% for G0/G1 and S phases, respectively. (B) the cell cycle of the treated cells showed 46.4 and 13.1 for G0/G1 and S phases, respectively.

Employing the flowcytometry technique, the cell cycle pattern of the untrated Caco-2 cancer cells (Figure 6(A)) was compared with that of the treated cells with compound 7. The cell cycle pattern of Caco-2 cell line after treatment (Figure 6(B)) showed a decrease in the cell population in G0/G1 and S phases (46.4 and 13.1%, respectively) compared with the untreated cells (51.7 and 24.7%, respectively) which means the considered compound caused a cellular arrest in sub G0 (Apoptotic phase).

### 2.3. *In silico* (computational) studies

#### 2.3.1. Molecular docking

Molecular docking experiments were applied for the considered compounds to clarify their proposed binding modes against VEGFR-2 (PDB ID: 2OH4) using sorafenib as a reference. Table 5 summarises the calculated binding energies (ΔG) of the tested compounds and sorafenib.

**Table 5.** The computed ΔG values of the considered compounds and sorafenib against VEGFR-2.

Comp.	ΔG [kcal. mol^−1^]
**7**	−21.94
**8**	−21.84
**9**	−21.53
**13**	−17.44
**14**	−19.34
**Sorafenib**	−21.11

**Figure 7.**
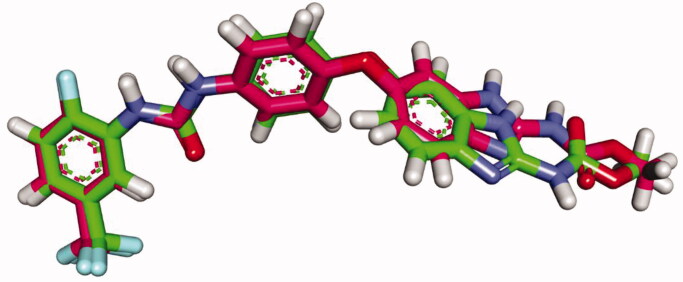
Superimposition of sorafenib (green) and the redocked one (pink) in the VEGFR-2 active site (RMSD = 0.98 Å).

To verify the docking procedure, sorafenib was docked alone against the active site. As shown in Figure 7, the re-docked pose showed a high degree of superimposition on the original ligand with an RMSD value of 0.98 A indicating the docking process validity.

**Figure 8.**
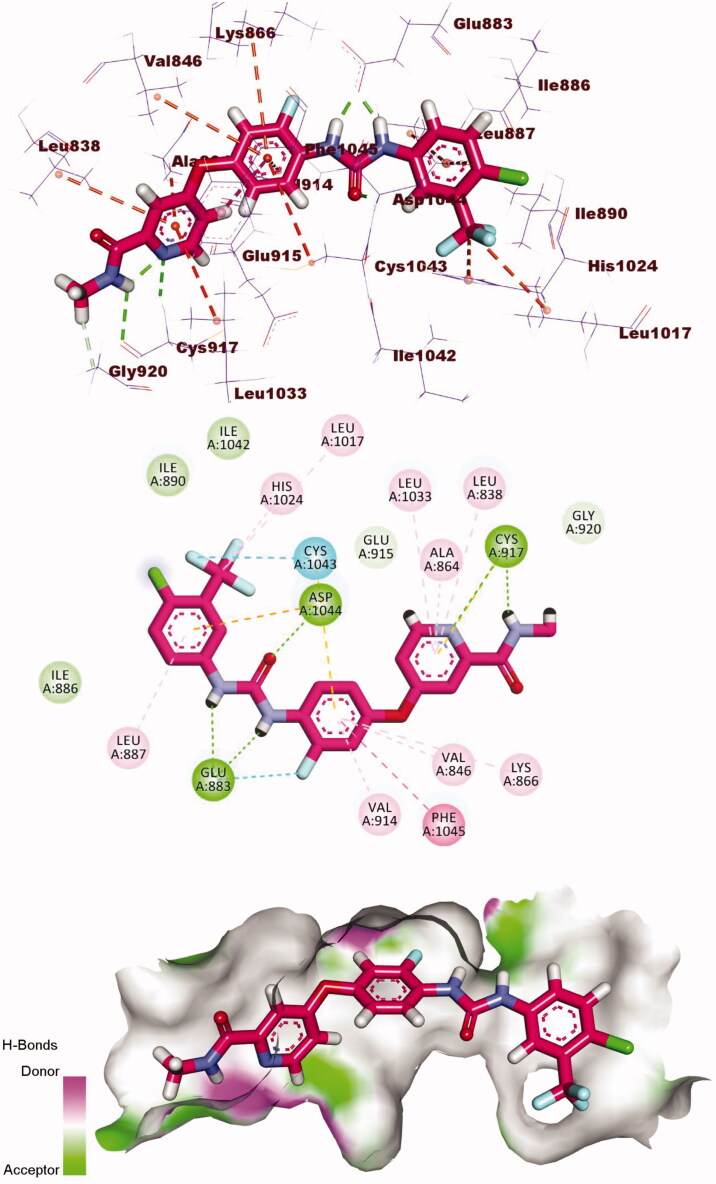
3D, 2D, and surface mapping of the binding mode of sorafenib into VEGFR-2. The hydrogen bonds were presented in green colour with Cys917, Glu883, and Asp1044. The hydrophobic bonds were presented in orange colour with Leu1033, Leu838, Ala864, Val846, Val914, Phe1045, Cys1043, Leu1017, His1024, and Leu887.

Sorafenib exhibited a binding energy of −21.11 kcal/mol. Sorafenib occupied the four essential regions on the active site forming two hydrogen bonds (H.Bs) with Cys917 and three hydrophobic interactions (H.Is) with Leu1033, Leu838, and Ala864 at the hinge region. The central phenyl ring formed six H.Is with Val846, Val914, Phe1045, and Cys1043. The urea group formed three H.Bs with Glu883 and Asp1044. The 1-chloro-2-(trifluoromethyl)benzene moiety formed five H.Is with Leu1017, His1024, cys1043, and Leu887. In addition, The 1-chloro-2-(trifluoromethyl)benzene moiety formed an electrostatic interaction (E.I) with Asp1044^24^^,^^38^ (Figure 8).

**Figure 9.**
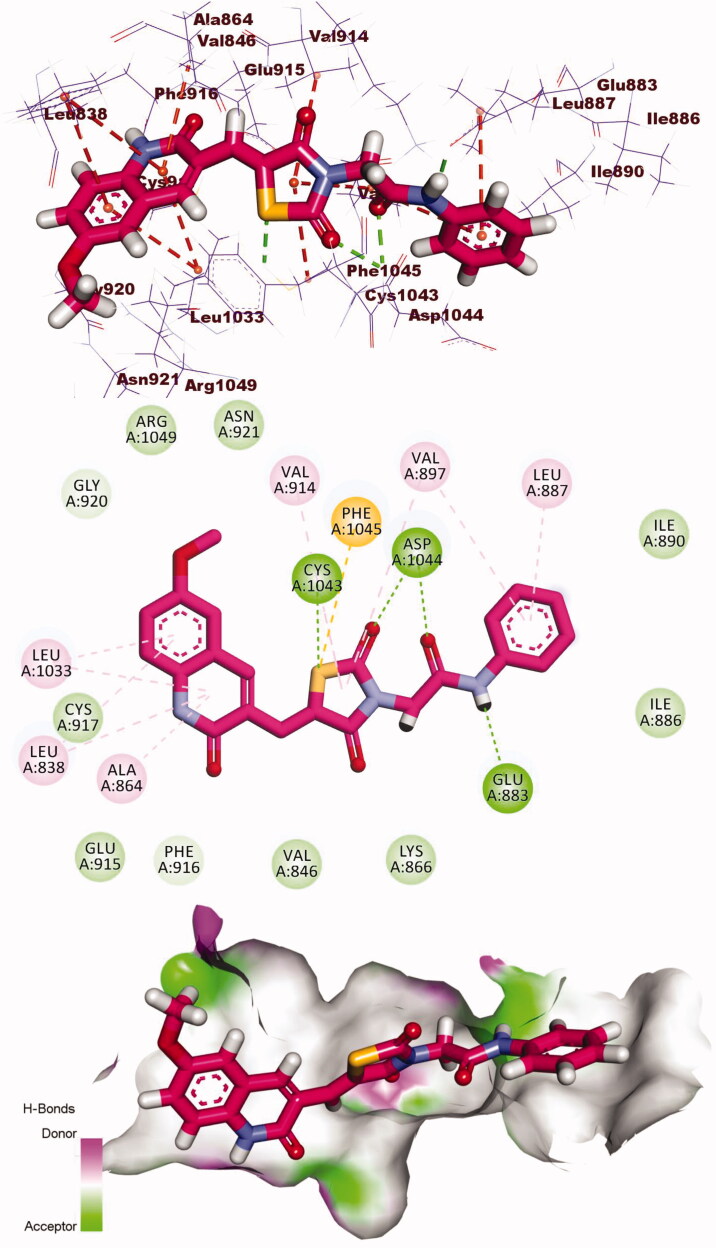
3D, 2D, and surface mapping of compound **7** into VEGFR-2. The hydrogen bonds were presented in green colour with Cys1043, Asp1044, and Glu883. The hydrophobic bonds were presented in orange colour with Leu838, Leu1033, Ala864, Cys917, Val914, Phe1045, Leu887 and Val897.

Compound **7** showed a binding mode like the reference molecule with a docking energy of −21.94 kcal/mol. The quinolin-2(1*H*)-one moiety formed five H.Is in the hinge region with Leu838, Leu1033, Ala864, and Cys917. The thiazolidine-2,4-dione (linker) moiety formed two H.B with Cys1043 and Asp1044. Also, it formed three hydrophobic bonds with Val914, Phe1045, and Val897. The pharmacophore (amide) moiety occupied the DFG region forming two H.Bs with Glu883 and Asp1044. The terminal phenyl ring occupied the allosteric pocket forming two H.I with Leu887 and Val897 (Figure 9).

**Figure 10.**
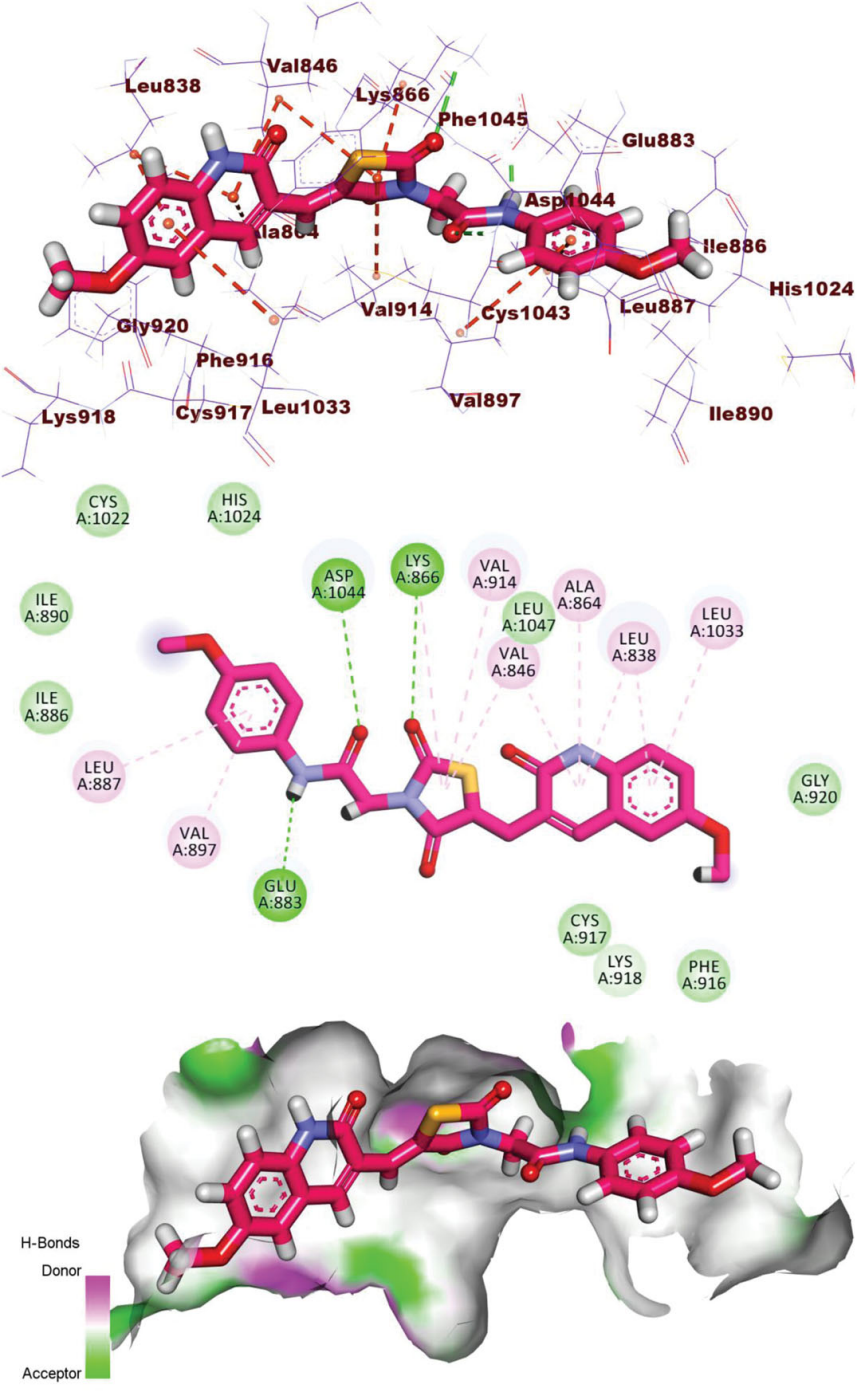
3D, 2D, and surface mapping of compound **8** into VEGFR-2. The hydrogen bonds were presented in green colour with Lys866, Asp1044, and Glu883. The hydrophobic bonds were presented in orange colour with Ala864, Leu838, Leu1033, Val846, Val914, Lys866, Leu887, and Val897.

Compound **8** showed docking energy of −21.84 kcal/mol. The quinolin-2(1*H*)-one moiety formed five H.Is in the hinge region with Ala864, Leu838, Leu1033, and Val846. The thiazolidine-2,4-dione (linker) moiety formed an extra H.B with Lys866 in addition to three hydrophobic bonds with Val846, Val914, and Lys866. The pharmacophore (amide) moiety occupied the DFG region forming two H.Bs with Glu883 Asp1044. The terminal phenyl ring occupied the allosteric pocket forming two H.I with Leu887 and Val897 (Figure 10).

**Figure 11.**
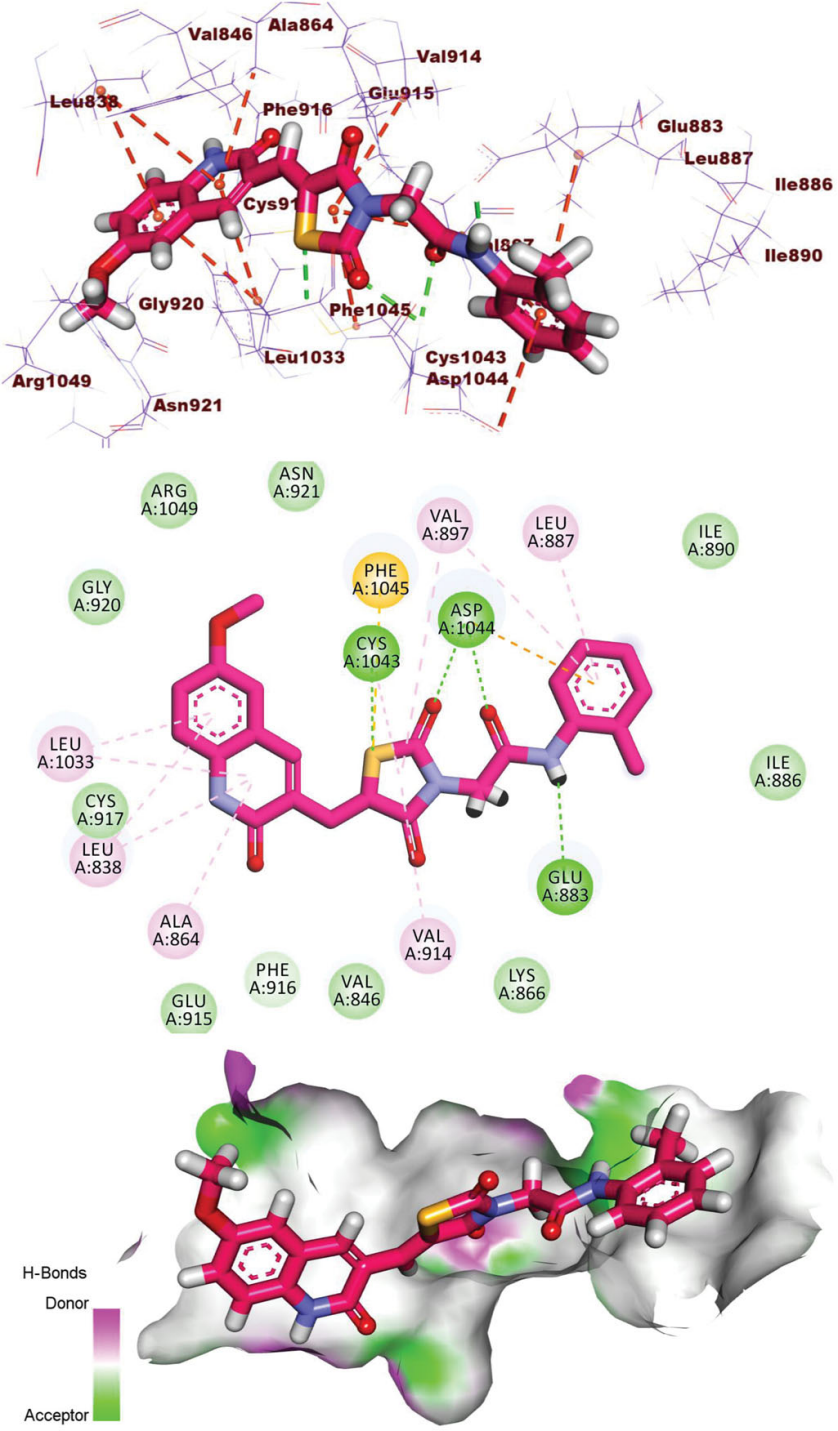
3D, 2D, and surface mapping of compound **9** into VEGFR-2. The hydrogen bonds were presented in green colour with Cys1043, Glu883, and Asp1044. The hydrophobic bonds were presented in orange colour with Ala864, Leu838, Leu1033, Val897, Val914, Phe1045, and Leu887.

Compound **9** showed docking energy of −21.53 kcal/mol. The quinolin-2(1*H*)-one moiety formed five H.Is in the hinge region with Ala864, Leu838, and Leu1033. The thiazolidine-2,4-dione (linker) moiety formed two extra H.Bs with Cys1043 and Asp1044 in addition to three hydrophobic bonds with Val897, Val914, and Phe1045. The pharmacophore (amide) moiety occupied the DFG region forming two H.Bs with Glu883 Asp1044. The terminal phenyl ring occupied the allosteric pocket forming two H.I with Leu887 and Val897 (Figure 11).

**Figure 12.**
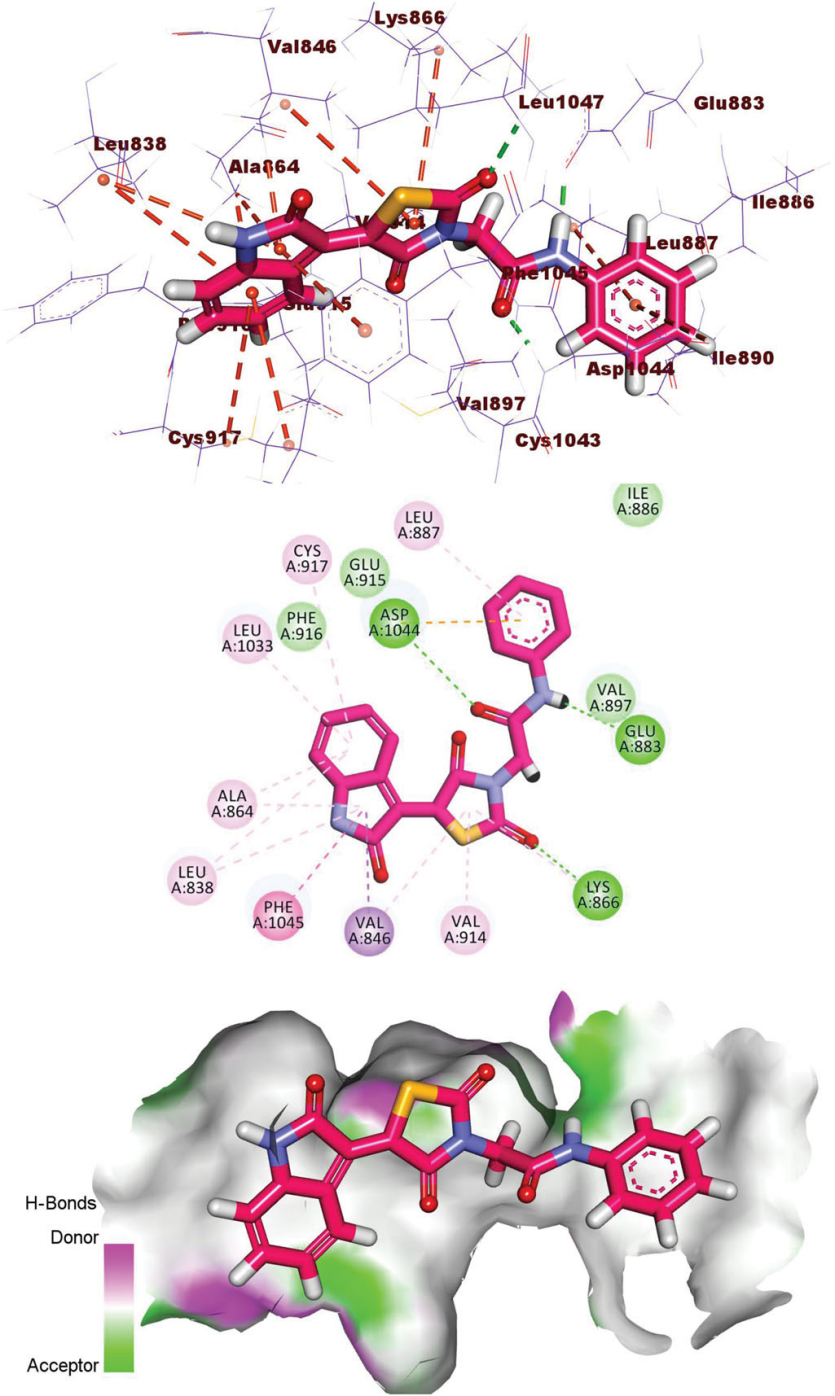
3D, 2D, and surface mapping of the compound **13** in the active site of VEGFR-2. The hydrogen bonds were presented in agreen colour with Lys866, Glu883 and Asp1044. The hydrophobic bonds were presented in orange colour with Cys917, Ala864, Leu838, Leu1033, Phe1045, Val846, Val914, and Leu887.

Compound **13** showed a good binding mode like that of sorafenib with a docking energy of −17.44 kcal/mol. The indolin-2-one moiety formed eight H.Is in the hinge region with Cys917, Ala864, Leu838, Leu1033, Phe1045, and Val846. The thiazolidine-2,4-dione (linker) moiety formed one H.B with Lys866, and two hydrophobic bonds with Val914, and Val846. The pharmacophore (amide) moiety occupied the DFG region forming two H.Bs with Glu883 Asp1044. The terminal phenyl ring occupied the allosteric pocket forming one H.I with Leu887 and one E.Iwith Asp1044 (Figure 12).

#### 2.3.2. *In silico* ADME analysis

**Figure 13.**
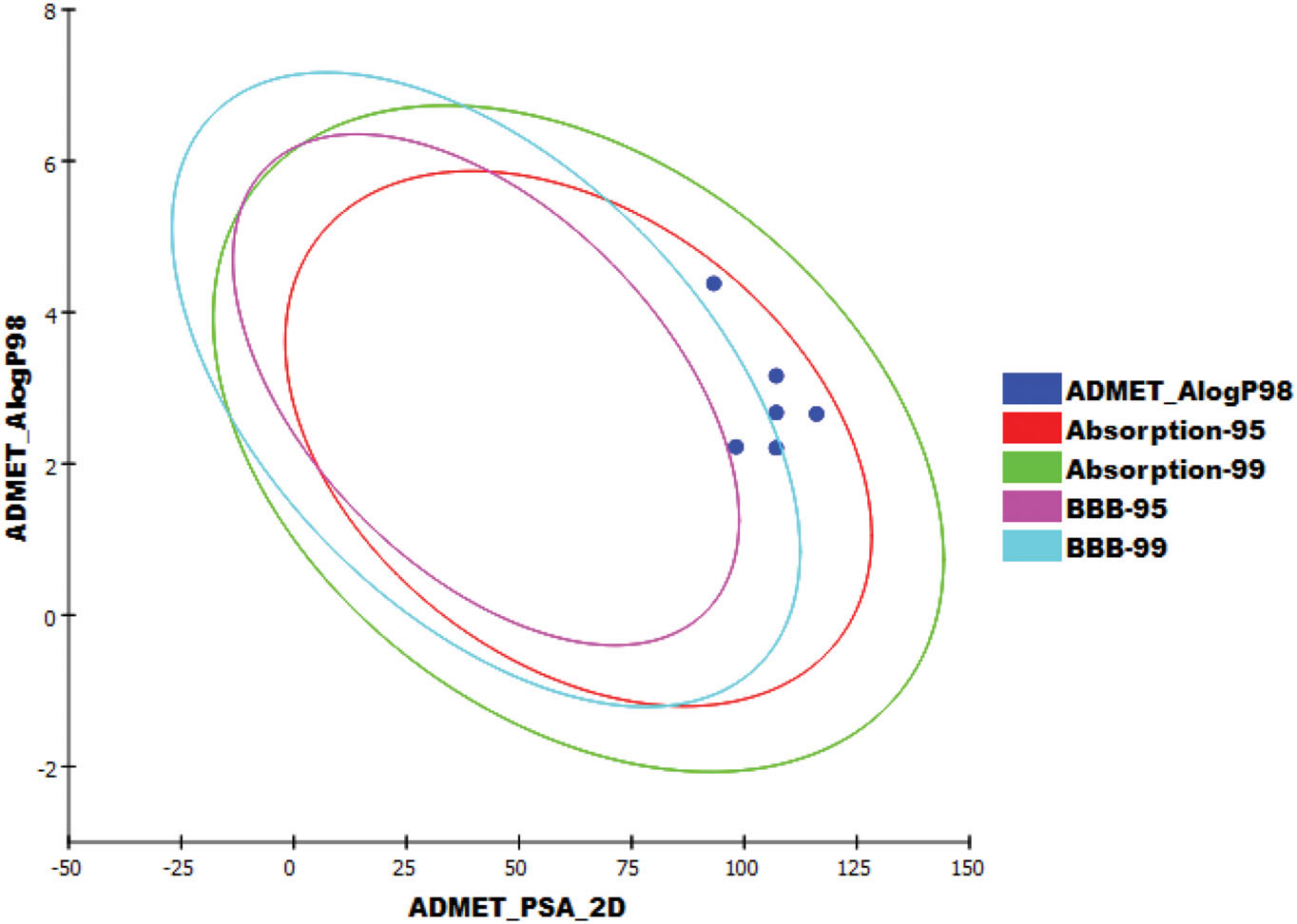
The ADMET plot of the considered compounds. Each componud is plotted with the 2 D polar surface area (PSA_2D) against the computed partition coefficient (ALogP98). The compound that is encompassed by the ellipse has good absorption and doesn’t violate of the ADMET properties. The ellipses (95% and 99% confidence limit)represent the blood–brain barrier penetration (BBB) and human intestinal absorption.

Discovery Studio 4.0 software was used to investigate ADMET parameters of the synthesised compounds utilising sorafenib as a reference. The results were summarised in Table 6. The tested compounds **7**, **8**, and **9** showed very low BBB penetration levels while compounds **13** and **14** exhibited low BBB penetration power. Hence, these compounds may be devoid of CNS toxicity. The aqueous solubility (A-S) of the tested compounds was predicted to be low while the intestinal absorption (I-A) levels were anticipated to be optimal. All examined compounds were expected to be non-inhibitors for the cytochrome P450 (CYP-2D6). So, the incidence of liver side effects is not expected upon their use. Except for compounds **8** and **14**, all the tested members were predicted to bind plasma protein more than 90% (Figure 13).

**Table 6.** ADMET screening of the synthesised compounds.

Compounds	BBB^a^	A-S^b^	I-A^c^	CYP-2D6^d^	PPB^e^
**7**	****	++	+	N-In	Mr
**8**	****	++	+	N-In	Ls
**9**	****	++	+	N-In	Mr
**13**	***	++	+	N-In	Mr
**14**	***	++	+	N-In	Ls
**Sorafenib**	****	+	+	N-In	Mr

^a^Very high (0), high (*), medium (**), low (***), very low (****).

^b^Optimal (++++), good (+++), low (++), very low (+).

^c^Good (+), moderate (++), poor (+++), or very poor (++++).

^d^Inhibitor (In) or non-inhibitor (N-In).

^e^PPB means plasma protein binding which may be less than 90% (Ls) or more than 90% (Mr).

#### 2.3.3. Toxicity studies

Discovery studio software version 4.0 was utilised to compute the predicted toxicity profile of the synthesised candidates as shown in Table 7.

**Table 7.** Toxicity study of the synthesised compounds

Compounds	Ames prediction	TD_50_^a^	R-MTD^b^	LD_50_^b^	LOAEL^b^	Skin irritancy	Ocular irritancy
**7**	Non-mutagen	83.279	0.021	0.899	0.005	None	Mild
**8**		37.833	0.021	1.320	0.003		
**9**		97.051	0.018	0.405	0.007		
**13**		74.651	0.048	1.404	0.040		
**14**		93.189	0.023	1.21	0.019		
**Sorafenib**		17.535	0.077	0.890	0.004		

^a^ Unit: mg/kg/day.

^b^ Unit: g/kg.

Starting with the Ames prediction model, all candidates were predicted to be non-mutagen. The carcinogenic potency TD_50_ in mice of the synthesised compounds ranged from 37.833 to 97.051 g/kg, which was safer than sorafenib (17.535 g/kg). The rat maximum tolerated doses (R-MTD) of these candidates were less than that of sorafenib **(**0.077 g/kg) with the range of 0.018 − 0.048 g/kg. Candidates **13** and **14** showed higher rat oral LD_50_ values of 1.404 and 1.21 g/kg, respectively than sorafenib (0.890 g/kg) while the other members showed lower oral LD_50_ values were in the range of 0.509–0.838 g/kg. For the rat chronic LOAEL model, except compound 8, the tested compounds showed LOAEL values in the range of 0.005–0.040 g/kg. These were safer than sorafenib (0.004 g/kg). All candidates were computed to be non-irritant and mildly irritant against the skin and the eyes, respictivly (Table 7).

#### 2.3.4. MD simulation

The Molecular dynamics (MD) simulations experiments are very close to being a routine computational approach in drug discovery^39^. There are two main strengths in the MD studies. Firstly, it can accurately examine both structural and entropic changes in both ligand and target. Secondly, it can track that changes over a definite time and every ultra-short period at an atomic resolution for ligand as well as protein target^40^. Accordingly, MD experiments can accurately estimate the thermodynamics as well as kinetics changes that are associated with ligand-protein binding^41^. These points implemented the MD simulations as a successful tool to examine the structure-function nature of the certain ligand-target complex. It identifies essential areas such as the stability of the certain ligand-target complex, ligand binding energy, and kinetics^42^.

**Figure 14.**
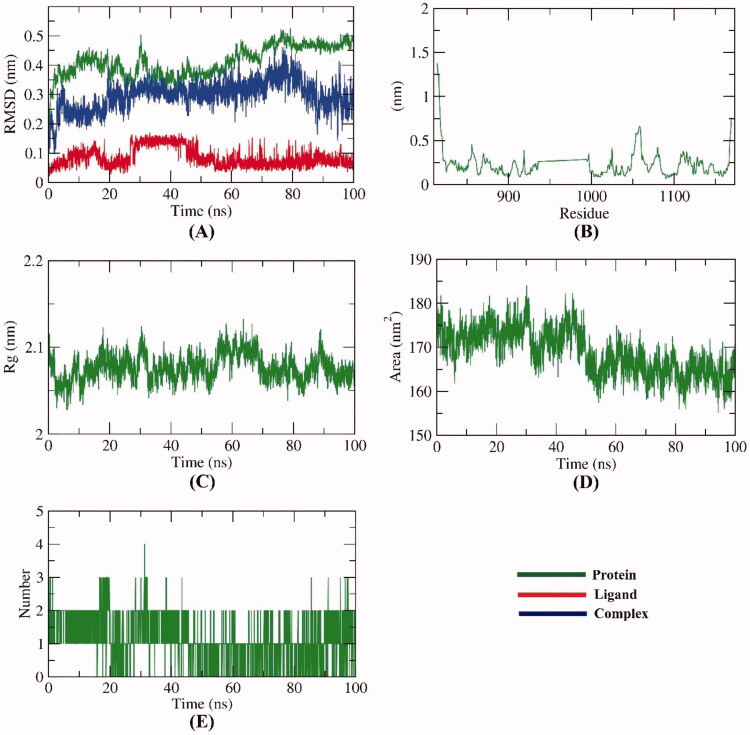
M D simulations; (A) RMSD, (B) RMSF (C) *R_g_* (D) SASA, and (E) H- bonding for compound **13**-VEGFR-2 complex over the MD run (100 ns).

First, the interaction of a compound with a protein’s active site results in structural changes in the protein^43^. Consequently, conformational changes, as well as dynamics of the compound **13**-VEGFR-2 complex, were studied as RMSD to understand stability after binding. The results (Figure 14(A)) demonstrated that the compound **13**-VEGFR-2 complex slightly fluctuated to 80 ns ∼ and got stabled in the last 20 ns of the MD run. The flexibility of the compound **13**-VEGFR-2 complex was examined by RMSF to predict the regions of changes of VEGFR-2 that were affected through the applied MD simulation experiment. Figure 14(B) demonstrates that the binding of compound **13** didn’t make the VEGFR-2 much more flexible. Based on the change in protein volume, *R_g_* identifies the 3 D changes of a protein besides its compactness, and the degree of fluctuation during the simulation time. The *R_g_* is inversely proportional to the stability and compactness of the system^44^^,^^45^. The computed *R_g_* values of the compound **13**-VEGFR-2 complex in the MD run (Figure 14(C)) remained slightly less than the starting time. Such results indicate the stability and compactness of the compound **13**-VEGFR-2 complex. As well as that, SASA calculations were used to determine the compound **13**-VEGFR-2 complex’s interaction with the solvents surrounding it. The resulting SASA values reveal how the complex’s conformation changed during the simulation study. Analogously, the SASA values of the compound **13**-VEGFR-2 complex were less than the starting period of expermint (Figure 14(D)), indicating that the surface area was reduced and therefore the stability of the compound **13**-VEGFR-2 complex was increased. H.Bing is an essential factor capable of stabilising a complex. Therefore, MD simulation experiments were allpied to explore the H.Bing through the compound **13**-VEGFR-2 complex. Figure 14(E) revealed that compound **13** formed up to two H.Bs with VEGFR-2.

**Figure 15.**
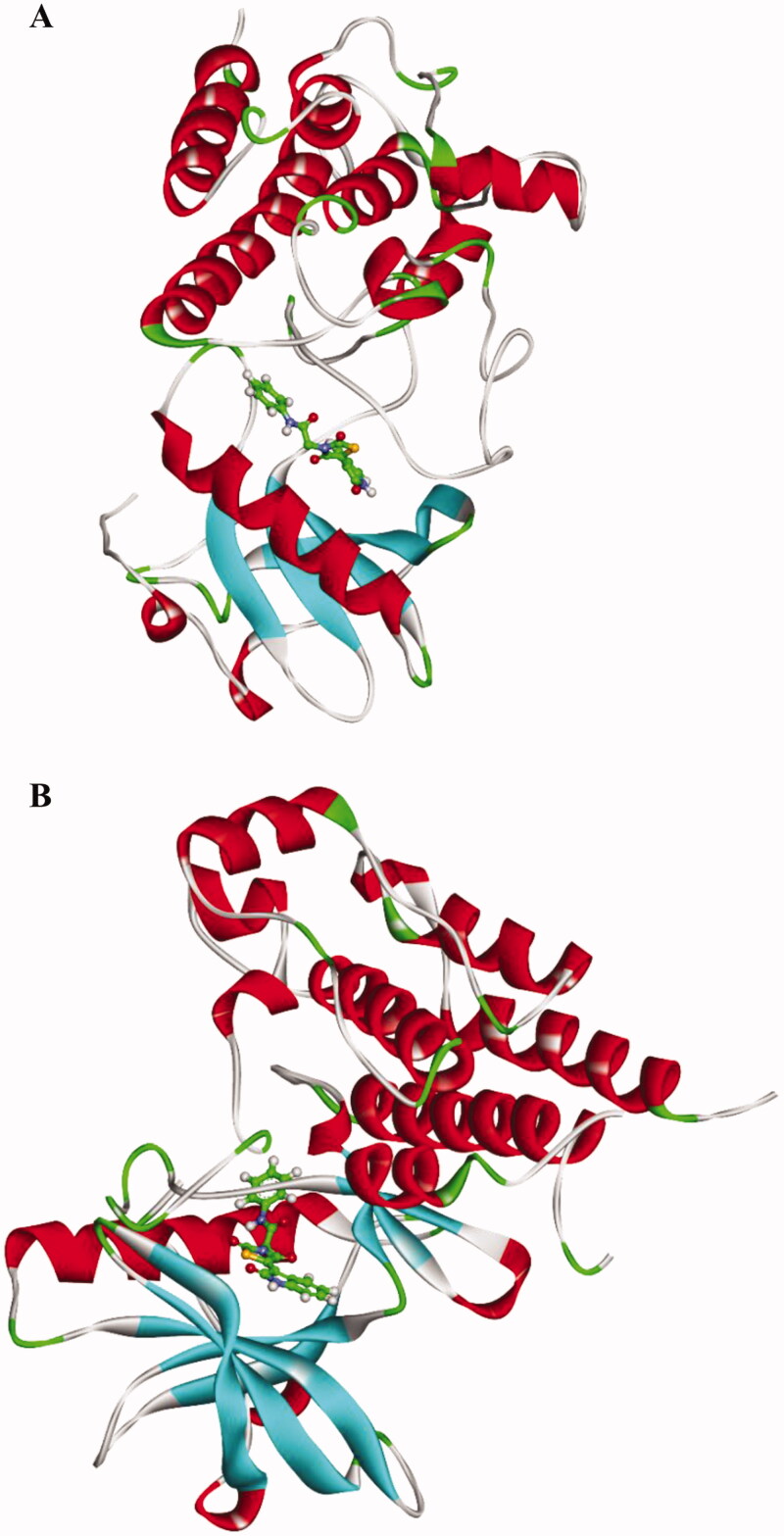
Compound **13**-VEGFR-2 complex structures at (A) 1 ns, (b) 100 ns.

As illustrated in Figure 15, the conformational change analysis of the compound **13**-VEGFR-2 complex was performed through the 1(Figure 15(A)), and 100 ns (Figure 15(B)) of the MD production in order to understand the changes caused by binding. The results indicated that minor conformational changes have taken place. Most importantly, compound **13** showed a high degree of binding stability and integrity inside VEGFR-2.

#### 2.3.5. MM-PBSA study

**Figure 16.**
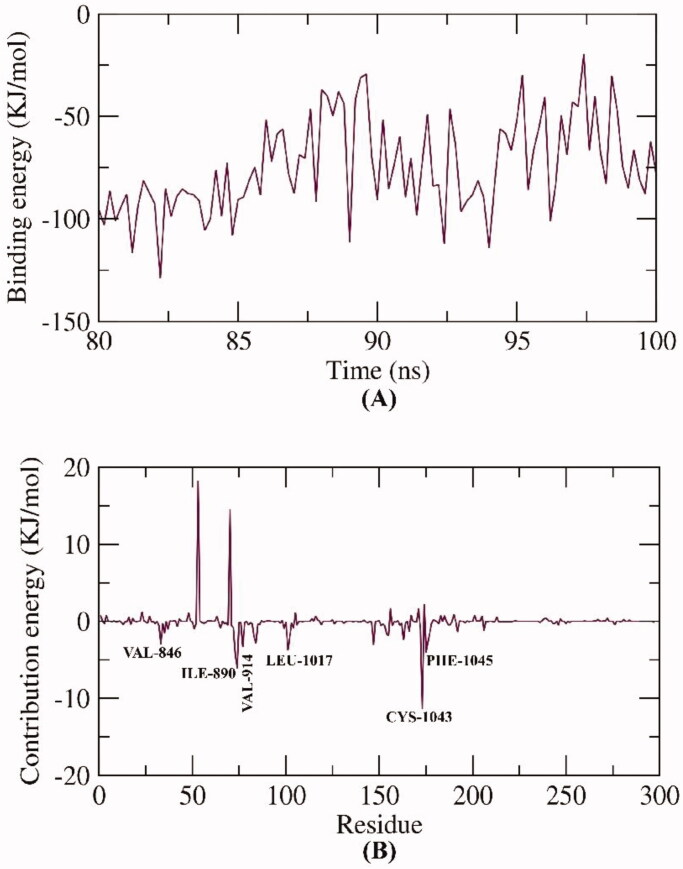
MM-PBSA outputs of the compound **13**-VEGFR-2 complex.

Using the MM/PBSA method to calculate the free binding energy from the MD trajectories through the last 20 ns of the MD run applying a 100 ps time interval of, compound **13** demonstrated a very low free binding energy of −74 KJ/mol with VEGFR-2. Interestingly, the binding energy remained stable throughout the entire 20 ns of analysis, showing the accurate binding of the compound **13**-VEGFR complex (Figure 16(A)).

Secondly, a total binding free energy analysis of the compound **13**-VEGFR-2 complex was performed (Figure 16(B)) to unravel the various components of the obtained binding energy, revealing the particular contributions of amino acids in VEGFR-2 to the binding process. Six residues (VAL-846, ILE-890, VAL-914, LEU-1017, CYS-1043 and PHE-1045) contributed higher binding energy than −4 KJ/mol and are considered key (vital) residues during binding with compound **13**.

## 3. Conclusion

In this work, five new quinoline and isatin derivatives were designed to possess the main features of VEGFR-2. These compounds were synthesised in good yields (74–88%) and confirmed using IR, ^1^H NMR, and ^13 ^C NMR. *In vitro* anti-proliferative activities were determined against four cancer cell lines (A549, Caco-2, HepG2, and MDA-MB-231). Compounds **13** (IC_50_ = 9.3 µM) and **14** (IC_50_ = 5.7 µM) showed comparable activity with doxorubicin (IC_50_ = 8.2 µM) against Caco-2 cells. Structure-activity relationship revealed that isatin derivatives (**13** and **14**) are higher cytotoxic agents than quinoline derivatives (**7**, **8**, and **9**) against three cell lines (A549, Caco-2, and MDA-MB-231). Furthermore, it was found that the phenyl ring is more advantageous as a hydrophobic tail than *p*-methoxyphenyl moiety, and the latter is more beneficial for activity than *o*-tolyl moiety. Compounds **13** and **14** exhibited strong inhibitory effects against VEGFR-2 with IC_50_ values of 69.11 and 85.89 nM, respectively. The selectivity index test revealed that compound **7** is the safest member. The wound healing assay for compound **7** exhibited the ability of such compound to prevent healing and migration in the cancer population. Compound **7** exhibited a significant down-regulation of Bcl2, Bcl-xl, and Survivin genes, and an upregulation of the TGF gene in Caco-2. The flowcytometric analysis confirmed the ability of compound **7** to arrest the cellular growth of Caco-2 in sub G0 (apoptotic phase). Computational studies (docking, ADMET, toxicity, and MD simulations) revealed the good binding mode of the synthesised compounds, an acceptable range of pharmacokinetic properties, and stability in the active site of VEGFR-2 at 100 ns.

## 4. Experimental

### 4.1. Chemistry

#### 4.1.1. General

All solvents, reagents, and devices were explained intensely in Supplementary data.

Compounds **2**, **5**, and **6** were obtained in accordance with the reported protocol^41–44^. The ^1^H NMR and ^13 ^C NMR analyses were carried out at 400 and 100 MHz, respectively in DMSO-d_6_ as a solvent. the chemical shifts were presented as ppm. The infra-red analyses were carried out using KBr disc and the results were presented as cm^−1^. Table 8 showed the colours, yields, and meting points of the target compounds

**Table 8.** Colours, yields, and meting points of the target compounds

Compounds	Colour	Yield (%)	Meting points (°C)
**7**	White crystals	87	260–262
**8**	Yellow crystals	88	257–259
**9**	White crystals	76	244–246
**13**	White crystals	87	249–251
**14**	White powder	74	265–267

#### 4.1.2. Synthesis of compounds 7, 8, and 9

Amixture of compound **6** (0.30 g, 0.001 mol) and anhydrous K_2_CO_3_(0.276 g, 0.002 mol) in DMF (30 ml) was heated in a water bath with the appropriate 2-chloroacetamide derivatives (0.001 mol) for a period of 8 h. Then, the reaction mixture was cooled and poured onto crushed ice. The obtained precipitate was filtered and recrystallized from absolute ethanol to afford compounds **7**, **8**, and **9**, respectively.

##### 4.1.2.1. (Z)-2–(5-((6-Methoxy-2-oxo-1,2-dihydroquinolin-3-yl)methylene)-2,4-dioxothiazolidin-3-yl)-N-phenylacetamide (7)


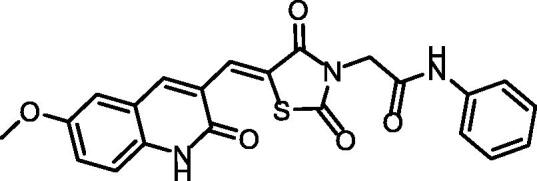


IR: 3282, 3141 (NH), 3001 (CH aromatic), 2922 (CH aliphatic), 1737, 1682 (C≡O); ^1^H NMR: 12.16 (s, 1H), 10.43 (s, 1H), 8.23 (s, 1H), 7.99 (s, 1H), 7.58 (d, *J* = 8.0 Hz, 2H), 7.41 (d, *J* = 2.6 Hz, 1H), 7.34 (t, *J* = 7.8 Hz, 3H), 7.30 (s, 1H), 7.28 (d, *J* = 2.6 Hz, 1H), 7.10 (t, *J* = 7.4 Hz, 1H), 4.53 (s, 2H), 3.83 (s, 3H); ^13 ^C NMR: 168.60, 166.16, 164.33, 160.57, 155.08, 142.56, 138.90, 134.39, 129.36, 129.18, 129.08, 127.19, 125.39, 124.17, 123.18, 120.12, 119.64, 117.12, 110.30, 56.03, 31.17; Anal. Calcd. For C_21_H_15_N_3_O_4_S (405.43).

##### 4.1.2.2. (Z)-2–(5-((6-Methoxy-2-oxo-1,2-dihydroquinolin-3-yl)methylene)-2,4-dioxothiazolidin-3-yl)-N-(4-methoxyphenyl)acetamide (8)


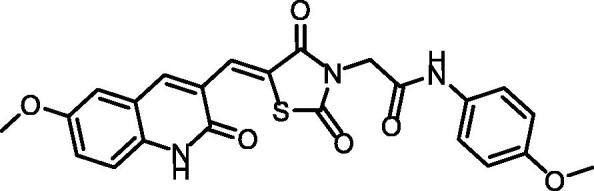


IR: 3267, 3145 (NH), 3067 (CH aromatic), 2977 (CH aliphatic), 1735, 1681 (C≡O); ^1^H NMR: 12.16 (s, 1H), 10.26 (s, 1H), 8.46 (s, 1H), 8.22 (s, 1H), 7.99 (s, 1H), 7.49 − 7.47 (m, 2H), 7.33 (d, *J* = 1.8 Hz, 2H), 6.94 − 6.88 (m, 2H), 4.49 (s, 2H), 3.83 (s, 3H), 3.74 (s, 3H); ^13 ^C NMR: 190.37, 161.55, 155.95, 154.99, 142.50, 142.23, 136.50, 134.37, 126.20, 124.16, 121.19, 119.17, 117.28, 114.45, 111.59, 56.04, 55.64, 31.17; Anal. Calcd. For C_22_H_17_N_3_O_5_S (435.45).

##### 4.1.2.3. (Z)-2–(5-((6-Methoxy-2-oxo-1,2-dihydroquinolin-3-yl)methylene)-2,4-dioxothiazolidin-3-yl)-N-(o-tolyl)acetamide (9)


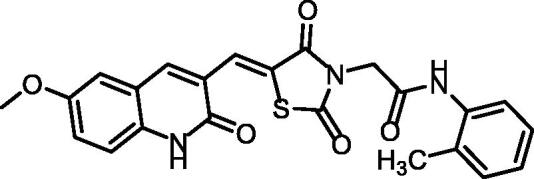


IR: 3254, 3224 (NH), 2991 (CH aromatic), 2907 (CH aliphatic), 1722, 1668 (C≡O); ^1^H NMR: 12.15 (s, 1H, NH), 10.24 (s, 1H, NH), 8.43 (s, 1H, H-4, quinolinone), 7.98 (s, 1H, C = CH), 7.45 (m, 1H, 1H, H-8, quinolinone), 7.38 − 7.25 (m, 3H, Ar-H), 7.18 − 7.10 (m, 3H, Ar-H), 6.95 (m, 1H), 4.55 (s, 2H, CH_2_), 3.35(s, 3H, OCH_3_), 2.23 (s, 3H, CH_3_); ^13 ^C NMR: 171.60, 164.59, 163.70, 161.55, 142.24, 137.75, 136.59, 134.39, 131.73, 131.02 (2), 129.43 (2), 129.31 (2), 126.98 (2), 124.16, 117.28, 111.59, 56.04, 46.73, 17.74; Anal. Calcd. For C_22_H_17_N_3_O_4_S (419.46).

#### 4.1.3. Synthesis of compounds 13 and 14

A mixture of **12** (0.28 g, 0.001 mol), the appropriate 2-chloroacetamide derivatives (0.001 mol) namely, 2-chloro-*N*-phenylacetamide and 2-chloro-*N*-(4-methoxyphenyl) acetamide and KI (0.067 g) in DMF (50 ml) was heated using a water bath for a period of 8 h. Then, cooled and poured onto crushed ice. The obtained precipitate was filtered and recrystallized from absolute ethanol to afford the corresponding compounds **13** and **14** respectively.

##### 4.1.3.1. 2-[2,4-Dioxo-5–(3-oxoindolin-2-ylidene)thiazolidin-3-yl]-N-phenylacetamide (13)


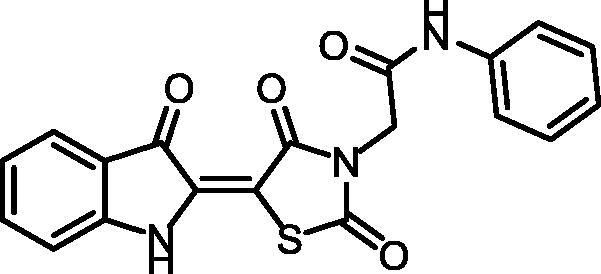


IR: 3293, 3175 (NH), 3060 (CH aromatic), 2943 (CH aliphatic), 1745, 1693 (C≡O); ^1^H NMR: 11.34 (s, 1H, NH), 10.49 (s, 1H, NH), 8.79 (s, 1H, Ar-H), 7.59 (d, *J* = 7.20 Hz, 2H, Ar-H), 7.36 (m, 1H, Ar-H), 7.34 (m, 2H, Ar-H), 7.11–7.10 (m, 2H, Ar-H), 6.99 (d, 1H, Ar-H), 4.59 (s, 2H, CH_2_); ^13 ^C NMR: 172.51, 170.24, 168.72, 165.71, 164.24, 144.64, 138.87, 133.56, 129.37(2), 128.43, 128.21, 127.19, 124.22, 122.66, 120.23, 119.68, 111.16, 44.13; Anal. Calcd. For C_19_H_13_N_3_O_4_S (379.39).

##### 4.1.3.2. 2–(2,4-Dioxo-5–(3-oxoindolin-2-ylidene)thiazolidin-3-yl)-N-(4-methoxyphenyl) acetamide 14


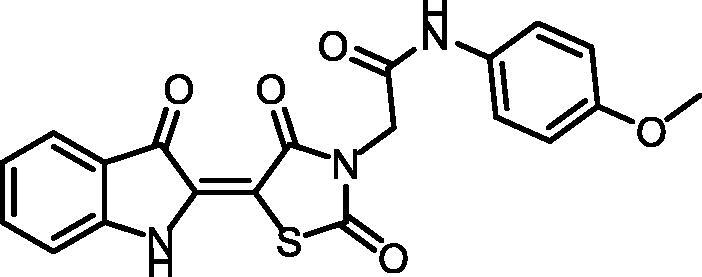


IR: 3269, 3274 (NH), 3059 (CH aromatic) 1744, 1691 (C = O); ^1^H NMR: 11.31 (s, 1H), 10.28 (s, 1H), 8.77 (d, *J* = 8.0 Hz, 1H), 7.49 (d, *J* = 8.6 Hz, 2H), 7.46 − 7.35 (m, 1H), 7.08 (t, *J* = 7.8 Hz, 1H), 6.98 (d, *J* = 7.9 Hz, 1H), 6.91 (d, *J* = 8.5 Hz, 2H), 4.54 (s, 2H), 3.73 (s, 3H); ^13 ^C NMR: 170.26, 168.70, 165.69, 163.71, 155.99, 144.61, 133.52, 131.96, 129.40, 128.42, 128.16, 122.64, 121.25, 120.22, 114.45, 111.13, 55.63, 44.01.

### 4.2. Biological testing

#### 4.2.1. *In vitro* anti-proliferative activity

Anti-proliferative activities were assessed using the MTT assay^31^^,^^46^ and were explained intensely in Supplementary data.

#### 4.2.2. *In vitro* VEGFR-2 kinase assay

Was tested using a VEGFR-2 ELISA kit and was explained intensely in Supplementary data^47^.

#### 4.2.3. Safety assay

The safety profiles were examined on Vero cells (non-cancerous cell line) and was explained intensely in Supplementary data^48^.

#### 4.2.4. Selectivity index (SI)

Was calculated and explained intensely in Supplementary data^49^.

##### 4.2.4.1. Cell Migration assay

Was performed as the described protocol^50^ and was explained intensely in Supplementary data.

##### 4.2.4.2. Gene expression pattern

Bcl2, Bcl-xl, TGF and Survivin genes levels were evaluated as reported^51^ and was explained intensely in Supplementary data.

### 4.3. *In silico* studies

#### 4.3.1. Docking studies

Were carried out using MOE software^52^ and were explained intensely in Supplementary data.

#### 4.3.2. ADMET studies

Were determined using Discovery studio 4.0 as reported method^53^ and were explained intensely in Supplementary data.

#### 4.3.3. Toxicity studies

Were calculated using Discovery studio 4.0 as described^54^ and were explained intensely in Supplementary data.

#### 4.3.4. Molecular dynamics simulation

MD studies were performed through CHARMM-GUI interface^55–57^ using CHARMM36 force field^58^ and NAMD 2.13 package^59^ as explained intensely in Supplementary data.

#### 4.3.5. MM-PBSA studies

Was performed using **MM-PBSA** package of GROMACS and was explained intensely in Supplementary data.

## Supplementary Material

Supplemental Material

## References

[1] WHO Cancer. Key facts. https://www.who.int/news-room/fact-sheets/detail/cancer [last accessed 26 Jan 2022].

[2] NHS Cancer. Overview. https://www.nhs.uk/conditions/cancer/ [last accessed 27 Jan 2022].

[3] Wong MC, Huang J, Lok V, et al. Differences in incidence and mortality trends of colorectal cancer worldwide based on sex, age, and anatomic location. Clin Gastroenterol Hepatol 2021;19:955–66. e61. 32088300 10.1016/j.cgh.2020.02.026

[4] Bray F, Ferlay J, Soerjomataram I, et al. Global cancer statistics 2018: GLOBOCAN estimates of incidence and mortality worldwide for 36 cancers in 185 countries. CA Cancer J Clin 2018;68:394–424. 30207593 10.3322/caac.21492

[5] Meier P, Finch A, Evan G. Apoptosis in development. Nature 2000;407:796–801. 11048731 10.1038/35037734

[6] Lowe SW, Lin AW. Apoptosis in cancer. Carcinogenesis 2000;21:485–95. 10688869 10.1093/carcin/21.3.485

[7] Fernald K, Kurokawa M. Evading apoptosis in cancer. Trends Cell Biol 2013;23:620–33. 23958396 10.1016/j.tcb.2013.07.006 PMC4091735

[8] Farghaly TA, Al-Hasani WA, Abdulwahab HG. An updated patent review of VEGFR-2 inhibitors (2017-present). Exp Opin Ther Pat 2021;31:989–1007. (just-accepted). 10.1080/13543776.2021.1935872 34043477

[9] Li Y, Zhang F, Nagai N, et al. VEGF-B inhibits apoptosis via VEGFR-1–mediated suppression of the expression of BH3-only protein genes in mice and rats. J Clin Invest 2008;118:913–23. 18259607 10.1172/JCI33673 PMC2230661

[10] Xiao X, Wu J, Zhu X, et al. Induction of cell cycle arrest and apoptosis in human nasopharyngeal carcinoma cells by ZD6474, an inhibitor of VEGFR tyrosine kinase with additional activity against EGFR tyrosine kinase. Int J Cancer 2007;121:2095–104. 17631646 10.1002/ijc.22955

[11] Otrock ZK, Mahfouz RA, Makarem JA, Shamseddine AI. Understanding the biology of angiogenesis: review of the most important molecular mechanisms. Blood Cells Mol Dis 2007;39:212–20. 17553709 10.1016/j.bcmd.2007.04.001

[12] Ferrara N, Gerber H-P, LeCouter J. The biology of VEGF and its receptors. Nat Med 2003;9:669–76. 12778165 10.1038/nm0603-669

[13] Dvorak HF. Vascular permeability factor/vascular endothelial growth factor: a critical cytokine in tumor angiogenesis and a potential target for diagnosis and therapy. J Clin Oncol 2002;20:4368–80. 12409337 10.1200/JCO.2002.10.088

[14] Cross MJ, Dixelius J, Matsumoto T, Claesson-Welsh L. VEGF-receptor signal transduction. Trends Biochem Sci 2003;28:488–94. 13678960 10.1016/S0968-0004(03)00193-2

[15] Marrone TJ, Briggs a, James M, McCammon JA. Structure-based drug design: computational advances. Annu Rev Pharmacol Toxicol 1997;37:71–90. 9131247 10.1146/annurev.pharmtox.37.1.71

[16] Li N, Wang Y, Li W, et al. Screening of some sulfonamide and sulfonylurea derivatives as anti-Alzheimer’s agents targeting BACE1 and PPARγ. J Chem 2020;2020:1–19.

[17] Abdel-Aziz HA, Eldehna WM, Fares M, et al. Synthesis, biological evaluation and 2D-QSAR study of halophenyl bis-hydrazones as antimicrobial and antitubercular agents. Int J Mol Sci 2015;16:8719–43. 25903147 10.3390/ijms16048719 PMC4425105

[18] Parmar DR, Soni JY, Guduru R, et al. Discovery of new anticancer thiourea-azetidine hybrids: design, synthesis, *in vitro* antiproliferative, SAR, in silico molecular docking against VEGFR-2, ADMET, toxicity, and DFT studies. Bioorg Chem 2021;115:105206. 34339975 10.1016/j.bioorg.2021.105206

[19] Eissa IH, Ibrahim MK, Metwaly AM, et al. Design, molecular docking, *in vitro*, and *in vivo* studies of new quinazolin-4 (3H)-ones as VEGFR-2 inhibitors with potential activity against hepatocellular carcinoma. Bioorg Chem. 2021;107:104532. 33334586 10.1016/j.bioorg.2020.104532

[20] Alanazi MM, Eissa IH, Alsaif NA, et al. Design, synthesis, docking, ADMET studies, and anticancer evaluation of new 3-methylquinoxaline derivatives as VEGFR-2 inhibitors and apoptosis inducers. J Enzyme Inhib Med Chem 2021;36:1760–82. 34340610 10.1080/14756366.2021.1956488 PMC8344243

[21] El-Metwally SA, Abou-El-Regal MM, Eissa IH, et al. Discovery of thieno [2, 3-d] pyrimidine-based derivatives as potent VEGFR-2 kinase inhibitors and anti-cancer agents. Bioorg Chem 2021;112:104947. 33964580 10.1016/j.bioorg.2021.104947

[22] Lee K, Jeong K-W, Lee Y, et al. Pharmacophore modeling and virtual screening studies for new VEGFR-2 kinase inhibitors. Eur J Med Chem 2010;45:5420–7. 20869793 10.1016/j.ejmech.2010.09.002

[23] Machado VA, Peixoto D, Costa R, et al. Synthesis, antiangiogenesis evaluation and molecular docking studies of 1-aryl-3-[(thieno [3, 2-b] pyridin-7-ylthio) phenyl] ureas: discovery of a new substitution pattern for type II VEGFR-2 Tyr kinase inhibitors. Bioorg Med Chem 2015;23:6497–509. 26344591 10.1016/j.bmc.2015.08.010

[24] Dietrich J, Hulme C, Hurley LH. The design, synthesis, and evaluation of 8 hybrid DFG-out allosteric kinase inhibitors: a structural analysis of the binding interactions of Gleevec^®^, Nexavar^®^, and BIRB-796. Bioorg Med Chem 2010;18:5738–48. 20621496 10.1016/j.bmc.2010.05.063

[25] Viegas-Junior C, Danuello A, da Silva Bolzani V, et al. Molecular hybridization: a useful tool in the design of new drug prototypes. Curr Med Chem 2007;14:1829–52. 17627520 10.2174/092986707781058805

[26] Meth-Cohn O, Narine B, Tarnowski B. A versatile new synthesis of quinolines and related fused pyridines, Part 5. The synthesis of 2-chloroquinoline-3-carbaldehydes. J Chem Soc Perkin Trans 1 1981;1520–30.

[27] Kar K, Krithika U, Basu P, et al. Design, synthesis and glucose uptake activity of some novel glitazones. Bioorg Chem 2014;56:27–33. 24927033 10.1016/j.bioorg.2014.05.006

[28] Shih M-H, Yeh M-Y. Access to the syntheses of sydnonyl-substituted α, β-unsaturated ketones and 1, 3-dihydro-indol-2-ones by modified Knoevenagel reaction. Tetrahedron 2003;59:4103–11.

[29] Mosmann T. Rapid colorimetric assay for cellular growth and survival: application to proliferation and cytotoxicity assays. J Immunol Methods 1983;65:55–63. 6606682 10.1016/0022-1759(83)90303-4

[30] Denizot F, Lang R. Rapid colorimetric assay for cell growth and survival: modifications to the tetrazolium dye procedure giving improved sensitivity and reliability. J Immunol Methods 1986;89:271–7. 3486233 10.1016/0022-1759(86)90368-6

[31] Thabrew M, Hughes RD, Mcfarlane IG. Screening of hepatoprotective plant components using a HepG2 cell cytotoxicity assay. J Pharm Pharmacol 2011;49:1132–5. 10.1111/j.2042-7158.1997.tb06055.x 9401951

[32] Pritchett JC, Naesens L, Montoya J. Treating HHV-6 infections: the laboratory efficacy and clinical use of anti-HHV-6 agents. In: Flamand L, Lautenschlager I, Krueger G, Ablashi D, ed. Human herpesviruses HHV-6A, HHV-6B & HHV-7. 3rd ed. Amsterdam, Netherlands: Elsevier; 2014.

[33] Peña-Morán OA, Villarreal ML, Álvarez-Berber L, et al. Cytotoxicity, post-treatment recovery, and selectivity analysis of naturally occurring podophyllotoxins from *Bursera fagaroides* var. fagaroides on breast cancer cell lines. Molecules. 2016;21:1013. 10.3390/molecules21081013 PMC6274026 27527135

[34] Indrayanto G, Putra GS, Suhud F. Excipients, R. Methodology, Validation of in-vitro bioassay methods: application in herbal drug research. Profiles Drug Subst Excip Relat Methodol. 2021;46:273–307. 33461699 10.1016/bs.podrm.2020.07.005

[35] Liang C-C, Park AY, Guan J-L. *In vitro* scratch assay: a convenient and inexpensive method for analysis of cell migration *in vitro*. Nat Protocols 2007;2:329–33. 17406593 10.1038/nprot.2007.30

[36] Kim R. Unknotting the roles of Bcl-2 and Bcl-xL in cell death. Biochem Biophys Res Commun 2005;333:336–43. 15922292 10.1016/j.bbrc.2005.04.161

[37] Yang J, Song K, Krebs TL, et al. Rb/E2F4 and Smad2/3 link survivin to TGF-β-induced apoptosis and tumor progression. Oncogene 2008;27:5326–38. 18504435 10.1038/onc.2008.165 PMC2762862

[38] Liu Y, Gray NS. Rational design of inhibitors that bind to inactive kinase conformations. Nat Chem Biol 2006;2:358–64. 16783341 10.1038/nchembio799

[39] Sousa SF, Fernandes PA, Ramos MJ. Protein–ligand docking: current status and future challenges. Proteins 2006;65:15–26. 16862531 10.1002/prot.21082

[40] Hollingsworth SA, Dror RO. Molecular dynamics simulation for all. Neuron 2018;99:1129–43. 30236283 10.1016/j.neuron.2018.08.011 PMC6209097

[41] Hansson T, Oostenbrink C, van Gunsteren W. Molecular dynamics simulations. Current Opin Struct Biol 2002;12:190–6. 10.1016/s0959-440x(02)00308-1 11959496

[42] Durrant JD, McCammon JA. Molecular dynamics simulations and drug discovery. BMC Biol 2011;9:71–9. 22035460 10.1186/1741-7007-9-71 PMC3203851

[43] Kuzmanic A, Zagrovic B. Determination of ensemble-average pairwise root mean-square deviation from experimental B-factors. Biophys J 2010;98:861–71. 20197040 10.1016/j.bpj.2009.11.011 PMC2830444

[44] Liu P, Lu J, Yu H, et al. Lubricant shear thinning behavior correlated with variation of radius of gyration via molecular dynamics simulations. J Chem Phys 2017;147:084904. 28863549 10.1063/1.4986552

[45] Kumar K, Anbarasu A, Ramaiah S. Molecular docking and molecular dynamics studies on β-lactamases and penicillin binding proteins. Mol BioSyst 2014;10:891–900. 24503740 10.1039/c3mb70537d

[46] El-Deeb NM, Ibrahim OM, Mohamed MA, et al. Alginate/κ-carrageenan oral microcapsules loaded with *Agaricus bisporus* polysaccharides MH751906 for natural killer cells mediated colon cancer immunotherapy. Int J Biol Macromol 2022;205:385–95. 35183600 10.1016/j.ijbiomac.2022.02.058

[47] Abou-Seri SM, Eldehna WM, Ali MM, Abou El Ella DA. 1-Piperazinylphthalazines as potential VEGFR-2 inhibitors and anticancer agents: synthesis and *in vitro* biological evaluation. Eur J Med Chem 2016;107:165–79. 26590508 10.1016/j.ejmech.2015.10.053

[48] Borenfreund E, Puerner JA. Toxicity determined *in vitro* by morphological alterations and neutral red absorption. Toxicol Lett 1985;24:119–24. 3983963 10.1016/0378-4274(85)90046-3

[49] Koch A, Tamez P, Pezzuto J, Soejarto D. Evaluation of plants used for antimalarial treatment by the Maasai of Kenya. J Ethnopharmacol 2005;101:95–9. 15878245 10.1016/j.jep.2005.03.011

[50] Arranz-Valsero I, Soriano-Romaní L, García-Posadas L, et al. IL-6 as a corneal wound healing mediator in an *in vitro* scratch assay. Exp Eye Res 2014;125:183–92. 24971496 10.1016/j.exer.2014.06.012

[51] Zucchini N, de Sousa G, Bailly-Maitre B, et al. Regulation of Bcl-2 and Bcl-xL anti-apoptotic protein expression by nuclear receptor PXR in primary cultures of human and rat hepatocytes. Biochim Biophys Acta Mol Cell Res 2005;1745:48–58. 10.1016/j.bbamcr.2005.02.005 16085054

[52] Ibrahim MK, Eissa IH, Abdallah AE, et al. Design, synthesis, molecular modeling and anti-hyperglycemic evaluation of novel quinoxaline derivatives as potential PPARγ and SUR agonists. Biorg Med Chem 2017;25:1496–513. 10.1016/j.bmc.2017.01.015 28117121

[53] Suleimen YM, Jose RA, Suleimen RN, et al. Jusanin, a new flavonoid from artemisia commutata with an in silico inhibitory potential against the SARS-CoV-2 main protease. Molecules 2022;27:1636. 35268738 10.3390/molecules27051636 PMC8911936

[54] Mohammed SO, El Ashry ESH, Khalid A, et al. Expression, purification, and comparative inhibition of helicobacter pylori urease by regio-selectively alkylated benzimidazole 2-thione derivatives. Molecules 2022;27:865. 35164122 10.3390/molecules27030865 PMC8838460

[55] Jo S, Kim T, Iyer VG, Im W. CHARMM-GUI: a web-based graphical user interface for CHARMM. J Comput Chem 2008;29:1859–65. 18351591 10.1002/jcc.20945

[56] Brooks BR, Brooks CL, III, Mackerell AD, Jr, et al. CHARMM: the biomolecular simulation program. J Comput Chem 2009;30:1545–614. 19444816 10.1002/jcc.21287 PMC2810661

[57] Lee J, Cheng X, Swails JM, et al. CHARMM-GUI input generator for NAMD, GROMACS, AMBER, OpenMM, and CHARMM/OpenMM simulations using the CHARMM36 additive force field. J Chem Theor Comput 2016;12:405–13. 10.1021/acs.jctc.5b00935 PMC4712441 26631602

[58] Best RB, Zhu X, Shim J, et al. Optimization of the additive CHARMM all-atom protein force field targeting improved sampling of the backbone phi, psi and side-chain chi(1) and chi(2) dihedral angles. J Chem Theor Comput 2012;8:3257–73. 10.1021/ct300400x PMC3549273 23341755

[59] Phillips JC, Braun R, Wang W, et al. Scalable molecular dynamics with NAMD. J Comput Chem 2005;26:1781–802. 16222654 10.1002/jcc.20289 PMC2486339

