# Supplementary material for: New quinoline and isatin derivatives as apoptotic VEGFR-2 inhibitors: design, synthesis, anti-proliferative activity, docking, ADMET, toxicity, and MD simulation studies
Source: J Enzyme Inhib Med Chem. 2022 Aug 16;37(1):2191–205. doi: 10.1080/14756366.2022.2110869 (PMC9387325; doi:10.1080/14756366.2022.2110869)
Supplement: Supplemental Material [file IENZ_A_2110869_SM6829.pdf]

New quinoline and isatine derivatives as apoptotic VEGFR-2 inhibitors: Design, synthesis, anti-proliferative activity, docking, ADMET, toxicity, and MD simulation studies

Eslam B. Elkaeed[a], Mohammed S. Taghour[b], Hazem. A. Mahdy[b], Wagdy M. Eldehna[c], Nehal El-Deeb[d,e], Ahmed M. Kenawy[f], Bshra A. Alsfouk[g], Mohammed A. Dahab[b], Ahmed M. Metwaly[h,i], Ibrahim. H. Eissa[b]*, Mohamed A. El-Zahabia[b]*

[a] Department of Pharmaceutical Sciences, College of Pharmacy, AlMaarefa University, Riyadh 13713, Saudi Arabia.

[b] Pharmaceutical Medicinal Chemistry & Drug Design Department, Faculty of Pharmacy (Boys), Al-Azhar University, Cairo11884, Egypt.

[c] Department of Pharmaceutical Chemistry, Faculty of Pharmacy, Kafrelsheikh University, P.O. Box 33516, Kafrelsheikh, Egypt.

[d] Biopharmaceutical Products Research Department, Genetic Engineering and Biotechnology Research Institute, City of Scientific Research and Technological Applications (SRTA-City), Alexandria 21934, Egypt.

[e] Pharmaceutical and Fermentation Industries Development Center, City of Scientific Research and Technological Applications (SRTA city), Alexandria, New Borg El-Arab City, 21934, Egypt

[f] Nucleic Acids Research Department, Genetic Engineering and Biotechnology Research Institute. City of Scientific Research and Technological Applications (SRTA-City). Alexandria 21934, Egypt

[g] Department of Pharmaceutical Sciences, College of Pharmacy, Princess Nourah bint Abdulrahman University, P.O. Box 84428, Riyadh 11671, Saudi Arabia.

[h] Pharmacognosy and Medicinal Plants Department, Faculty of Pharmacy (Boys), Al-Azhar University, Cairo 11884, Egypt.

[i] Biopharmaceutical Products Research Department, Genetic Engineering and Biotechnology Research Institute, City of Scientific Research and Technological Applications (SRTA-City), Alexandria, Egypt.

***Corresponding authors:**

Ibrahim H. Eissa

Pharmaceutical Medicinal Chemistry & Drug Design Department, Faculty of Pharmacy (Boys), Al-Azhar University, Cairo11884, Egypt. **Email:** Ibrahimeissa@azhar.edu.eg

Mohamed A. El-Zahabi

Pharmaceutical Medicinal Chemistry & Drug Design Department, Faculty of Pharmacy (Boys), Al-Azhar University, Cairo11884, Egypt. **Email:** malzahaby@yahoo.com

Content

1	**Biological testing**<br>✓ *In vitro* anti-proliferative activity.<br>✓ *In vitro* VEGFR-2 kinase assay.<br>✓ Safety assay<br>✓ Selectivity index (SI)<br>✓ Wound healing assay (Migration assay).<br>✓ Gene expression pattern of compound **7** in cancer cells.
2	***In silico* studies**<br>✓ Docking studies<br>✓ ADMET studies<br>✓ Toxicity studies<br>✓ MD simulation<br>✓ MMPBSA
3	**Chemistry and materials**
4	**Spectral data**

1- Biological testing

a- Mammalian cell lines culture

Lung carcinoma epithelial (A549), colon cancer (Caco-2)cell lines were cultured on DMEM media, meanwhile hepatocellular cancer (HepG2), and breast cancer (MDA-MB-231) cell lines were cultured on RBMI media. The cultured media were supplemented with 200 mM L-glutamine, 10.0% fetal bovine serum (Lonza), and 1.0% penicillin/streptomycin. Cells were seeded into 25.0 cm tissue culture flasks and incubated at 37°C in a 5.0% CO_2 incubator for 24 h or till confluency.

b- Safety assay

The safety profiles of the tested compounds were checked on one non-cancerous cell line (Vero) to determine the treatments concentrations that do not depict toxic effects against the tested cells. A portion of 100.0 µl of 6×10^4 cell/ml cells was seeded into each well of a 96-well plate and then the plates were incubated at 37°C in a humidified 5.0% CO_2 incubator for 24 h. At the end of incubation period, the exhausted medium was replaced with 100.0 µl of different concentrations of the designated treatment (prepared in RPMI medium starting from 1.0 mM). The inoculated plates were incubated at the same growth conditions for another 24 h. At the end of incubation, cellular viability was assessed using MTS assay kit (Promega) according to the manual instruction.

c- *In-vitro* anticancer activity

Anticancer activities of the tested compounds against lung carcinoma epithelial (A549), colon cancer (Caco-2), hepatocellular cancer (HepG2), and breast cancer (MDA-MB-231) cell lines were quantified using MTS assay kit (Promega) as described by the Manufacturer.

d- Selectivity index (SI)

The selectivity index values of the tested compounds on cancer cells were calculated as described by Koch et al. [57], with slight modifications; SI=IC_{50}nc/IC_{50}cc, where IC_{50}nc: the IC_{50} value of the tested compound on normal cells and IC_{50}cc: IC_{50} of the tested compound on cancer cell line**.**

e- Wound healing assay (Migration assay)

CaCo-2 cells were grown to 95.0% confluency in a complete DMEM medium and then the wounds were formed using a plastic tip. After washing with pre-warmed PBS, the cells were incubated in the specific medium or the **7** treatment. After incubation at 37°C and 5.0% CO_2 for 24h, the cells were washed with PBS and the wounds distance was determined as the scratch width of the treated and untreated groups using ImageJ software.

f- **Gene expression pattern alternation of cancer cell after treatment with compound 7.**

The molecular anticancer mode of action of **7** was investigated by screening their ability to affect the gene expression levels of Bcl2, Bcl-xl, TGF and Survivin genes using specific forward and reverse primers and RTq-PCR technique (Table 1) in CaCo-2 cells (chosen as the most sensitive cancer cell line). After cellular treatment, CaCo-2 cell line was cultured into 12 well plates (6×10^3 cell/ml) for 24 h. with the sub-IC50 concentration of **7**. After treatment, total RNA extraction was performed using RNA extraction kit (Qiagen, Germany). Then, 1 ug of the obtained RNA was used to synthesiz cDNA using cDNA synthesis kit (Promega Corp., Madison, WI) as recommended by the manufacturer. Simultaneously, GAPDH forward and reverse primers (Table 1) were used to amplify the house keeping gene as internal control for standardization of PCR products. The RTq-PCR was done using SYBR Green dye (QuantiTect SYBR Green PCR Kits) and Light Cycler fluorimeter (Bio-RAD S1000 Tm thermal cycler). The PCR cycling program was as follows: 95°C for 2 min, followed by 40 cycles of 95°C for 30s, 55°C for 30 s, and 60°C for 45s, and finally 60ºC for 5 min.

-Sequence of the primers

Primer ID	Sequence
Bcl-F	5′-TATAAGCTGTCGCAGAGGGGCTA-3′
Bcl-R	5′-GTACTCAGTCATCCACAGGGCGAT-3′
Bcl-Xlf	5’CAGAGCTTTGAACAGGTAG-3′
Bcl-XlR	5’GCTCTCGGGTGCTGTATTG-3′
Surv-F	5′-TGCCCCGACGTTGCC-3′
Surv-R	5′-CAGTTCTTGAATGTAGAGATGCGGT-3′
TGF-F	5′CAAGGGCTACCATGCCAACT3′
TGF-R	5′AGGGCCAGGACCTTGCTG3′
β-actin-F	5′-GTGGGGCGCCCCAGGCACCA-3′
β-actin-R	5′-CTCCTTAATGTCACGCACGATTTC-3′

2- ***In silico* studies**

a- Docking studies

The docking studies were performed utilizing MOE.19 software to explore the binding mode of the synthesized compounds towards VEGFR-2. The 3D crystal structures of the target macromolecules VEGFR-2 were downloaded from the protein databank, http://www.pdb.org (PDB ID; 2OH4). Sorafenib was used as reference ligand. To prepare the target protein, water molecules were removed, and the valances of atoms were corrected through protonation of the whole molecule. Then energy minimization was carried out by applying CHARMM and MMFF94 force fields. After that, the active binding site was defined and prepared for docking. The validation process was performed by redocking the co-crystallized ligand. The designed compounds together with sorafenib were drawn using ChemBioDraw Ultra 14.0 and saved as MDL-SD format. The sketched compounds were constructed from fragment libraries in MOE program, protonated, followed by energy minimization then prepared for docking. Docking process was carried through Triangle matcher placement inserted in compute window, and the scoring function was London dG. Ten conformers (poses) for each molecule were generated using genetic algorithm searches. The free energies and binding modes of the designed molecules against VEGFR-2 were determined. The most ideal pose was selected according to its binding free energy as well as its binding mode with target molecule.

b- ADMET studies

ADMET descriptors (absorption, distribution, metabolism, excretion and toxicity) of the synthesized compounds were determined using Discovery studio 4.0. At first, the CHARMM force field was applied then the compounds were prepared and minimized according to the preparation of small molecule protocol. Then ADMET descriptors protocol was applied to carry out these studies.

c- Toxicity studies

The toxicity parameters of the synthesized compounds were calculated using Discovery studio 4.0. Sorafenib was used as a reference drug. At first, the CHARMM force field was applied then the compounds were prepared and minimized according to the preparation of small molecule protocol. Then different parameters were calculated from toxicity prediction (extensible) protocol.

d- **Molecular dynamics simulation**

Molecular dynamics simulation of the protein-ligand complexes was performed using GROMACS 2021 and Linux 5.4 package. The GROMOS96 54a7 forcefield was selected as the force field for proteins and the ligand topologies were generated from the PRODRG server. All the complexes were solvated using simple point charge (SPC) water molecules in a rectangular box. To make the simulation system electrically neutral, required number of Na^+ and Cl^- ions were added while 0.15 mol/L salt concentrations were set in all the systems. Using the steepest descent method, all the solvated systems were subjected to energy minimization for 5000 steps. Afterwards, NVT (constant number of particles, volume, and temperature) series, NPT (constant number of particles, pressure, and temperature) series, and the production run were conducted in the MD simulation. The NVT and the NPT series were conducted at a 300 K temperature and 1 atm pressure for the duration of 300 ps. V-rescale thermostat and Parrinello-Rahman barostat were selected of the performed simulation. Finally, the production run was performed at 300 K for a duration of 100 ns (nanoseconds). Thereafter, a comparative analysis was performed measuring root mean square deviation (RMSD), root mean square fluctuation (RMSF), radius of gyration (Rg), solvent accessible surface area (SASA) and hydrogen bonds to analyze their stability. The Xmgrace program was used to represent the analyses in the form of plots.

e- **MM/PBSA**

The g_mmpbsa package of GROMACS was utilized to calculate the MM/PBSA (Molecular Mechanics/Poisson Boltzmann Surface Area) binding free energies followed by final MD production run to get a detailed overview of the molecular interactions between the protein and ligand. The free solvation energy (polar and nonpolar solvation energies) and potential energy (electrostatic and Van der Waals interactions) of each protein-ligand complex were analyzed to determine the total ΔG_{bind} of the complex. The binding energies were calculated using the following equation in this method:

$$\Delta G_{binding} = G_{complex} - (G_{protein} + G_{ligand})$$

Here, the $\Delta G_{binding}$ = the total binding energy of the protein-ligand complex, $G_{protein}$ = the binding energy of free protein, and G_{ligand} = the binding energy of unbounded ligand.

Chemistry and material

All melting points were carried out by open capillary method on a Gallen kamp Melting point apparatus. The infrared spectra were recorded on pye Unicam SP 1000 IR spectrophotometer using potassium bromide disc technique. Proton magnetic resonance 1HNMR spectra were recorded on a Bruker 400 Megahertz-nuclear magnetic resonance (400 MHZ-NMR) spectrophotometer. Carbon-13 (C13) nuclear magnetic resonance (13CNMR) spectra were recorded on a Bruker 100 Megahertz-nuclear magnetic resonance (100 MHZ-NMR) spectrophotometer. Tetramethylsilane (TMS) was used as internal standard and chemical shifts were measured in δ scale one part per million (ppm). All compounds were within ±0.4 of the theoretical values. The reactions were monitored by thin-layer chromatography (TLC) using TLC sheets precoated with UV fluorescent silica gel Merck 60 F254 plates and were visualized using ultraviolet (UV) lamp and different solvents as mobile phases.

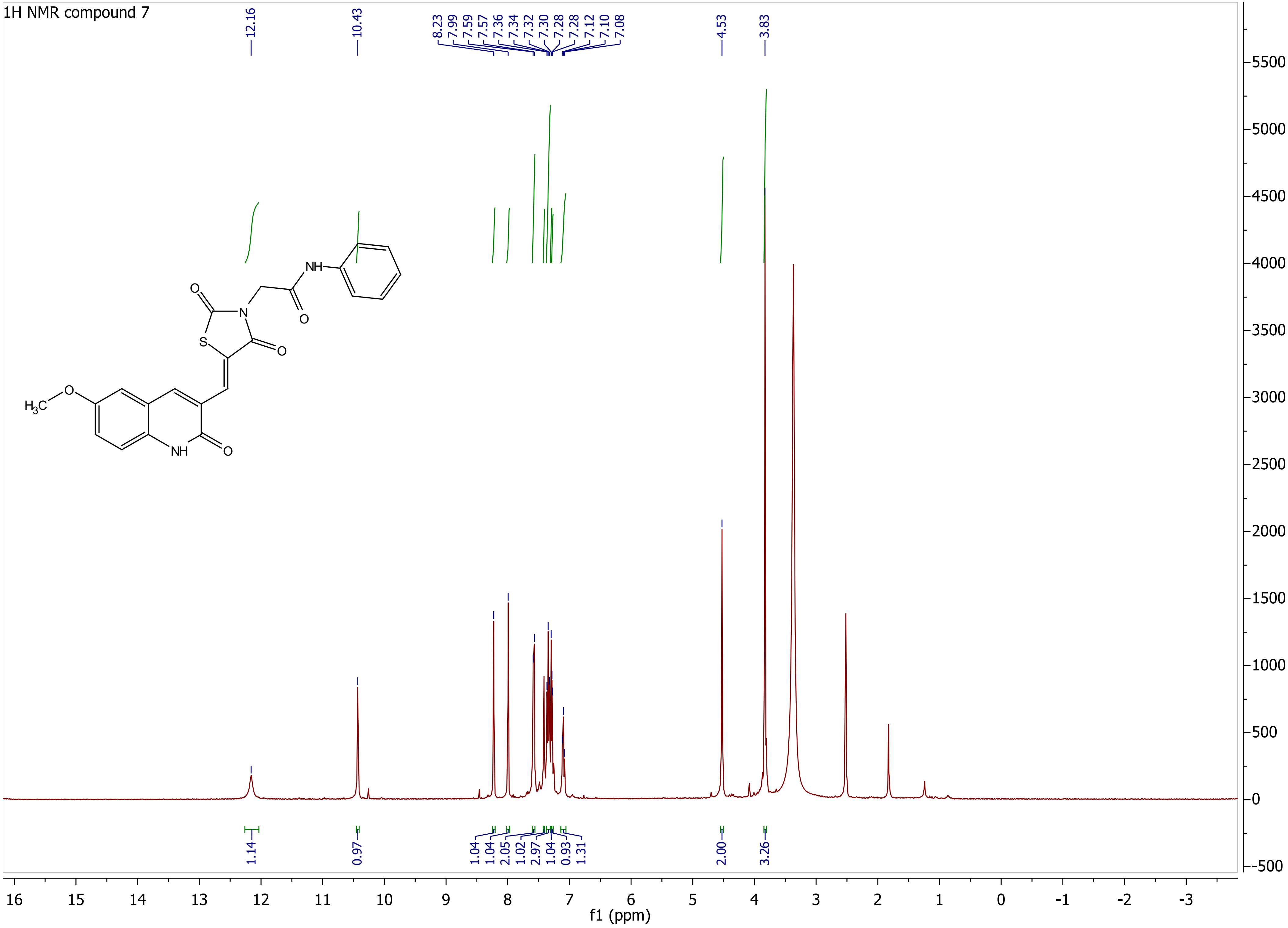
1H NMR compound 7
12.16
10.43
8.23
7.99
7.59
7.57
7.36
7.34
7.32
7.30
7.28
7.28
7.12
7.10
7.08
4.53
3.83
H3C
O
NH
O
S
N
O
O
NH
O
1.14
0.97
1.04
1.04
2.05
1.02
2.97
1.04
0.93
1.31
2.00
3.26
f1 (ppm)

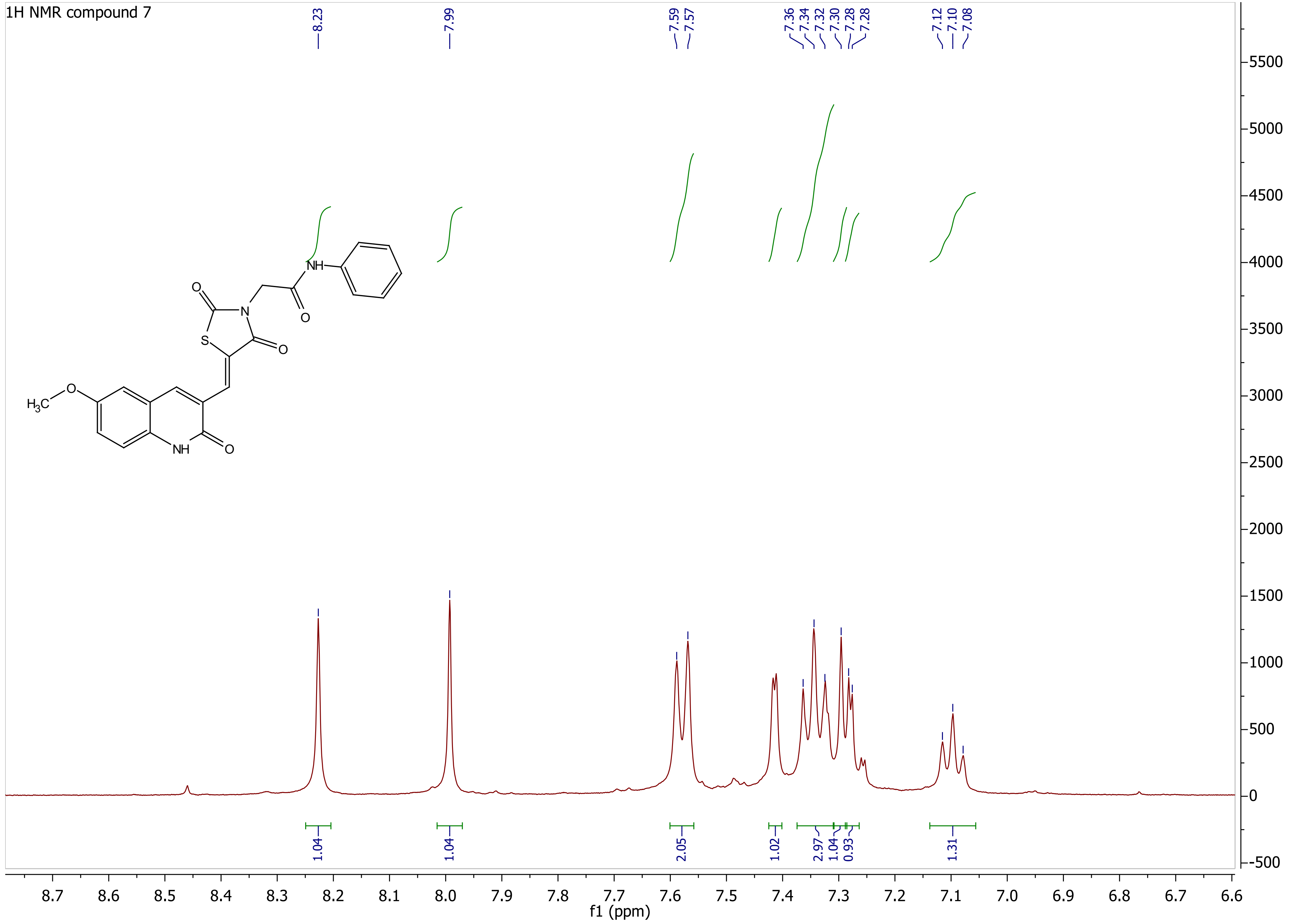
1H NMR compound 7
8.23
7.99
7.59
7.57
7.36
7.34
7.32
7.30
7.28
7.28
7.12
7.10
7.08
1.04
1.04
2.05
1.02
2.97
1.04
0.93
1.31
f1 (ppm)
8.7 8.6 8.5 8.4 8.3 8.2 8.1 8.0 7.9 7.8 7.7 7.6 7.5 7.4 7.3 7.2 7.1 7.0 6.9 6.8 6.7 6.6
5500 5000 4500 4000 3500 3000 2500 2000 1500 1000 500 0 -500
NH
O
N
S
O
O
H_3C
O
NH
O

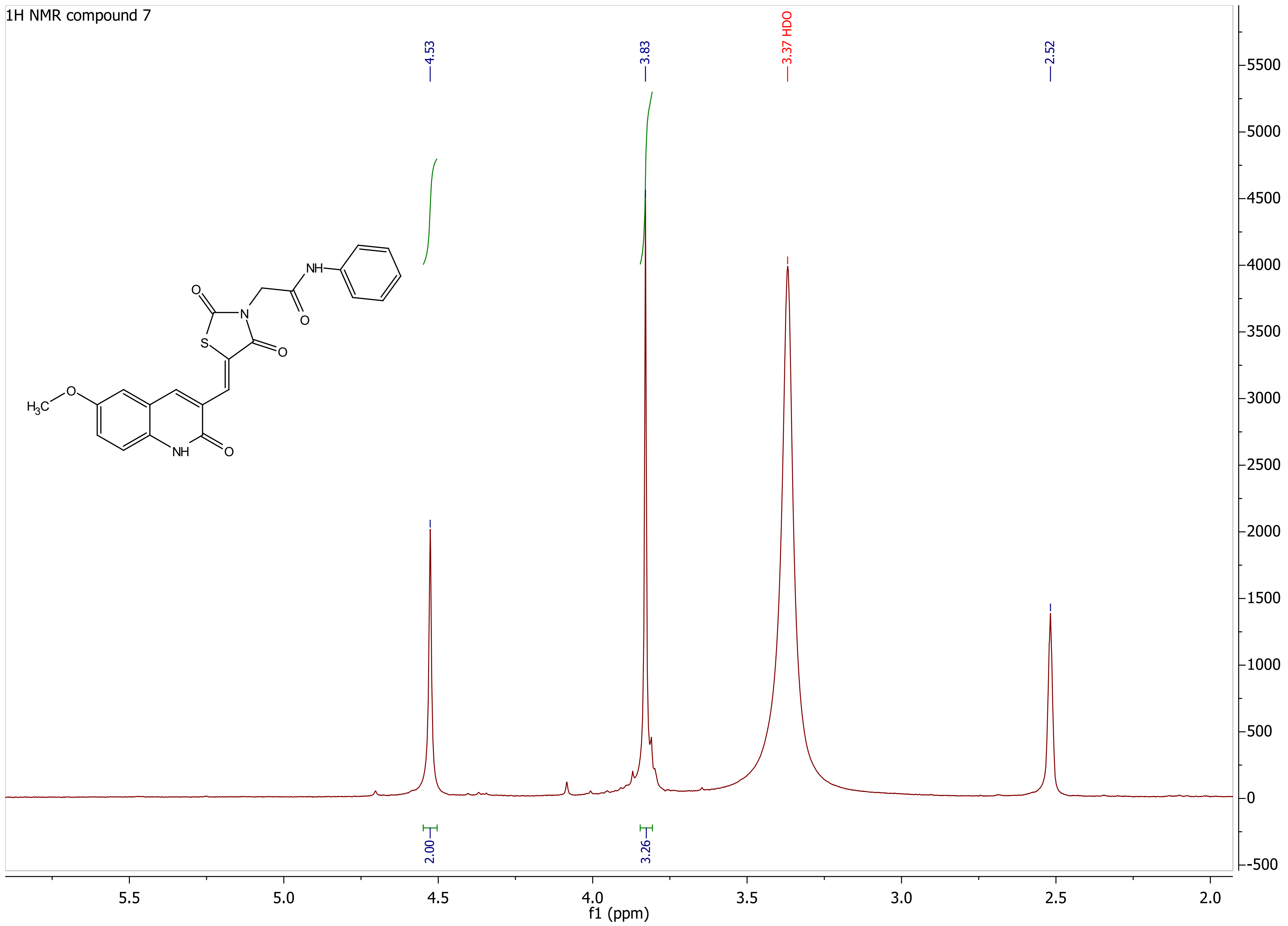

1H NMR compound 7
4.53
3.83
3.37 HDO
2.52
H3C
O
NH
O
S
N
O
O
NH
O
2.00
3.26
5.5
5.0
4.5
4.0
3.5
3.0
2.5
2.0
f1 (ppm)
5500
5000
4500
4000
3500
3000
2500
2000
1500
1000
500
0
-500

13C NMR compound 7

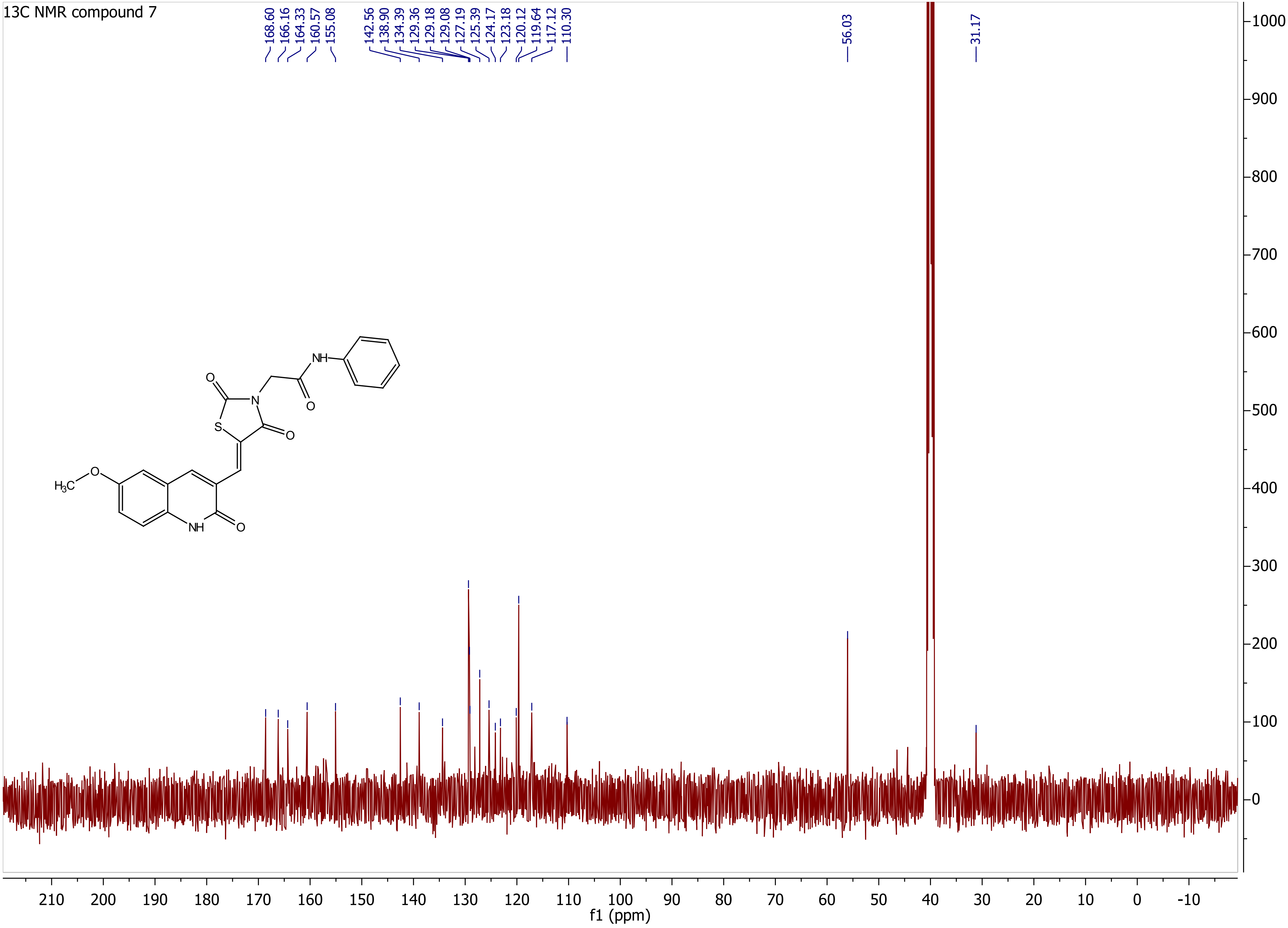

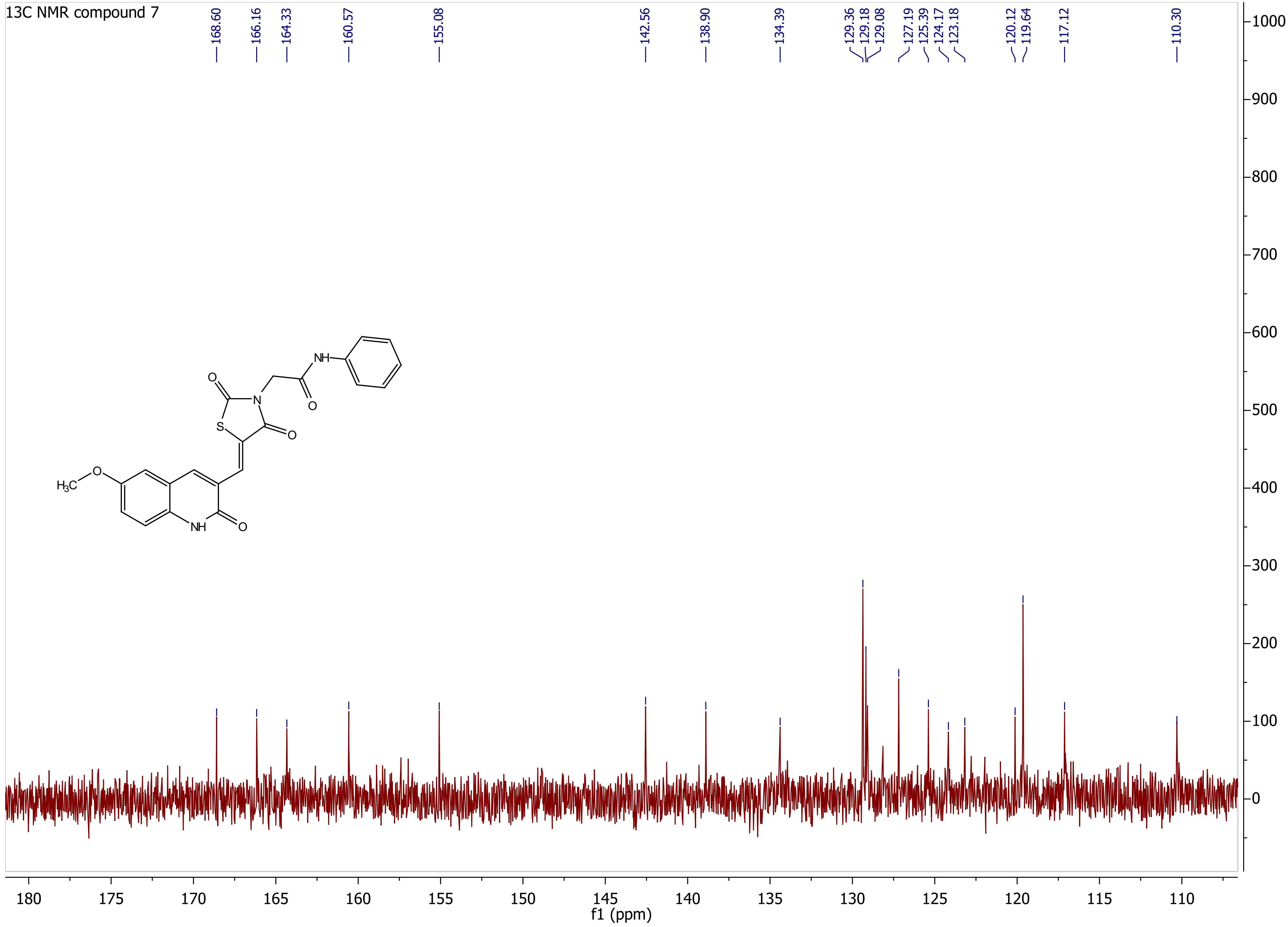
13C NMR compound 7
168.60
166.16
164.33
160.57
155.08
142.56
138.90
134.39
129.36
129.18
129.08
127.19
125.39
124.17
123.18
120.12
119.64
117.12
110.30
NH
O
N
S
O
O
H3C
O
NH
O
f1 (ppm)

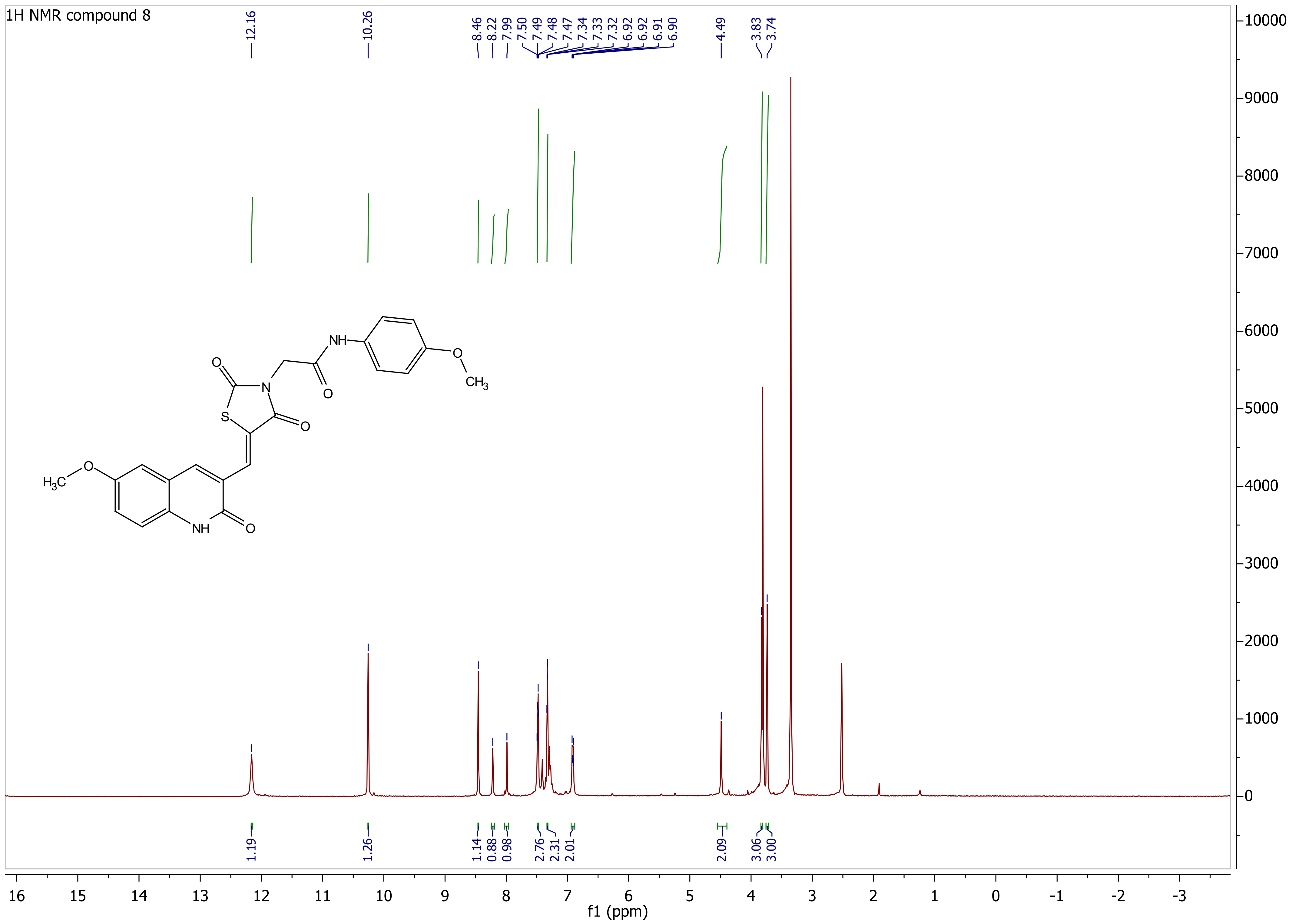
1H NMR compound 8
12.16
10.26
8.46
8.22
7.99
7.50
7.49
7.48
7.47
7.34
7.33
7.32
6.92
6.92
6.91
6.90
4.49
3.83
3.74
1.19
1.26
1.14
0.88
0.98
2.76
2.31
2.01
2.09
3.06
3.00
f1 (ppm)

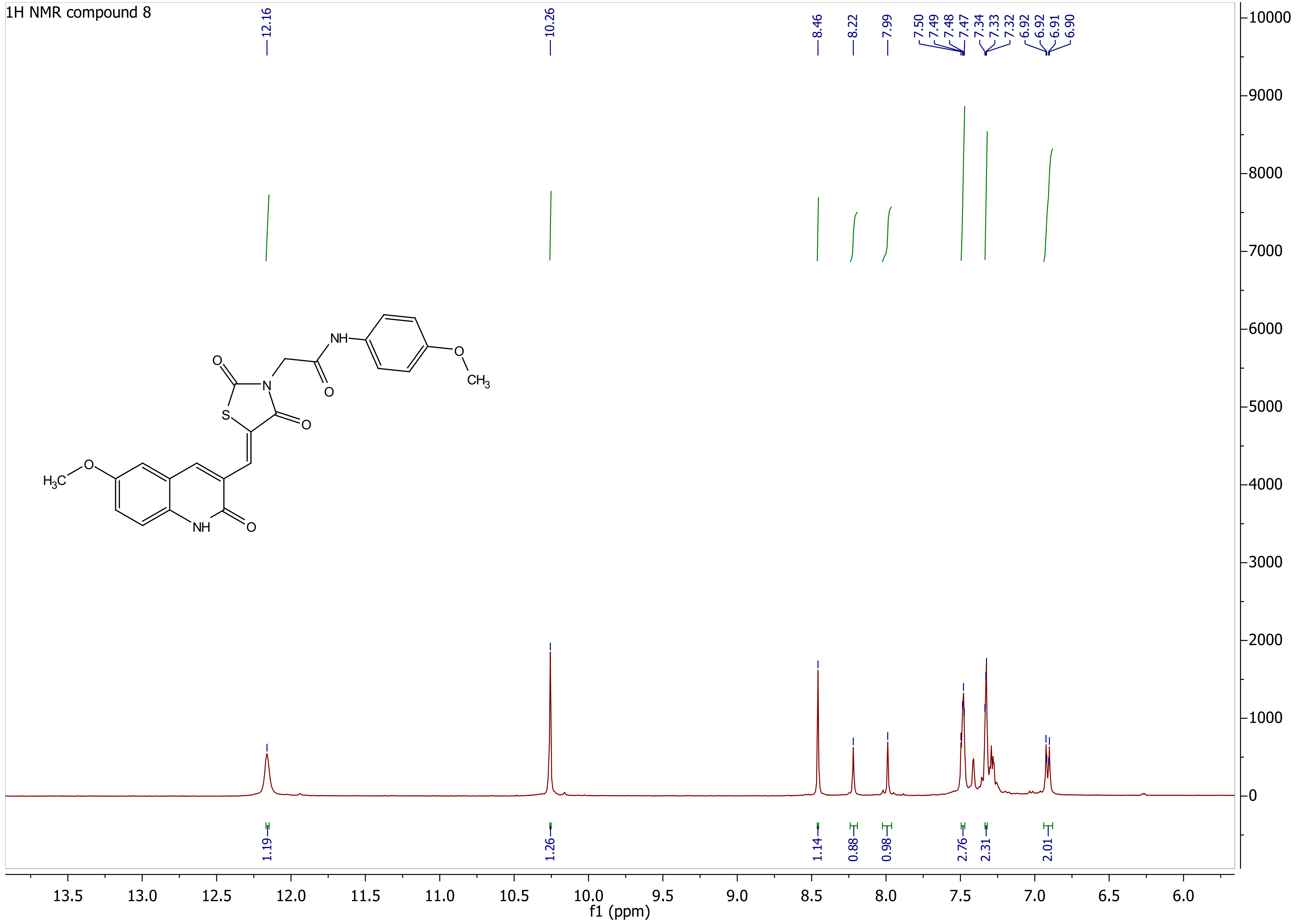
1H NMR compound 8
12.16
10.26
8.46
8.22
7.99
7.50
7.49
7.48
7.47
7.34
7.33
7.32
6.92
6.92
6.91
6.90
1.19
1.26
1.14
0.88
0.98
2.76
2.31
2.01
f1 (ppm)
13.5
13.0
12.5
12.0
11.5
11.0
10.5
10.0
9.5
9.0
8.5
8.0
7.5
7.0
6.5
6.0
10000
9000
8000
7000
6000
5000
4000
3000
2000
1000
0
NH
O
CH3
N
S
H3C
NH

1H NMR compound 8

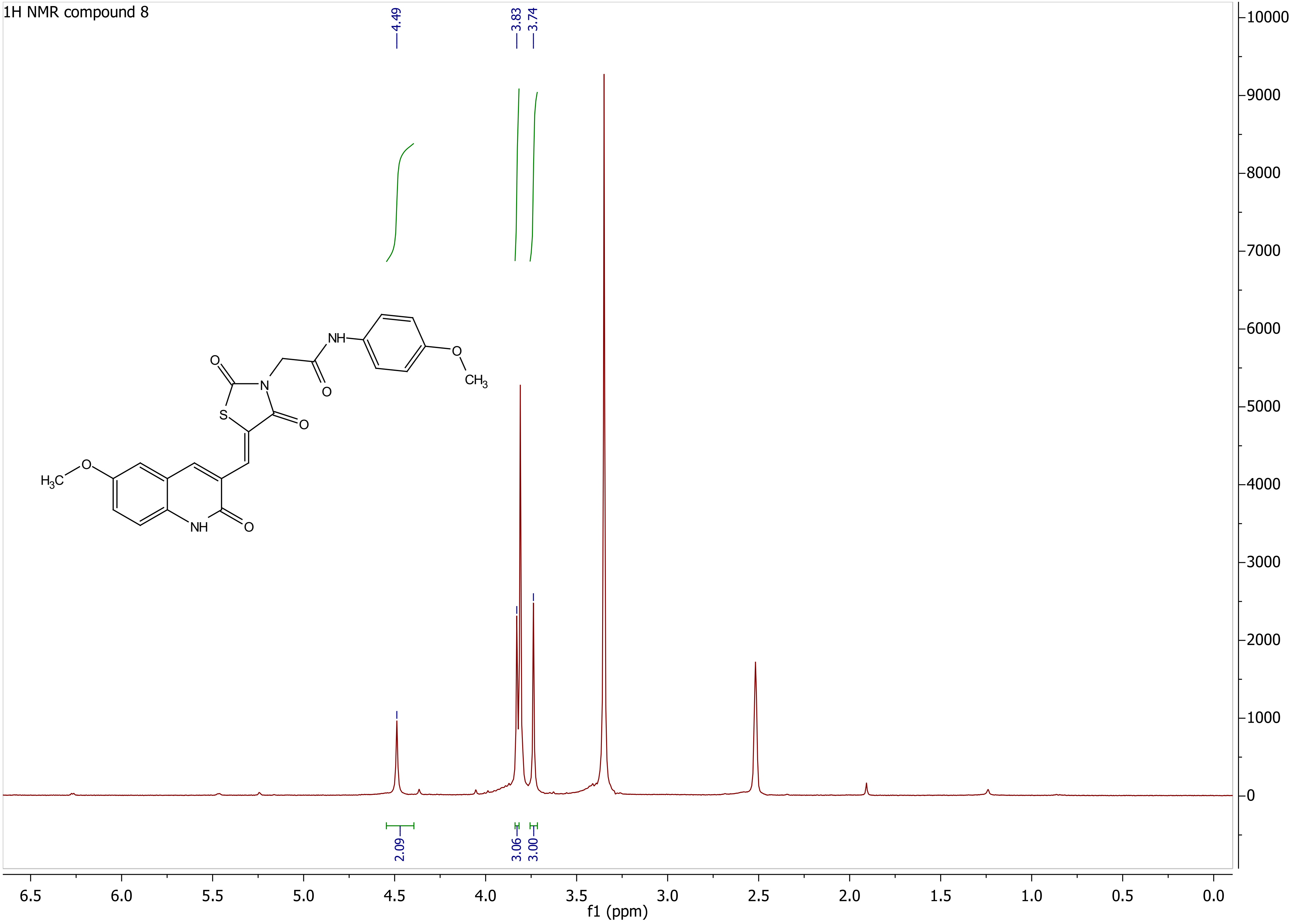

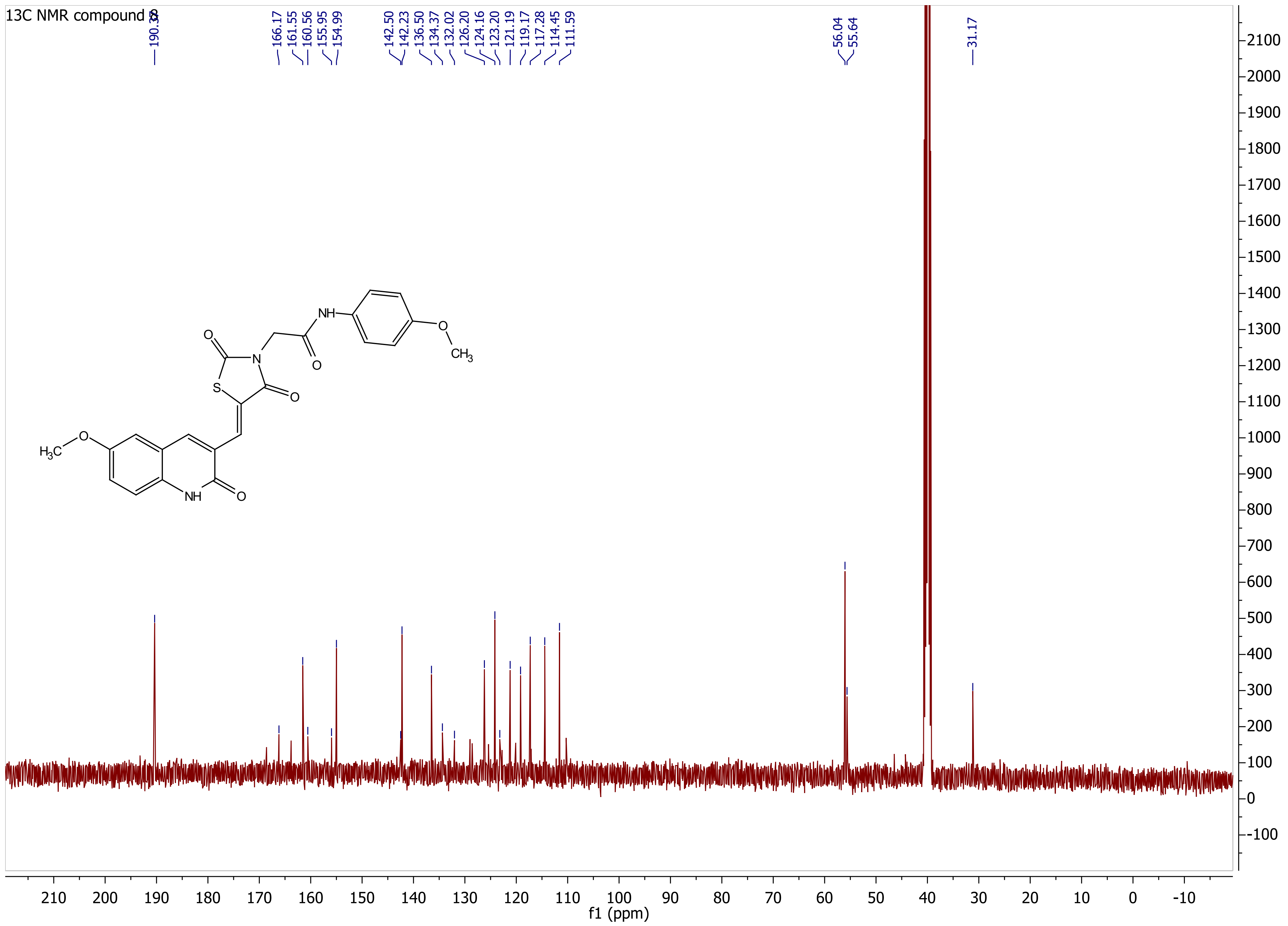

13C NMR compound 8
166.17
161.55
160.56
155.95
154.99
142.50
142.23
136.50
134.37
132.02
126.20
124.16
123.20
121.19
119.17
117.28
114.45
111.59
56.04
55.64
31.17
NH
O
N
S
O
O
CH3
H3C
O
NH
O
f1 (ppm)

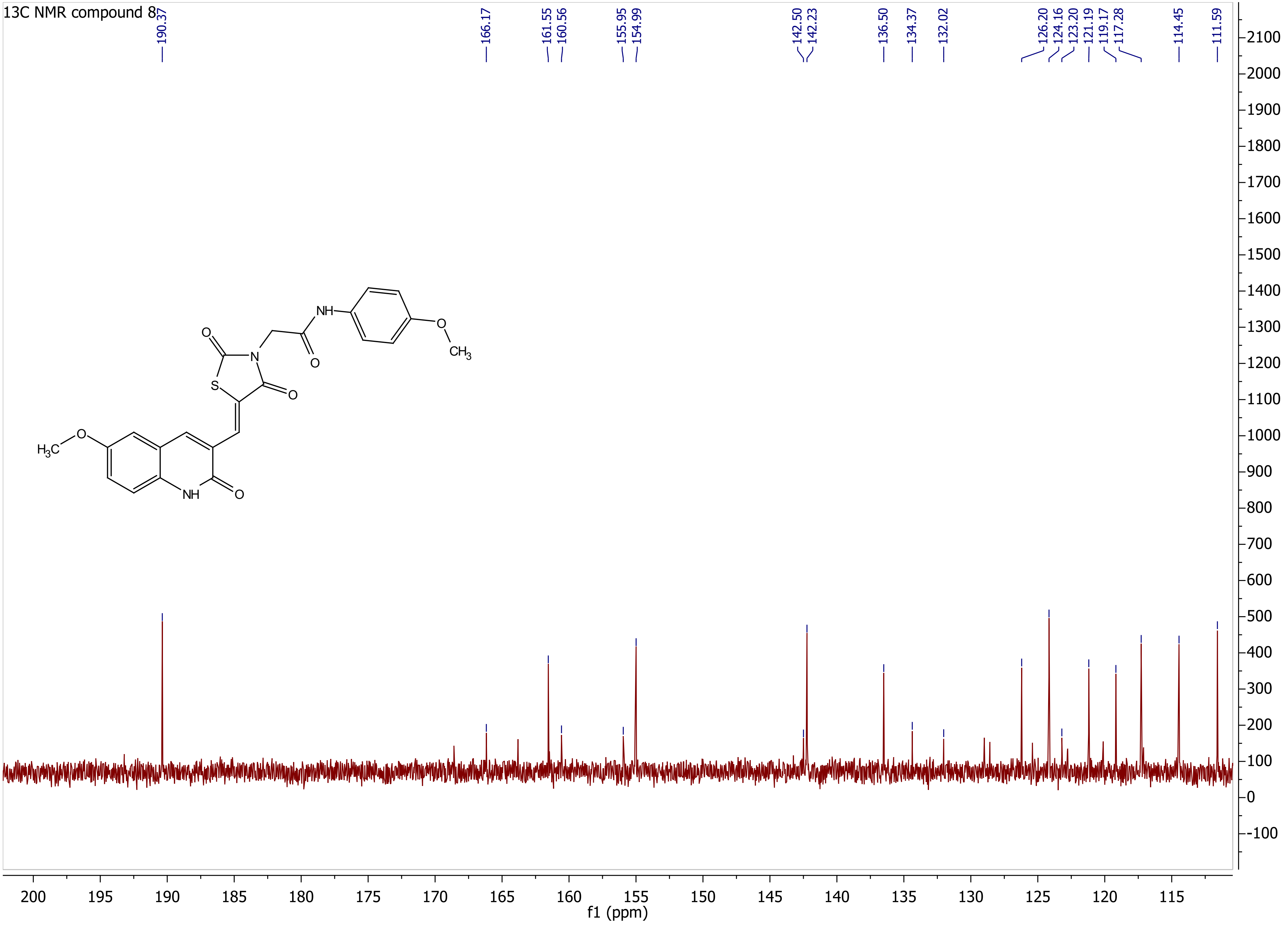
13C NMR compound 8
190.37
166.17
161.55
160.56
155.95
154.99
142.50
142.23
136.50
134.37
132.02
126.20
124.16
123.20
121.19
119.17
117.28
114.45
111.59
f1 (ppm)
200 195 190 185 180 175 170 165 160 155 150 145 140 135 130 125 120 115
2100 2000 1900 1800 1700 1600 1500 1400 1300 1200 1100 1000 900 800 700 600 500 400 300 200 100 0 -100
NH
O
CH3
N
S
H3C
NH

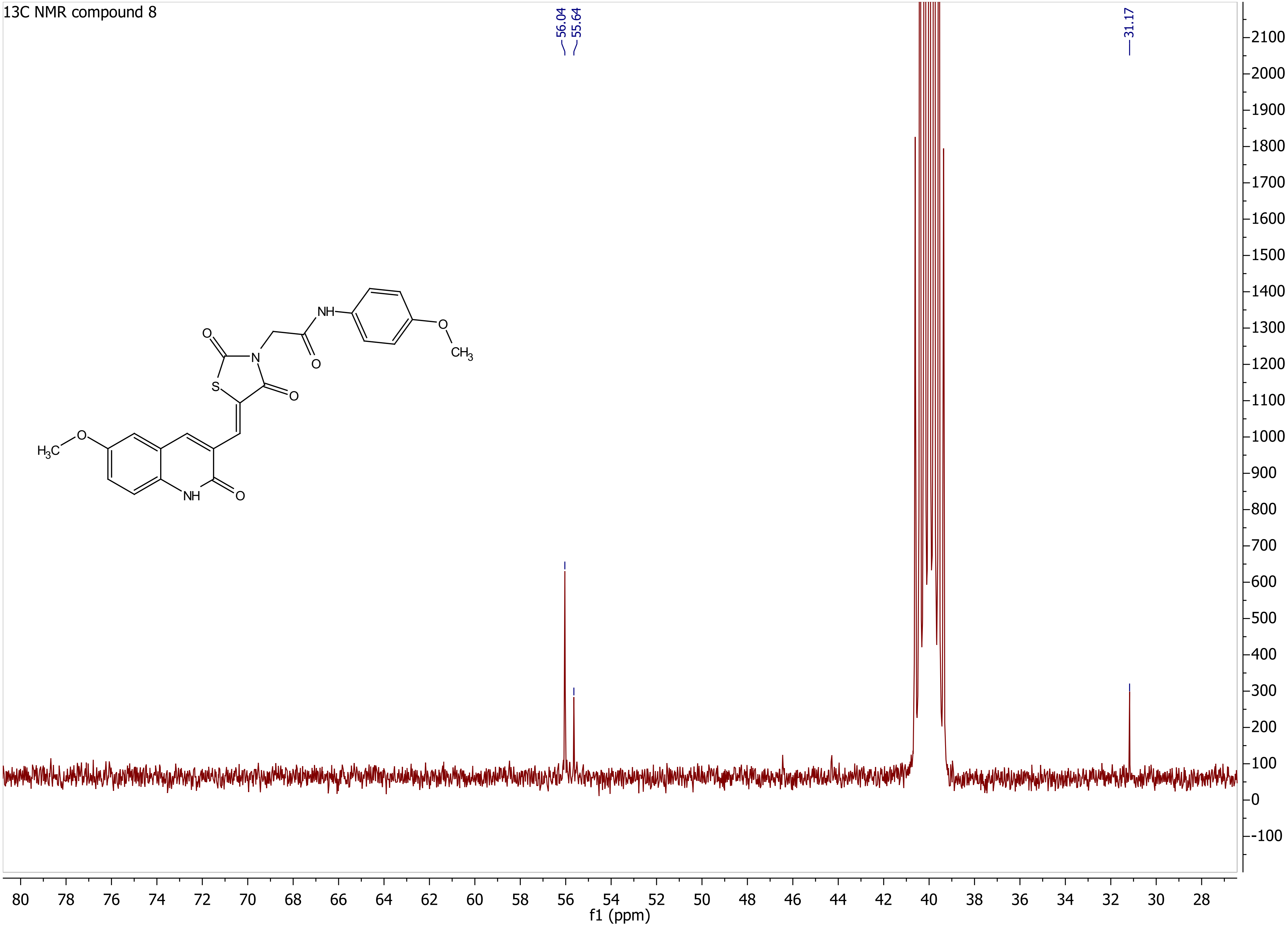

13C NMR compound 8
56.04
55.64
31.17
NH
O
CH3
O
N
O
S
O
H3C
O
NH
O
f1 (ppm)
80
78
76
74
72
70
68
66
64
62
60
58
56
54
52
50
48
46
44
42
40
38
36
34
32
30
28
2100
2000
1900
1800
1700
1600
1500
1400
1300
1200
1100
1000
900
800
700
600
500
400
300
200
100
0
-100

FQ3

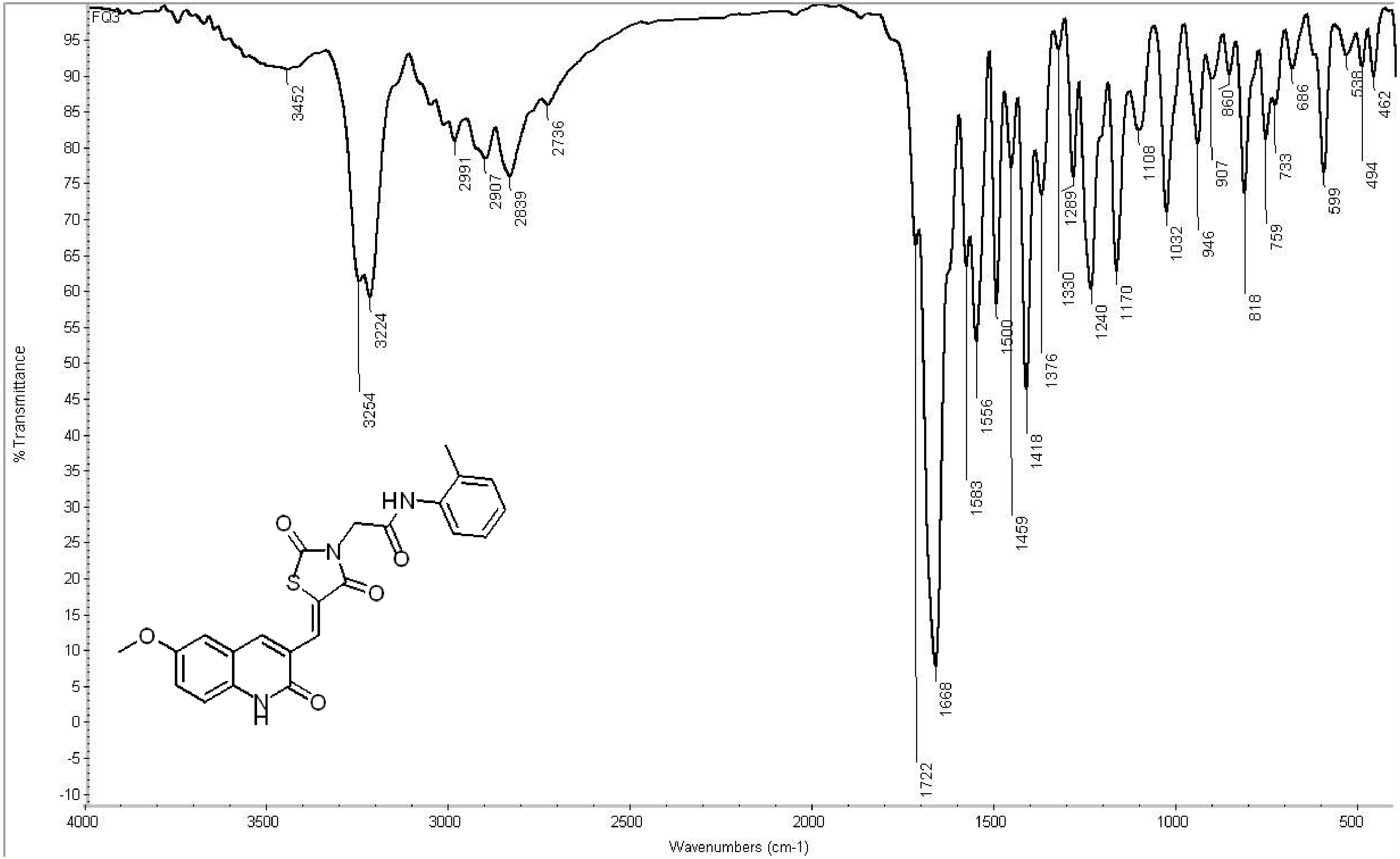
FQ3
%Transmittance
Wavenumbers (cm-1)
3452
3254
3224
2991
2907
2839
2736
1722
1668
1583
1556
1500
1459
1418
1376
1330
1289
1240
1170
1108
1032
946
907
860
818
759
733
686
599
538
494
462
HN
O
N
S
O
O
O
N
H
O

IR of Comp. 13

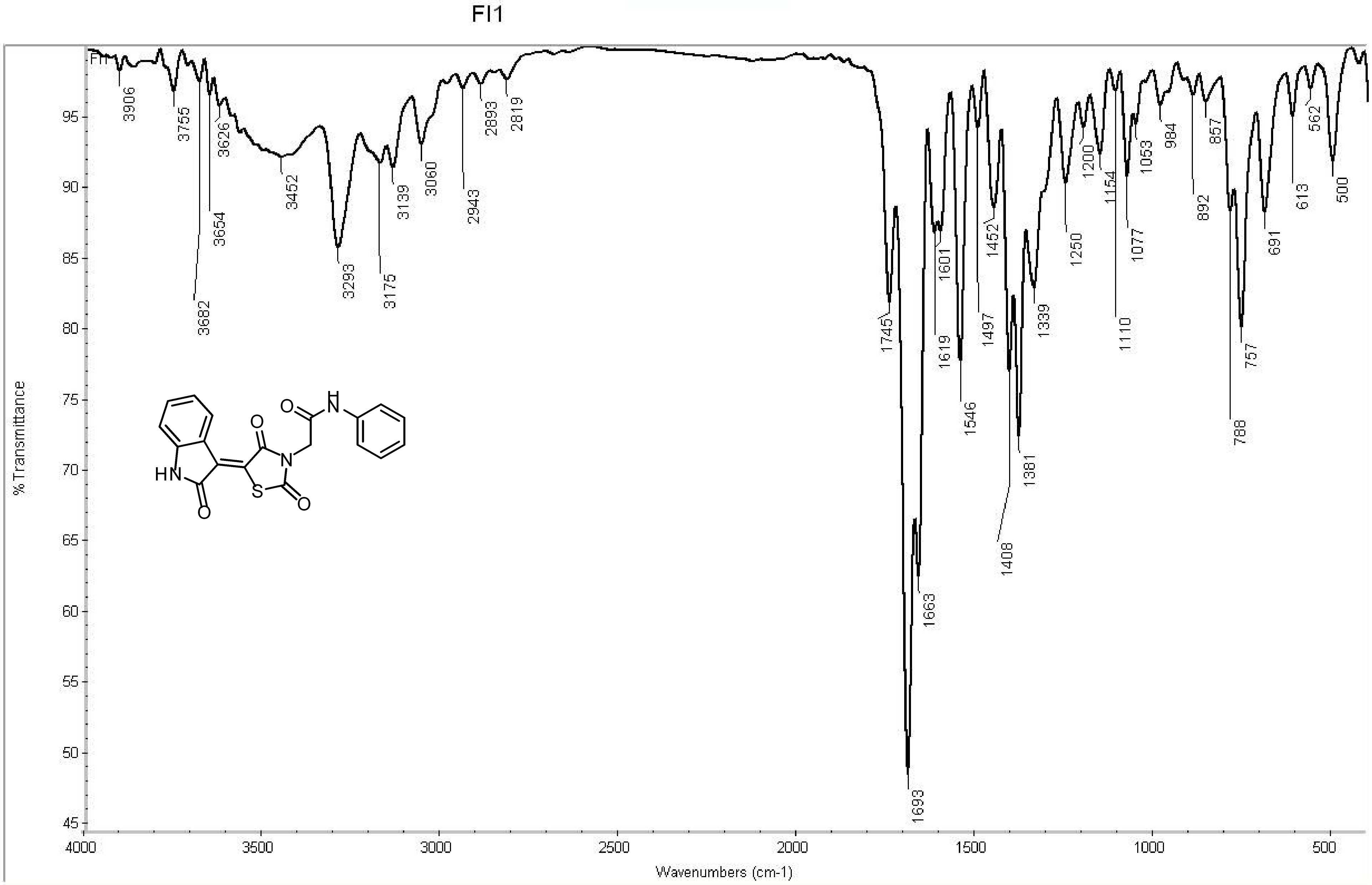
FI1
% Transmittance
Wavenumbers (cm-1)
3906
3755
3682
3654
3626
3452
3293
3175
3139
3060
2943
2893
2819
1745
1693
1663
1619
1601
1546
1497
1452
1408
1381
1339
1250
1200
1154
1110
1077
1053
984
892
857
788
757
691
613
562
500
HN
O
S
N
H

^{1}H NMR of Comp. 13

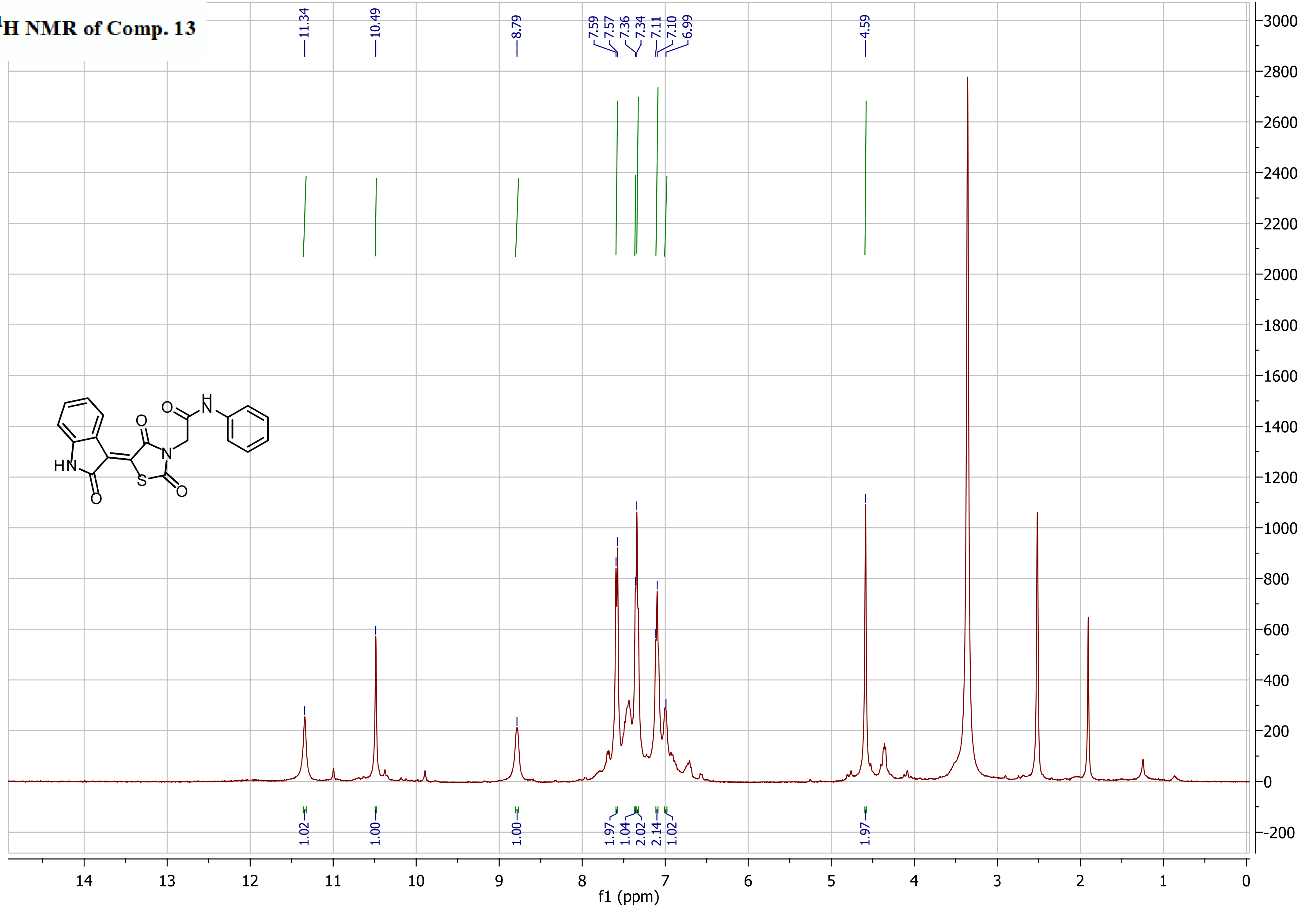

^{1}H NMR of Comp. 13

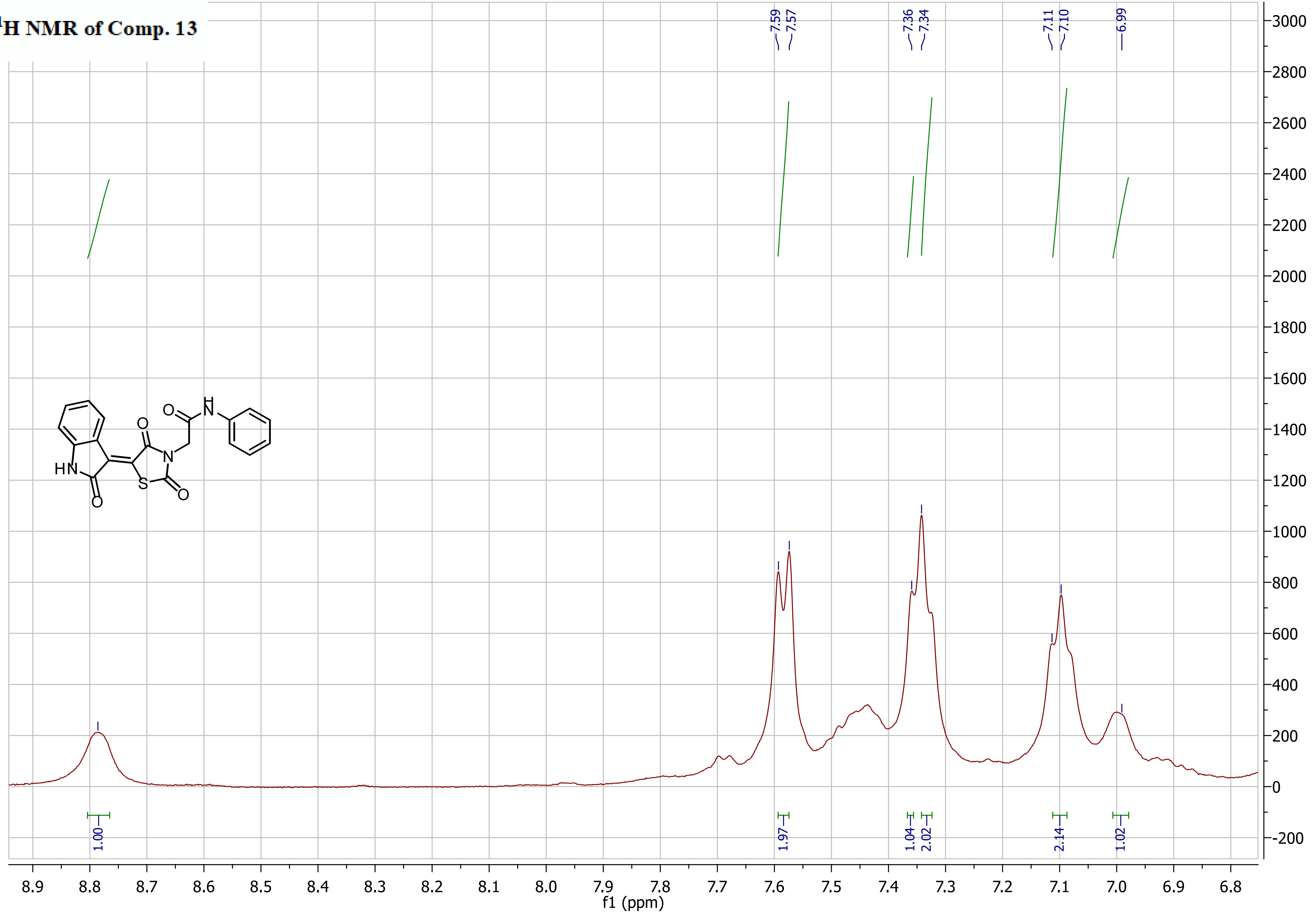

^{13}C NMR of Comp. 13

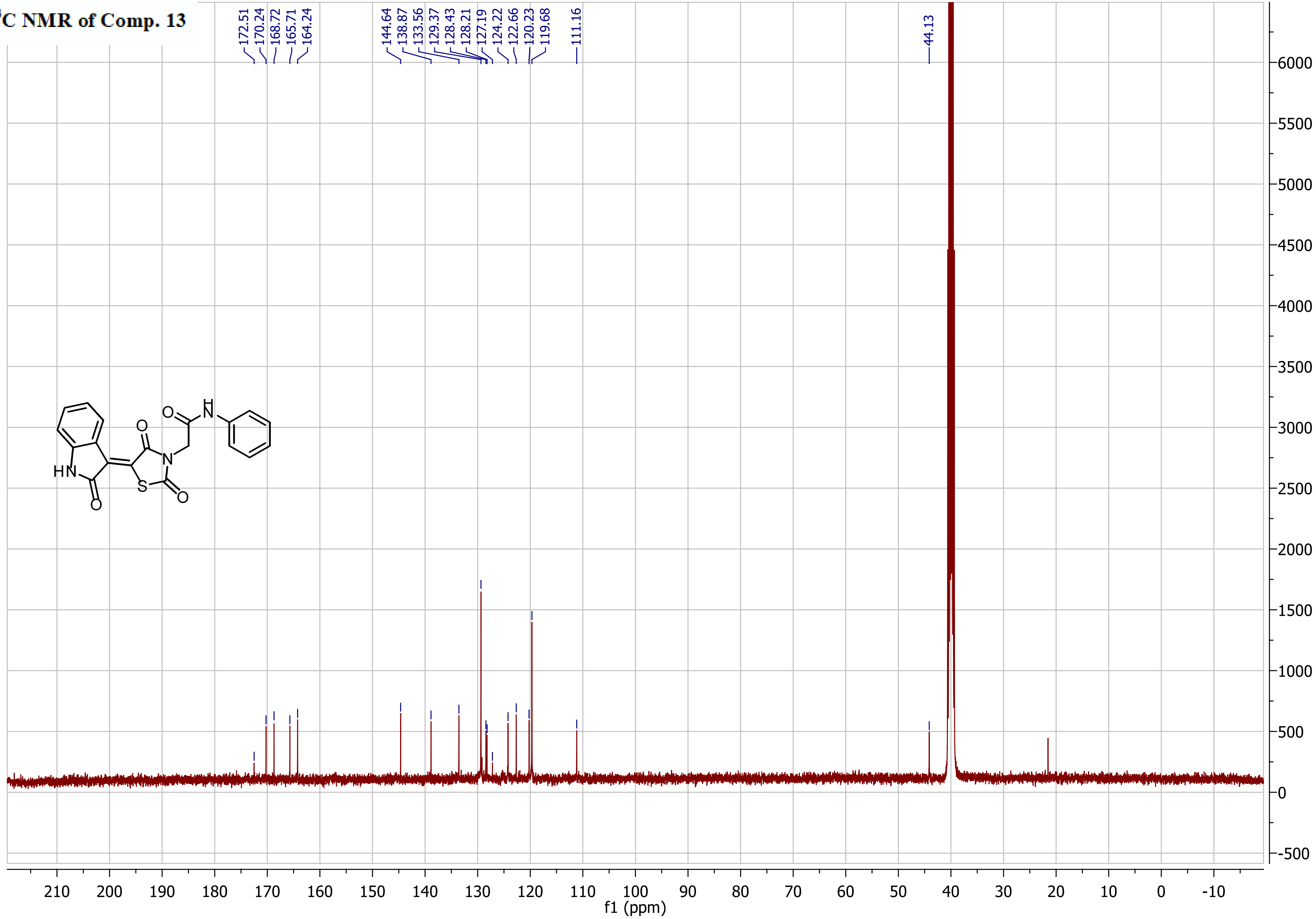

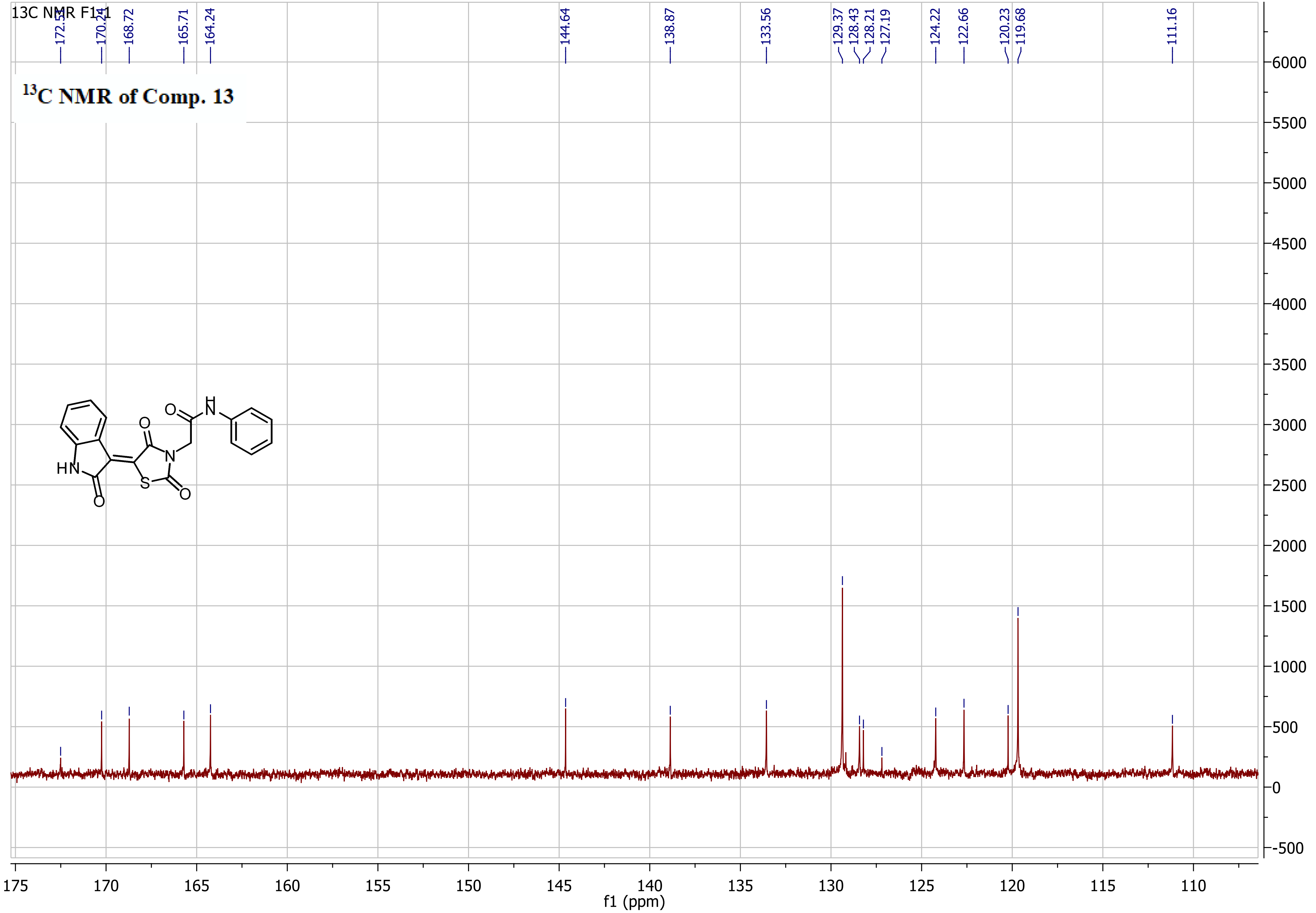
13C NMR of Comp. 13
168.72
165.71
164.24
144.64
138.87
133.56
129.37
128.43
128.21
127.19
124.22
122.66
120.23
119.68
111.16
f1 (ppm)

IR of Comp. 14

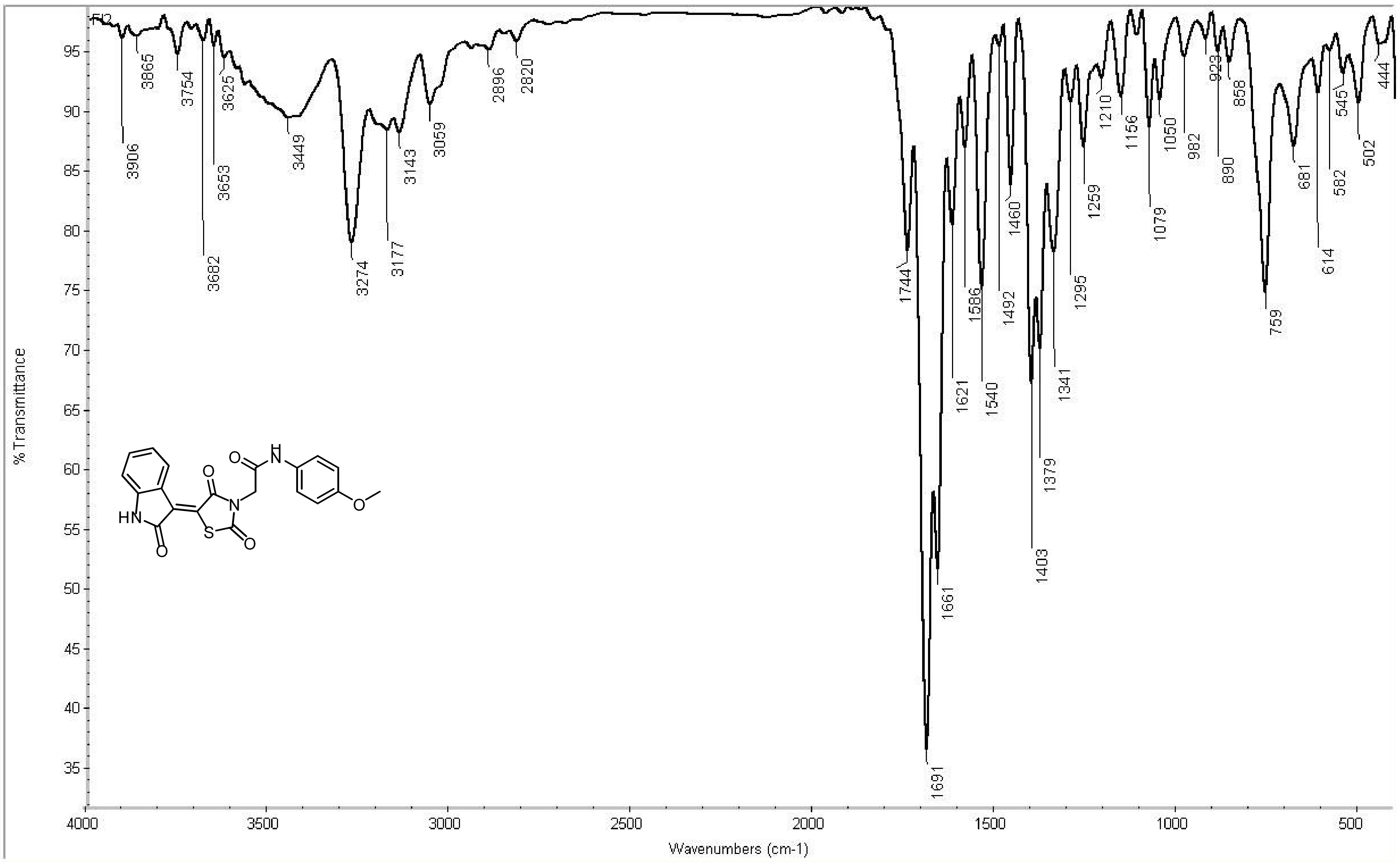

%Transmittance
Wavenumbers (cm-1)
95
90
85
80
75
70
65
60
55
50
45
40
35
4000
3500
3000
2500
2000
1500
1000
500
3906
3865
3754
3682
3653
3625
3449
3274
3177
3143
3059
2896
2820
1744
1691
1661
1621
1586
1540
1492
1460
1403
1379
1341
1295
1259
1210
1156
1079
1050
982
923
890
858
759
681
614
582
545
502
444
HN
O
S
N
H

^{1}H NMR of Comp. 14

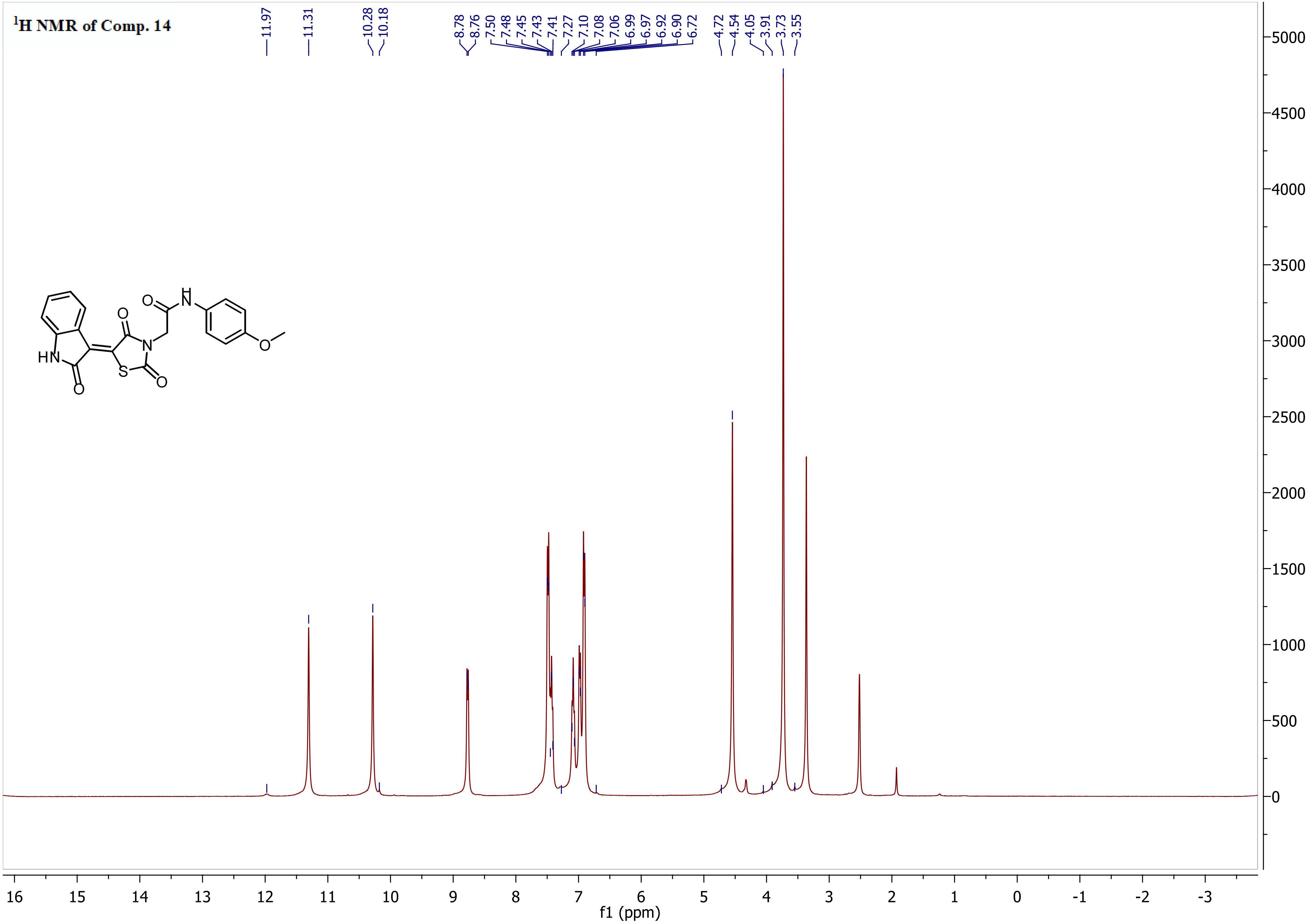

^{13}C NMR of Comp. 14

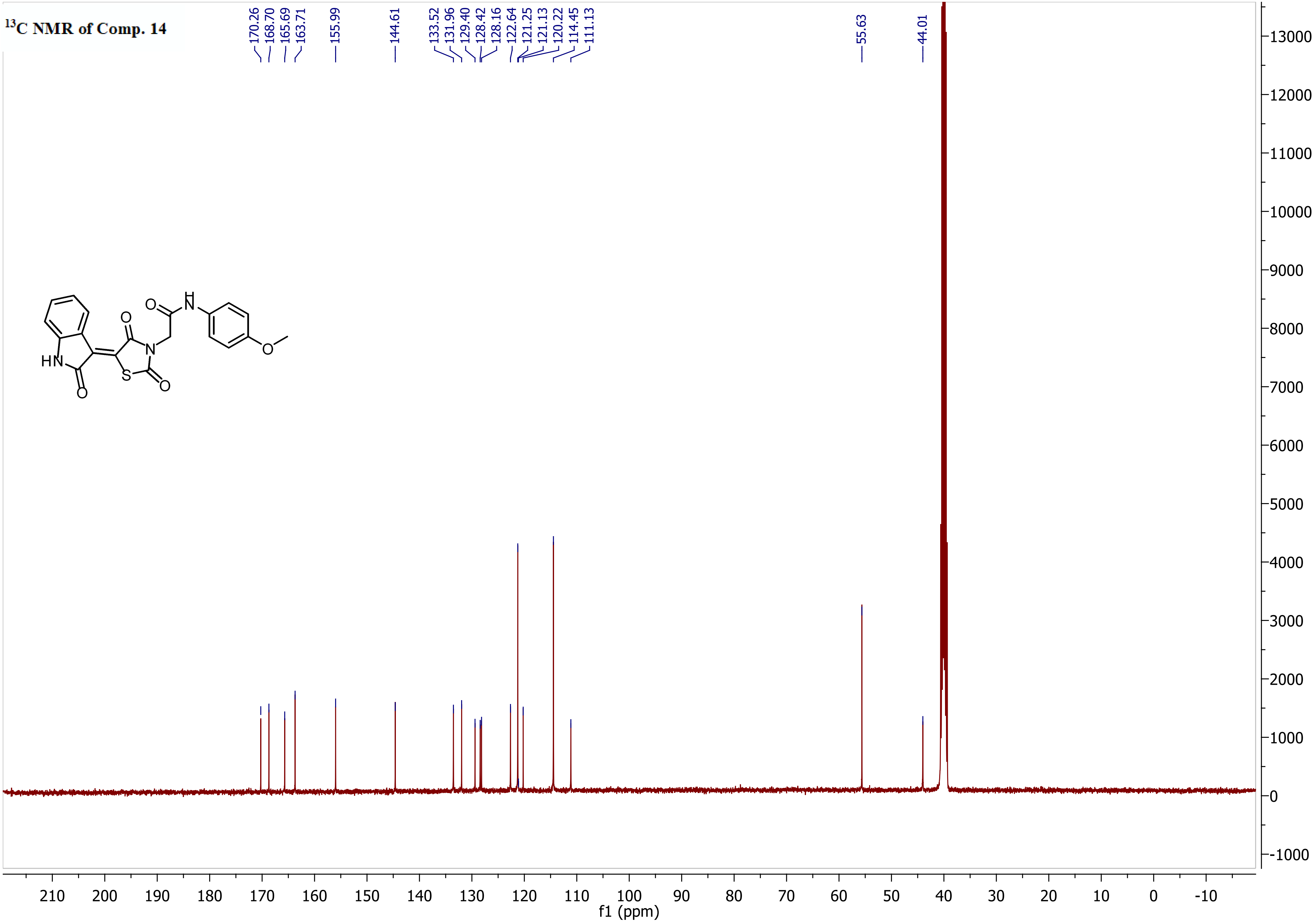

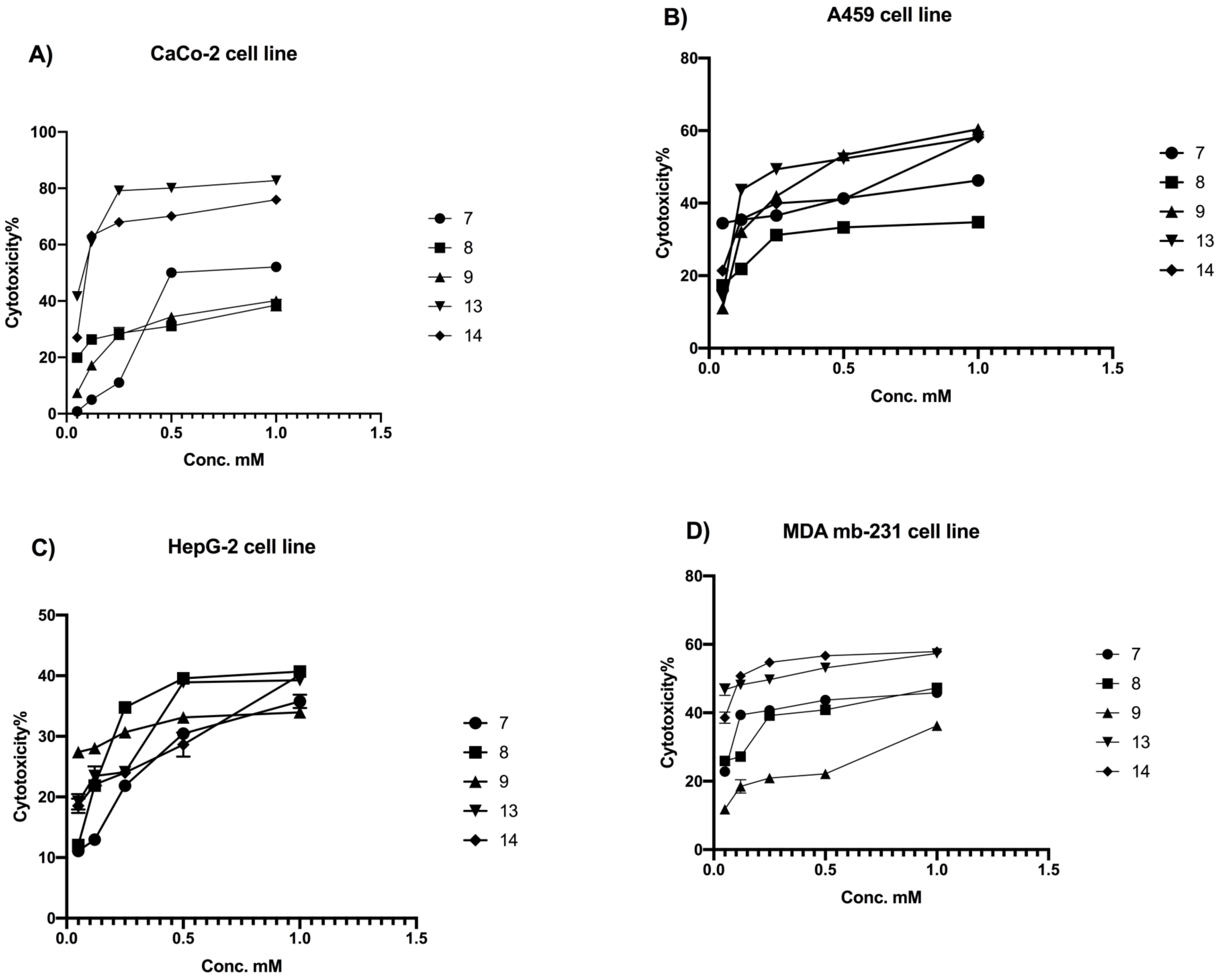
A)
CaCo-2 cell line
Cytotoxicity%
Conc. mM
B)
A459 cell line
Cytotoxicity%
Conc. mM
C)
HepG-2 cell line
Cytotoxicity%
Conc. mM
D)
MDA mb-231 cell line
Cytotoxicity%
Conc. mM
7
8
9
13
14

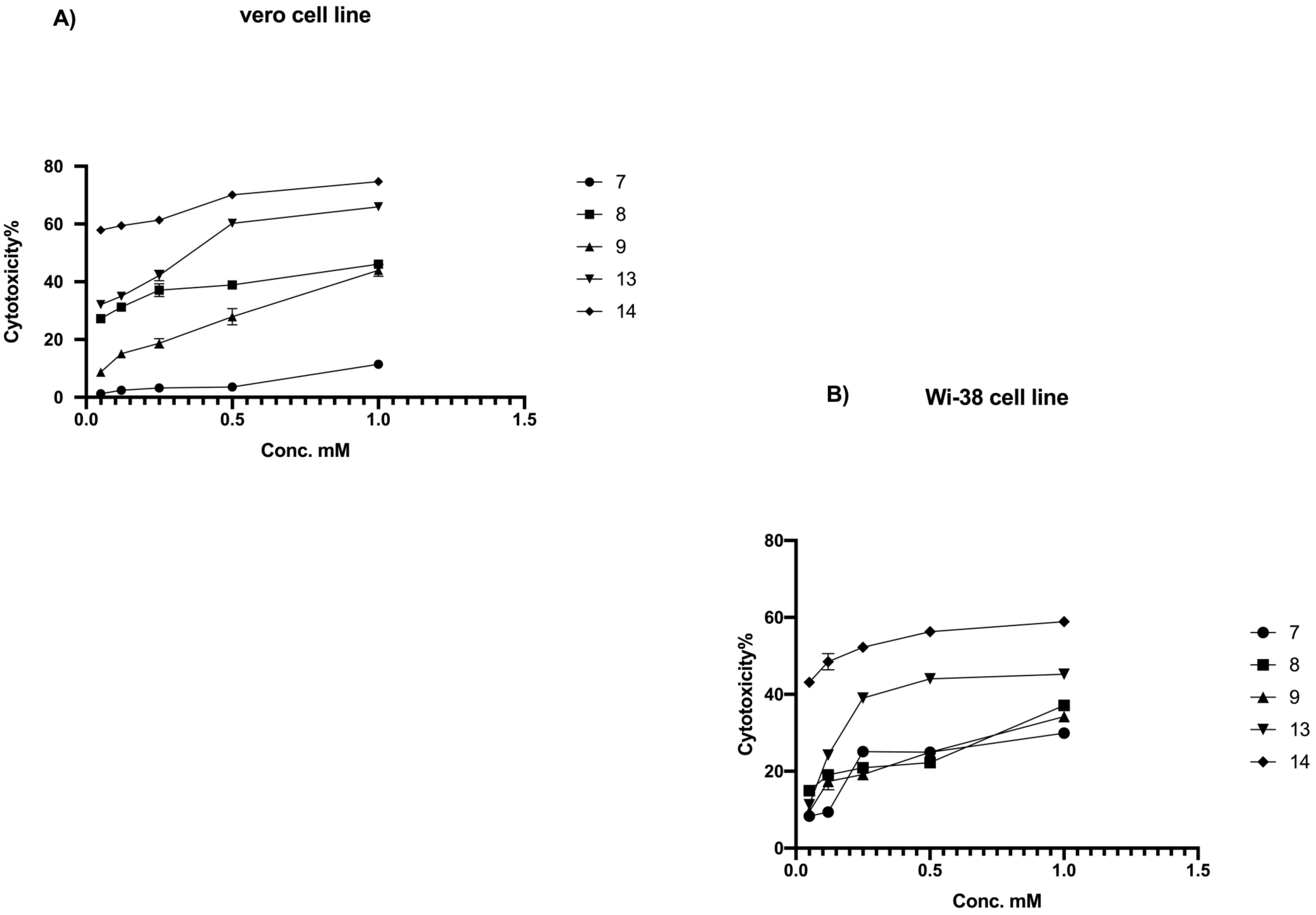
A)
vero cell line
Cytotoxicity%
Conc. mM
80
60
40
20
0
0.0
0.5
1.0
1.5
7
8
9
13
14
B)
Wi-38 cell line
Cytotoxicity%
Conc. mM
80
60
40
20
0
0.0
0.5
1.0
1.5
7
8
9
13
14

Best-fit values	
LogIC50	2.138
HillSlope	-0.4960
IC50	137.4

FQ1

7

IC_{50} = 137.4

R^2 = 0.8990

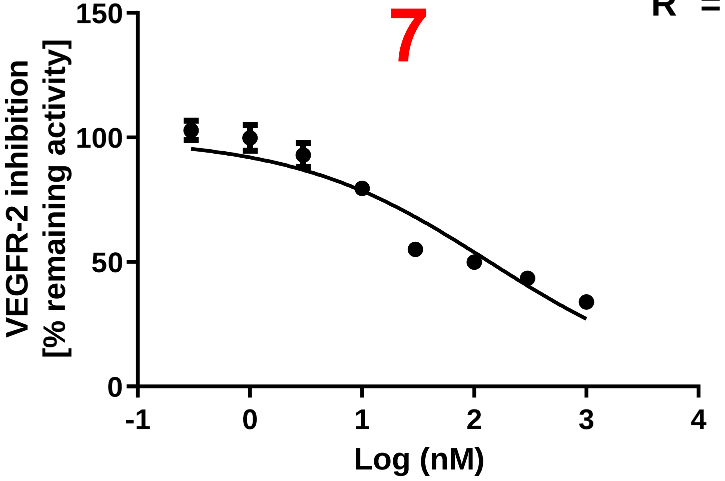

Best-fit values	
LogIC50	2.272
HillSlope	-0.3463
IC50	187.0

FQ2

8

IC_{50} = 187.0

R^2 = 0.8905

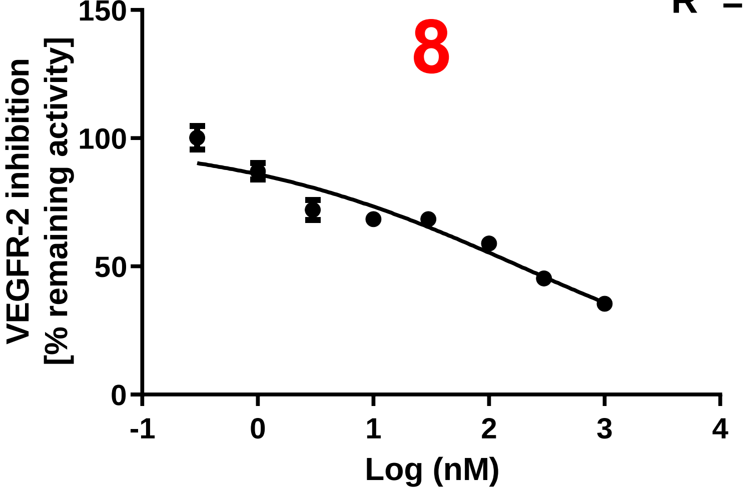

Best-fit values	
LogIC50	1.994
HillSlope	-0.5262
IC50	98.53

FQ3

9

IC_{50} = 98.53

R^2 = 0.9566

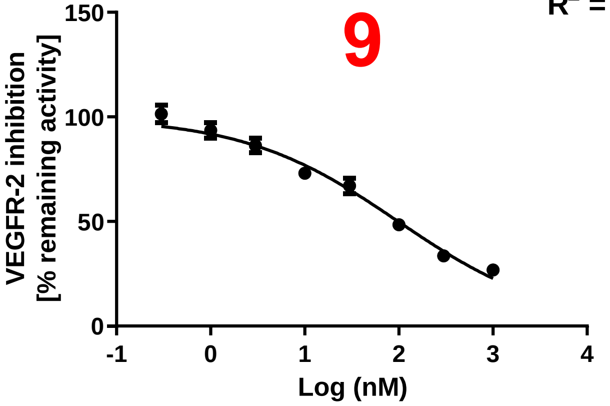

Best-fit values	
LogIC50	1.840
HillSlope	-0.4656
IC50	69.11

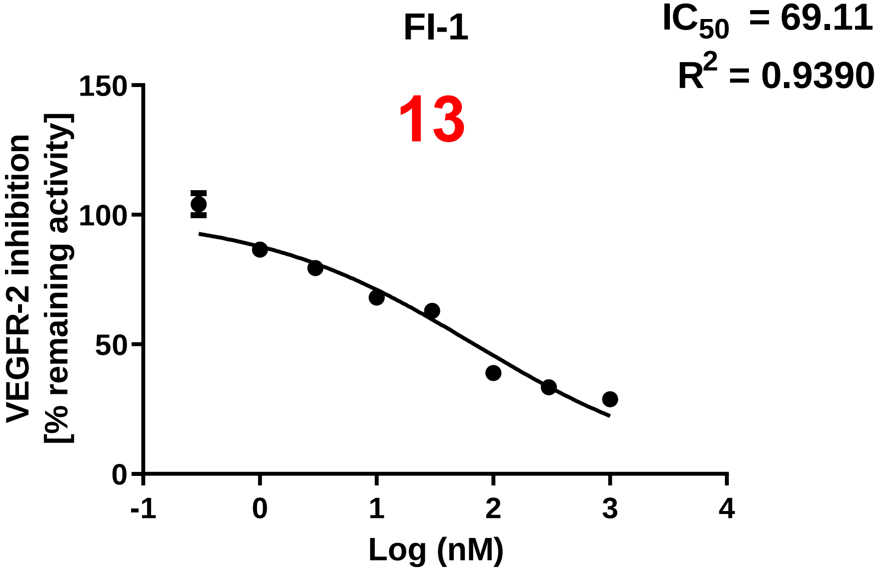

Best-fit values	
LogIC50	1.934
HillSlope	-0.3788
IC50	85.89

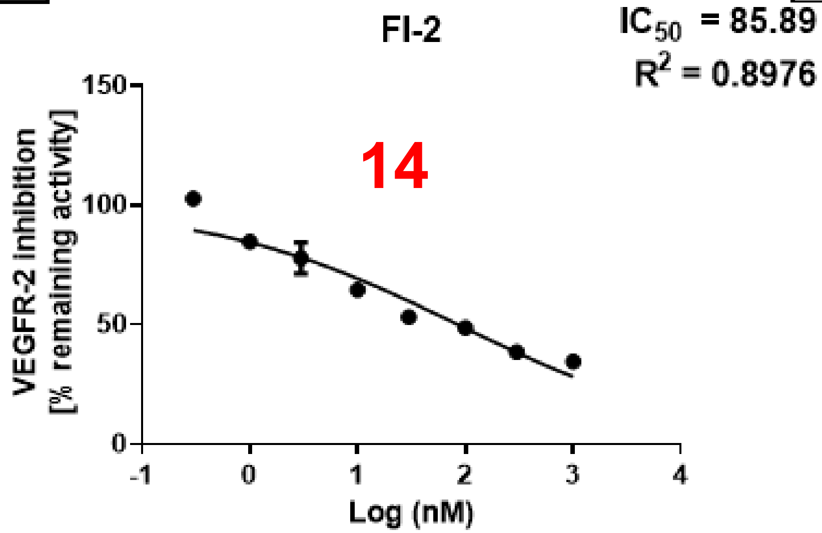

Best-fit values	
LogIC50	1.730
HillSlope	-0.4752
IC50	53.65

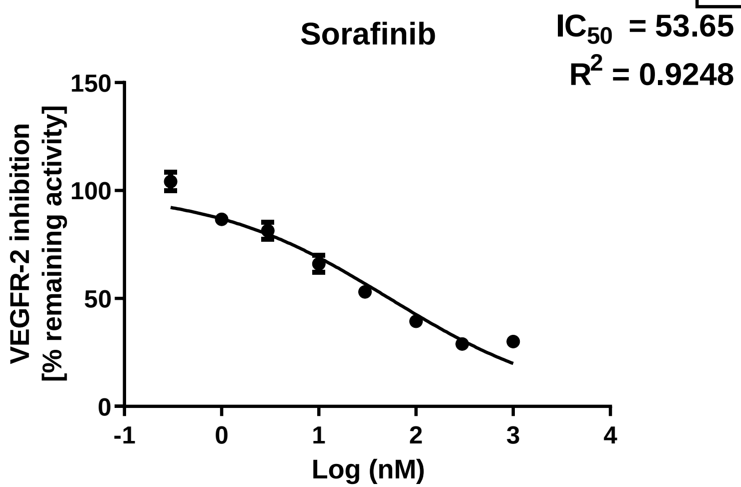

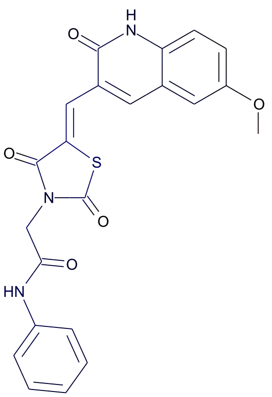

$C_{22}H_{17}N_3O_5S$
Molecular Weight: 435.45248
ALogP: 2.15
Rotatable Bonds: 5
Acceptors: 6
Donors: 2

Structural Similar Compounds

Name	Polythiazide	Delavirdine	119525-97-2
Structure	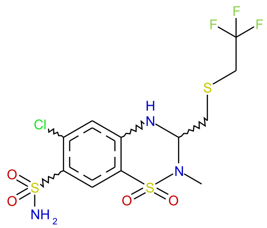	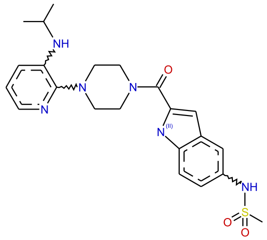	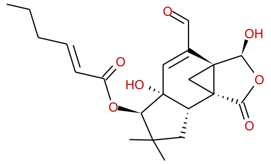
Actual Endpoint	Non-Mutagen	Non-Mutagen	Mutagen
Predicted Endpoint	Non-Mutagen	Non-Mutagen	Mutagen
Distance	0.587	0.594	0.598
Reference	Environ. Mol. Mut. 19(21):1992	Contrera, J.F., Matthews, E.J., Kruhlak, N.L., and Benz, R.D., Regulatory Toxicology and Pharmacology 2005, 313-323.	Kazius et. al., J. Med. Chem. (2005) 48, 312-320

Model Prediction

Prediction: Non-Mutagen
Probability: 0.543
Enrichment: 0.972
Bayesian Score: -6.51
Mahalanobis Distance: 11.8
Mahalanobis Distance p-value: 0.00169

Prediction: Positive if the Bayesian score is above the estimated best cutoff value from minimizing the false positive and false negative rate.
Probability: The esimated probability that the sample is in the positive category. This assumes that the Bayesian score follows a normal distribution and is different from the prediction using a cutoff.
Enrichment: An estimate of enrichment, that is, the increased likelihood (versus random) of this sample being in the category.
Bayesian Score: The standard Laplacian-modified Bayesian score.
Mahalanobis Distance: The Mahalanobis distance (MD) is the distance to the center of the training data. The larger the MD, the less trustworthy the prediction.
Mahalanobis Distance p-value: The p-value gives the fraction of training data with an MD greater than or equal to the one for the given sample, assuming normally distributed data. The smaller the p-value, the less trustworthy the prediciton. For highly non-normal X properties (e.g., fingerprints), the MD p-value is wildly inaccurate.

Model Applicability

Unknown features are fingerprint features in the query molecule, but not found or appearing too infrequently in the training set.

1. All properties and OPS components are within expected ranges.

Feature Contribution

Top features for positive contribution				
Fingerprint	Bit/Smiles	Feature Structure	Score	Mutagen in training set
SCFP_12	-577289847	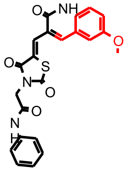 [*][c]1:[cH]:[cH]:[c](OC):[cH]:[c]:1C=[*]	0.399	9 out of 10

SCFP_12	-1971137145	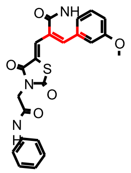 [*]C(=C[c](:[*]):[*]) [*]	0.167	225 out of 333
SCFP_12	-1379591900	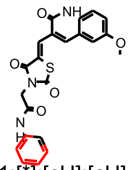 [*][c]1:[*]:[cH]:[cH] :[cH]:[cH]:1	0.108	1480 out of 2326

Top Features for negative contribution

**Fingerprint**	**Bit/Smiles**	**Feature Structure**	**Score**	**Mutagen in training set**
SCFP_12	-316886873	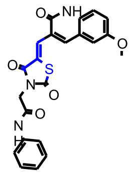 [*]\C=C\1/S[*][*]C1=[*]	-0.998	0 out of 3
SCFP_12	-1630519606	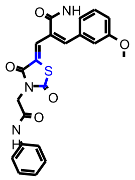 [*]=C1[*][*]C(=[*])S1	-0.998	0 out of 3
SCFP_12	399659969	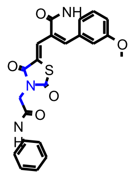 [*]CN1C(=[*])[*][*]C1 =[*]	-0.55	21 out of 65

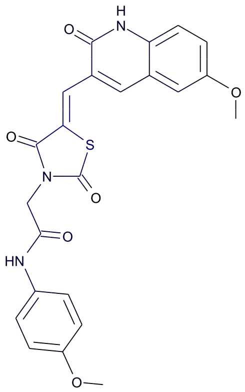

$C_{23}H_{19}N_3O_6S$
Molecular Weight: 465.47846
ALogP: 2.134
Rotatable Bonds: 6
Acceptors: 7
Donors: 2

Structural Similar Compounds

Name	Delavirdine	119525-97-2	7336-20-1
Structure	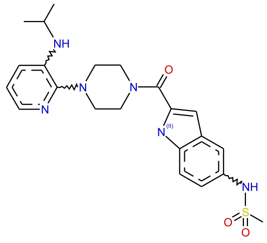	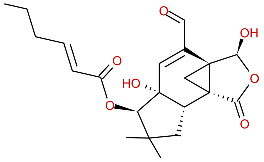	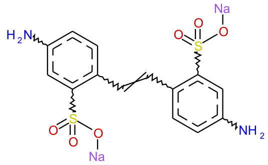
Actual Endpoint	Non-Mutagen	Mutagen	Non-Mutagen
Predicted Endpoint	Non-Mutagen	Mutagen	Non-Mutagen
Distance	0.550	0.595	0.620
Reference	Contrera, J.F., Matthews, E.J., Kruhlak, N.L., and Benz, R.D., Regulatory Toxicology and Pharmacology 2005, 313-323.	Kazius et. al., J. Med. Chem. (2005) 48, 312-320	Helma, C., Cramer, T., Kramer, S., and De Raedt, L., J. Chem. Inf. Comput. Sci., 2004, pp. 1402-1411

Model Prediction

Prediction: Non-Mutagen
Probability: 0.556
Enrichment: 0.995
Bayesian Score: -6.19
Mahalanobis Distance: 11.9
Mahalanobis Distance p-value: 0.0014

Prediction: Positive if the Bayesian score is above the estimated best cutoff value from minimizing the false positive and false negative rate.
Probability: The esimated probability that the sample is in the positive category. This assumes that the Bayesian score follows a normal distribution and is different from the prediction using a cutoff.
Enrichment: An estimate of enrichment, that is, the increased likelihood (versus random) of this sample being in the category.
Bayesian Score: The standard Laplacian-modified Bayesian score.
Mahalanobis Distance: The Mahalanobis distance (MD) is the distance to the center of the training data. The larger the MD, the less trustworthy the prediction.
Mahalanobis Distance p-value: The p-value gives the fraction of training data with an MD greater than or equal to the one for the given sample, assuming normally distributed data. The smaller the p-value, the less trustworthy the prediciton. For highly non-normal X properties (e.g., fingerprints), the MD p-value is wildly inaccurate.

Model Applicability

Unknown features are fingerprint features in the query molecule, but not found or appearing too infrequently in the training set.

1. All properties and OPS components are within expected ranges.

Feature Contribution

Top features for positive contribution

Fingerprint	Bit/Smiles	Feature Structure	Score	Mutagen in training set
SCFP_12	-577289847	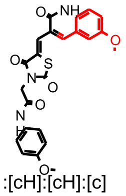 [*][c]1:[cH]:[cH]:[c](OC):[cH]:[c]:1C=[*]	0.399	9 out of 10

SCFP_12	-332023286	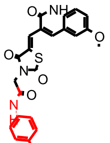 [*]C(=[*])N[c]1:[cH]:[cH]:[c](OC):[cH]:[cH]:1	0.241	1 out of 1
SCFP_12	-1971137145	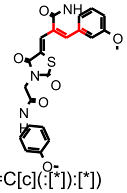 [*]C(=C[c](:[*]):[*])[*]	0.167	225 out of 333

Top Features for negative contribution

**Fingerprint**	**Bit/Smiles**	**Feature Structure**	**Score**	**Mutagen in training set**
SCFP_12	-1630519606	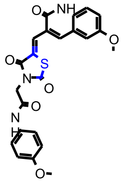 [*]=C1[*][*]C(=[*])S1	-0.998	0 out of 3
SCFP_12	-316886873	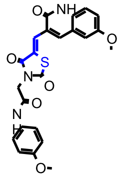 [*]\C=C\1/S[*][*]C1=[*]	-0.998	0 out of 3
SCFP_12	399659969	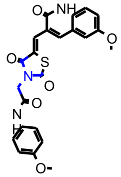 [*]CN1C(=[*])[*][*]C1=[*]	-0.55	21 out of 65

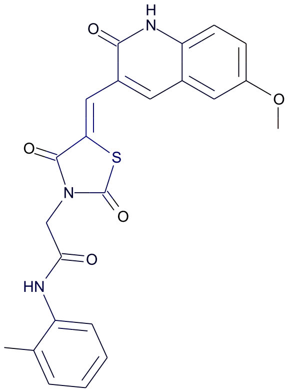

$C_{23}H_{19}N_3O_5S$
Molecular Weight: 449.47906
ALogP: 2.637
Rotatable Bonds: 5
Acceptors: 6
Donors: 2

Structural Similar Compounds

Name	55256-55-8	Delavirdine	Ochratoxin A
Structure	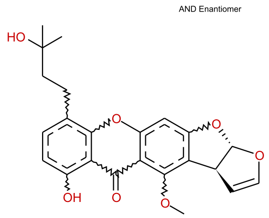	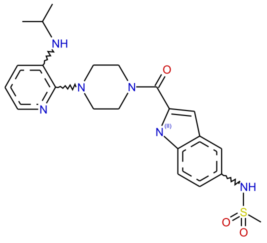	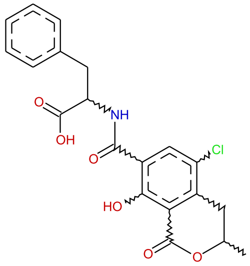
Actual Endpoint	Mutagen	Non-Mutagen	Non-Mutagen
Predicted Endpoint	Mutagen	Non-Mutagen	Non-Mutagen
Distance	0.593	0.595	0.602
Reference	Kazius et. al., J. Med. Chem. (2005) 48, 312-320	Contrera, J.F., Matthews, E.J., Kruhlak, N.L., and Benz, R.D., Regulatory Toxicology and Pharmacology 2005, 313-323.	EMIC

Model Prediction

Prediction: Non-Mutagen
Probability: 0.557
Enrichment: 0.997
Bayesian Score: -6.16
Mahalanobis Distance: 11.8
Mahalanobis Distance p-value: 0.00163

Prediction: Positive if the Bayesian score is above the estimated best cutoff value from minimizing the false positive and false negative rate.
Probability: The esimated probability that the sample is in the positive category. This assumes that the Bayesian score follows a normal distribution and is different from the prediction using a cutoff.
Enrichment: An estimate of enrichment, that is, the increased likelihood (versus random) of this sample being in the category.
Bayesian Score: The standard Laplacian-modified Bayesian score.
Mahalanobis Distance: The Mahalanobis distance (MD) is the distance to the center of the training data. The larger the MD, the less trustworthy the prediction.
Mahalanobis Distance p-value: The p-value gives the fraction of training data with an MD greater than or equal to the one for the given sample, assuming normally distributed data. The smaller the p-value, the less trustworthy the prediciton. For highly non-normal X properties (e.g., fingerprints), the MD p-value is wildly inaccurate.

Model Applicability

Unknown features are fingerprint features in the query molecule, but not found or appearing too infrequently in the training set.

1. All properties and OPS components are within expected ranges.

Feature Contribution

Top features for positive contribution

Fingerprint	Bit/Smiles	Feature Structure	Score	Mutagen in training set
SCFP_12	-577289847	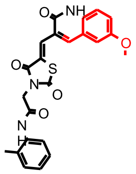 [*][c]1:[cH]:[cH]:[c](OC):[cH]:[c]:1C=[*]	0.399	9 out of 10

SCFP_12	-1971137145	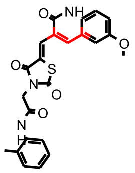 [*]C(=C[c](:[*]):[*])[*]	0.167	225 out of 333
SCFP_12	136686699	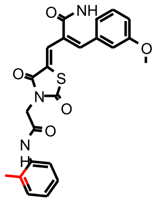 [*]:[c](:[*])C	0.129	446 out of 686
**Top Features for negative contribution**				
**Fingerprint**	**Bit/Smiles**	**Feature Structure**	**Score**	**Mutagen in training set**
SCFP_12	-1630519606	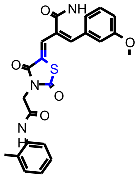 [*]=C1[*][*]C(=[*])S1	-0.998	0 out of 3
SCFP_12	-316886873	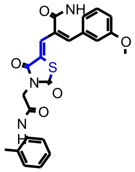 [*]\C=C\1/S[*][*]C1=[*]	-0.998	0 out of 3
SCFP_12	399659969	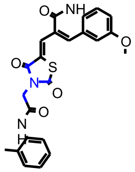 [*]CN1C(=[*])[*][*]C1=[*]	-0.55	21 out of 65

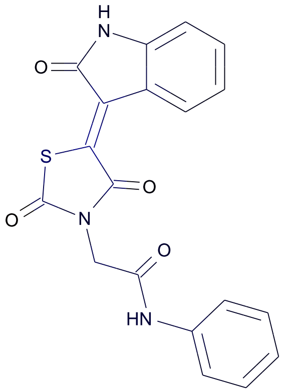

$C_{19}H_{13}N_3O_4S$

Molecular Weight: 379.38922

ALogP: 1.699

Rotatable Bonds: 3

Acceptors: 5

Donors: 2

Model Prediction

Prediction: Non-Mutagen

Probability: 0.582

Enrichment: 1.04

Bayesian Score: -5.51

Mahalanobis Distance: 11.1

Mahalanobis Distance p-value: 0.0257

Prediction: Positive if the Bayesian score is above the estimated best cutoff value from minimizing the false positive and false negative rate.
Probability: The esimated probability that the sample is in the positive category. This assumes that the Bayesian score follows a normal distribution and is different from the prediction using a cutoff.
Enrichment: An estimate of enrichment, that is, the increased likelihood (versus random) of this sample being in the category.
Bayesian Score: The standard Laplacian-modified Bayesian score.
Mahalanobis Distance: The Mahalanobis distance (MD) is the distance to the center of the training data. The larger the MD, the less trustworthy the prediction.
Mahalanobis Distance p-value: The p-value gives the fraction of training data with an MD greater than or equal to the one for the given sample, assuming normally distributed data. The smaller the p-value, the less trustworthy the prediciton. For highly non-normal X properties (e.g., fingerprints), the MD p-value is wildly inaccurate.

Structural Similar Compounds

Name	PENICILLIN G POTASSIUM	77-46-3	97919-22-7
Structure	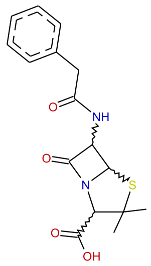	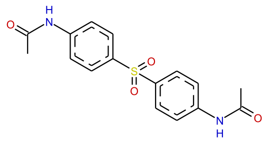	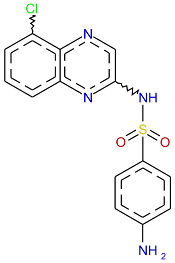
Actual Endpoint	Non-Mutagen	Non-Mutagen	Non-Mutagen
Predicted Endpoint	Non-Mutagen	Non-Mutagen	Non-Mutagen
Distance	0.556	0.560	0.561
Reference	EMIC	Kazius et. al., J. Med. Chem. (2005) 48, 312-320	Kazius et. al., J. Med. Chem. (2005) 48, 312-320

Model Applicability

Unknown features are fingerprint features in the query molecule, but not found or appearing too infrequently in the training set.

1. All properties and OPS components are within expected ranges.

Feature Contribution

Top features for positive contribution				
**Fingerprint**	**Bit/Smiles**	**Feature Structure**	**Score**	**Mutagen in training set**
SCFP_12	-1379591900	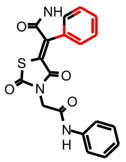 [*][c]1:[*]:[cH]:[cH]:[cH]:[cH]:1	0.108	1480 out of 2326

SCFP_12	-834984590	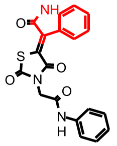 [*]=C1N[c]2:[cH]:[cH]:[cH]:[cH]:[c]:2C1=[*]	0.1	2 out of 3
SCFP_12	-496409612	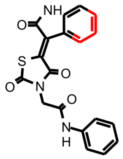 [*][c](:[*]):[cH]:[cH]:[*]	0.0771	2616 out of 4239

Top Features for negative contribution

Fingerprint	Bit/Smiles	Feature Structure	Score	Mutagen in training set
SCFP_12	-316886873	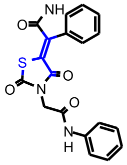 [*]\C=C\1/S[*][*]C1=[*]	-0.998	0 out of 3
SCFP_12	-1630519606	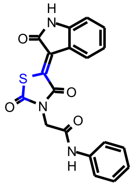 [*]=C1[*][*]C(=[*])S1	-0.998	0 out of 3
SCFP_12	399659969	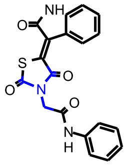 [*]CN1C(=[*])[*][*]C1=[*]	-0.55	21 out of 65

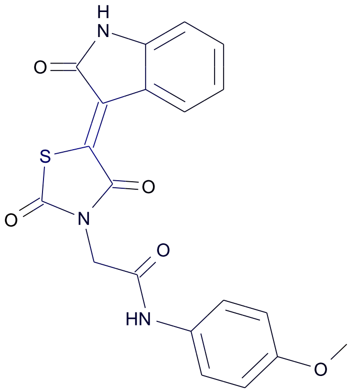

$C_{20}H_{15}N_3O_5S$
Molecular Weight: 409.4152
ALogP: 1.683
Rotatable Bonds: 4
Acceptors: 6
Donors: 2

Model Prediction

Prediction: Non-Mutagen
Probability: 0.562
Enrichment: 1.01
Bayesian Score: -6.04
Mahalanobis Distance: 11.5
Mahalanobis Distance p-value: 0.00573

Prediction: Positive if the Bayesian score is above the estimated best cutoff value from minimizing the false positive and false negative rate.
Probability: The esimated probability that the sample is in the positive category. This assumes that the Bayesian score follows a normal distribution and is different from the prediction using a cutoff.
Enrichment: An estimate of enrichment, that is, the increased likelihood (versus random) of this sample being in the category.
Bayesian Score: The standard Laplacian-modified Bayesian score.
Mahalanobis Distance: The Mahalanobis distance (MD) is the distance to the center of the training data. The larger the MD, the less trustworthy the prediction.
Mahalanobis Distance p-value: The p-value gives the fraction of training data with an MD greater than or equal to the one for the given sample, assuming normally distributed data. The smaller the p-value, the less trustworthy the prediciton. For highly non-normal X properties (e.g., fingerprints), the MD p-value is wildly inaccurate.

Structural Similar Compounds

Name	Polythiazide	PENICILLIN G POTASSIUM	77-46-3
Structure	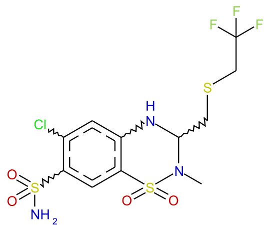	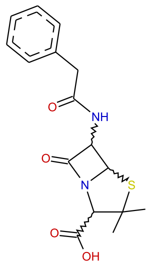	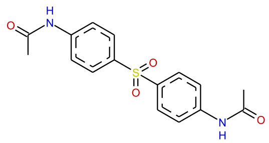
Actual Endpoint	Non-Mutagen	Non-Mutagen	Non-Mutagen
Predicted Endpoint	Non-Mutagen	Non-Mutagen	Non-Mutagen
Distance	0.577	0.595	0.620
Reference	Environ. Mol. Mut. 19(21):1992	EMIC	Kazius et. al., J. Med. Chem. (2005) 48, 312-320

Model Applicability

Unknown features are fingerprint features in the query molecule, but not found or appearing too infrequently in the training set.

1. All properties and OPS components are within expected ranges.

Feature Contribution

Top features for positive contribution				
**Fingerprint**	**Bit/Smiles**	**Feature Structure**	**Score**	**Mutagen in training set**
SCFP_12	-332023286	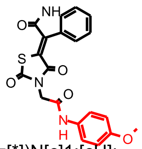 [*]C(=[*])N[c]1:[cH]:[cH]:[c](OC):[cH]:[cH]:1	0.241	1 out of 1

SCFP_12	-1379591900	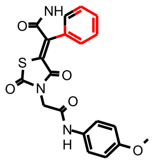 [*][c]1:[*]:[cH]:[cH]:[cH]:[cH]:1	0.108	1480 out of 2326
SCFP_12	-834984590	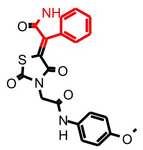 [*]=C1N[c]2:[cH]:[cH]:[cH]:[cH]:[c]:2C1=[*]	0.1	2 out of 3

Top Features for negative contribution

Fingerprint	Bit/Smiles	Feature Structure	Score	Mutagen in training set
SCFP_12	-316886873	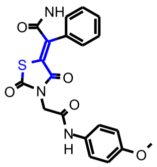 [*]\C=C\1/S[*][*]C1=[*]	-0.998	0 out of 3
SCFP_12	-1630519606	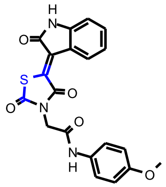 [*]=C1[*][*]C(=[*])S1	-0.998	0 out of 3
SCFP_12	399659969	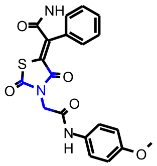 [*]CN1C(=[*])[*][*]C1=[*]	-0.55	21 out of 65

Sorafenib TOPKAT_Ames_Mutagenicity

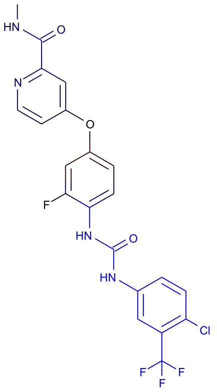

$C_{21}H_{15}ClF_4N_4O_3$
Molecular Weight: 482.81541
ALogP: 4.381
Rotatable Bonds: 6
Acceptors: 4
Donors: 3

Structural Similar Compounds

Name	GLYBURIDE	93957-54-1	38914-96-4
Structure	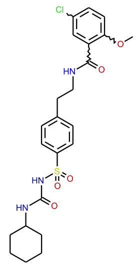	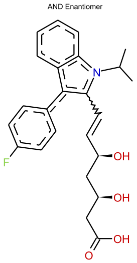	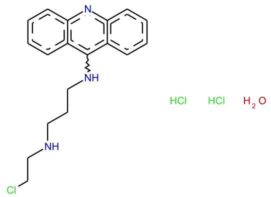
Actual Endpoint	Non-Mutagen	Non-Mutagen	Mutagen
Predicted Endpoint	Non-Mutagen	Non-Mutagen	Mutagen
Distance	0.591	0.610	0.612
Reference	PDR 1994	US Environmental Protection Agency at http://www.epa.gov/NCCT/dsstox/sdf_isscan_external.html	Kazius et. al., J. Med. Chem. (2005) 48, 312-320

Model Prediction

Prediction: Non-Mutagen
Probability: 0.134
Enrichment: 0.24
Bayesian Score: -16.1
Mahalanobis Distance: 13.1
Mahalanobis Distance p-value: 2.19e-006

Prediction: Positive if the Bayesian score is above the estimated best cutoff value from minimizing the false positive and false negative rate.
Probability: The esimated probability that the sample is in the positive category. This assumes that the Bayesian score follows a normal distribution and is different from the prediction using a cutoff.
Enrichment: An estimate of enrichment, that is, the increased likelihood (versus random) of this sample being in the category.
Bayesian Score: The standard Laplacian-modified Bayesian score.
Mahalanobis Distance: The Mahalanobis distance (MD) is the distance to the center of the training data. The larger the MD, the less trustworthy the prediction.
Mahalanobis Distance p-value: The p-value gives the fraction of training data with an MD greater than or equal to the one for the given sample, assuming normally distributed data. The smaller the p-value, the less trustworthy the prediciton. For highly non-normal X properties (e.g., fingerprints), the MD p-value is wildly inaccurate.

Model Applicability

Unknown features are fingerprint features in the query molecule, but not found or appearing too infrequently in the training set.

1. All properties and OPS components are within expected ranges.

Feature Contribution

Top features for positive contribution

Fingerprint	Bit/Smiles	Feature Structure	Score	Mutagen in training set
SCFP_12	-347281112	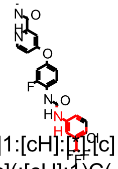 [*]N[c]1:[cH]:[c]:[c]([*]):[c](:[cH]:1)C([*])([*])[*]	0.337	18 out of 22

SCFP_12	1208843554	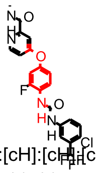 [*]N[c]1:[cH]:[cH]:[c](O[c](:[*]):[*]):[cH]:[c]:1[*]	0.337	6 out of 7
SCFP_12	-711471096	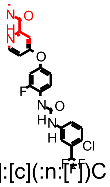 [*]:[cH]:[c](:n:[*])C(=O)NC	0.264	7 out of 9
**Top Features for negative contribution**				
**Fingerprint**	**Bit/Smiles**	**Feature Structure**	**Score**	**Mutagen in training set**
SCFP_12	-1903175541	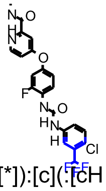 [*][c](:[*]):[c](:[cH]:[*])C(F)(F)F	-1.51	3 out of 30
SCFP_12	-300280774	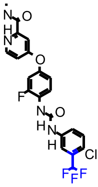 [*]:[c](:[*])C(F)(F)F	-1.51	3 out of 30
SCFP_12	2102306623	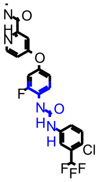 [*]NC(=O)N[c](:[cH]:[*]):[c]([*]):[*]	-1.18	3 out of 21

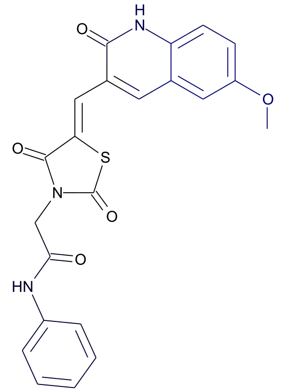

$C_{22}H_{17}N_3O_5S$
Molecular Weight: 435.45248
ALogP: 2.15
Rotatable Bonds: 5
Acceptors: 6
Donors: 2

Structural Similar Compounds

Name	ANTHRAQUINONE; 1-AMINO-4-HYDROXY-2-PHENOXY-	5-NORBORNENE-2;3-DICARBOXYLIC ACID; 1;4;5;6;7;7-HEXACHLORO-	1-AMINO-4-BENZOYLAMINO-ANTHRAQUINONE
Structure	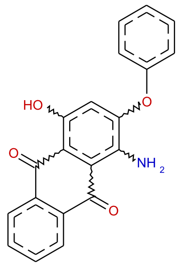	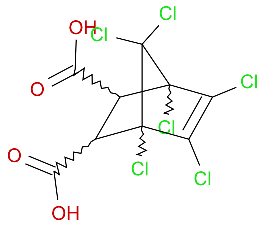	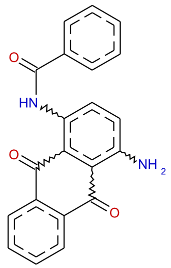
Actual Endpoint	Mild	Moderate_Severe	Mild
Predicted Endpoint	Mild	Moderate_Severe	Mild
Distance	0.716	0.726	0.729
Reference	28ZPAK 239;72	28ZPAK-;92;72	28ZPAK-;124;72

Model Prediction

Prediction: Mild
Probability: 0.637
Enrichment: 0.925
Bayesian Score: -4.51
Mahalanobis Distance: 11
Mahalanobis Distance p-value: 0.00529

Prediction: Positive if the Bayesian score is above the estimated best cutoff value from minimizing the false positive and false negative rate.
Probability: The esimated probability that the sample is in the positive category. This assumes that the Bayesian score follows a normal distribution and is different from the prediction using a cutoff.
Enrichment: An estimate of enrichment, that is, the increased likelihood (versus random) of this sample being in the category.
Bayesian Score: The standard Laplacian-modified Bayesian score.
Mahalanobis Distance: The Mahalanobis distance (MD) is the distance to the center of the training data. The larger the MD, the less trustworthy the prediction.
Mahalanobis Distance p-value: The p-value gives the fraction of training data with an MD greater than or equal to the one for the given sample, assuming normally distributed data. The smaller the p-value, the less trustworthy the prediciton. For highly non-normal X properties (e.g., fingerprints), the MD p-value is wildly inaccurate.

Model Applicability

Unknown features are fingerprint features in the query molecule, but not found or appearing too infrequently in the training set.

1. All properties and OPS components are within expected ranges.

Feature Contribution

Top features for positive contribution

Fingerprint	Bit/Smiles	Feature Structure	Score	Moderate_Severe in training set
FCFP_10	346218766	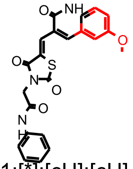 [*][c]1:[*]:[cH]:[cH]:[c](OC):[cH]:1	0.197	30 out of 37

FCFP_10	3	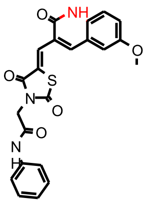 [*]N[*]	0.165	383 out of 491
FCFP_10	436886043	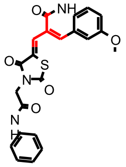 [*]\C=C(\C=[*])/C(=[*])[*]	0.16	25 out of 32
**Top Features for negative contribution**				
**Fingerprint**	**Bit/Smiles**	**Feature Structure**	**Score**	**Moderate_Severe in training set**
FCFP_10	-1977641857	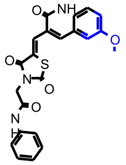 [*]:[cH]:[c](OC):[cH]:[*]	-0.78	4 out of 15
FCFP_10	723745966	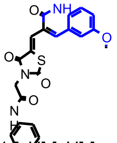 [*]O[c]1:[cH]:[cH]:[c]2NC(=[*])[*]=C[c]:2:[cH]:1	-0.507	0 out of 1
FCFP_10	-1757681964	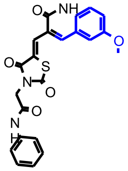 [*][c]1:[cH]:[cH]:[c](OC):[cH]:[c]:1C=[*]	-0.507	0 out of 1

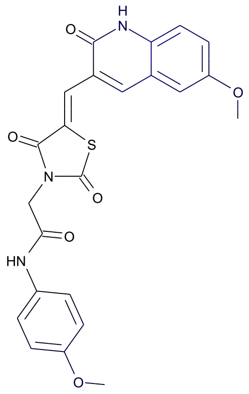

$C_{23}H_{19}N_3O_6S$
Molecular Weight: 465.47846
ALogP: 2.134
Rotatable Bonds: 6
Acceptors: 7
Donors: 2

Model Prediction

Prediction: Mild
Probability: 0.68
Enrichment: 0.987
Bayesian Score: -3.86
Mahalanobis Distance: 11
Mahalanobis Distance p-value: 0.00468

Prediction: Positive if the Bayesian score is above the estimated best cutoff value from minimizing the false positive and false negative rate.
Probability: The esimated probability that the sample is in the positive category. This assumes that the Bayesian score follows a normal distribution and is different from the prediction using a cutoff.
Enrichment: An estimate of enrichment, that is, the increased likelihood (versus random) of this sample being in the category.
Bayesian Score: The standard Laplacian-modified Bayesian score.
Mahalanobis Distance: The Mahalanobis distance (MD) is the distance to the center of the training data. The larger the MD, the less trustworthy the prediction.
Mahalanobis Distance p-value: The p-value gives the fraction of training data with an MD greater than or equal to the one for the given sample, assuming normally distributed data. The smaller the p-value, the less trustworthy the prediciton. For highly non-normal X properties (e.g., fingerprints), the MD p-value is wildly inaccurate.

Structural Similar Compounds

Name	COLCHICINE	4;4'-DIAMINO-1;1'-DIANTHRIMIDE	ANTHRAQUINONE; 1-AMINO-4-HYDROXY-2-PHENOXY-
Structure	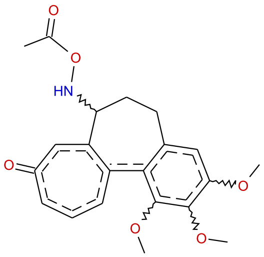	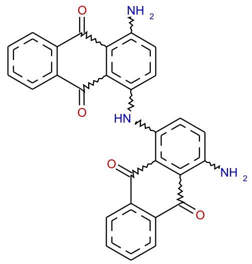	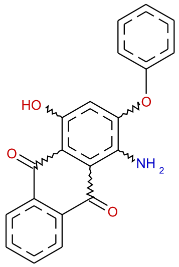
Actual Endpoint	Moderate_Severe	Mild	Mild
Predicted Endpoint	Moderate_Severe	Mild	Mild
Distance	0.760	0.777	0.811
Reference	AJOPAA 31;837;48	28ZPAK-;125;72	28ZPAK 239;72

Model Applicability

Unknown features are fingerprint features in the query molecule, but not found or appearing too infrequently in the training set.

1. All properties and OPS components are within expected ranges.

Feature Contribution

Top features for positive contribution				
**Fingerprint**	**Bit/Smiles**	**Feature Structure**	**Score**	**Moderate_Severe in training set**
FCFP_10	346218766	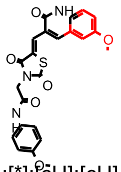 [*][c]1:[*]:[cH]:[cH]:[c](OC):[cH]:1	0.197	30 out of 37

FCFP_10	70423256	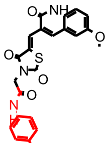 [*]C(=[*])N[c]1:[cH]:[cH]:[c](OC):[cH]:[cH]:1	0.186	1 out of 1
FCFP_10	3	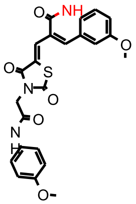 [*]N[*]	0.165	383 out of 491

Top Features for negative contribution

Fingerprint	Bit/Smiles	Feature Structure	Score	Moderate_Severe in training set
FCFP_10	-1977641857	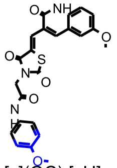 [*]:[cH]:[c](OC):[cH]:[*]	-0.78	4 out of 15
FCFP_10	-1757681964	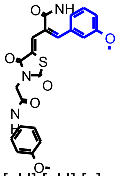 [*][c]1:[cH]:[cH]:[c](OC):[cH]:[c]:1C=[*]	-0.507	0 out of 1
FCFP_10	723745966	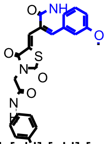 [*]O[c]1:[cH]:[cH]:[c]2NC(=[*])[*]=C[c]:2:[cH]:1	-0.507	0 out of 1

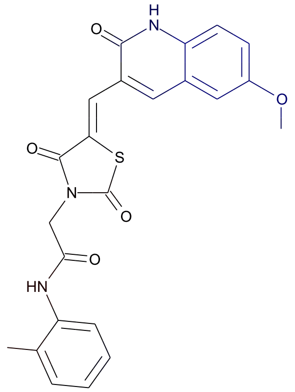

$C_{23}H_{19}N_3O_5S$

Molecular Weight: 449.47906

ALogP: 2.637

Rotatable Bonds: 5

Acceptors: 6

Donors: 2

Structural Similar Compounds

Name	ANTHRAQUINONE; 1-AMINO-4-HYDROXY-2-PHENOXY-	5-NORBORNENE-2;3-DICARBOXYLIC ACID; 1;4;5;6;7;7-HEXACHLORO-	1-AMINO-4-BENZOYLAMINO-ANTHRAQUINONE
Structure	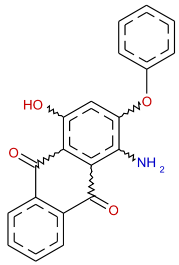	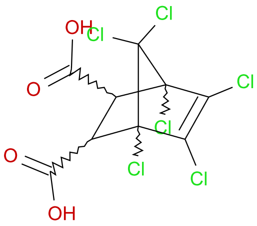	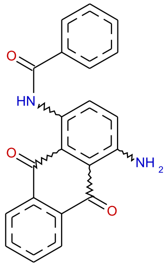
Actual Endpoint	Mild	Moderate_Severe	Mild
Predicted Endpoint	Mild	Moderate_Severe	Mild
Distance	0.709	0.726	0.730
Reference	28ZPAK 239;72	28ZPAK-;92;72	28ZPAK-;124;72

Model Prediction

Prediction: Mild

Probability: 0.71

Enrichment: 1.03

Bayesian Score: -3.34

Mahalanobis Distance: 11

Mahalanobis Distance p-value: 0.00428

Prediction: Positive if the Bayesian score is above the estimated best cutoff value from minimizing the false positive and false negative rate.
Probability: The esimated probability that the sample is in the positive category. This assumes that the Bayesian score follows a normal distribution and is different from the prediction using a cutoff.
Enrichment: An estimate of enrichment, that is, the increased likelihood (versus random) of this sample being in the category.
Bayesian Score: The standard Laplacian-modified Bayesian score.
Mahalanobis Distance: The Mahalanobis distance (MD) is the distance to the center of the training data. The larger the MD, the less trustworthy the prediction.
Mahalanobis Distance p-value: The p-value gives the fraction of training data with an MD greater than or equal to the one for the given sample, assuming normally distributed data. The smaller the p-value, the less trustworthy the prediciton. For highly non-normal X properties (e.g., fingerprints), the MD p-value is wildly inaccurate.

Model Applicability

Unknown features are fingerprint features in the query molecule, but not found or appearing too infrequently in the training set.

1. All properties and OPS components are within expected ranges.

Feature Contribution

Top features for positive contribution

Fingerprint	Bit/Smiles	Feature Structure	Score	Moderate_Severe in training set
FCFP_10	1396506317	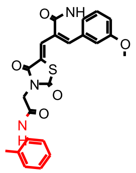 [*]N[c]1:[cH]:[cH]:[cH]:[cH]:[c]:1C	0.317	4 out of 4

FCFP_10	755520106	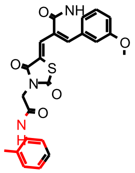 [*]N[c]1:[cH]:[*]:[cH]:[cH]:[c]:1C	0.273	9 out of 10
FCFP_10	136120670	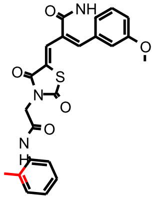 [*]:[c](:[*])C	0.206	53 out of 65
**Top Features for negative contribution**				
**Fingerprint**	**Bit/Smiles**	**Feature Structure**	**Score**	**Moderate_Severe in training set**
FCFP_10	-1977641857	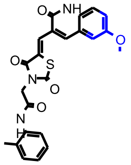 [*]:[cH]:[c](OC):[cH] :[*]	-0.78	4 out of 15
FCFP_10	723745966	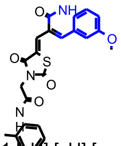 [*]O[c]1:[cH]:[cH]:[c]2NC(=[*])[*]=C[c]:2 :[cH]:1	-0.507	0 out of 1
FCFP_10	-1757681964	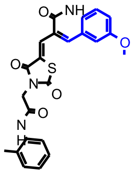 [*][c]1:[cH]:[cH]:[c] (OC):[cH]:[c]:1C=[*]	-0.507	0 out of 1

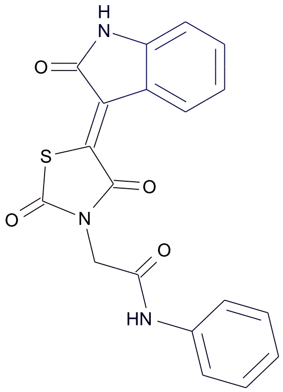

$C_{19}H_{13}N_3O_4S$
Molecular Weight: 379.38922
ALogP: 1.699
Rotatable Bonds: 3
Acceptors: 5
Donors: 2

Model Prediction

Prediction: Mild
Probability: 0.743
Enrichment: 1.08
Bayesian Score: -2.66
Mahalanobis Distance: 10.3
Mahalanobis Distance p-value: 0.0408

Prediction: Positive if the Bayesian score is above the estimated best cutoff value from minimizing the false positive and false negative rate.
Probability: The esimated probability that the sample is in the positive category. This assumes that the Bayesian score follows a normal distribution and is different from the prediction using a cutoff.
Enrichment: An estimate of enrichment, that is, the increased likelihood (versus random) of this sample being in the category.
Bayesian Score: The standard Laplacian-modified Bayesian score.
Mahalanobis Distance: The Mahalanobis distance (MD) is the distance to the center of the training data. The larger the MD, the less trustworthy the prediction.
Mahalanobis Distance p-value: The p-value gives the fraction of training data with an MD greater than or equal to the one for the given sample, assuming normally distributed data. The smaller the p-value, the less trustworthy the prediciton. For highly non-normal X properties (e.g., fingerprints), the MD p-value is wildly inaccurate.

Structural Similar Compounds

Name	1-AMINO-4-BENZOYLAMINO-ANTHRAQUINONE	2-NAPHTHALENESULFONIC ACID;5-AMINO-6-ETHOXY-	ANTHRAQUINONE; 1-AMINO-4-HYDROXY-2-PHENOXY-
Structure	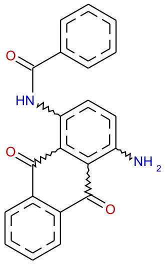	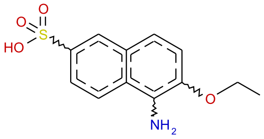	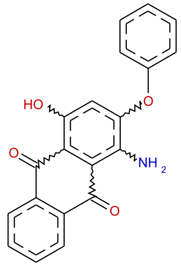
Actual Endpoint	Mild	Moderate_Severe	Mild
Predicted Endpoint	Mild	Mild	Mild
Distance	0.627	0.655	0.661
Reference	28ZPAK-;124;72	28ZPAK-;191;72	28ZPAK 239;72

Model Applicability

Unknown features are fingerprint features in the query molecule, but not found or appearing too infrequently in the training set.

1. All properties and OPS components are within expected ranges.

Feature Contribution

Top features for positive contribution				
**Fingerprint**	**Bit/Smiles**	**Feature Structure**	**Score**	**Moderate_Severe in training set**
FCFP_10	3	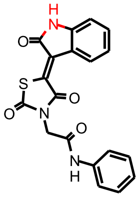 [*]N[*]	0.165	383 out of 491

FCFP_10	-1553874037	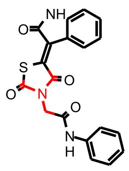<br>[*]CN1C(=[*])[*][*]C1<br>=[*]	0.107	31 out of 42
FCFP_10	2036120522	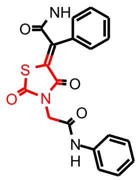<br>[*]CN1C(=O)SC(=[*])C1<br>=[*]	0.0934	3 out of 4

Top Features for negative contribution

Fingerprint	Bit/Smiles	Feature Structure	Score	Moderate_Severe in training set
FCFP_10	-792685140	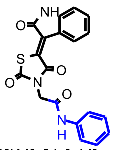<br>[*]C(=[*])N[c]1:[cH]:<br>[cH]:[cH]:[cH]:[cH]:<br>1	-0.361	2 out of 5
FCFP_10	1011367537	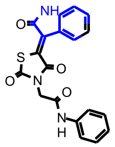<br>[*]=C1[*]=C[c]2:[cH]:<br>[*]:[cH]:[cH]:[c]:2N<br>1	-0.329	4 out of 9
FCFP_10	-773983804	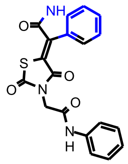<br>[*]N[c]1:[cH]:[cH]:[c<br>]([*]):[*]:[c]:1[*]	-0.294	50 out of 102

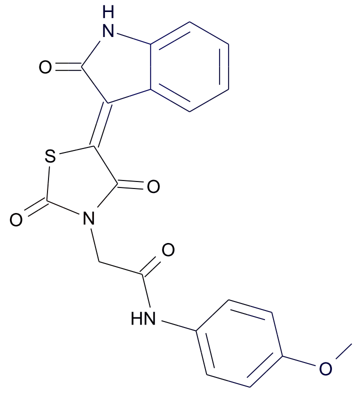

$C_{20}H_{15}N_3O_5S$
Molecular Weight: 409.4152
ALogP: 1.683
Rotatable Bonds: 4
Acceptors: 6
Donors: 2

Model Prediction

Prediction: Mild
Probability: 0.711
Enrichment: 1.03
Bayesian Score: -3.32
Mahalanobis Distance: 10.2
Mahalanobis Distance p-value: 0.0628

Prediction: Positive if the Bayesian score is above the estimated best cutoff value from minimizing the false positive and false negative rate.
Probability: The esimated probability that the sample is in the positive category. This assumes that the Bayesian score follows a normal distribution and is different from the prediction using a cutoff.
Enrichment: An estimate of enrichment, that is, the increased likelihood (versus random) of this sample being in the category.
Bayesian Score: The standard Laplacian-modified Bayesian score.
Mahalanobis Distance: The Mahalanobis distance (MD) is the distance to the center of the training data. The larger the MD, the less trustworthy the prediction.
Mahalanobis Distance p-value: The p-value gives the fraction of training data with an MD greater than or equal to the one for the given sample, assuming normally distributed data. The smaller the p-value, the less trustworthy the prediciton. For highly non-normal X properties (e.g., fingerprints), the MD p-value is wildly inaccurate.

Structural Similar Compounds

Name	ANTHRAQUINONE; 1-AMINO-4-HYDROXY-2-PHENOXY-	1-AMINO-4-BENZOYLAMINO-ANTHRAQUINONE	2-NAPHTHALENESULFONIC ACID;5-AMINO-6-ETHOXY-
Structure	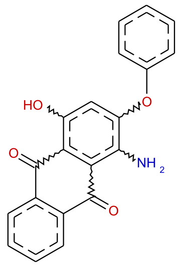	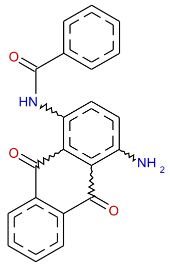	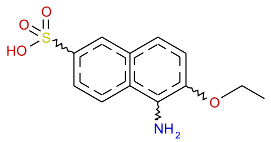
Actual Endpoint	Mild	Mild	Moderate_Severe
Predicted Endpoint	Mild	Mild	Mild
Distance	0.706	0.715	0.716
Reference	28ZPAK 239;72	28ZPAK-;124;72	28ZPAK-;191;72

Model Applicability

Unknown features are fingerprint features in the query molecule, but not found or appearing too infrequently in the training set.

1. All properties and OPS components are within expected ranges.

Feature Contribution

Top features for positive contribution				
**Fingerprint**	**Bit/Smiles**	**Feature Structure**	**Score**	**Moderate_Severe in training set**
FCFP_10	346218766	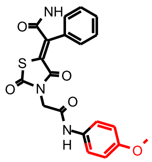 [*][c]1:[*]:[cH]:[cH]:[c](OC):[cH]:1	0.197	30 out of 37

FCFP_10	70423256	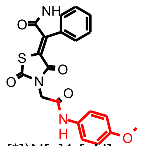 [*]C(=[*])N[c]1:[cH]:[cH]:[c](OC):[cH]:[cH]:1	0.186	1 out of 1
FCFP_10	3	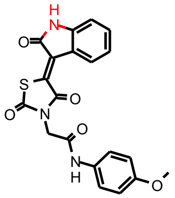 [*]N[*]	0.165	383 out of 491
**Top Features for negative contribution**				
**Fingerprint**	**Bit/Smiles**	**Feature Structure**	**Score**	**Moderate_Severe in training set**
FCFP_10	-1977641857	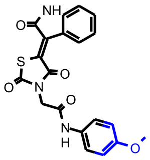 [*]:[cH]:[c](OC):[cH]:[*]	-0.78	4 out of 15
FCFP_10	-9847677	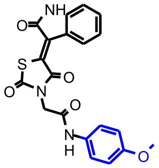 [*][c]1:[cH]:[cH]:[c](OC):[cH]:[cH]:1	-0.4	1 out of 3
FCFP_10	1011367537	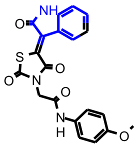 [*]=C1[*]=C[c]2:[cH]:[*]:[cH]:[cH]:[c]:2N1	-0.329	4 out of 9

Sorafenib

TOPKAT_Ocular_Irritancy_Mild_vs_Moderate_Severe

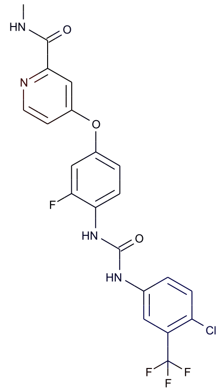

$C_{21}H_{15}ClF_4N_4O_3$

Molecular Weight: 482.81541

ALogP: 4.381

Rotatable Bonds: 6

Acceptors: 4

Donors: 3

Model Prediction

Prediction: Mild

Probability: 0.769

Enrichment: 1.12

Bayesian Score: -2.02

Mahalanobis Distance: 9.09

Mahalanobis Distance p-value: 0.463

Prediction: Positive if the Bayesian score is above the estimated best cutoff value from minimizing the false positive and false negative rate.
Probability: The esimated probability that the sample is in the positive category. This assumes that the Bayesian score follows a normal distribution and is different from the prediction using a cutoff.
Enrichment: An estimate of enrichment, that is, the increased likelihood (versus random) of this sample being in the category.
Bayesian Score: The standard Laplacian-modified Bayesian score.
Mahalanobis Distance: The Mahalanobis distance (MD) is the distance to the center of the training data. The larger the MD, the less trustworthy the prediction.
Mahalanobis Distance p-value: The p-value gives the fraction of training data with an MD greater than or equal to the one for the given sample, assuming normally distributed data. The smaller the p-value, the less trustworthy the prediciton. For highly non-normal X properties (e.g., fingerprints), the MD p-value is wildly inaccurate.

Structural Similar Compounds

Name	4;4'-DIAMINO-1;1'-DIANTHRIMIDE	ANTHRAQUINONE; 1;4-BIS(p-TOLYLAMINO)-	5-NORBORNENE-2;3-DICARBOXYLIC ACID; 1;4;5;6;7;7-HEXACHLORO-
Structure	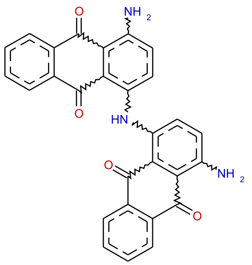	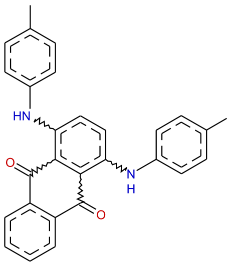	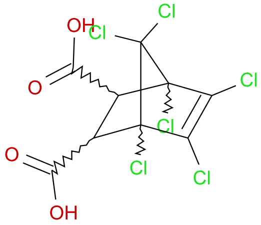
Actual Endpoint	Mild	Moderate_Severe	Moderate_Severe
Predicted Endpoint	Mild	Mild	Moderate_Severe
Distance	0.809	0.841	0.842
Reference	28ZPAK-;125;72	28ZPAK -;124;72	28ZPAK-;92;72

Model Applicability

Unknown features are fingerprint features in the query molecule, but not found or appearing too infrequently in the training set.

1. All properties and OPS components are within expected ranges.

Feature Contribution

Top features for positive contribution				
**Fingerprint**	**Bit/Smiles**	**Feature Structure**	**Score**	**Moderate_Severe in training set**
FCFP_10	-1695756380	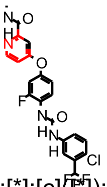 [*][c]1:[*]:[c]([*]):n:[cH]:[cH]:1	0.285	10 out of 11

FCFP_10	-124655670	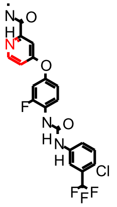 [*]:[cH]:[cH]:n:[*]	0.259	14 out of 16
FCFP_10	-885550502	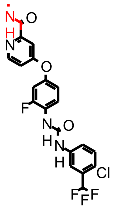 [*]C(=[*])NC	0.239	54 out of 64

Top Features for negative contribution

Fingerprint	Bit/Smiles	Feature Structure	Score	Moderate_Severe in training set
FCFP_10	2104062943	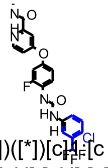 [*]C([*])([*])[c]1:[c H]:[*]:[cH]:[cH]:[c] :1Cl	-0.745	7 out of 24
FCFP_10	-174293376	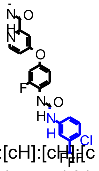 [*]N[c]1:[cH]:[cH]:[c](Cl):[c](:[cH]:1)C([*])([*])[*]	-0.507	0 out of 1
FCFP_10	-1549103449	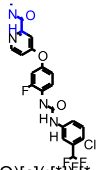 [*]NC(=O)[c](:[*]):[*]	-0.504	2 out of 6

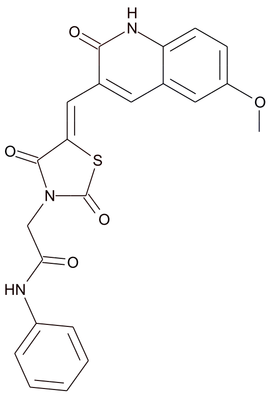

$C_{22}H_{17}N_3O_5S$

Molecular Weight: 435.45248

ALogP: 2.15

Rotatable Bonds: 5

Acceptors: 6

Donors: 2

Structural Similar Compounds

Name	ANTHRAQUINONE; 1-AMINO-4-HYDROXY-2-PHENOXY-	5-NORBORNENE-2;3-DICARBOXYLIC ACID; 1;4;5;6;7;7-HEXACHLORO-	1-AMINO-4-BENZOYLAMINO-ANTHRAQUINONE
Structure	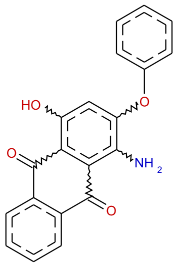	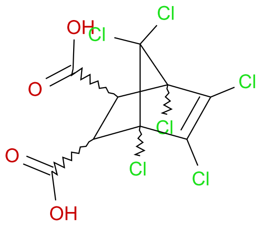	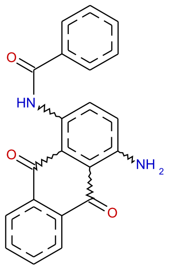
Actual Endpoint	Irritant	Irritant	Irritant
Predicted Endpoint	Irritant	Irritant	Irritant
Distance	0.701	0.717	0.719
Reference	28ZPAK 239;72	28ZPAK-;92;72	28ZPAK-;124;72

Model Prediction

Prediction: Irritant

Probability: 1

Enrichment: 1.18

Bayesian Score: 1.35

Mahalanobis Distance: 11.5

Mahalanobis Distance p-value: 0.000675

Prediction: Positive if the Bayesian score is above the estimated best cutoff value from minimizing the false positive and false negative rate.
Probability: The esimated probability that the sample is in the positive category. This assumes that the Bayesian score follows a normal distribution and is different from the prediction using a cutoff.
Enrichment: An estimate of enrichment, that is, the increased likelihood (versus random) of this sample being in the category.
Bayesian Score: The standard Laplacian-modified Bayesian score.
Mahalanobis Distance: The Mahalanobis distance (MD) is the distance to the center of the training data. The larger the MD, the less trustworthy the prediction.
Mahalanobis Distance p-value: The p-value gives the fraction of training data with an MD greater than or equal to the one for the given sample, assuming normally distributed data. The smaller the p-value, the less trustworthy the prediciton. For highly non-normal X properties (e.g., fingerprints), the MD p-value is wildly inaccurate.

Model Applicability

Unknown features are fingerprint features in the query molecule, but not found or appearing too infrequently in the training set.

1. All properties and OPS components are within expected ranges.

Feature Contribution

Top features for positive contribution

Fingerprint	Bit/Smiles	Feature Structure	Score	Irritant in training set
FCFP_12	1175665944	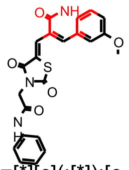 [*]C1=[*][c](:[*]):[c](NC1=O):[cH]:[*]	0.198	14 out of 14

FCFP_12	436915834	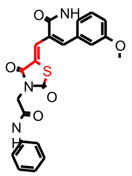 [*]\C=C\1/S[*][*]C1=[*]	0.167	4 out of 4
FCFP_12	2036120522	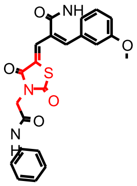 [*]CN1C(=O)SC(=[*])C1=[*]	0.167	4 out of 4

Top Features for negative contribution

Fingerprint	Bit/Smiles	Feature Structure	Score	Irritant in training set
FCFP_12	-1060187936	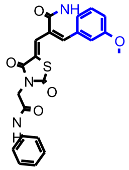 [*]N[c]1:[cH]:[cH]:[c](OC):[cH]:[c]:1[*]	-0.344	2 out of 4
FCFP_12	-1757681964	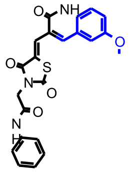 [*][c]1:[cH]:[cH]:[c](OC):[cH]:[c]:1C=[*]	-0.268	1 out of 2
FCFP_12	451371068	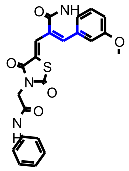 [*]C(=C[c](:[*]):[*])[*]	-0.167	6 out of 9

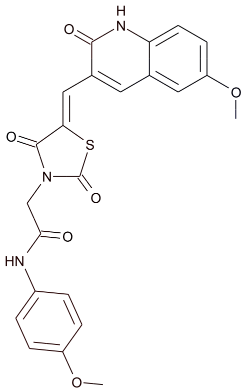

$C_{23}H_{19}N_3O_6S$
Molecular Weight: 465.47846
ALogP: 2.134
Rotatable Bonds: 6
Acceptors: 7
Donors: 2

Model Prediction

Prediction: Irritant

Probability: 1

Enrichment: 1.18

Bayesian Score: 1.67

Mahalanobis Distance: 11.4

Mahalanobis Distance p-value: 0.000792

Prediction: Positive if the Bayesian score is above the estimated best cutoff value from minimizing the false positive and false negative rate.
Probability: The esimated probability that the sample is in the positive category. This assumes that the Bayesian score follows a normal distribution and is different from the prediction using a cutoff.
Enrichment: An estimate of enrichment, that is, the increased likelihood (versus random) of this sample being in the category.
Bayesian Score: The standard Laplacian-modified Bayesian score.
Mahalanobis Distance: The Mahalanobis distance (MD) is the distance to the center of the training data. The larger the MD, the less trustworthy the prediction.
Mahalanobis Distance p-value: The p-value gives the fraction of training data with an MD greater than or equal to the one for the given sample, assuming normally distributed data. The smaller the p-value, the less trustworthy the prediciton. For highly non-normal X properties (e.g., fingerprints), the MD p-value is wildly inaccurate.

Structural Similar Compounds

Name	COLCHICINE	4;4'-DIAMINO-1;1'-DIANTHRIMIDE	ANTHRAQUINONE; 1-AMINO-4-HYDROXY-2-PHENOXY-
Structure	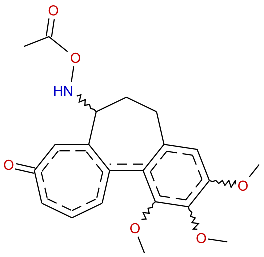	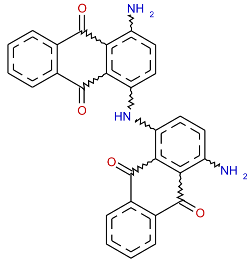	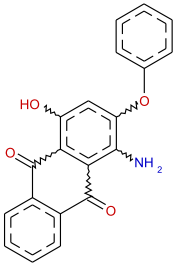
Actual Endpoint	Irritant	Irritant	Irritant
Predicted Endpoint	Irritant	Irritant	Irritant
Distance	0.761	0.764	0.793
Reference	AJOPAA 31;837;48	28ZPAK-;125;72	28ZPAK 239;72

Model Applicability

Unknown features are fingerprint features in the query molecule, but not found or appearing too infrequently in the training set.

1. All properties and OPS components are within expected ranges.

Feature Contribution

Top features for positive contribution

Fingerprint	Bit/Smiles	Feature Structure	Score	Irritant in training set
FCFP_12	1175665944	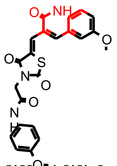 [*]C1=[*][c](:[*]):[c](NC1=O):[cH]:[*]	0.198	14 out of 14

FCFP_12	-789307649	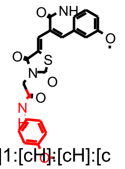 [*]O[c]1:[cH]:[cH]:[c](NC(=[*])[*]):[cH]:[cH]:1	0.167	4 out of 4
FCFP_12	436915834	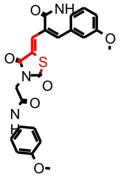 [*]\C=C\1/S[*][*]C1=[*]	0.167	4 out of 4

Top Features for negative contribution

Fingerprint	Bit/Smiles	Feature Structure	Score	Irritant in training set
FCFP_12	-1060187936	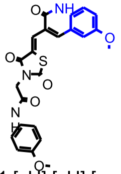 [*]N[c]1:[cH]:[cH]:[c](OC):[cH]:[c]:1[*]	-0.344	2 out of 4
FCFP_12	-1757681964	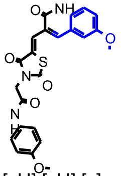 [*][c]1:[cH]:[cH]:[c](OC):[cH]:[c]:1C=[*]	-0.268	1 out of 2
FCFP_12	451371068	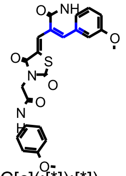 [*]C(=C[c](:[*]):[*])[*]	-0.167	6 out of 9

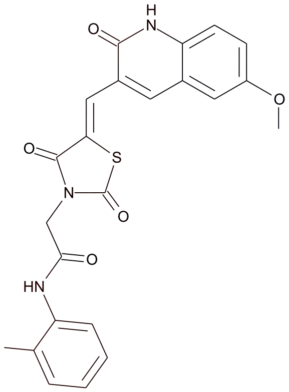

$C_{23}H_{19}N_3O_5S$
Molecular Weight: 449.47906
ALogP: 2.637
Rotatable Bonds: 5
Acceptors: 6
Donors: 2

Model Prediction

Prediction: Irritant
Probability: 1
Enrichment: 1.18
Bayesian Score: 1.64
Mahalanobis Distance: 11.5
Mahalanobis Distance p-value: 0.000646

Prediction: Positive if the Bayesian score is above the estimated best cutoff value from minimizing the false positive and false negative rate.
Probability: The esimated probability that the sample is in the positive category. This assumes that the Bayesian score follows a normal distribution and is different from the prediction using a cutoff.
Enrichment: An estimate of enrichment, that is, the increased likelihood (versus random) of this sample being in the category.
Bayesian Score: The standard Laplacian-modified Bayesian score.
Mahalanobis Distance: The Mahalanobis distance (MD) is the distance to the center of the training data. The larger the MD, the less trustworthy the prediction.
Mahalanobis Distance p-value: The p-value gives the fraction of training data with an MD greater than or equal to the one for the given sample, assuming normally distributed data. The smaller the p-value, the less trustworthy the prediciton. For highly non-normal X properties (e.g., fingerprints), the MD p-value is wildly inaccurate.

Structural Similar Compounds

Name	ANTHRAQUINONE; 1-AMINO-4-HYDROXY-2-PHENOXY-	5-NORBORNENE-2;3-DICARBOXYLIC ACID; 1;4;5;6;7;7-HEXACHLORO-	1-AMINO-4-BENZOYLAMINO-ANTHRAQUINONE
Structure	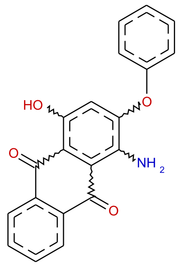	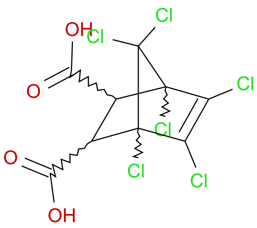	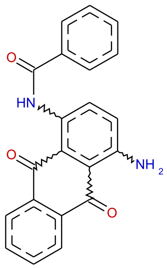
Actual Endpoint	Irritant	Irritant	Irritant
Predicted Endpoint	Irritant	Irritant	Irritant
Distance	0.698	0.717	0.721
Reference	28ZPAK 239;72	28ZPAK-;92;72	28ZPAK-;124;72

Model Applicability

Unknown features are fingerprint features in the query molecule, but not found or appearing too infrequently in the training set.

1. All properties and OPS components are within expected ranges.

Feature Contribution

Top features for positive contribution				
Fingerprint	Bit/Smiles	Feature Structure	Score	Irritant in training set
FCFP_12	1175665944	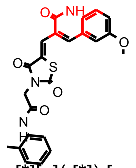 [*]C1=[*][c](:[*]):[c](NC1=O):[cH]:[*]	0.198	14 out of 14

FCFP_12	755520106	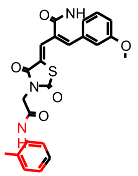<br>[*]N[c]1:[cH]:[*]:[cH<br>]:[cH]:[c]:1C	0.192	10 out of 10
FCFP_12	1396506317	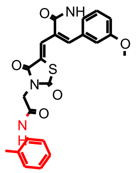<br>[*]N[c]1:[cH]:[cH]:[c<br>H]:[cH]:[c]:1C	0.167	4 out of 4
**Top Features for negative contribution**				
**Fingerprint**	**Bit/Smiles**	**Feature Structure**	**Score**	**Irritant in training set**
FCFP_12	-1060187936	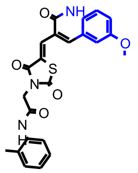<br>[*]N[c]1:[cH]:[cH]:[c<br>](OC):[cH]:[c]:1[*]	-0.344	2 out of 4
FCFP_12	-1757681964	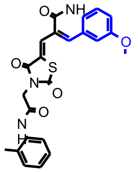<br>[*][c]1:[cH]:[cH]:[c]<br>(OC):[cH]:[c]:1C=[*]	-0.268	1 out of 2
FCFP_12	451371068	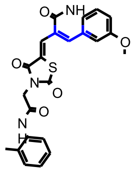<br>[*]C(=C[c](:[*]):[*])<br>[*]	-0.167	6 out of 9

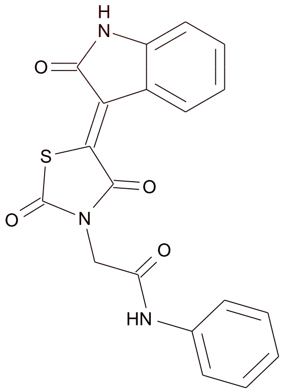

$C_{19}H_{13}N_3O_4S$
Molecular Weight: 379.38922
ALogP: 1.699
Rotatable Bonds: 3
Acceptors: 5
Donors: 2

Structural Similar Compounds

Name	1-AMINO-4-BENZOYLAMINO-ANTHRAQUINONE	Anthraquinone; 1-amino-2-bromo-4-hydroxy-	ANTHRAQUINONE; 1-AMINO-4-HYDROXY-2-PHENOXY-
Structure	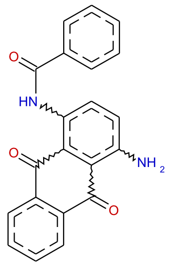	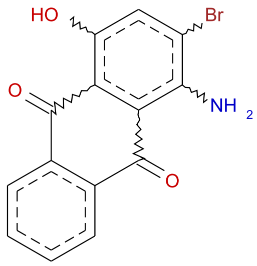	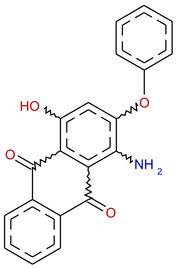
Actual Endpoint	Irritant	Non-Irritant	Irritant
Predicted Endpoint	Irritant	Non-Irritant	Irritant
Distance	0.621	0.629	0.645
Reference	28ZPAK-;124;72	Prehled Prumyslove Toxikologie; Organicke Latky; Marhold; J. pp 535;86	28ZPAK 239;72

Model Prediction

Prediction: Irritant
Probability: 1
Enrichment: 1.18
Bayesian Score: 1.57
Mahalanobis Distance: 8.24
Mahalanobis Distance p-value: 0.863

Prediction: Positive if the Bayesian score is above the estimated best cutoff value from minimizing the false positive and false negative rate.
Probability: The esimated probability that the sample is in the positive category. This assumes that the Bayesian score follows a normal distribution and is different from the prediction using a cutoff.
Enrichment: An estimate of enrichment, that is, the increased likelihood (versus random) of this sample being in the category.
Bayesian Score: The standard Laplacian-modified Bayesian score.
Mahalanobis Distance: The Mahalanobis distance (MD) is the distance to the center of the training data. The larger the MD, the less trustworthy the prediction.
Mahalanobis Distance p-value: The p-value gives the fraction of training data with an MD greater than or equal to the one for the given sample, assuming normally distributed data. The smaller the p-value, the less trustworthy the prediciton. For highly non-normal X properties (e.g., fingerprints), the MD p-value is wildly inaccurate.

Model Applicability

Unknown features are fingerprint features in the query molecule, but not found or appearing too infrequently in the training set.

1. All properties and OPS components are within expected ranges.

Feature Contribution

Top features for positive contribution

Fingerprint	Bit/Smiles	Feature Structure	Score	Irritant in training set
FCFP_12	1175665944	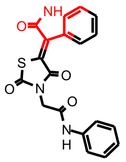 [*]C1=[*][c](:[*]):[c](NC1=O):[cH]:[*]	0.198	14 out of 14

FCFP_12	2036120522	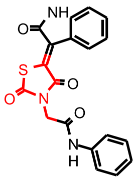 [*]CN1C(=O)SC(=[*])C1 =[*]	0.167	4 out of 4
FCFP_12	436915834	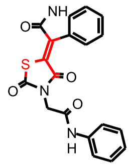 [*]\C=C\1/S[*][*]C1=[*]	0.167	4 out of 4

Top Features for negative contribution

**Fingerprint**	**Bit/Smiles**	**Feature Structure**	**Score**	**Irritant in training set**
FCFP_12	-1698724694	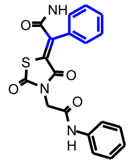 [*][c]1:[cH]:[cH]:[cH]:[cH]:[c]:1C	-0.0964	107 out of 146
FCFP_12	565998553	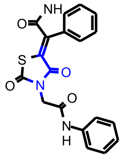 [*]N1[*][*]C(=[*])C1= O	-0.0662	198 out of 262
FCFP_12	-1678275541	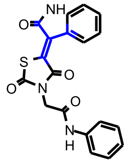 [*]C(=C1C(=[*])[*][*] :[c]1:[*])[*]	-0.0561	3 out of 4

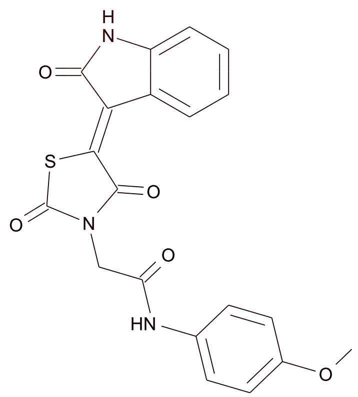

$C_{20}H_{15}N_3O_5S$
Molecular Weight: 409.4152
ALogP: 1.683
Rotatable Bonds: 4
Acceptors: 6
Donors: 2

Structural Similar Compounds

Name	ANTHRAQUINONE; 1-AMINO-4-HYDROXY-2-PHENOXY-	1-AMINO-4-BENZOYLAMINO-ANTHRAQUINONE	2-NAPHTHALENESULFONIC ACID;5-AMINO-6-ETHOXY-
Structure	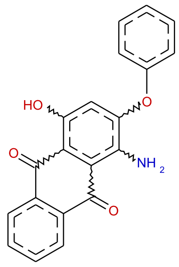	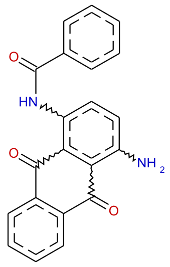	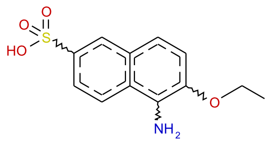
Actual Endpoint	Irritant	Irritant	Irritant
Predicted Endpoint	Irritant	Irritant	Irritant
Distance	0.690	0.705	0.707
Reference	28ZPAK 239;72	28ZPAK-;124;72	28ZPAK-;191;72

Model Prediction

Prediction: Irritant
Probability: 1
Enrichment: 1.18
Bayesian Score: 1.93
Mahalanobis Distance: 8.88
Mahalanobis Distance p-value: 0.579

Prediction: Positive if the Bayesian score is above the estimated best cutoff value from minimizing the false positive and false negative rate.
Probability: The esimated probability that the sample is in the positive category. This assumes that the Bayesian score follows a normal distribution and is different from the prediction using a cutoff.
Enrichment: An estimate of enrichment, that is, the increased likelihood (versus random) of this sample being in the category.
Bayesian Score: The standard Laplacian-modified Bayesian score.
Mahalanobis Distance: The Mahalanobis distance (MD) is the distance to the center of the training data. The larger the MD, the less trustworthy the prediction.
Mahalanobis Distance p-value: The p-value gives the fraction of training data with an MD greater than or equal to the one for the given sample, assuming normally distributed data. The smaller the p-value, the less trustworthy the prediciton. For highly non-normal X properties (e.g., fingerprints), the MD p-value is wildly inaccurate.

Model Applicability

Unknown features are fingerprint features in the query molecule, but not found or appearing too infrequently in the training set.

1. All properties and OPS components are within expected ranges.

Feature Contribution

Top features for positive contribution				
Fingerprint	Bit/Smiles	Feature Structure	Score	Irritant in training set
FCFP_12	1175665944	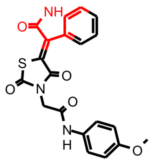 [*]C1=[*][c](:[*]):[c](NC1=O):[cH]:[*]	0.198	14 out of 14

FCFP_12	-568981285	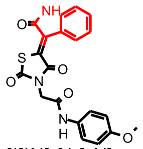 [*]C(=[*])N[c]1:[cH]:[cH]:[cH]:[cH]:[c]:1C	0.167	4 out of 4
FCFP_12	-789307649	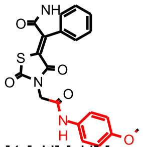 [*]O[c]1:[cH]:[cH]:[c](NC(=[*])[*]):[cH]:[cH]:1	0.167	4 out of 4
**Top Features for negative contribution**				
**Fingerprint**	**Bit/Smiles**	**Feature Structure**	**Score**	**Irritant in training set**
FCFP_12	-1060187936	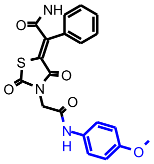 [*]N[c]1:[cH]:[cH]:[c](OC):[cH]:[c]:1[*]	-0.344	2 out of 4
FCFP_12	-1698724694	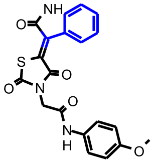 [*][c]1:[cH]:[cH]:[cH]:[cH]:[c]:1C	-0.0964	107 out of 146
FCFP_12	565998553	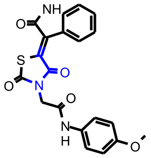 [*]N1[*][*]C(=[*])C1=O	-0.0662	198 out of 262

Sorafenib

TOPKAT_Ocular_Irritancy_None_vs_Irritant

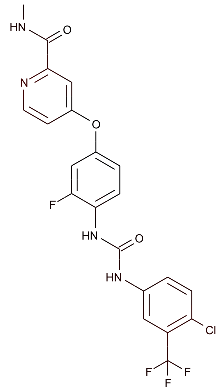

$C_{21}H_{15}ClF_4N_4O_3$
Molecular Weight: 482.81541
ALogP: 4.381
Rotatable Bonds: 6
Acceptors: 4
Donors: 3

Model Prediction

Prediction: Irritant
Probability: 1
Enrichment: 1.18
Bayesian Score: 3.33
Mahalanobis Distance: 6.51
Mahalanobis Distance p-value: 1

Prediction: Positive if the Bayesian score is above the estimated best cutoff value from minimizing the false positive and false negative rate.
Probability: The esimated probability that the sample is in the positive category. This assumes that the Bayesian score follows a normal distribution and is different from the prediction using a cutoff.
Enrichment: An estimate of enrichment, that is, the increased likelihood (versus random) of this sample being in the category.
Bayesian Score: The standard Laplacian-modified Bayesian score.
Mahalanobis Distance: The Mahalanobis distance (MD) is the distance to the center of the training data. The larger the MD, the less trustworthy the prediction.
Mahalanobis Distance p-value: The p-value gives the fraction of training data with an MD greater than or equal to the one for the given sample, assuming normally distributed data. The smaller the p-value, the less trustworthy the prediciton. For highly non-normal X properties (e.g., fingerprints), the MD p-value is wildly inaccurate.

Structural Similar Compounds

Name	BENZANILIDE;2';2'''-DITHIOBIS-	4;4'-DIAMINO-1;1'-DIANTHRIMIDE	ANTHRAQUINONE; 1;4-BIS(p-TOLYLAMINO)-
Structure	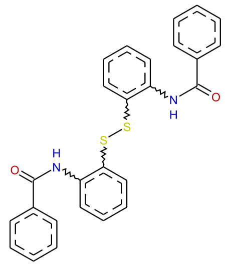	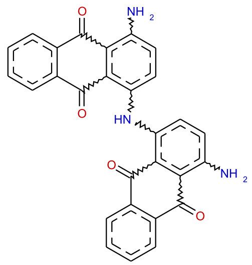	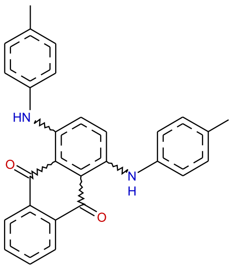
Actual Endpoint	Non-Irritant	Irritant	Irritant
Predicted Endpoint	Non-Irritant	Irritant	Non-Irritant
Distance	0.739	0.800	0.820
Reference	28ZPAK-;173;72	28ZPAK-;125;72	28ZPAK -;124;72

Model Applicability

Unknown features are fingerprint features in the query molecule, but not found or appearing too infrequently in the training set.

1. All properties and OPS components are within expected ranges.

Feature Contribution

Top features for positive contribution

Fingerprint	Bit/Smiles	Feature Structure	Score	Irritant in training set
FCFP_12	1747237384	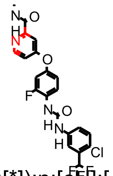 [*][c](:[*]):n:[cH]:[*]	0.208	44 out of 44

FCFP_12	-124655670	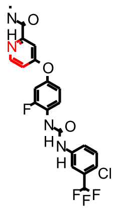 [*]:[cH]:[cH]:n:[*]	0.2	16 out of 16
FCFP_12	-1539132615	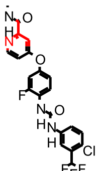 [*]C(=[*])[c](:[cH]:[*]):n:[*]	0.197	13 out of 13

Top Features for negative contribution

Fingerprint	Bit/Smiles	Feature Structure	Score	Irritant in training set
FCFP_12	-747629521	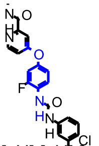 [*]N[c]1:[cH]:[cH]:[c](O[c](:[*]):[*]):[c H]:[c]:1[*]	-0.268	1 out of 2
FCFP_12	1035613116	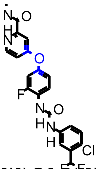 [*]:[c](:[*])O[c](:[*]):[*]	0	9 out of 11
FCFP_12	1	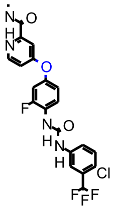 [*]=O	0	872 out of 1051

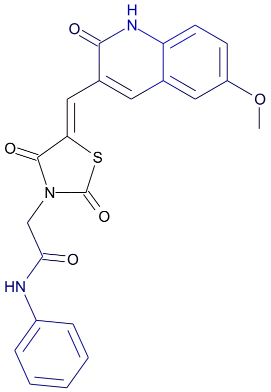

$C_{22}H_{17}N_3O_5S$

Molecular Weight: 435.45248

ALogP: 2.15

Rotatable Bonds: 5

Acceptors: 6

Donors: 2

Model Prediction

Prediction: Non-Irritant

Probability: 0.0103

Enrichment: 0.0112

Bayesian Score: -7.05

Mahalanobis Distance: 13.1

Mahalanobis Distance p-value: 4.55e-008

Prediction: Positive if the Bayesian score is above the estimated best cutoff value from minimizing the false positive and false negative rate.
Probability: The esimated probability that the sample is in the positive category. This assumes that the Bayesian score follows a normal distribution and is different from the prediction using a cutoff.
Enrichment: An estimate of enrichment, that is, the increased likelihood (versus random) of this sample being in the category.
Bayesian Score: The standard Laplacian-modified Bayesian score.
Mahalanobis Distance: The Mahalanobis distance (MD) is the distance to the center of the training data. The larger the MD, the less trustworthy the prediction.
Mahalanobis Distance p-value: The p-value gives the fraction of training data with an MD greater than or equal to the one for the given sample, assuming normally distributed data. The smaller the p-value, the less trustworthy the prediciton. For highly non-normal X properties (e.g., fingerprints), the MD p-value is wildly inaccurate.

Structural Similar Compounds

Name	2-Anthracenesulfonic acid, 1-amino-9,10-dihydro-9,10-dioxo-4-(2,4,6 -trimethylanilino)-, monosodium salt	Pregna-1,4-diene-3,20-dione, 21-(acetyloxy)-11-hydroxy-6-methyl-17- (1-oxopropoxy)-, (6-alpha,11-beta)-	Benzenesulfonamide, 4-amino-N-(5,6-dimethoxy-4-pyrimidinyl)-
Structure	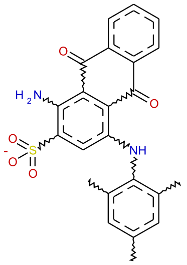	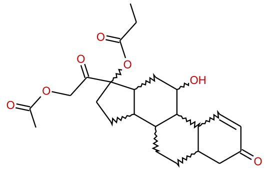	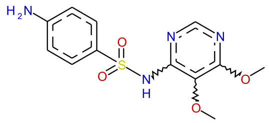
Actual Endpoint	Irritant	Irritant	Irritant
Predicted Endpoint	Non-Irritant	Irritant	Non-Irritant
Distance	0.613	0.762	0.779
Reference	85JCAE "Prehled Prumyslove Toxikologie; Organicke Latky," Marhold, J., Prague , Czechoslovakia, Avicenum, 1986 Volume(issue)/page/year: -,1327,1986	YACHDS Yakuri to Chiryo. Pharmacology and Therapeutics. (Raifu Saiensu Shup pan K.K., 2-5-13, Yaesu, Chuo-ku, Tokyo 104, Japan) V.1-1972- Volume(issue) /page/year: 19,3103,1991	FCTXAV 14,307,76

Model Applicability

Unknown features are fingerprint features in the query molecule, but not found or appearing too infrequently in the training set.

1. All properties and OPS components are within expected ranges.

Feature Contribution

Top features for positive contribution				
**Fingerprint**	**Bit/Smiles**	**Feature Structure**	**Score**	**Irritant in training set**

FCFP_12	-1986158408	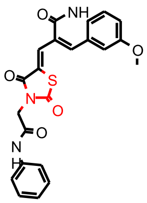 [*]N1[*][*]SC1=O	0.0821	13 out of 13
FCFP_12	436886043	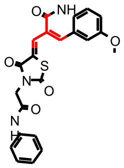 [*]\C=C(\C=[*])/C(=[*])[*]	0.0804	129 out of 130
FCFP_12	1383817444	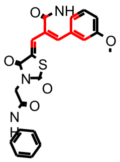 [*]=CC1=C[c](:[cH]:[*]):[c](:[*])[*]C1=[*]	0.0772	7 out of 7

Top Features for negative contribution

**Fingerprint**	**Bit/Smiles**	**Feature Structure**	**Score**	**Irritant in training set**
FCFP_12	1175665944	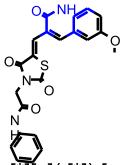 [*]C1=[*][c](:[*]):[c](NC1=O):[cH]:[*]	-1.02	2 out of 8
FCFP_12	-1838187238	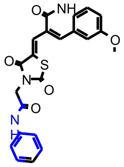 [*]C(=[*])N[c]1:[cH]:[cH]:[*]:[cH]:[cH]:1	-0.692	5 out of 12

FCFP_12	-792685140	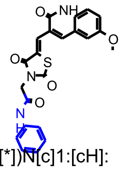 [*]C(=[*])N[c]1:[cH]:[cH]:[cH]:[cH]:[cH]:1	-0.65	0 out of 1

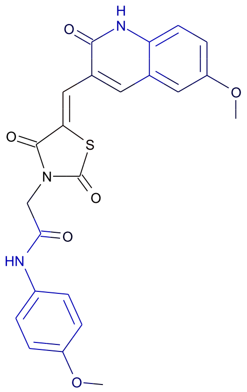

$C_{23}H_{19}N_3O_6S$
Molecular Weight: 465.47846
ALogP: 2.134
Rotatable Bonds: 6
Acceptors: 7
Donors: 2

Model Prediction

Prediction: Non-Irritant
Probability: 0.00107
Enrichment: 0.00116
Bayesian Score: -8.04
Mahalanobis Distance: 13
Mahalanobis Distance p-value: 6.4e-008

Prediction: Positive if the Bayesian score is above the estimated best cutoff value from minimizing the false positive and false negative rate.
Probability: The esimated probability that the sample is in the positive category. This assumes that the Bayesian score follows a normal distribution and is different from the prediction using a cutoff.
Enrichment: An estimate of enrichment, that is, the increased likelihood (versus random) of this sample being in the category.
Bayesian Score: The standard Laplacian-modified Bayesian score.
Mahalanobis Distance: The Mahalanobis distance (MD) is the distance to the center of the training data. The larger the MD, the less trustworthy the prediction.
Mahalanobis Distance p-value: The p-value gives the fraction of training data with an MD greater than or equal to the one for the given sample, assuming normally distributed data. The smaller the p-value, the less trustworthy the prediciton. For highly non-normal X properties (e.g., fingerprints), the MD p-value is wildly inaccurate.

Structural Similar Compounds

Name	2-Anthracenesulfonic acid, 1-amino-9,10-dihydro-9,10-dioxo-4-(2,4,6 -trimethylanilino)-, monosodium salt	Pregna-1,4-diene-3,20-dione, 21-(acetyloxy)-11-hydroxy-6-methyl-17- (1-oxopropoxy)-, (6-alpha,11-beta)-	Benzenesulfonamide, 4-amino-N-(5,6-dimethoxy-4-pyrimidinyl)-
Structure	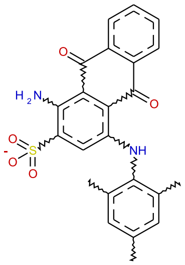	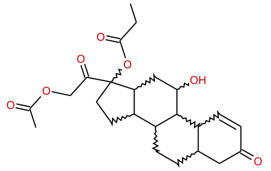	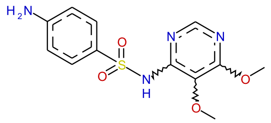
Actual Endpoint	Irritant	Irritant	Irritant
Predicted Endpoint	Non-Irritant	Irritant	Non-Irritant
Distance	0.626	0.767	0.831
Reference	85JCAE "Prehled Prumyslove Toxikologie; Organicke Latky," Marhold, J., Prague , Czechoslovakia, Avicenum, 1986 Volume(issue)/page/year: -,1327,1986	YACHDS Yakuri to Chiryo. Pharmacology and Therapeutics. (Raifu Saiensu Shup pan K.K., 2-5-13, Yaesu, Chuo-ku, Tokyo 104, Japan) V.1-1972- Volume(issue) /page/year: 19,3103,1991	FCTXAV 14,307,76

Model Applicability

Unknown features are fingerprint features in the query molecule, but not found or appearing too infrequently in the training set.

1. All properties and OPS components are within expected ranges.

Feature Contribution

Top features for positive contribution				
Fingerprint	Bit/Smiles	Feature Structure	Score	Irritant in training set

FCFP_12	-1986158408	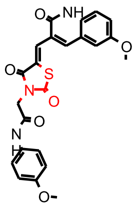 [*]N1[*][*]SC1=O	0.0821	13 out of 13
FCFP_12	436886043	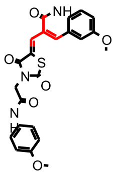 [*]\C=C(\C=[*])/C(=[*])[*]	0.0804	129 out of 130
FCFP_12	1383817444	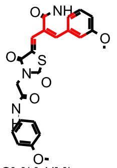 [*]=CC1=C[c](:[cH]:[*]):[c](:[*])[*]C1=[*]	0.0772	7 out of 7

Top Features for negative contribution

Fingerprint	Bit/Smiles	Feature Structure	Score	Irritant in training set
FCFP_12	-789307649	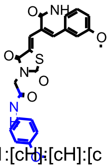 [*]O[c]1:[cH]:[cH]:[c](NC(=[*])[*]):[cH]: [cH]:1	-1.54	0 out of 4
FCFP_12	1175665944	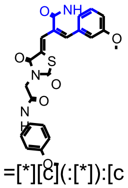 [*]C1=[*][c](:[*]):[c](NC1=O):[cH]:[*]	-1.02	2 out of 8

FCFP_12	-1838187238	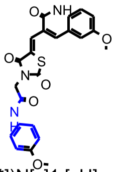 [*]C(=[*])N[c]1:[cH]:[cH]:[*]:[cH]:[cH]:1	-0.692	5 out of 12

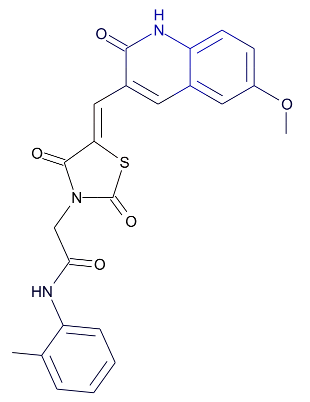

$C_{23}H_{19}N_3O_5S$
Molecular Weight: 449.47906
ALogP: 2.637
Rotatable Bonds: 5
Acceptors: 6
Donors: 2

Structural Similar Compounds

Name	2-Anthracenesulfonic acid, 1-amino-9,10-dihydro-9,10-dioxo-4-(2,4,6 -trimethylanilino)-, monosodium salt	Pregna-1,4-diene-3,20-dione, 21-(acetyloxy)-11-hydroxy-6-methyl-17- (1-oxopropoxy)-, (6-alpha,11-beta)-	Benzenesulfonic acid, 2,2'-(4,4'-biphenylylenedivinylene)di-, disod ium salt
Structure	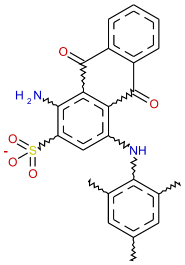	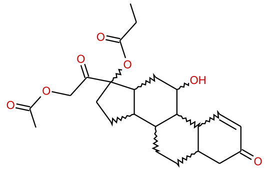	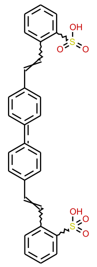
Actual Endpoint	Irritant	Irritant	Irritant
Predicted Endpoint	Non-Irritant	Irritant	Non-Irritant
Distance	0.615	0.765	0.820
Reference	85JCAE "Prehled Prumyslove Toxikologie; Organicke Latky," Marhold, J., Prague , Czechoslovakia, Avicenum, 1986 Volume(issue)/page/year: -,1327,1986	YACHDS Yakuri to Chiryo. Pharmacology and Therapeutics. (Raifu Saiensu Shup pan K.K., 2-5-13, Yaesu, Chuo-ku, Tokyo 104, Japan) V.1- 1972- Volume(issue) /page/year: 19,3103,1991	MVCRB3 MVC-Report. (Stockholm, Sweden) No.1-2, 1972-73. Discontinued. Volu me(issue)/page/year: 2,193,1973

Model Prediction

Prediction: Non-Irritant

Probability: 0.161

Enrichment: 0.174

Bayesian Score: -5.57

Mahalanobis Distance: 13.1

Mahalanobis Distance p-value: 3.79e-008

Prediction: Positive if the Bayesian score is above the estimated best cutoff value from minimizing the false positive and false negative rate.
Probability: The esimated probability that the sample is in the positive category. This assumes that the Bayesian score follows a normal distribution and is different from the prediction using a cutoff.
Enrichment: An estimate of enrichment, that is, the increased likelihood (versus random) of this sample being in the category.
Bayesian Score: The standard Laplacian-modified Bayesian score.
Mahalanobis Distance: The Mahalanobis distance (MD) is the distance to the center of the training data. The larger the MD, the less trustworthy the prediction.
Mahalanobis Distance p-value: The p-value gives the fraction of training data with an MD greater than or equal to the one for the given sample, assuming normally distributed data. The smaller the p-value, the less trustworthy the prediciton. For highly non-normal X properties (e.g., fingerprints), the MD p-value is wildly inaccurate.

Model Applicability

Unknown features are fingerprint features in the query molecule, but not found or appearing too infrequently in the training set.

1. All properties and OPS components are within expected ranges.

Feature Contribution

Top features for positive contribution				
**Fingerprint**	**Bit/Smiles**	**Feature Structure**	**Score**	**Irritant in training set**

FCFP_12	-1986158408	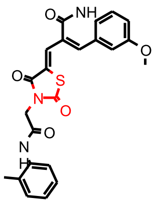<br>[*]N1[*][*]SC1=O	0.0821	13 out of 13
FCFP_12	436886043	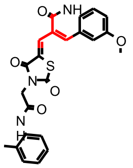<br>[*]\C=C(\C=[*])/C(=[*])[*]	0.0804	129 out of 130
FCFP_12	1383817444	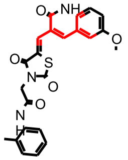<br>[*]=CC1=C[c](:[cH]:[*]):[c](:[*])[*]C1=[*]	0.0772	7 out of 7

Top Features for negative contribution

Fingerprint	Bit/Smiles	Feature Structure	Score	Irritant in training set
FCFP_12	1175665944	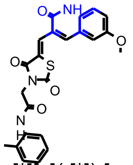<br>[*]C1=[*][c](:[*]):[c](NC1=O):[cH]:[*]	-1.02	2 out of 8
FCFP_12	-1757681964	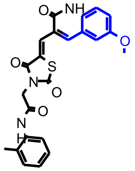<br>[*][c]1:[cH]:[cH]:[c](OC):[cH]:[c]:1C=[*]	-0.627	1 out of 3

FCFP_12	1294255210	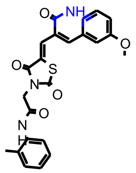 [*]C(=[*])N[c](:[*]):[*]	-0.486	12 out of 22

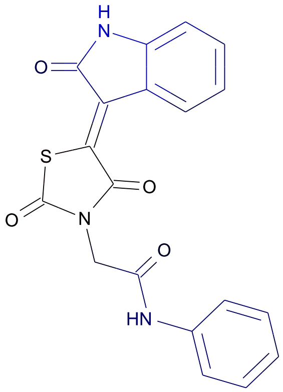

$C_{19}H_{13}N_3O_4S$
Molecular Weight: 379.38922
ALogP: 1.699
Rotatable Bonds: 3
Acceptors: 5
Donors: 2

Model Prediction

Prediction: Non-Irritant
Probability: 0.028
Enrichment: 0.0304
Bayesian Score: -6.57
Mahalanobis Distance: 11.1
Mahalanobis Distance p-value: 0.00155

Prediction: Positive if the Bayesian score is above the estimated best cutoff value from minimizing the false positive and false negative rate.
Probability: The esimated probability that the sample is in the positive category. This assumes that the Bayesian score follows a normal distribution and is different from the prediction using a cutoff.
Enrichment: An estimate of enrichment, that is, the increased likelihood (versus random) of this sample being in the category.
Bayesian Score: The standard Laplacian-modified Bayesian score.
Mahalanobis Distance: The Mahalanobis distance (MD) is the distance to the center of the training data. The larger the MD, the less trustworthy the prediction.
Mahalanobis Distance p-value: The p-value gives the fraction of training data with an MD greater than or equal to the one for the given sample, assuming normally distributed data. The smaller the p-value, the less trustworthy the prediciton. For highly non-normal X properties (e.g., fingerprints), the MD p-value is wildly inaccurate.

Structural Similar Compounds

Name	Benzenesulfonic acid, 2-anilino-5-nitro-	2-Anthracenesulfonic acid, 1-amino-9,10-dihydro-9,10-dioxo-4-(2,4,6 -trimethylanilino)-, monosodium salt	Benzenesulfonamide, 4-amino-N-(5,6-dimethoxy-4-pyrimidinyl)-
Structure	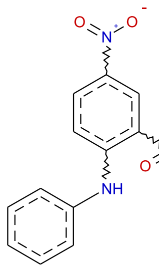	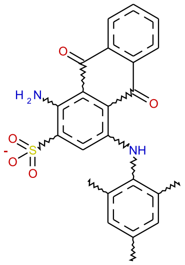	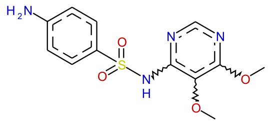
Actual Endpoint	Irritant	Irritant	Irritant
Predicted Endpoint	Non-Irritant	Non-Irritant	Non-Irritant
Distance	0.700	0.710	0.736
Reference	85JCAE "Prehled Prumyslove Toxikologie; Organicke Latky," Marhold, J., Prague , Czechoslovakia, Avicenum, 1986 Volume(issue)/page/year: -,1061,1986	85JCAE "Prehled Prumyslove Toxikologie; Organicke Latky," Marhold, J., Prague , Czechoslovakia, Avicenum, 1986 Volume(issue)/page/year: -,1327,1986	FCTXAV 14,307,76

Model Applicability

Unknown features are fingerprint features in the query molecule, but not found or appearing too infrequently in the training set.

1. All properties and OPS components are within expected ranges.

Feature Contribution

Top features for positive contribution				
Fingerprint	Bit/Smiles	Feature Structure	Score	Irritant in training set

FCFP_12	-1986158408	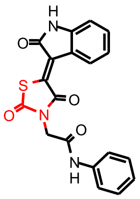 [*]N1[*][*]SC1=O	0.0821	13 out of 13
FCFP_12	436915834	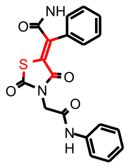 [*]\C=C\1/S[*][*]C1=[*]	0.0756	6 out of 6
FCFP_12	-1143715940	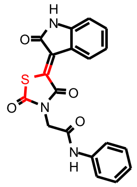 [*]=C1[*][*]C(=[*])S1	0.0575	475 out of 490
**Top Features for negative contribution**				
**Fingerprint**	**Bit/Smiles**	**Feature Structure**	**Score**	**Irritant in training set**
FCFP_12	1175665944	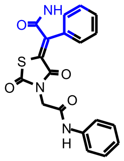 [*]C1=[*][c](:[*]):[c](NC1=O):[cH]:[*]	-1.02	2 out of 8
FCFP_12	-1838187238	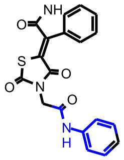 [*]C(=[*])N[c]1:[cH]:[cH]:[*]:[cH]:[cH]:1	-0.692	5 out of 12

FCFP_12	-451043714	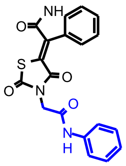 [*]CC(=O)N[c]1:[cH]:[cH]:[cH]:[cH]:[cH]:1	-0.65	0 out of 1

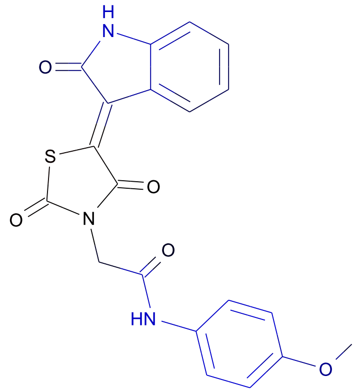

$C_{20}H_{15}N_3O_5S$
Molecular Weight: 409.4152
ALogP: 1.683
Rotatable Bonds: 4
Acceptors: 6
Donors: 2

Model Prediction

Prediction: Non-Irritant
Probability: 0.000249
Enrichment: 0.00027
Bayesian Score: -8.61
Mahalanobis Distance: 10.9
Mahalanobis Distance p-value: 0.00358

Prediction: Positive if the Bayesian score is above the estimated best cutoff value from minimizing the false positive and false negative rate.
Probability: The esimated probability that the sample is in the positive category. This assumes that the Bayesian score follows a normal distribution and is different from the prediction using a cutoff.
Enrichment: An estimate of enrichment, that is, the increased likelihood (versus random) of this sample being in the category.
Bayesian Score: The standard Laplacian-modified Bayesian score.
Mahalanobis Distance: The Mahalanobis distance (MD) is the distance to the center of the training data. The larger the MD, the less trustworthy the prediction.
Mahalanobis Distance p-value: The p-value gives the fraction of training data with an MD greater than or equal to the one for the given sample, assuming normally distributed data. The smaller the p-value, the less trustworthy the prediciton. For highly non-normal X properties (e.g., fingerprints), the MD p-value is wildly inaccurate.

Structural Similar Compounds

Name	2-Anthracenesulfonic acid, 1-amino-9,10-dihydro-9,10-dioxo-4-(2,4,6 -trimethylanilino)-, monosodium salt	Benzenesulfonamide, 4-amino-N-(5,6-dimethoxy-4-pyrimidinyl)-	Benzenesulfonic acid, 2-anilino-5-nitro-
Structure	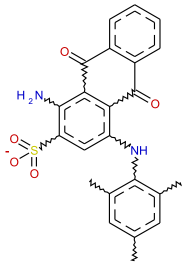	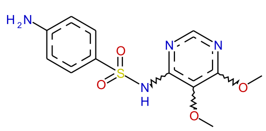	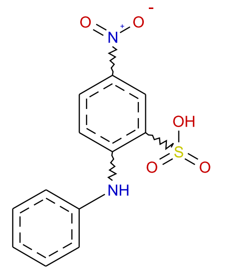
Actual Endpoint	Irritant	Irritant	Irritant
Predicted Endpoint	Non-Irritant	Non-Irritant	Non-Irritant
Distance	0.599	0.704	0.747
Reference	85JCAE "Prehled Prumyslove Toxikologie; Organicke Latky," Marhold, J., Prague , Czechoslovakia, Avicenum, 1986 Volume(issue)/page/year: -,1327,1986	FCTXAV 14,307,76	85JCAE "Prehled Prumyslove Toxikologie; Organicke Latky," Marhold, J., Prague , Czechoslovakia, Avicenum, 1986 Volume(issue)/page/year: -,1061,1986

Model Applicability

Unknown features are fingerprint features in the query molecule, but not found or appearing too infrequently in the training set.

1. All properties and OPS components are within expected ranges.

Feature Contribution

Top features for positive contribution

Fingerprint	Bit/Smiles	Feature Structure	Score	Irritant in training set

FCFP_12	-1986158408	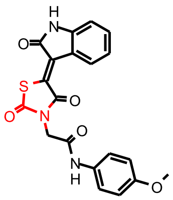 [*]N1[*][*]SC1=O	0.0821	13 out of 13
FCFP_12	436915834	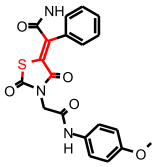 [*]\C=C\1/S[*][*]C1=[*]	0.0756	6 out of 6
FCFP_12	-1143715940	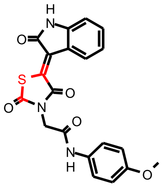 [*]=C1[*][*]C(=[*])S1	0.0575	475 out of 490
**Top Features for negative contribution**				
**Fingerprint**	**Bit/Smiles**	**Feature Structure**	**Score**	**Irritant in training set**
FCFP_12	-789307649	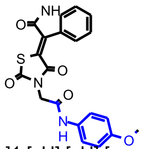 [*]O[c]1:[cH]:[cH]:[c](NC(=[*])[*]):[cH]:[cH]:1	-1.54	0 out of 4
FCFP_12	1175665944	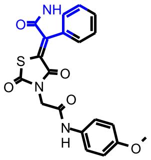 [*]C1=[*][c](:[*]):[c](NC1=O):[cH]:[*]	-1.02	2 out of 8

FCFP_12	-1838187238	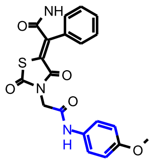<br>[*]C(=[*])N[c]1:[cH]:[cH]:[*]:[cH]:[cH]:1	-0.692	5 out of 12

Sorafenib

TOPKAT_Skin_Irritancy_None_vs_Irritant

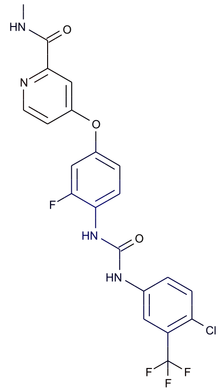

$C_{21}H_{15}ClF_4N_4O_3$
Molecular Weight: 482.81541
ALogP: 4.381
Rotatable Bonds: 6
Acceptors: 4
Donors: 3

Model Prediction

Prediction: Non-Irritant
Probability: 0.622
Enrichment: 0.676
Bayesian Score: -4.2
Mahalanobis Distance: 8.39
Mahalanobis Distance p-value: 0.742

Prediction: Positive if the Bayesian score is above the estimated best cutoff value from minimizing the false positive and false negative rate.
Probability: The esimated probability that the sample is in the positive category. This assumes that the Bayesian score follows a normal distribution and is different from the prediction using a cutoff.
Enrichment: An estimate of enrichment, that is, the increased likelihood (versus random) of this sample being in the category.
Bayesian Score: The standard Laplacian-modified Bayesian score.
Mahalanobis Distance: The Mahalanobis distance (MD) is the distance to the center of the training data. The larger the MD, the less trustworthy the prediction.
Mahalanobis Distance p-value: The p-value gives the fraction of training data with an MD greater than or equal to the one for the given sample, assuming normally distributed data. The smaller the p-value, the less trustworthy the prediciton. For highly non-normal X properties (e.g., fingerprints), the MD p-value is wildly inaccurate.

Structural Similar Compounds

Name	Benzenesulfonic acid, 2,2'-(4,4'-biphenylylenedivinylene)di-, disod ium salt	5-Norbornene-2,3-dicarboxylic acid, 1,4,5,6,7,7-hexachloro-	Sulfide, bis(4-t-butyl-m-cresyl)-
Structure	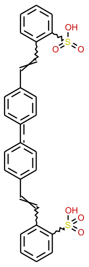	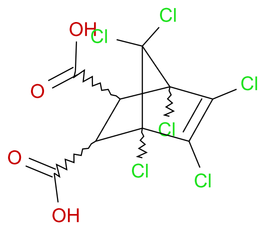	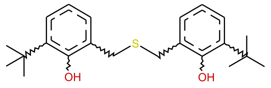
Actual Endpoint	Irritant	Irritant	Irritant
Predicted Endpoint	Non-Irritant	Irritant	Irritant
Distance	0.856	0.871	0.897
Reference	MVCRB3 MVC-Report. (Stockholm, Sweden) No.1-2, 1972-73. Discontinued. Volu me(issue)/page/year: 2,193,1973	85JCAE "Prehled Prumyslove Toxikologie; Organicke Latky," Marhold, J., Prague , Czechoslovakia, Avicenum, 1986 Volume(issue)/page/year: -,581,1986	AMIHBC AMA Archives of Industrial Hygiene and Occupational Medicine. (Chicago , IL) V.2-10, 1950-54. For publisher information, see AEHLAU. Volume(issue)/pag e/year: 5,311,1952

Model Applicability

Unknown features are fingerprint features in the query molecule, but not found or appearing too infrequently in the training set.

1. All properties and OPS components are within expected ranges.

Feature Contribution

Top features for positive contribution				
Fingerprint	Bit/Smiles	Feature Structure	Score	Irritant in training set

FCFP_12	-124655670	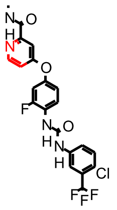 [*]:[cH]:[cH]:n:[*]	0.0821	13 out of 13
FCFP_12	-1539132615	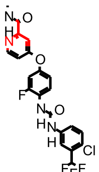 [*]C(=[*])[c](:[cH]:[*]):n:[*]	0.0795	9 out of 9
FCFP_12	-1695756380	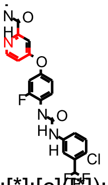 [*][c]1:[*]:[c]([*]): n:[cH]:[cH]:1	0.0772	7 out of 7
**Top Features for negative contribution**				
**Fingerprint**	**Bit/Smiles**	**Feature Structure**	**Score**	**Irritant in training set**
FCFP_12	-1838187238	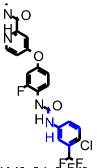 [*]C(=[*])N[c]1:[cH]: [cH]:[*]:[cH]:[cH]:1	-0.692	5 out of 12
FCFP_12	1783756416	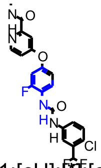 [*]N[c]1:[cH]:[*]:[c] ([*]):[cH]:[c]:1F	-0.509	4 out of 8

FCFP_12	1294255210	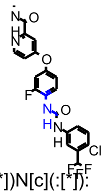 [*]C(=[*])N[c](:[*]):[*]	-0.486	12 out of 22

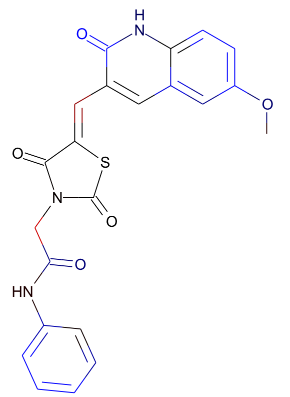

$C_{22}H_{17}N_3O_5S$
Molecular Weight: 435.45248
ALogP: 2.15
Rotatable Bonds: 5
Acceptors: 6
Donors: 2

Model Prediction

Prediction: 83.3
Unit: mg/kg_body_weight/day
Mahalanobis Distance: 13.7
Mahalanobis Distance p-value: 1.71e-009

Mahalanobis Distance: The Mahalanobis distance (MD) is a generalization of the Euclidean distance that accounts for correlations among the X properties. It is calculated as the distance to the center of the training data. The larger the MD, the less trustworthy the prediction.
Mahalanobis Distance p-value: The p-value gives the fraction of training data with an MD greater than or equal to the one for the given sample, assuming normally distributed data. The smaller the p-value, the less trustworthy the prediciton. For highly non-normal X properties (e.g., fingerprints), the MD p-value is wildly inaccurate.

Structural Similar Compounds

Name	Ochratoxin A	542	470
Structure	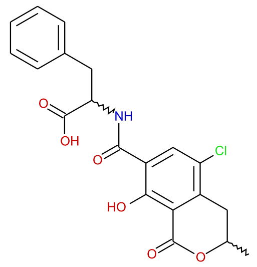	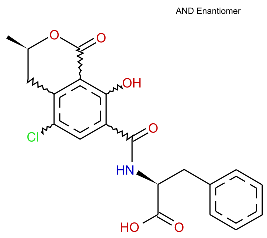	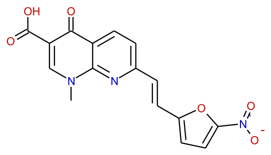
Actual Endpoint (-log C)	4.79932	4.79932	4.62839
Predicted Endpoint (-log C)	3.6353	3.6353	3.93264
Distance	0.642	0.642	0.685
Reference	CPDB	CPDB	CPDB

Model Applicability

Unknown features are fingerprint features in the query molecule, but not found or appearing too infrequently in the training set.

1. OPS PC7 out of range. Value: 5.9597. Training min, max, SD, explained variance: -5.1479, 5.5527, 1.707, 0.0363.
2. Unknown ECFP_2 feature: 2131425032: [*]\C=C(\C=[*])/C(=[*])[*]
3. Unknown ECFP_2 feature: 1182722866: [*]C(=CC(=[*])[*])[*]
4. Unknown ECFP_2 feature: 1000552169: [*]\C=C\1/S[*][*]C1=[*]
5. Unknown ECFP_2 feature: -661097313: [*]CN1C(=[*])[*][*]C1=[*]
6. Unknown ECFP_2 feature: -37698365: [*]N([*])CC(=[*])[*]

Feature Contribution

Top features for positive contribution

Fingerprint	Bit/Smiles	Feature Structure	Score
ECFP_6	1559650422	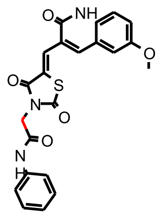 [*]C[*]	0.203

ECFP_6	-1925046727	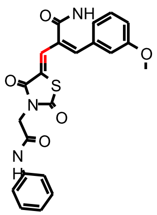 [*]C=[*]	0.145
ECFP_6	-176455838	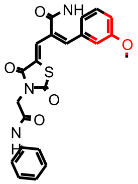 [*]O[c](:[cH]:[*]):[cH]:[*]	0.0818

Top Features for negative contribution

Fingerprint	Bit/Smiles	Feature Structure	Score
ECFP_6	2106656448	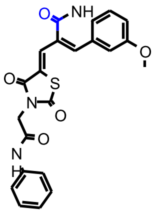 [*]C(=O)[*]	-0.275
ECFP_6	1996767644	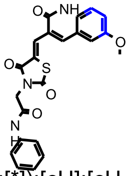 [*][c](:[*]):[cH]:[cH]:[*]	-0.251
ECFP_6	642810091	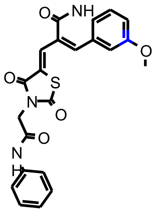 [*][c](:[*]):[*]	-0.247

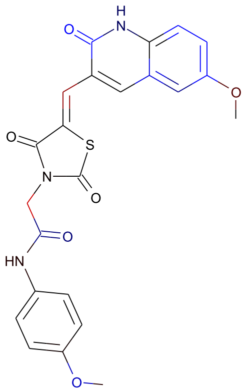

$C_{23}H_{19}N_3O_6S$
Molecular Weight: 465.47846
ALogP: 2.134
Rotatable Bonds: 6
Acceptors: 7
Donors: 2

Model Prediction

Prediction: 37.8
Unit: mg/kg_body_weight/day
Mahalanobis Distance: 13.5
Mahalanobis Distance p-value: 5.33e-009

Mahalanobis Distance: The Mahalanobis distance (MD) is a generalization of the Euclidean distance that accounts for correlations among the X properties. It is calculated as the distance to the center of the training data. The larger the MD, the less trustworthy the prediction.
Mahalanobis Distance p-value: The p-value gives the fraction of training data with an MD greater than or equal to the one for the given sample, assuming normally distributed data. The smaller the p-value, the less trustworthy the prediciton. For highly non-normal X properties (e.g., fingerprints), the MD p-value is wildly inaccurate.

Structural Similar Compounds

Name	Salicylazosulfapyridine	Ochratoxin A	542
Structure	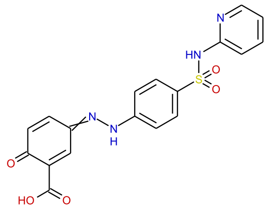	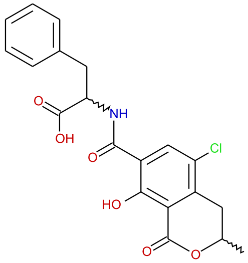	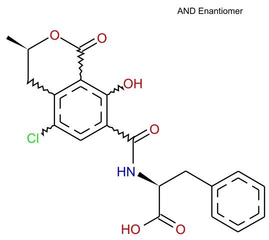
Actual Endpoint (-log C)	2.5034	4.79932	4.79932
Predicted Endpoint (-log C)	3.54214	3.6353	3.6353
Distance	0.671	0.692	0.692
Reference	CPDB	CPDB	CPDB

Model Applicability

Unknown features are fingerprint features in the query molecule, but not found or appearing too infrequently in the training set.

1. All properties and OPS components are within expected ranges.
2. Unknown ECFP_2 feature: 2131425032: [*]\C=C(\C=[*])/C(=[*])[*]
3. Unknown ECFP_2 feature: 1182722866: [*]C(=CC(=[*])[*])[*]
4. Unknown ECFP_2 feature: 1000552169: [*]\C=C\1/S[*][*]C1=[*]
5. Unknown ECFP_2 feature: -661097313: [*]CN1C(=[*])[*][*]C1=[*]
6. Unknown ECFP_2 feature: -37698365: [*]N([*])CC(=[*])[*]

Feature Contribution

Top features for positive contribution

Fingerprint	Bit/Smiles	Feature Structure	Score
ECFP_6	1559650422	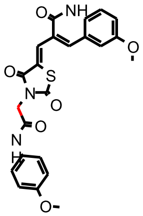 [*]C[*]	0.203

ECFP_6	-1925046727	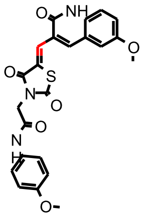 [*]C=[*]	0.145
ECFP_6	-176455838	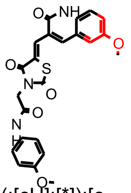 [*]O[c](:[cH]:[*]):[cH]:[*]	0.0818

Top Features for negative contribution

Fingerprint	Bit/Smiles	Feature Structure	Score
ECFP_6	2106656448	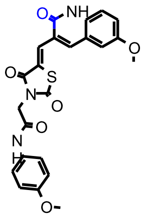 [*]C(=O)[*]	-0.275
ECFP_6	1996767644	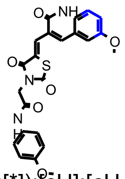 [*][c](:[*]):[cH]:[cH]:[*]	-0.251
ECFP_6	642810091	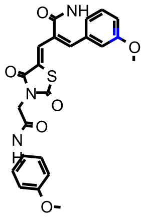 [*][c](:[*]):[*]	-0.247

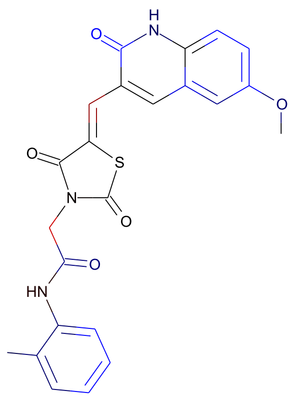

$C_{23}H_{19}N_3O_5S$

Molecular Weight: 449.47906

ALogP: 2.637

Rotatable Bonds: 5

Acceptors: 6

Donors: 2

Model Prediction

Prediction: 97.1

Unit: mg/kg_body_weight/day

Mahalanobis Distance: 15.7

Mahalanobis Distance p-value: 4.02e-015

Mahalanobis Distance: The Mahalanobis distance (MD) is a generalization of the Euclidean distance that accounts for correlations among the X properties. It is calculated as the distance to the center of the training data. The larger the MD, the less trustworthy the prediction.
Mahalanobis Distance p-value: The p-value gives the fraction of training data with an MD greater than or equal to the one for the given sample, assuming normally distributed data. The smaller the p-value, the less trustworthy the prediciton. For highly non-normal X properties (e.g., fingerprints), the MD p-value is wildly inaccurate.

Structural Similar Compounds

Name	Ochratoxin A	542	470
Structure	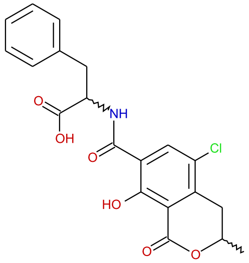	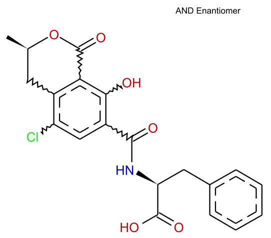	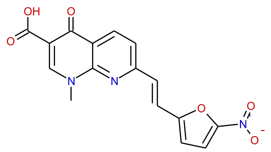
Actual Endpoint (-log C)	4.79932	4.79932	4.62839
Predicted Endpoint (-log C)	3.6353	3.6353	3.93264
Distance	0.644	0.644	0.695
Reference	CPDB	CPDB	CPDB

Model Applicability

Unknown features are fingerprint features in the query molecule, but not found or appearing too infrequently in the training set.

1. All properties and OPS components are within expected ranges.
2. Unknown ECFP_2 feature: 2131425032: [*]\C=C(\C=[*])/C(=[*])[*]
3. Unknown ECFP_2 feature: 1182722866: [*]C(=CC(=[*])[*])[*]
4. Unknown ECFP_2 feature: 1000552169: [*]\C=C\1/S[*][*]C1=[*]
5. Unknown ECFP_2 feature: -661097313: [*]CN1C(=[*])[*][*]C1=[*]
6. Unknown ECFP_2 feature: -37698365: [*]N([*])CC(=[*])[*]

Feature Contribution

Top features for positive contribution

Fingerprint	Bit/Smiles	Feature Structure	Score
ECFP_6	1559650422	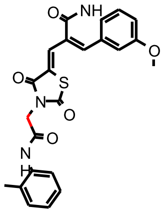 [*]C[*]	0.203

ECFP_6	-1925046727	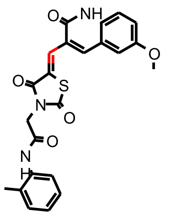 [*]C=[*]	0.145
ECFP_6	-176455838	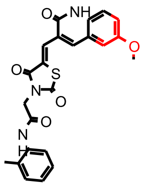 [*]O[c](:[cH]:[*]):[cH]:[*]	0.0818
**Top Features for negative contribution**			
**Fingerprint**	**Bit/Smiles**	**Feature Structure**	**Score**
ECFP_6	2106656448	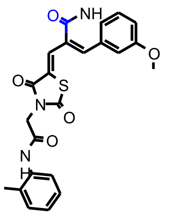 [*]C(=O)[*]	-0.275
ECFP_6	1996767644	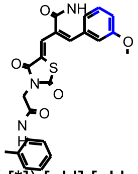 [*][c](:[*]):[cH]:[cH]:[*]	-0.251
ECFP_6	642810091	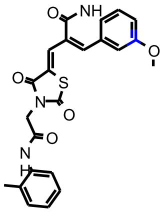 [*][c](:[*]):[*]	-0.247

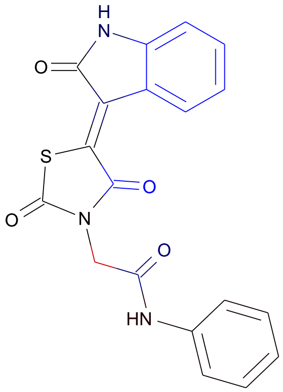

$C_{19}H_{13}N_3O_4S$
Molecular Weight: 379.38922
ALogP: 1.699
Rotatable Bonds: 3
Acceptors: 5
Donors: 2

Model Prediction

Prediction: 74.7
Unit: mg/kg_body_weight/day
Mahalanobis Distance: 11.6
Mahalanobis Distance p-value: 0.000148

Mahalanobis Distance: The Mahalanobis distance (MD) is a generalization of the Euclidean distance that accounts for correlations among the X properties. It is calculated as the distance to the center of the training data. The larger the MD, the less trustworthy the prediction.
Mahalanobis Distance p-value: The p-value gives the fraction of training data with an MD greater than or equal to the one for the given sample, assuming normally distributed data. The smaller the p-value, the less trustworthy the prediciton. For highly non-normal X properties (e.g., fingerprints), the MD p-value is wildly inaccurate.

Structural Similar Compounds

Name	542	Ochratoxin A	470
Structure	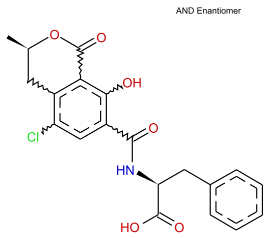	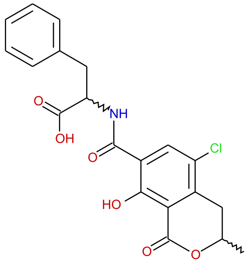	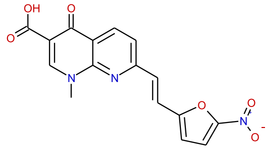
Actual Endpoint (-log C)	4.79932	4.79932	4.62839
Predicted Endpoint (-log C)	3.6353	3.6353	3.93264
Distance	0.684	0.684	0.700
Reference	CPDB	CPDB	CPDB

Model Applicability

Unknown features are fingerprint features in the query molecule, but not found or appearing too infrequently in the training set.

1. All properties and OPS components are within expected ranges.
2. Unknown ECFP_2 feature: -631778390: [*]C(=C1S[*][*]C1=[*])[*]
3. Unknown ECFP_2 feature: -661097313: [*]CN1C(=[*])[*][*]C1=[*]
4. Unknown ECFP_2 feature: -37698365: [*]N([*])CC(=[*])[*]

Feature Contribution

Top features for positive contribution			
Fingerprint	Bit/Smiles	Feature Structure	Score
ECFP_6	1559650422	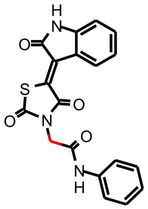 [*]C[*]	0.203

ECFP_6	-1897341097	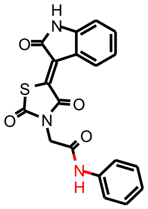<br>[*]N[*]	0.0284
ECFP_6	1571214559	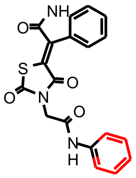<br>[*]1:[cH]:[cH]:[cH]:[cH]:[cH]:1	0.0145
**Top Features for negative contribution**			
**Fingerprint**	**Bit/Smiles**	**Feature Structure**	**Score**
ECFP_6	2106656448	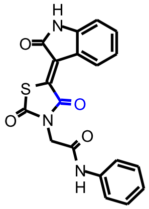<br>[*]C(=O)[*]	-0.275
ECFP_6	1996767644	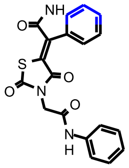<br>[*][c](:[*]):[cH]:[cH]:[*]	-0.251
ECFP_6	642810091	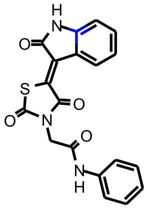<br>[*][c](:[*]):[*]	-0.247

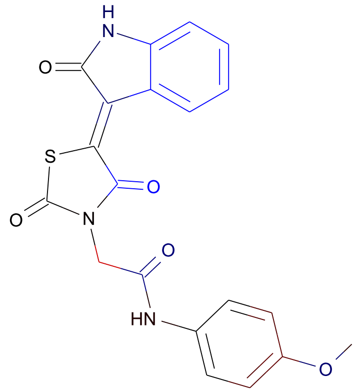

$C_{20}H_{15}N_3O_5S$
Molecular Weight: 409.4152
ALogP: 1.683
Rotatable Bonds: 4
Acceptors: 6
Donors: 2

Model Prediction

Prediction: 93.2
Unit: mg/kg_body_weight/day
Mahalanobis Distance: 14.9
Mahalanobis Distance p-value: 1.1e-012

Mahalanobis Distance: The Mahalanobis distance (MD) is a generalization of the Euclidean distance that accounts for correlations among the X properties. It is calculated as the distance to the center of the training data. The larger the MD, the less trustworthy the prediction.
Mahalanobis Distance p-value: The p-value gives the fraction of training data with an MD greater than or equal to the one for the given sample, assuming normally distributed data. The smaller the p-value, the less trustworthy the prediciton. For highly non-normal X properties (e.g., fingerprints), the MD p-value is wildly inaccurate.

Structural Similar Compounds

Name	470	Ochratoxin A	542
Structure	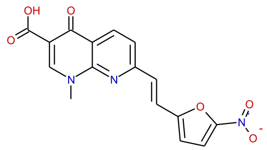	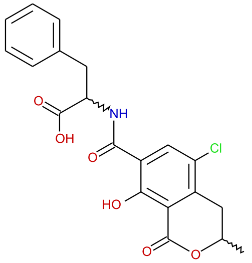	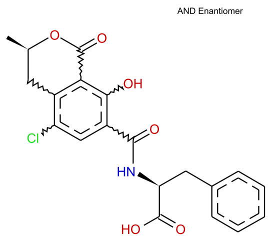
Actual Endpoint (-log C)	4.62839	4.79932	4.79932
Predicted Endpoint (-log C)	3.93264	3.6353	3.6353
Distance	0.669	0.669	0.669
Reference	CPDB	CPDB	CPDB

Model Applicability

Unknown features are fingerprint features in the query molecule, but not found or appearing too infrequently in the training set.

1. OPS PC22 out of range. Value: 4.1413. Training min, max, SD, explained variance: -3.1587, 3.8589, 1.086, 0.0147.
2. Unknown ECFP_2 feature: -631778390: [*]C(=C1S[*][*]C1=[*])[*]
3. Unknown ECFP_2 feature: -661097313: [*]CN1C(=[*])[*][*]C1=[*]
4. Unknown ECFP_2 feature: -37698365: [*]N([*])CC(=[*])[*]

Feature Contribution

Top features for positive contribution			
Fingerprint	Bit/Smiles	Feature Structure	Score
ECFP_6	1559650422	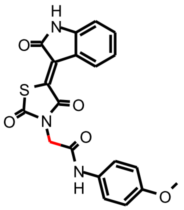 [*]C[*]	0.203

ECFP_6	-176455838	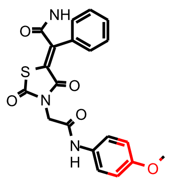 [*]O[c](:[cH]:[*]):[cH]:[*]	0.0818
ECFP_6	734603939	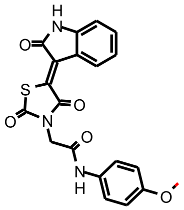 [*]C	0.0424
**Top Features for negative contribution**			
**Fingerprint**	**Bit/Smiles**	**Feature Structure**	**Score**
ECFP_6	2106656448	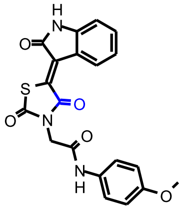 [*]C(=O)[*]	-0.275
ECFP_6	1996767644	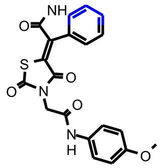 [*][c](:[*]):[cH]:[cH]:[*]	-0.251
ECFP_6	642810091	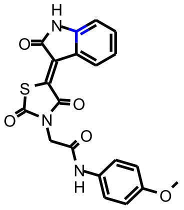 [*][c](:[*]):[*]	-0.247

Sorafenib

TOPKAT_Carcinogenic_Potency_TD50_Mouse

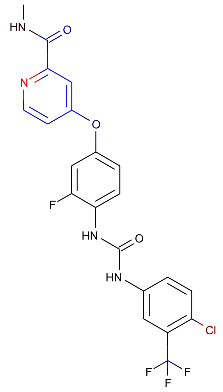

$C_{21}H_{15}ClF_4N_4O_3$

Molecular Weight: 482.81541

ALogP: 4.381

Rotatable Bonds: 6

Acceptors: 4

Donors: 3

Structural Similar Compounds

Name	Ochratoxin A	542	4-Chloro-6-(2,3-xylidino)-2-pyri-mi-dinylthio(N-b-hydroxy-ethyl) acetamide
Structure	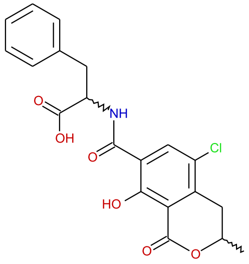	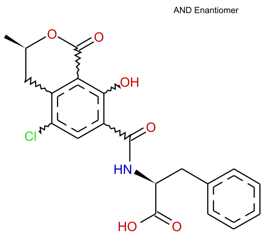	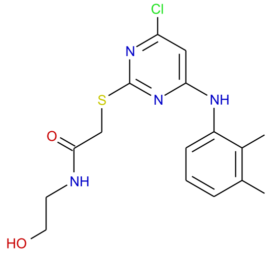
Actual Endpoint (-log C)	4.79932	4.79932	3.91517
Predicted Endpoint (-log C)	3.6353	3.6353	3.92186
Distance	0.731	0.731	0.748
Reference	CPDB	CPDB	CPDB

Model Prediction

Prediction: 17.5

Unit: mg/kg_body_weight/day

Mahalanobis Distance: 12.4

Mahalanobis Distance p-value: 2.31e-006

Mahalanobis Distance: The Mahalanobis distance (MD) is a generalization of the Euclidean distance that accounts for correlations among the X properties. It is calculated as the distance to the center of the training data. The larger the MD, the less trustworthy the prediction.

Mahalanobis Distance p-value: The p-value gives the fraction of training data with an MD greater than or equal to the one for the given sample, assuming normally distributed data. The smaller the p-value, the less trustworthy the prediciton. For highly non-normal X properties (e.g., fingerprints), the MD p-value is wildly inaccurate.

Model Applicability

Unknown features are fingerprint features in the query molecule, but not found or appearing too infrequently in the training set.

1. All properties and OPS components are within expected ranges.
2. Unknown ECFP_2 feature: 1413420509: [*]C(=[*])[c](:n:[*]):c:[*]
3. Unknown ECFP_2 feature: 1338334141: [*]C(=[*])NC
4. Unknown ECFP_2 feature: -1311285389: [*][c](:[*]):[c](F):c:[*]

Feature Contribution

Top features for positive contribution

Fingerprint	Bit/Smiles	Feature Structure	Score
ECFP_6	655739385	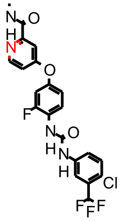 [*]:n:[*]	0.229

ECFP_6	-817402818	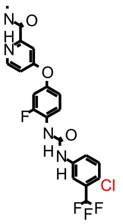<br>[*]Cl	0.129
ECFP_6	-176455838	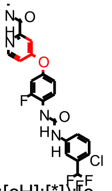<br>[*]O[c](:[cH]:[*]):[c H]:[*]	0.0818
**Top Features for negative contribution**			
**Fingerprint**	**Bit/Smiles**	**Feature Structure**	**Score**
ECFP_6	1996767644	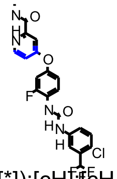<br>[*][c](:[*]):[cH]:[cH]:[*]	-0.251
ECFP_6	642810091	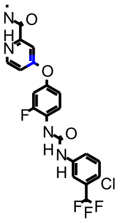<br>[*][c](:[*]):[*]	-0.247
ECFP_6	-182236392	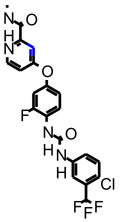<br>[*]:[cH]:[*]	-0.232

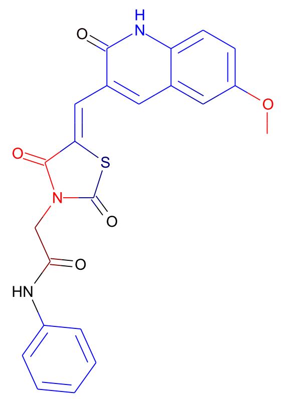

$C_{22}H_{17}N_3O_5S$
Molecular Weight: 435.45248
ALogP: 2.15
Rotatable Bonds: 5
Acceptors: 6
Donors: 2

Model Prediction

Prediction: 149
Unit: mg/kg_body_weight/day
Mahalanobis Distance: 15.4
Mahalanobis Distance p-value: 2.48e-012

Mahalanobis Distance: The Mahalanobis distance (MD) is a generalization of the Euclidean distance that accounts for correlations among the X properties. It is calculated as the distance to the center of the training data. The larger the MD, the less trustworthy the prediction.
Mahalanobis Distance p-value: The p-value gives the fraction of training data with an MD greater than or equal to the one for the given sample, assuming normally distributed data. The smaller the p-value, the less trustworthy the prediciton. For highly non-normal X properties (e.g., fingerprints), the MD p-value is wildly inaccurate.

Structural Similar Compounds

Name	Ochratoxin A	542	Salicylazosulfapyridine
Structure	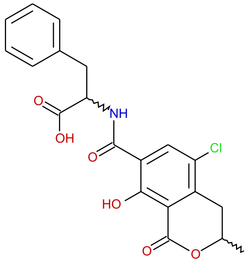	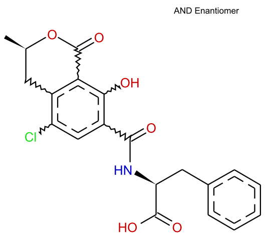	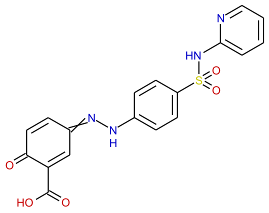
Actual Endpoint (-log C)	6.47264	6.59334	2.39891
Predicted Endpoint (-log C)	5.06501	5.06501	3.17598
Distance	0.648	0.648	0.663
Reference	CPDB	CPDB	CPDB

Model Applicability

Unknown features are fingerprint features in the query molecule, but not found or appearing too infrequently in the training set.

1. OPS PC7 out of range. Value: -6.2114. Training min, max, SD, explained variance: -5.0422, 6.1749, 1.868, 0.0335.

Feature Contribution

Top features for positive contribution

Fingerprint	Bit/Smiles	Feature Structure	Score
FCFP_6	136627117	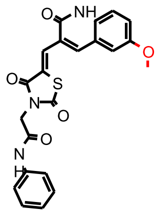 [*]OC	0.69

FCFP_6	565998553	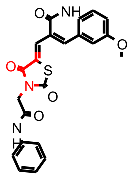<br>[*]N1[*][*]C(=[*])C1=O	0.357
FCFP_6	1	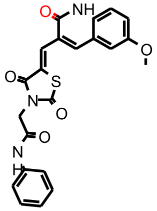<br>[*]=O	0.234
**Top Features for negative contribution**			
**Fingerprint**	**Bit/Smiles**	**Feature Structure**	**Score**
FCFP_6	451847724	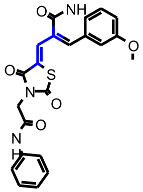<br>[*]C(=CC(=[*])[*])[*]	-0.436
FCFP_6	991735244	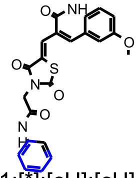<br>[*][c]1:[*]:[cH]:[cH]:[cH]:[cH]:1	-0.422
FCFP_6	436886043	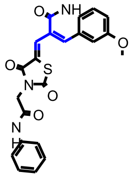<br>[*]\C=C(\C=[*])/C(=[*])[*]	-0.383

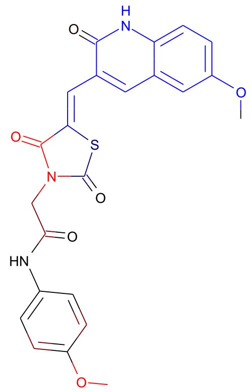

$C_{23}H_{19}N_3O_6S$
Molecular Weight: 465.47846
ALogP: 2.134
Rotatable Bonds: 6
Acceptors: 7
Donors: 2

Structural Similar Compounds

Name	Salicylazosulfapyridine	C.I. direct brown 95	Ochratoxin A
Structure	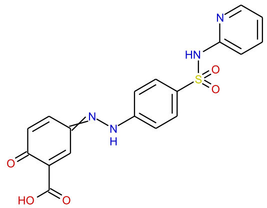	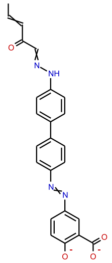	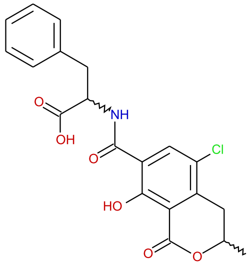
Actual Endpoint (-log C)	2.39891	5.31387	6.47264
Predicted Endpoint (-log C)	3.17598	4.30266	5.06501
Distance	0.650	0.697	0.703
Reference	CPDB	CPDB	CPDB

Model Prediction

Prediction: 22.9
Unit: mg/kg_body_weight/day
Mahalanobis Distance: 14.3
Mahalanobis Distance p-value: 2.95e-009

Mahalanobis Distance: The Mahalanobis distance (MD) is a generalization of the Euclidean distance that accounts for correlations among the X properties. It is calculated as the distance to the center of the training data. The larger the MD, the less trustworthy the prediction.
Mahalanobis Distance p-value: The p-value gives the fraction of training data with an MD greater than or equal to the one for the given sample, assuming normally distributed data. The smaller the p-value, the less trustworthy the prediciton. For highly non-normal X properties (e.g., fingerprints), the MD p-value is wildly inaccurate.

Model Applicability

Unknown features are fingerprint features in the query molecule, but not found or appearing too infrequently in the training set.

1. OPS PC7 out of range. Value: -5.9796. Training min, max, SD, explained variance: -5.0422, 6.1749, 1.868, 0.0335.

Feature Contribution

Top features for positive contribution

Fingerprint	Bit/Smiles	Feature Structure	Score
FCFP_6	136627117	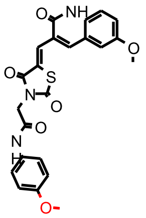 [*]OC	0.69

FCFP_6	565998553	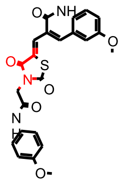 [*]N1[*][*]C(=[*])C1= O	0.357
FCFP_6	1	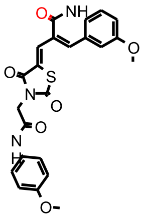 [*]=O	0.234

Top Features for negative contribution			
**Fingerprint**	**Bit/Smiles**	**Feature Structure**	**Score**
FCFP_6	451847724	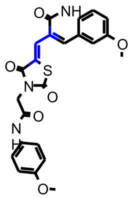 [*]C(=CC(=[*])[*])[*]	-0.436
FCFP_6	436886043	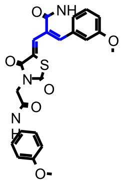 [*]\C=C(\C=[*])/C(=[*])[*]	-0.383
FCFP_6	16	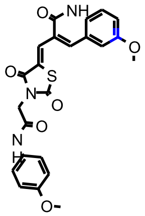 [*][c](:[*]):[*]	-0.354

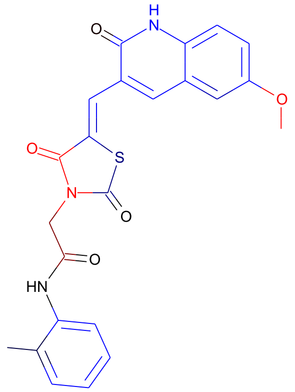

$C_{23}H_{19}N_3O_5S$
Molecular Weight: 449.47906
ALogP: 2.637
Rotatable Bonds: 5
Acceptors: 6
Donors: 2

Model Prediction

Prediction: 67.4
Unit: mg/kg_body_weight/day
Mahalanobis Distance: 15.7
Mahalanobis Distance p-value: 1.9e-013

Mahalanobis Distance: The Mahalanobis distance (MD) is a generalization of the Euclidean distance that accounts for correlations among the X properties. It is calculated as the distance to the center of the training data. The larger the MD, the less trustworthy the prediction.
Mahalanobis Distance p-value: The p-value gives the fraction of training data with an MD greater than or equal to the one for the given sample, assuming normally distributed data. The smaller the p-value, the less trustworthy the prediciton. For highly non-normal X properties (e.g., fingerprints), the MD p-value is wildly inaccurate.

Structural Similar Compounds

Name	Ochratoxin A	542	Salicylazosulfapyridine
Structure	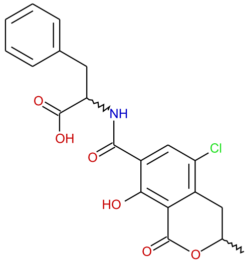	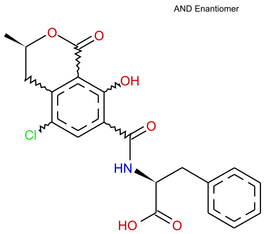	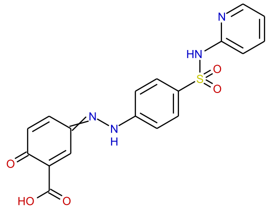
Actual Endpoint (-log C)	6.47264	6.59334	2.39891
Predicted Endpoint (-log C)	5.06501	5.06501	3.17598
Distance	0.642	0.642	0.670
Reference	CPDB	CPDB	CPDB

Model Applicability

Unknown features are fingerprint features in the query molecule, but not found or appearing too infrequently in the training set.

1. OPS PC7 out of range. Value: -5.8922. Training min, max, SD, explained variance: -5.0422, 6.1749, 1.868, 0.0335.

Feature Contribution

Top features for positive contribution

Fingerprint	Bit/Smiles	Feature Structure	Score
FCFP_6	136627117	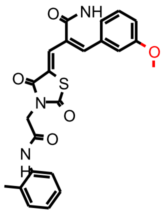 [*]OC	0.69

FCFP_6	565998553	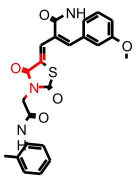 [*]N1[*][*]C(=[*])C1=O	0.357
FCFP_6	1	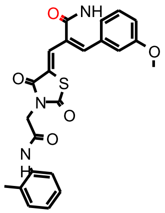 [*]=O	0.234

Top Features for negative contribution

Fingerprint	Bit/Smiles	Feature Structure	Score
FCFP_6	451847724	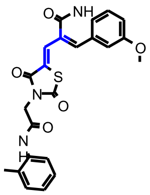 [*]C(=CC(=[*])[*])[*]	-0.436
FCFP_6	991735244	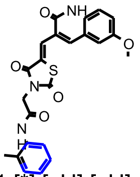 [*][c]1:[*]:[cH]:[cH]:[cH]:[cH]:1	-0.422
FCFP_6	436886043	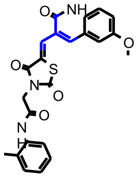 [*]\C=C(\C=[*])/C(=[*])[*]	-0.383

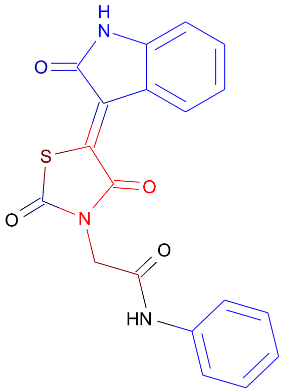

$C_{19}H_{13}N_3O_4S$
Molecular Weight: 379.38922
ALogP: 1.699
Rotatable Bonds: 3
Acceptors: 5
Donors: 2

Structural Similar Compounds

Name	4,4´-Sulfonylbisacetanilide	1,2-Dihydro-2-(5-nitro-2-thi-enyl) quinazolin-4(3H)-one	542
Structure	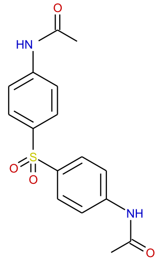	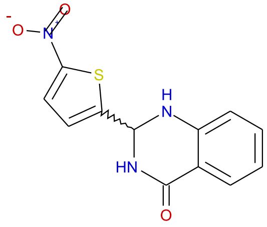	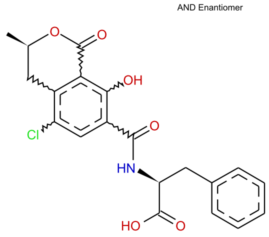
Actual Endpoint (-log C)	3.77655	5.25509	6.59334
Predicted Endpoint (-log C)	3.55337	3.89291	5.06501
Distance	0.618	0.622	0.655
Reference	CPDB	CPDB	CPDB

Model Prediction

Prediction: 53.2
Unit: mg/kg_body_weight/day
Mahalanobis Distance: 10.7
Mahalanobis Distance p-value: 0.043

Mahalanobis Distance: The Mahalanobis distance (MD) is a generalization of the Euclidean distance that accounts for correlations among the X properties. It is calculated as the distance to the center of the training data. The larger the MD, the less trustworthy the prediction.
Mahalanobis Distance p-value: The p-value gives the fraction of training data with an MD greater than or equal to the one for the given sample, assuming normally distributed data. The smaller the p-value, the less trustworthy the prediciton. For highly non-normal X properties (e.g., fingerprints), the MD p-value is wildly inaccurate.

Model Applicability

Unknown features are fingerprint features in the query molecule, but not found or appearing too infrequently in the training set.

1. All properties and OPS components are within expected ranges.

Feature Contribution

Top features for positive contribution

Fingerprint	Bit/Smiles	Feature Structure	Score
FCFP_6	565998553	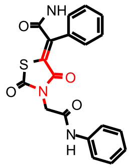 [*]N1[*][*]C(=[*])C1=O	0.357

FCFP_6	1	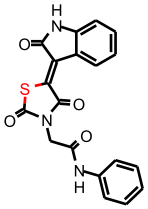 [*]=O	0.234
FCFP_6	203677720	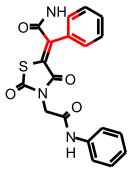 [*][c](:[*]):[c](C=[*]):[cH]:[*]	0.137

Top Features for negative contribution

Fingerprint	Bit/Smiles	Feature Structure	Score
FCFP_6	991735244	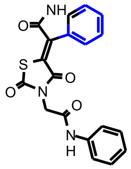 [*][c]1:[*]:[cH]:[cH]:[cH]:[cH]:1	-0.422
FCFP_6	-2093839777	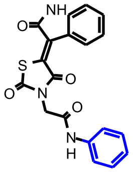 [*][c]1:[cH]:[cH]:[cH]:[cH]:[cH]:1	-0.378
FCFP_6	16	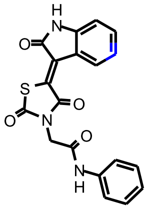 [*][c](:[*]):[*]	-0.354

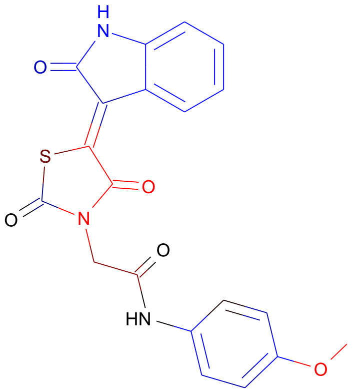

$C_{20}H_{15}N_3O_5S$

Molecular Weight: 409.4152

ALogP: 1.683

Rotatable Bonds: 4

Acceptors: 6

Donors: 2

Model Prediction

Prediction: 8.83

Unit: mg/kg_body_weight/day

Mahalanobis Distance: 13

Mahalanobis Distance p-value: 4.57e-006

Mahalanobis Distance: The Mahalanobis distance (MD) is a generalization of the Euclidean distance that accounts for correlations among the X properties. It is calculated as the distance to the center of the training data. The larger the MD, the less trustworthy the prediction.
Mahalanobis Distance p-value: The p-value gives the fraction of training data with an MD greater than or equal to the one for the given sample, assuming normally distributed data. The smaller the p-value, the less trustworthy the prediciton. For highly non-normal X properties (e.g., fingerprints), the MD p-value is wildly inaccurate.

Structural Similar Compounds

Name	Ochratoxin A	542	Salicylazosulfapyridine
Structure	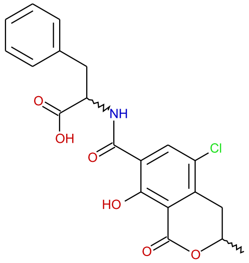	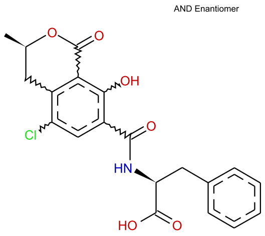	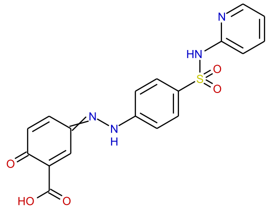
Actual Endpoint (-log C)	6.47264	6.59334	2.39891
Predicted Endpoint (-log C)	5.06501	5.06501	3.17598
Distance	0.651	0.651	0.671
Reference	CPDB	CPDB	CPDB

Model Applicability

Unknown features are fingerprint features in the query molecule, but not found or appearing too infrequently in the training set.

1. OPS PC7 out of range. Value: -5.447. Training min, max, SD, explained variance: -5.0422, 6.1749, 1.868, 0.0335.

Feature Contribution

Top features for positive contribution

Fingerprint	Bit/Smiles	Feature Structure	Score
FCFP_6	136627117	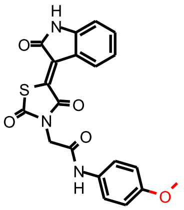 [*]OC	0.69

FCFP_6	565998553	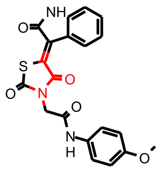 [*]N1[*][*]C(=[*])C1=O	0.357
FCFP_6	1	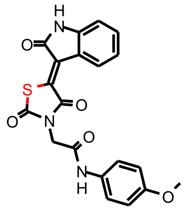 [*]=O	0.234
**Top Features for negative contribution**			
**Fingerprint**	**Bit/Smiles**	**Feature Structure**	**Score**
FCFP_6	991735244	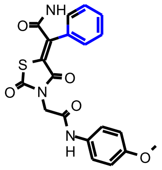 [*][c]1:[*]:[cH]:[cH]:[cH]:[cH]:1	-0.422
FCFP_6	16	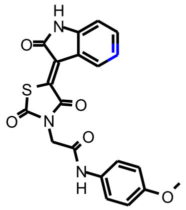 [*][c](:[*]):[*]	-0.354
FCFP_6	590925877	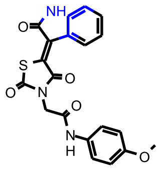 [*]N[c](:[cH]:[*]):[c]([*]):[*]	-0.323

Sorafenib

TOPKAT_Carcinogenic_Potency_TD50_Rat

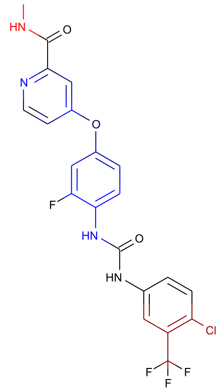

$C_{21}H_{15}ClF_4N_4O_3$
Molecular Weight: 482.81541
ALogP: 4.381
Rotatable Bonds: 6
Acceptors: 4
Donors: 3

Model Prediction

Prediction: 13.9
Unit: mg/kg_body_weight/day
Mahalanobis Distance: 20.4
Mahalanobis Distance p-value: 1.16e-030

Mahalanobis Distance: The Mahalanobis distance (MD) is a generalization of the Euclidean distance that accounts for correlations among the X properties. It is calculated as the distance to the center of the training data. The larger the MD, the less trustworthy the prediction.
Mahalanobis Distance p-value: The p-value gives the fraction of training data with an MD greater than or equal to the one for the given sample, assuming normally distributed data. The smaller the p-value, the less trustworthy the prediciton. For highly non-normal X properties (e.g., fingerprints), the MD p-value is wildly inaccurate.

Structural Similar Compounds

Name	Fluvastatin	913	Ochratoxin A
Structure	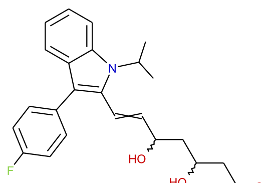	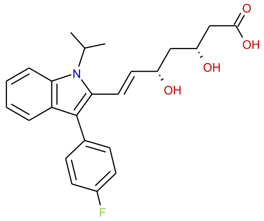	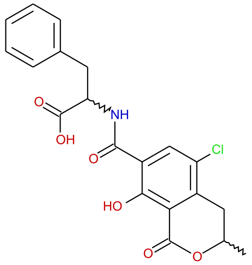
Actual Endpoint (-log C)	3.51742	3.51742	6.47264
Predicted Endpoint (-log C)	5.41573	5.41573	5.06501
Distance	0.611	0.611	0.682
Reference	CPDB	CPDB	CPDB

Model Applicability

Unknown features are fingerprint features in the query molecule, but not found or appearing too infrequently in the training set.

1. All properties and OPS components are within expected ranges.
2. Unknown FCFP_2 feature: -1029533685: [*]:[c](:[*])C(F)(F)F

Feature Contribution

Top features for positive contribution

Fingerprint	Bit/Smiles	Feature Structure	Score
FCFP_6	1	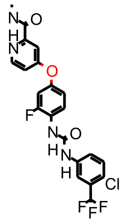 [*]=O	0.234

FCFP_6	-885550502	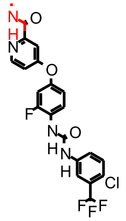 [*]C(=[*])NC	0.229
FCFP_6	32	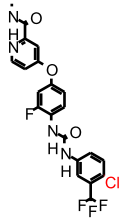 [*]Cl	0.154
**Top Features for negative contribution**			
**Fingerprint**	**Bit/Smiles**	**Feature Structure**	**Score**
FCFP_6	16	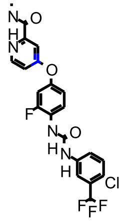 [*][c](:[*]):[*]	-0.354
FCFP_6	590925877	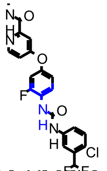 [*]N[c](:[cH]:[*]):[c]([*]):[*]	-0.323
FCFP_6	1674451008	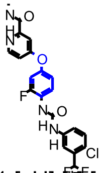 [*]O[c]1:[cH]:[*]:[c]([*]):[cH]:[cH]:1	-0.233

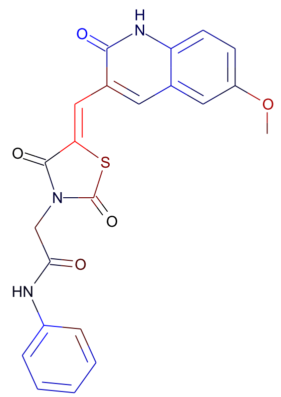

$C_{22}H_{17}N_3O_5S$
Molecular Weight: 435.45248
ALogP: 2.15
Rotatable Bonds: 5
Acceptors: 6
Donors: 2

Model Prediction

Prediction: 0.00466
Unit: g/kg_body_weight
Mahalanobis Distance: 32.5
Mahalanobis Distance p-value: 3.07e-029

Mahalanobis Distance: The Mahalanobis distance (MD) is a generalization of the Euclidean distance that accounts for correlations among the X properties. It is calculated as the distance to the center of the training data. The larger the MD, the less trustworthy the prediction.
Mahalanobis Distance p-value: The p-value gives the fraction of training data with an MD greater than or equal to the one for the given sample, assuming normally distributed data. The smaller the p-value, the less trustworthy the prediciton. For highly non-normal X properties (e.g., fingerprints), the MD p-value is wildly inaccurate.

Structural Similar Compounds

Name	GLIPIZIDE	CHLORSULFURON	PENICILLIN VK
Structure	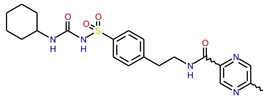	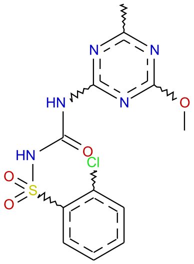	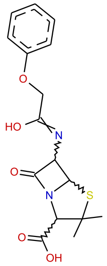
Actual Endpoint (-log C)	3.94991	4.15566	2.99188
Predicted Endpoint (-log C)	3.95594	3.79771	4.18433
Distance	0.583	0.603	0.611
Reference	NDA-17583	EPA COVER SHEET 0027;880301;(1)	NTP REPORT # 336

Model Applicability

Unknown features are fingerprint features in the query molecule, but not found or appearing too infrequently in the training set.

1. OPS PC22 out of range. Value: -6.8867. Training min, max, SD, explained variance: -4.3287, 5.3383, 1.588, 0.0110.
2. Unknown ECFP_6 feature: -154530762: [*]N[*]
3. Unknown ECFP_6 feature: 912478223: [*]S[*]
4. Unknown ECFP_6 feature: 1335833675: [*]N[c](:[cH]:[*]):[c]([*]):[*]
5. Unknown ECFP_6 feature: 1336666212: [*][c](:[*]):[c](C=[*]):[cH]:[*]
6. Unknown ECFP_6 feature: -1699286547: [*]C(=[*])N[c](:[*]):[*]
7. Unknown ECFP_6 feature: 1298725959: [*]NC(=O)C(=[*])[*]
8. Unknown ECFP_6 feature: 2131425032: [*]\C=C(\C=[*])/C(=[*])[*]
9. Unknown ECFP_6 feature: 464808839: [*]C(=C[c](:[*]):[*])[*]
10. Unknown ECFP_6 feature: 1182722866: [*]C(=CC(=[*])[*])[*]
11. Unknown ECFP_6 feature: 1000552169: [*]\C=C\1/S[*][*]C1=[*]
12. Unknown ECFP_6 feature: 1945129186: [*]N1[*][*]C(=[*])C1=O
13. Unknown ECFP_6 feature: -661097313: [*]CN1C(=[*])[*][*]C1=[*]
14. Unknown ECFP_6 feature: 190445529: [*]N1[*][*]SC1=O
15. Unknown ECFP_6 feature: 2122741631: [*]=C1[*][*]C(=[*])S1
16. Unknown ECFP_6 feature: -37698365: [*]N([*])CC(=[*])[*]
17. Unknown ECFP_6 feature: 1731843802: [*]CC(=O)N[*]
18. Unknown ECFP_6 feature: -177077903: [*]N[c](:[cH]:[*]):[cH]:[*]

19. Unknown ECFP_6 feature: 1997021792: [*]:[cH]:[cH]:[cH]:[*]
20. Unknown ECFP_6 feature: 1307307440: [*]:[c](:[*])OC

Feature Contribution

Top features for positive contribution			
**Fingerprint**	**Bit/Smiles**	**Feature Structure**	**Score**
FCFP_6	451847724	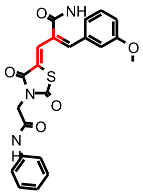 [*]C(=CC(=[*])[*])[*]	0.16
FCFP_6	-1143715940	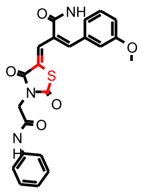 [*]=C1[*][*]C(=[*])S1	0.13
ECFP_6	1559650422	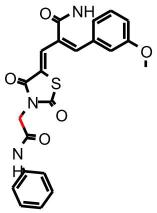 [*]C[*]	0.129

Top Features for negative contribution			
**Fingerprint**	**Bit/Smiles**	**Feature Structure**	**Score**
FCFP_6	991735244	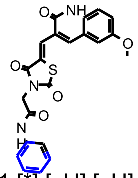 [*][c]1:[*]:[cH]:[cH]:[cH]:[cH]:1	-0.134

ECFP_6	1564392544	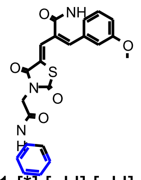 [*][c]1:[*]:[cH]:[cH]:[cH]:[cH]:1	-0.133
ECFP_6	2106656448	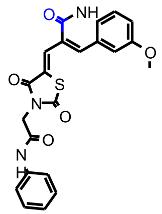 [*]C(=O)[*]	-0.11

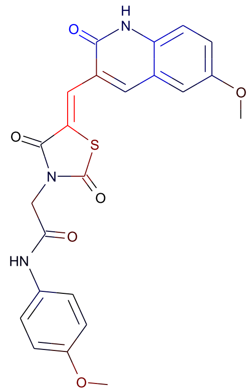

$C_{23}H_{19}N_3O_6S$
Molecular Weight: 465.47846
ALogP: 2.134
Rotatable Bonds: 6
Acceptors: 7
Donors: 2

Model Prediction

Prediction: 0.00345
Unit: g/kg_body_weight
Mahalanobis Distance: 32.1
Mahalanobis Distance p-value: 1.83e-028

Mahalanobis Distance: The Mahalanobis distance (MD) is a generalization of the Euclidean distance that accounts for correlations among the X properties. It is calculated as the distance to the center of the training data. The larger the MD, the less trustworthy the prediction.
Mahalanobis Distance p-value: The p-value gives the fraction of training data with an MD greater than or equal to the one for the given sample, assuming normally distributed data. The smaller the p-value, the less trustworthy the prediciton. For highly non-normal X properties (e.g., fingerprints), the MD p-value is wildly inaccurate.

Structural Similar Compounds

Name	GLIPIZIDE	ALLY	PENICILLIN VK
Structure	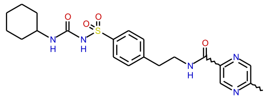	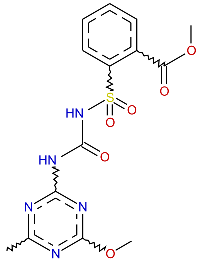	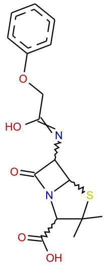
Actual Endpoint (-log C)	3.94991	3.1834	2.99188
Predicted Endpoint (-log C)	3.95594	3.59541	4.18433
Distance	0.574	0.651	0.661
Reference	NDA-17583	EPA COVER SHEET 0288;891101;(1)	NTP REPORT # 336

Model Applicability

Unknown features are fingerprint features in the query molecule, but not found or appearing too infrequently in the training set.

1. OPS PC22 out of range. Value: -6.642. Training min, max, SD, explained variance: -4.3287, 5.3383, 1.588, 0.0110.
2. Unknown ECFP_6 feature: -154530762: [*]N[*]
3. Unknown ECFP_6 feature: 912478223: [*]S[*]
4. Unknown ECFP_6 feature: 1335833675: [*]N[c](:[cH]:[*]):[c]([*]):[*]
5. Unknown ECFP_6 feature: 1336666212: [*][c](:[*]):[c](C=[*]):[cH]:[*]
6. Unknown ECFP_6 feature: -1699286547: [*]C(=[*])N[c](:[*]):[*]
7. Unknown ECFP_6 feature: 1298725959: [*]NC(=O)C(=[*])[*]
8. Unknown ECFP_6 feature: 2131425032: [*]\C=C(\C=[*])/C(=[*])[*]
9. Unknown ECFP_6 feature: 464808839: [*]C(=C[c](:[*]):[*])[*]
10. Unknown ECFP_6 feature: 1182722866: [*]C(=CC(=[*])[*])[*]
11. Unknown ECFP_6 feature: 1000552169: [*]\C=C\1/S[*][*]C1=[*]
12. Unknown ECFP_6 feature: 1945129186: [*]N1[*][*]C(=[*])C1=O
13. Unknown ECFP_6 feature: -661097313: [*]CN1C(=[*])[*][*]C1=[*]
14. Unknown ECFP_6 feature: 190445529: [*]N1[*][*]SC1=O
15. Unknown ECFP_6 feature: 2122741631: [*]=C1[*][*]C(=[*])S1
16. Unknown ECFP_6 feature: -37698365: [*]N([*])CC(=[*])[*]
17. Unknown ECFP_6 feature: 1731843802: [*]CC(=O)N[*]
18. Unknown ECFP_6 feature: -177077903: [*]N[c](:[cH]:[*]):[cH]:[*]

19. Unknown ECFP_6 feature: 1307307440: [*]:[c](:[*])OC

Feature Contribution

Top features for positive contribution

Fingerprint	Bit/Smiles	Feature Structure	Score
FCFP_6	451847724	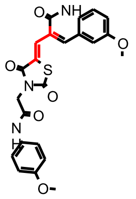 [*]C(=CC(=[*])[*])[*]	0.16
FCFP_6	-1143715940	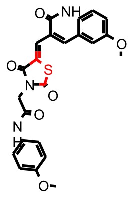 [*]=C1[*][*]C(=[*])S1	0.13
ECFP_6	1559650422	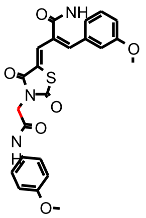 [*]C[*]	0.129

Top Features for negative contribution

Fingerprint	Bit/Smiles	Feature Structure	Score
ECFP_6	2106656448	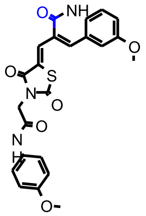 [*]C(=O)[*]	-0.11

FCFP_6	1	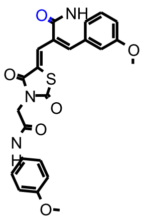 [*]=O	-0.102
ECFP_6	-1236483485	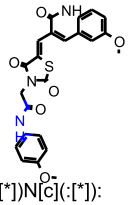 [*]C(=[*])N[c](:[*]):[*]	-0.0747

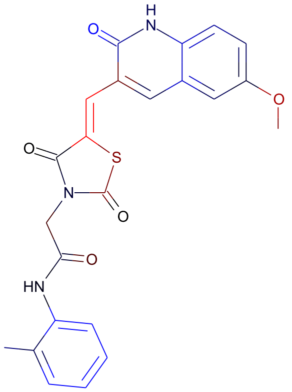

$C_{23}H_{19}N_3O_5S$

Molecular Weight: 449.47906

ALogP: 2.637

Rotatable Bonds: 5

Acceptors: 6

Donors: 2

Model Prediction

Prediction: 0.00653

Unit: g/kg_body_weight

Mahalanobis Distance: 31.5

Mahalanobis Distance p-value: 2.08e-027

Mahalanobis Distance: The Mahalanobis distance (MD) is a generalization of the Euclidean distance that accounts for correlations among the X properties. It is calculated as the distance to the center of the training data. The larger the MD, the less trustworthy the prediction.
Mahalanobis Distance p-value: The p-value gives the fraction of training data with an MD greater than or equal to the one for the given sample, assuming normally distributed data. The smaller the p-value, the less trustworthy the prediciton. For highly non-normal X properties (e.g., fingerprints), the MD p-value is wildly inaccurate.

Structural Similar Compounds

Name	GLIPIZIDE	CHLORSULFURON	PENICILLIN VK
Structure	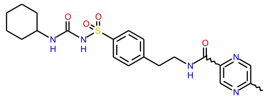	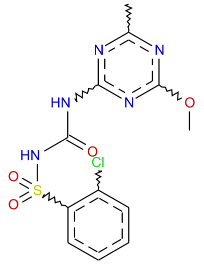	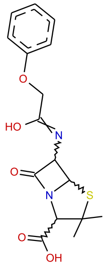
Actual Endpoint (-log C)	3.94991	4.15566	2.99188
Predicted Endpoint (-log C)	3.95594	3.79771	4.18433
Distance	0.590	0.629	0.644
Reference	NDA-17583	EPA COVER SHEET 0027;880301;(1)	NTP REPORT # 336

Model Applicability

Unknown features are fingerprint features in the query molecule, but not found or appearing too infrequently in the training set.

1. OPS PC22 out of range. Value: -6.9885. Training min, max, SD, explained variance: -4.3287, 5.3383, 1.588, 0.0110.
2. Unknown ECFP_6 feature: -154530762: [*]N[*]
3. Unknown ECFP_6 feature: 912478223: [*]S[*]
4. Unknown ECFP_6 feature: 1335833675: [*]N[c](:[cH]:[*]):[c]([*]):[*]
5. Unknown ECFP_6 feature: 1336666212: [*][c](:[*]):[c](C=[*]):[cH]:[*]
6. Unknown ECFP_6 feature: -1699286547: [*]C(=[*])N[c](:[*]):[*]
7. Unknown ECFP_6 feature: 1298725959: [*]NC(=O)C(=[*])[*]
8. Unknown ECFP_6 feature: 2131425032: [*]\C=C(\C=[*])/C(=[*])[*]
9. Unknown ECFP_6 feature: 464808839: [*]C(=C[c](:[*]):[*])[*]
10. Unknown ECFP_6 feature: 1182722866: [*]C(=CC(=[*])[*])[*]
11. Unknown ECFP_6 feature: 1000552169: [*]\C=C\1/S[*][*]C1=[*]
12. Unknown ECFP_6 feature: 1945129186: [*]N1[*][*]C(=[*])C1=O
13. Unknown ECFP_6 feature: -661097313: [*]CN1C(=[*])[*][*]C1=[*]
14. Unknown ECFP_6 feature: 190445529: [*]N1[*][*]SC1=O
15. Unknown ECFP_6 feature: 2122741631: [*]=C1[*][*]C(=[*])S1
16. Unknown ECFP_6 feature: -37698365: [*]N([*])CC(=[*])[*]
17. Unknown ECFP_6 feature: 1731843802: [*]CC(=O)N[*]
18. Unknown ECFP_6 feature: 1335108269: [*]N[c](:[cH]:[*]):[c]([*]):[*]

19. Unknown ECFP_6 feature: 1997021792: [*]:[cH]:[cH]:[cH]:[*]
20. Unknown ECFP_6 feature: 1307307440: [*]:[c](:[*])OC

Feature Contribution

Top features for positive contribution			
**Fingerprint**	**Bit/Smiles**	**Feature Structure**	**Score**
FCFP_6	451847724	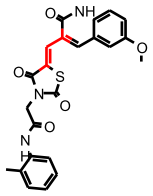 [*]C(=CC(=[*])[*])[*]	0.16
FCFP_6	-1143715940	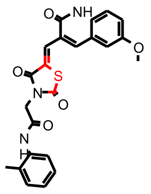 [*]=C1[*][*]C(=[*])S1	0.13
ECFP_6	1559650422	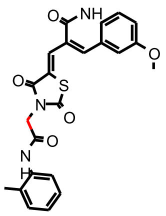 [*]C[*]	0.129

Top Features for negative contribution			
**Fingerprint**	**Bit/Smiles**	**Feature Structure**	**Score**
FCFP_6	991735244	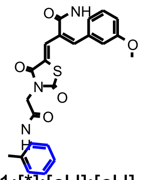 [*][c]1:[*]:[cH]:[cH] :[cH]:[cH]:1	-0.134

ECFP_6	1564392544	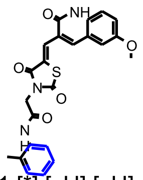 [*][c]1:[*]:[cH]:[cH]:[cH]:[cH]:1	-0.133
ECFP_6	2106656448	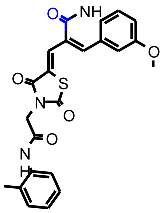 [*]C(=O)[*]	-0.11

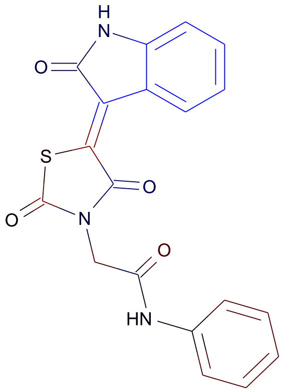

$C_{19}H_{13}N_3O_4S$

Molecular Weight: 379.38922

ALogP: 1.699

Rotatable Bonds: 3

Acceptors: 5

Donors: 2

Model Prediction

Prediction: 0.0399

Unit: g/kg_body_weight

Mahalanobis Distance: 29

Mahalanobis Distance p-value: 7.46e-023

Mahalanobis Distance: The Mahalanobis distance (MD) is a generalization of the Euclidean distance that accounts for correlations among the X properties. It is calculated as the distance to the center of the training data. The larger the MD, the less trustworthy the prediction.
Mahalanobis Distance p-value: The p-value gives the fraction of training data with an MD greater than or equal to the one for the given sample, assuming normally distributed data. The smaller the p-value, the less trustworthy the prediciton. For highly non-normal X properties (e.g., fingerprints), the MD p-value is wildly inaccurate.

Structural Similar Compounds

Name	PIROXICAM	DANTROLENE.NA	CHLORSULFURON
Structure	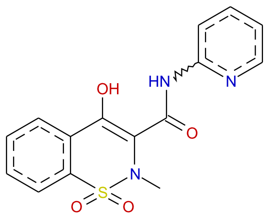	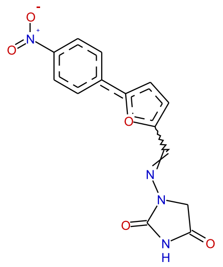	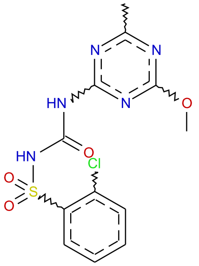
Actual Endpoint (-log C)	5.52028	4.19625	4.15566
Predicted Endpoint (-log C)	4.06087	4.62637	3.79771
Distance	0.523	0.554	0.576
Reference	NDA-18147	NDA-17443	EPA COVER SHEET 0027;880301;(1)

Model Applicability

Unknown features are fingerprint features in the query molecule, but not found or appearing too infrequently in the training set.

1. OPS PC22 out of range. Value: -5.114. Training min, max, SD, explained variance: -4.3287, 5.3383, 1.588, 0.0110.
2. Unknown ECFP_6 feature: -154530762: [*]N[*]
3. Unknown ECFP_6 feature: 912478223: [*]S[*]
4. Unknown ECFP_6 feature: 1997021792: [*]:[cH]:[cH]:[cH]:[*]
5. Unknown ECFP_6 feature: 1335833675: [*]N[c](:[cH]:[*]):[c]([*]):[*]
6. Unknown ECFP_6 feature: -1699286547: [*]C(=[*])N[c](:[*]):[*]
7. Unknown ECFP_6 feature: 1298725959: [*]NC(=O)C(=[*])[*]
8. Unknown ECFP_6 feature: 1790105651: [*]C(=C1C(=[*])[*][*]:[c]1:[*])[*]
9. Unknown ECFP_6 feature: -631778390: [*]C(=C1S[*][*]C1=[*])[*]
10. Unknown ECFP_6 feature: 2122741631: [*]=C1[*][*]C(=[*])S1
11. Unknown ECFP_6 feature: 190445529: [*]N1[*][*]SC1=O
12. Unknown ECFP_6 feature: -661097313: [*]CN1C(=[*])[*][*]C1=[*]
13. Unknown ECFP_6 feature: 1945129186: [*]N1[*][*]C(=[*])C1=O
14. Unknown ECFP_6 feature: -37698365: [*]N([*])CC(=[*])[*]
15. Unknown ECFP_6 feature: 1731843802: [*]CC(=O)N[*]
16. Unknown ECFP_6 feature: -177077903: [*]N[c](:[cH]:[*]):[cH]:[*]

Feature Contribution

Top features for positive contribution

Fingerprint	Bit/Smiles	Feature Structure	Score
FCFP_6	-1143715940	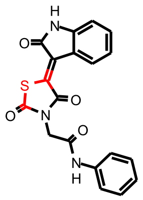 [*]=C1[*][*]C(=[*])S1	0.13
ECFP_6	1559650422	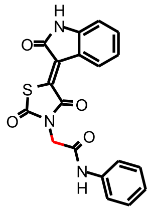 [*]C[*]	0.129
FCFP_6	3	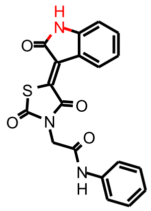 [*]N[*]	0.0924

Top Features for negative contribution

Fingerprint	Bit/Smiles	Feature Structure	Score
FCFP_6	991735244	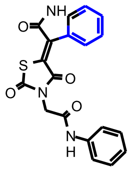 [*][c]1:[*]:[cH]:[cH]:[cH]:[cH]:1	-0.134

ECFP_6	1564392544	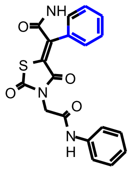 [*][c]1:[*]:[cH]:[cH]:[cH]:[cH]:1	-0.133
ECFP_6	2106656448	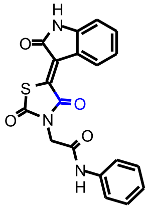 [*]C(=O)[*]	-0.11

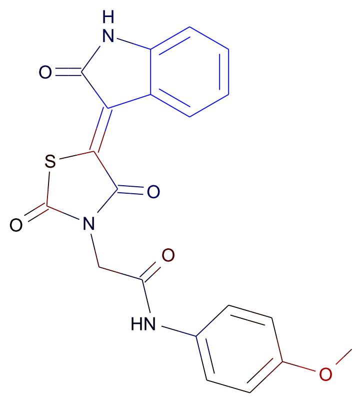

$C_{20}H_{15}N_3O_5S$
Molecular Weight: 409.4152
ALogP: 1.683
Rotatable Bonds: 4
Acceptors: 6
Donors: 2

Structural Similar Compounds

Name	CHLORSULFURON	PENICILLIN VK	PIROXICAM
Structure	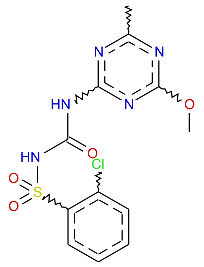	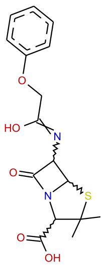	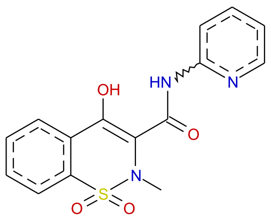
Actual Endpoint (-log C)	4.15566	2.99188	5.52028
Predicted Endpoint (-log C)	3.79771	4.18433	4.06087
Distance	0.543	0.579	0.596
Reference	EPA COVER SHEET 0027;880301;(1)	NTP REPORT # 336	NDA-18147

Model Prediction

Prediction: 0.0185
Unit: g/kg_body_weight
Mahalanobis Distance: 30.3
Mahalanobis Distance p-value: 3.27e-025

Mahalanobis Distance: The Mahalanobis distance (MD) is a generalization of the Euclidean distance that accounts for correlations among the X properties. It is calculated as the distance to the center of the training data. The larger the MD, the less trustworthy the prediction.
Mahalanobis Distance p-value: The p-value gives the fraction of training data with an MD greater than or equal to the one for the given sample, assuming normally distributed data. The smaller the p-value, the less trustworthy the prediciton. For highly non-normal X properties (e.g., fingerprints), the MD p-value is wildly inaccurate.

Model Applicability

Unknown features are fingerprint features in the query molecule, but not found or appearing too infrequently in the training set.

1. OPS PC22 out of range. Value: -5.8211. Training min, max, SD, explained variance: -4.3287, 5.3383, 1.588, 0.0110.
2. Unknown ECFP_6 feature: -154530762: [*]N[*]
3. Unknown ECFP_6 feature: 912478223: [*]S[*]
4. Unknown ECFP_6 feature: 1997021792: [*]:[cH]:[cH]:[cH]:[*]
5. Unknown ECFP_6 feature: 1335833675: [*]N[c](:[cH]:[*]):[c]([*]):[*]
6. Unknown ECFP_6 feature: -1699286547: [*]C(=[*])N[c](:[*]):[*]
7. Unknown ECFP_6 feature: 1298725959: [*]NC(=O)C(=[*])[*]
8. Unknown ECFP_6 feature: 1790105651: [*]C(=C1C(=[*])[*][*]:[c]1:[*])[*]
9. Unknown ECFP_6 feature: -631778390: [*]C(=C1S[*][*]C1=[*])[*]
10. Unknown ECFP_6 feature: 2122741631: [*]=C1[*][*]C(=[*])S1
11. Unknown ECFP_6 feature: 190445529: [*]N1[*][*]SC1=O
12. Unknown ECFP_6 feature: -661097313: [*]CN1C(=[*])[*][*]C1=[*]
13. Unknown ECFP_6 feature: 1945129186: [*]N1[*][*]C(=[*])C1=O
14. Unknown ECFP_6 feature: -37698365: [*]N([*])CC(=[*])[*]
15. Unknown ECFP_6 feature: 1731843802: [*]CC(=O)N[*]
16. Unknown ECFP_6 feature: -177077903: [*]N[c](:[cH]:[*]):[cH]:[*]
17. Unknown ECFP_6 feature: 1307307440: [*]:[c](:[*])OC

Feature Contribution

Top features for positive contribution

Fingerprint	Bit/Smiles	Feature Structure	Score
FCFP_6	-1143715940	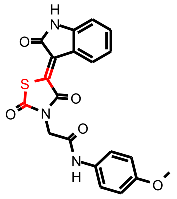 [*]=C1[*][*]C(=[*])S1	0.13
ECFP_6	1559650422	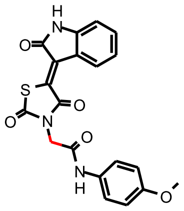 [*]C[*]	0.129
ECFP_6	-176455838	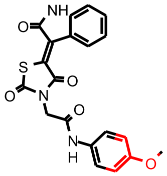 [*]O[c](:[cH]:[*]):[cH]:[*]	0.106

Top Features for negative contribution

Fingerprint	Bit/Smiles	Feature Structure	Score
FCFP_6	991735244	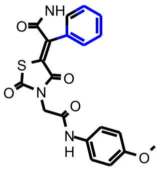 [*][c]1:[*]:[cH]:[cH]:[cH]:[cH]:1	-0.134

ECFP_6	1564392544	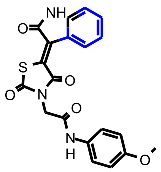 [*][c]1:[*]:[cH]:[cH]:[cH]:[cH]:1	-0.133
ECFP_6	2106656448	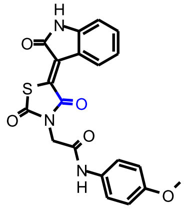 [*]C(=O)[*]	-0.11

Sorafenib

TOPKAT_Chronic_LOAEL

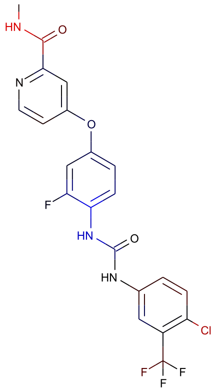

$C_{21}H_{15}ClF_4N_4O_3$

Molecular Weight: 482.81541

ALogP: 4.381

Rotatable Bonds: 6

Acceptors: 4

Donors: 3

Model Prediction

Prediction: 0.00426

Unit: g/kg_body_weight

Mahalanobis Distance: 30.1

Mahalanobis Distance p-value: 9.03e-025

Mahalanobis Distance: The Mahalanobis distance (MD) is a generalization of the Euclidean distance that accounts for correlations among the X properties. It is calculated as the distance to the center of the training data. The larger the MD, the less trustworthy the prediction.

Mahalanobis Distance p-value: The p-value gives the fraction of training data with an MD greater than or equal to the one for the given sample, assuming normally distributed data. The smaller the p-value, the less trustworthy the prediciton. For highly non-normal X properties (e.g., fingerprints), the MD p-value is wildly inaccurate.

Structural Similar Compounds

Name	GLYBURIDE	D & C RED 9	FLUVALINATE
Structure	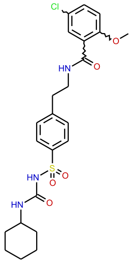	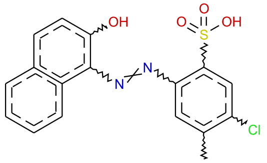	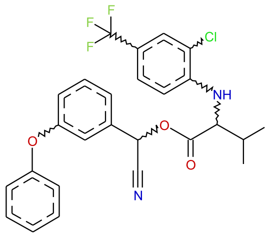
Actual Endpoint (-log C)	4.21661	3.87715	5.30356
Predicted Endpoint (-log C)	4.21035	3.6546	4.89944
Distance	0.635	0.738	0.755
Reference	UPJ-26452	NTP REPORT # 225	EPA COVER SHEET 0281;880630;(1)

Model Applicability

Unknown features are fingerprint features in the query molecule, but not found or appearing too infrequently in the training set.

1. All properties and OPS components are within expected ranges.
2. Unknown ECFP_6 feature: -1046436026: [*]F
3. Unknown ECFP_6 feature: 1305253718: [*]:[c](:[*])O[c](:[*]):[*]
4. Unknown ECFP_6 feature: 1413420509: [*]C(=[*])[c](:[cH]:[*]):n:[*]
5. Unknown ECFP_6 feature: -677309799: [*][c](:[*]):n:[cH]:[*]
6. Unknown ECFP_6 feature: 1996163143: [*]:[cH]:[cH]:n:[*]
7. Unknown ECFP_6 feature: 1430169877: [*]NC(=O)[c](:[*]):[*]
8. Unknown ECFP_6 feature: 1338334141: [*]C(=[*])NC
9. Unknown ECFP_6 feature: 864287155: [*]NC
10. Unknown ECFP_6 feature: 1335108269: [*]N[c](:[cH]:[*]):[c]([*]):[*]
11. Unknown ECFP_6 feature: -1311285389: [*][c](:[*]):[c](F):[cH]:[*]
12. Unknown ECFP_6 feature: -649580166: [*]NC(=O)N[*]
13. Unknown ECFP_6 feature: -177077903: [*]N[c](:[cH]:[*]):[cH]:[*]
14. Unknown ECFP_6 feature: 1336678434: [*][c](:[*]):[c](:[cH]:[*])C([*])([*])[*]
15. Unknown ECFP_6 feature: 99947387: [*]:[c](:[*])Cl
16. Unknown ECFP_6 feature: 220735655: [*]:[c](:[*])F
17. Unknown ECFP_6 feature: -1952889961: [*]:[c](:[*])C(F)(F)F
18. Unknown ECFP_6 feature: 226796801: [*]C([*])([*])F

Feature Contribution

Top features for positive contribution			
**Fingerprint**	**Bit/Smiles**	**Feature Structure**	**Score**
ECFP_6	-176455838	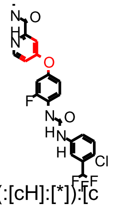 [*]O[c](:[cH]:[*]):[cH]:[*]	0.106
FCFP_6	32	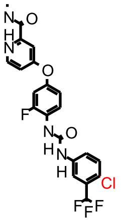 [*]Cl	0.101
FCFP_6	3	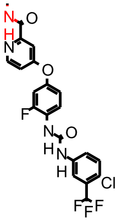 [*]N[*]	0.0924

Top Features for negative contribution			
**Fingerprint**	**Bit/Smiles**	**Feature Structure**	**Score**
FCFP_6	1	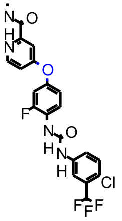 [*]=O	-0.102

ECFP_6	-1236483485	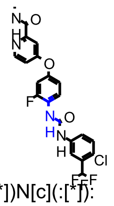<br>[*]C(=[*])N[c](:[*]):<br>[*]	-0.0747
FCFP_6	203677720	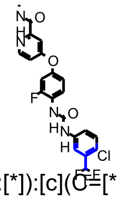<br>[*][c](:[*]):[c](C=[*<br>]):[cH]:[*]	-0.0713

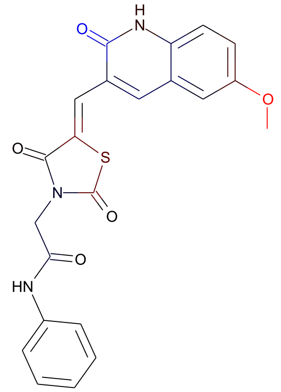

$C_{22}H_{17}N_3O_5S$

Molecular Weight: 435.45248

ALogP: 2.15

Rotatable Bonds: 5

Acceptors: 6

Donors: 2

Model Prediction

Prediction: 0.0217

Unit: g/kg_body_weight

Mahalanobis Distance: 11.6

Mahalanobis Distance p-value: 7.25e-008

Mahalanobis Distance: The Mahalanobis distance (MD) is a generalization of the Euclidean distance that accounts for correlations among the X properties. It is calculated as the distance to the center of the training data. The larger the MD, the less trustworthy the prediction.
Mahalanobis Distance p-value: The p-value gives the fraction of training data with an MD greater than or equal to the one for the given sample, assuming normally distributed data. The smaller the p-value, the less trustworthy the prediciton. For highly non-normal X properties (e.g., fingerprints), the MD p-value is wildly inaccurate.

Structural Similar Compounds

Name	FUROSEMIDE	SALICYLAZOSULFAPYRIDINE	ACETOHEXAMIDE
Structure	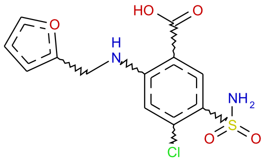	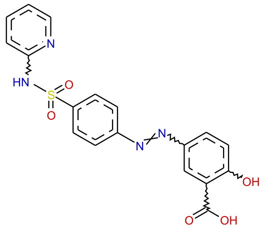	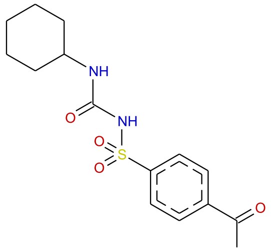
Actual Endpoint (-log C)	4.04236	3.375	2.55683
Predicted Endpoint (-log C)	2.8614	2.80292	3.62413
Distance	0.651	0.729	0.734
Reference	NCI/NTP TR-356	NCI/NTP TR-457	NCI/NTP TR-050

Model Applicability

Unknown features are fingerprint features in the query molecule, but not found or appearing too infrequently in the training set.

1. All properties and OPS components are within expected ranges.

Feature Contribution

Top features for positive contribution			
Fingerprint	Bit/Smiles	Feature Structure	Score
FCFP_2	136627117	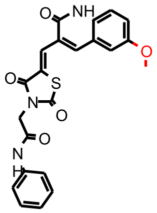 [*]OC	0.173

FCFP_2	-1143715940	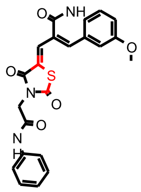 [*]=C1[*][*]C(=[*])S1	0.095
FCFP_2	1036089772	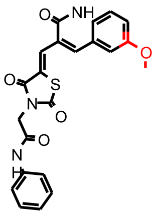 [*]:[c](:[*])OC	0.0749

Top Features for negative contribution

Fingerprint	Bit/Smiles	Feature Structure	Score
FCFP_2	1872154524	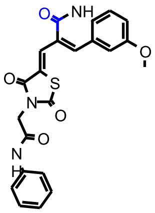 [*]C(=O)[*]	-0.105
FCFP_2	203677720	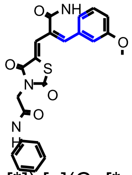 [*][c](:[*]):[c](C=[*])):[cH]:[*]	-0.0829
FCFP_2	1	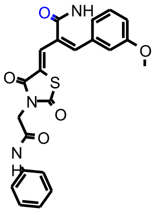 [*]=O	-0.0796

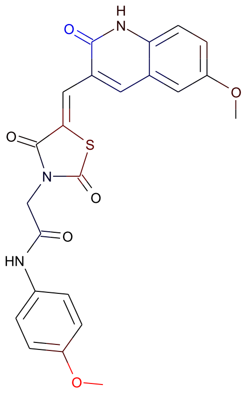

$C_{23}H_{19}N_3O_6S$
Molecular Weight: 465.47846
ALogP: 2.134
Rotatable Bonds: 6
Acceptors: 7
Donors: 2

Model Prediction

Prediction: 0.0209
Unit: g/kg_body_weight
Mahalanobis Distance: 11.2
Mahalanobis Distance p-value: 3.63e-007

Mahalanobis Distance: The Mahalanobis distance (MD) is a generalization of the Euclidean distance that accounts for correlations among the X properties. It is calculated as the distance to the center of the training data. The larger the MD, the less trustworthy the prediction.
Mahalanobis Distance p-value: The p-value gives the fraction of training data with an MD greater than or equal to the one for the given sample, assuming normally distributed data. The smaller the p-value, the less trustworthy the prediciton. For highly non-normal X properties (e.g., fingerprints), the MD p-value is wildly inaccurate.

Structural Similar Compounds

Name	SALICYLAZOSULFAPYRIDINE	FUROSEMIDE	PARATHION
Structure	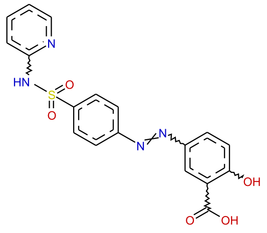	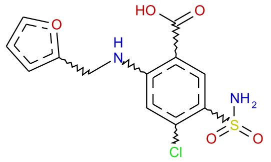	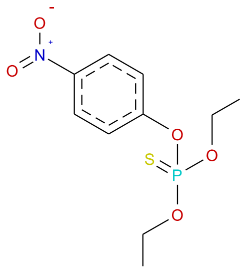
Actual Endpoint (-log C)	3.375	4.04236	5.01172
Predicted Endpoint (-log C)	2.80292	2.8614	3.88389
Distance	0.697	0.715	0.791
Reference	NCI/NTP TR-457	NCI/NTP TR-356	NCI/NTP TR-070

Model Applicability

Unknown features are fingerprint features in the query molecule, but not found or appearing too infrequently in the training set.

1. OPS PC9 out of range. Value: 3.4684. Training min, max, SD, explained variance: -2.8548, 3.3954, 1.263, 0.0360.

Feature Contribution

Top features for positive contribution			
Fingerprint	Bit/Smiles	Feature Structure	Score
FCFP_2	136627117	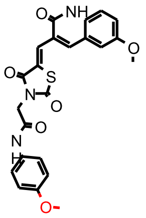 [*]OC	0.173

FCFP_2	-1143715940	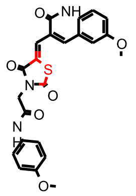 [*]=C1[*][*]C(=[*])S1	0.095
FCFP_2	1036089772	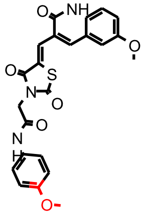 [*]:[c](:[*])OC	0.0749

Top Features for negative contribution

Fingerprint	Bit/Smiles	Feature Structure	Score
FCFP_2	1872154524	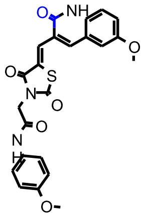 [*]C(=O)[*]	-0.105
FCFP_2	203677720	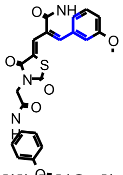 [*][c](:[*]):[c](C=[*]):[cH]:[*]	-0.0829
FCFP_2	1	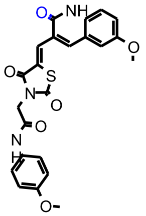 [*]=O	-0.0796

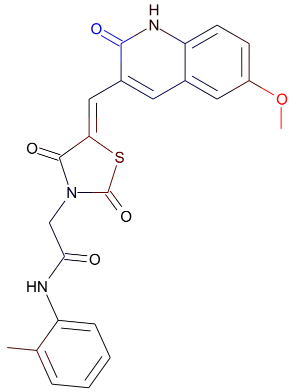

$C_{23}H_{19}N_3O_5S$
Molecular Weight: 449.47906
ALogP: 2.637
Rotatable Bonds: 5
Acceptors: 6
Donors: 2

Model Prediction

Prediction: 0.0178
Unit: g/kg_body_weight
Mahalanobis Distance: 12.2
Mahalanobis Distance p-value: 5.02e-009

Mahalanobis Distance: The Mahalanobis distance (MD) is a generalization of the Euclidean distance that accounts for correlations among the X properties. It is calculated as the distance to the center of the training data. The larger the MD, the less trustworthy the prediction.
Mahalanobis Distance p-value: The p-value gives the fraction of training data with an MD greater than or equal to the one for the given sample, assuming normally distributed data. The smaller the p-value, the less trustworthy the prediciton. For highly non-normal X properties (e.g., fingerprints), the MD p-value is wildly inaccurate.

Structural Similar Compounds

Name	FUROSEMIDE	SALICYLAZOSULFAPYRIDINE	ACETOHEXAMIDE
Structure	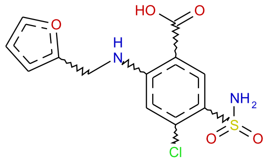	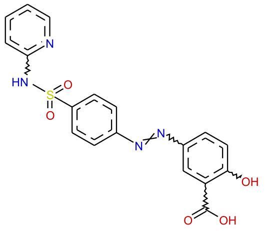	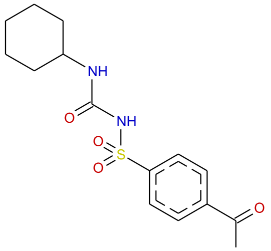
Actual Endpoint (-log C)	4.04236	3.375	2.55683
Predicted Endpoint (-log C)	2.8614	2.80292	3.62413
Distance	0.682	0.726	0.753
Reference	NCI/NTP TR-356	NCI/NTP TR-457	NCI/NTP TR-050

Model Applicability

Unknown features are fingerprint features in the query molecule, but not found or appearing too infrequently in the training set.

1. All properties and OPS components are within expected ranges.

Feature Contribution

Top features for positive contribution			
**Fingerprint**	**Bit/Smiles**	**Feature Structure**	**Score**
FCFP_2	136627117	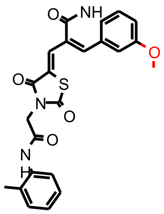 [*]OC	0.173

FCFP_2	-1143715940	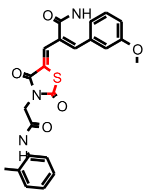 [*]=C1[*][*]C(=[*])S1	0.095
FCFP_2	1036089772	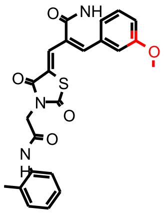 [*]:[c](:[*])OC	0.0749

Top Features for negative contribution

Fingerprint	Bit/Smiles	Feature Structure	Score
FCFP_2	1872154524	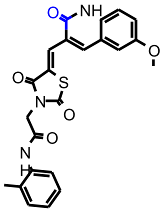 [*]C(=O)[*]	-0.105
FCFP_2	203677720	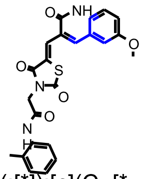 [*][c](:[*]):[c](C=[*]):[cH]:[*]	-0.0829
FCFP_2	1	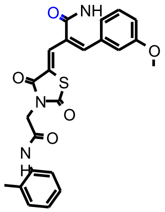 [*]=O	-0.0796

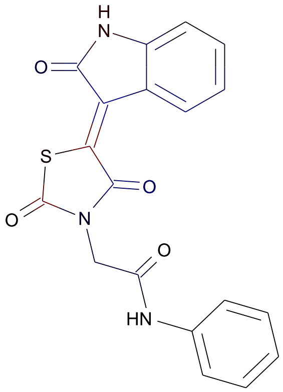

$C_{19}H_{13}N_3O_4S$
Molecular Weight: 379.38922
ALogP: 1.699
Rotatable Bonds: 3
Acceptors: 5
Donors: 2

Model Prediction

Prediction: 0.0482
Unit: g/kg_body_weight
Mahalanobis Distance: 10.3
Mahalanobis Distance p-value: 1.71e-005

Mahalanobis Distance: The Mahalanobis distance (MD) is a generalization of the Euclidean distance that accounts for correlations among the X properties. It is calculated as the distance to the center of the training data. The larger the MD, the less trustworthy the prediction.
Mahalanobis Distance p-value: The p-value gives the fraction of training data with an MD greater than or equal to the one for the given sample, assuming normally distributed data. The smaller the p-value, the less trustworthy the prediciton. For highly non-normal X properties (e.g., fingerprints), the MD p-value is wildly inaccurate.

Structural Similar Compounds

Name	DAPSONE	FUROSEMIDE	ACETOHEXAMIDE
Structure	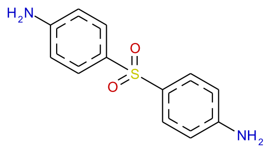	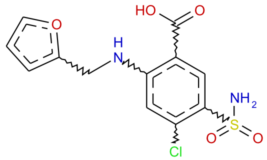	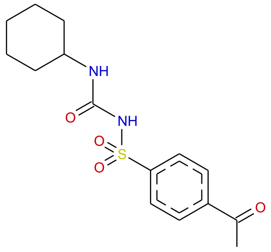
Actual Endpoint (-log C)	3.66258	4.04236	2.55683
Predicted Endpoint (-log C)	3.26993	2.8614	3.62413
Distance	0.557	0.600	0.622
Reference	NCI/NTP TR-20	NCI/NTP TR-356	NCI/NTP TR-050

Model Applicability

Unknown features are fingerprint features in the query molecule, but not found or appearing too infrequently in the training set.

1. All properties and OPS components are within expected ranges.

Feature Contribution

Top features for positive contribution			
**Fingerprint**	**Bit/Smiles**	**Feature Structure**	**Score**
FCFP_2	-1143715940	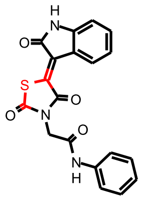 [*]=C1[*][*]C(=[*])S1	0.095

FCFP_2	3	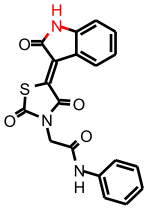 [*]N[*]	0.0737
FCFP_2	565998553	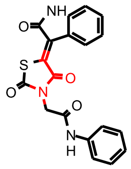 [*]N1[*][*]C(=[*])C1=O	0.00813

Top Features for negative contribution

Fingerprint	Bit/Smiles	Feature Structure	Score
FCFP_2	1872154524	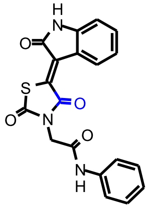 [*]C(=O)[*]	-0.105
FCFP_2	203677720	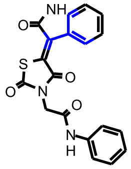 [*][c](:[*]):[c](C=[*]):[cH]:[*]	-0.0829
FCFP_2	1	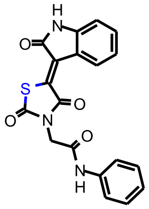 [*]=O	-0.0796

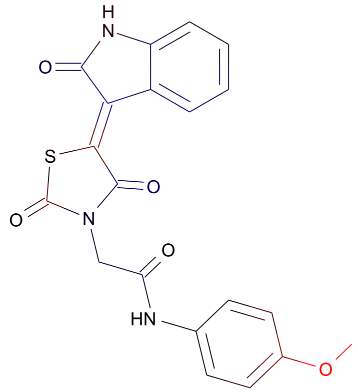

$C_{20}H_{15}N_3O_5S$
Molecular Weight: 409.4152
ALogP: 1.683
Rotatable Bonds: 4
Acceptors: 6
Donors: 2

Model Prediction

Prediction: 0.0229
Unit: g/kg_body_weight
Mahalanobis Distance: 11.6
Mahalanobis Distance p-value: 8.61e-008

Mahalanobis Distance: The Mahalanobis distance (MD) is a generalization of the Euclidean distance that accounts for correlations among the X properties. It is calculated as the distance to the center of the training data. The larger the MD, the less trustworthy the prediction.
Mahalanobis Distance p-value: The p-value gives the fraction of training data with an MD greater than or equal to the one for the given sample, assuming normally distributed data. The smaller the p-value, the less trustworthy the prediciton. For highly non-normal X properties (e.g., fingerprints), the MD p-value is wildly inaccurate.

Structural Similar Compounds

Name	FUROSEMIDE	DAPSONE	3-NITRO-P-ACETOPHENETIDE
Structure	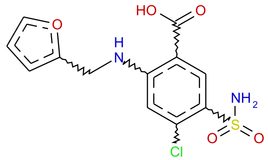	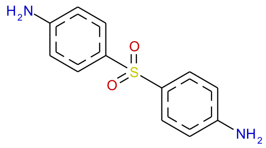	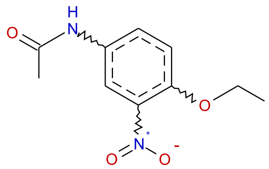
Actual Endpoint (-log C)	4.04236	3.66258	3.11767
Predicted Endpoint (-log C)	2.8614	3.26993	3.42808
Distance	0.617	0.686	0.699
Reference	NCI/NTP TR-356	NCI/NTP TR-20	NCI/NTP TR-133

Model Applicability

Unknown features are fingerprint features in the query molecule, but not found or appearing too infrequently in the training set.

1. All properties and OPS components are within expected ranges.

Feature Contribution

Top features for positive contribution

Fingerprint	Bit/Smiles	Feature Structure	Score
FCFP_2	136627117	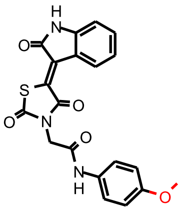 [*]OC	0.173

FCFP_2	-1143715940	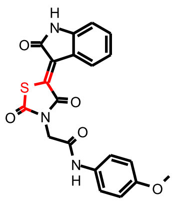 [*]=C1[*][*]C(=[*])S1	0.095
FCFP_2	1036089772	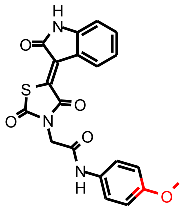 [*]:[c](:[*])OC	0.0749

Top Features for negative contribution

Fingerprint	Bit/Smiles	Feature Structure	Score
FCFP_2	1872154524	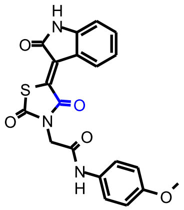 [*]C(=O)[*]	-0.105
FCFP_2	203677720	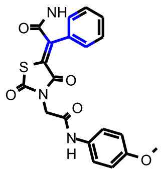 [*][c](:[*]):[c](C=[*]):[cH]:[*]	-0.0829
FCFP_2	1	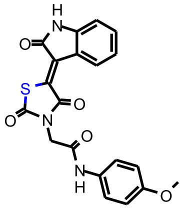 [*]=O	-0.0796

Sorafenib

TOPKAT_Rat_Maximum_Tolerated_Dose_Feed

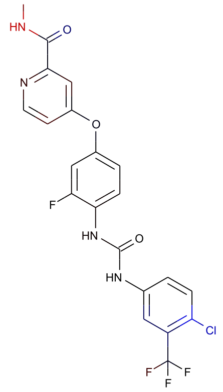

$C_{21}H_{15}ClF_4N_4O_3$

Molecular Weight: 482.81541

ALogP: 4.381

Rotatable Bonds: 6

Acceptors: 4

Donors: 3

Structural Similar Compounds

Name	FUROSEMIDE	PHENOLPHTHALEIN	SALICYLAZOSULFAPYRIDINE
Structure	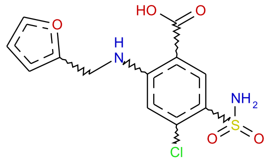	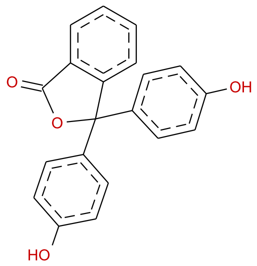	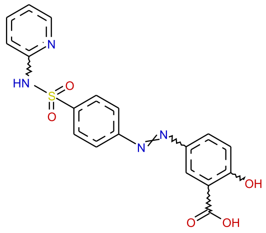
Actual Endpoint (-log C)	4.04236	2.20184	3.375
Predicted Endpoint (-log C)	2.8614	2.8857	2.80292
Distance	0.764	0.801	0.818
Reference	NCI/NTP TR-356	NCI/NTP TR-465	NCI/NTP TR-457

Model Applicability

Unknown features are fingerprint features in the query molecule, but not found or appearing too infrequently in the training set.

1. All properties and OPS components are within expected ranges.

Model Prediction

Prediction: 0.0769

Unit: g/kg_body_weight

Mahalanobis Distance: 12.4

Mahalanobis Distance p-value: 1.91e-009

Mahalanobis Distance: The Mahalanobis distance (MD) is a generalization of the Euclidean distance that accounts for correlations among the X properties. It is calculated as the distance to the center of the training data. The larger the MD, the less trustworthy the prediction.

Mahalanobis Distance p-value: The p-value gives the fraction of training data with an MD greater than or equal to the one for the given sample, assuming normally distributed data. The smaller the p-value, the less trustworthy the prediciton. For highly non-normal X properties (e.g., fingerprints), the MD p-value is wildly inaccurate.

Feature Contribution

Top features for positive contribution

Fingerprint	Bit/Smiles	Feature Structure	Score
FCFP_2	-885550502	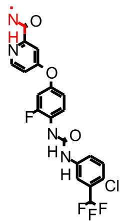 [*]C(=[*])NC	0.115

FCFP_2	3	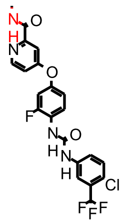 [*]N[*]	0.0737
FCFP_2	332760439	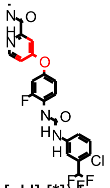 [*]O[c](:[cH]:[*]):[cH]:[*]	0.0611
**Top Features for negative contribution**			
**Fingerprint**	**Bit/Smiles**	**Feature Structure**	**Score**
FCFP_2	71476542	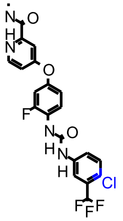 [*]:[c](:[*])Cl	-0.134
FCFP_2	1872154524	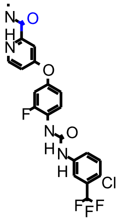 [*]C(=O)[*]	-0.105
FCFP_2	203677720	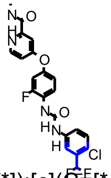 [*][c](:[*]):[c](C=[*]):[cH]:[*]	-0.0829

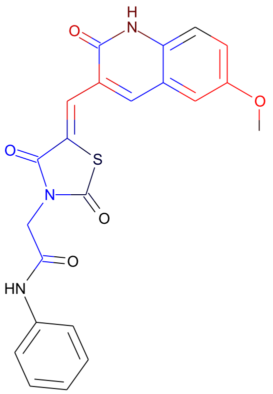

$C_{22}H_{17}N_3O_5S$
Molecular Weight: 435.45248
ALogP: 2.15
Rotatable Bonds: 5
Acceptors: 6
Donors: 2

Model Prediction

Prediction: 0.00971
Unit: g/kg_body_weight
Mahalanobis Distance: 10.2
Mahalanobis Distance p-value: 2.93e-006

Mahalanobis Distance: The Mahalanobis distance (MD) is a generalization of the Euclidean distance that accounts for correlations among the X properties. It is calculated as the distance to the center of the training data. The larger the MD, the less trustworthy the prediction.
Mahalanobis Distance p-value: The p-value gives the fraction of training data with an MD greater than or equal to the one for the given sample, assuming normally distributed data. The smaller the p-value, the less trustworthy the prediciton. For highly non-normal X properties (e.g., fingerprints), the MD p-value is wildly inaccurate.

Structural Similar Compounds

Name	PENICILLIN VK	OCHRATOXIN	SULFISOOXAZOLE
Structure	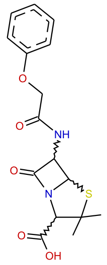	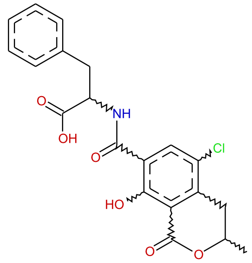	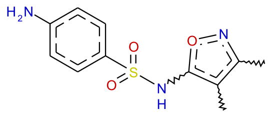
Actual Endpoint (-log C)	2.54455	6.28396	2.82494
Predicted Endpoint (-log C)	3.9702	5.12358	3.0705
Distance	0.634	0.665	0.815
Reference	NCI/NTP TR-336	NCI/NTP TR-358	NCI/NTP TR-138

Model Applicability

Unknown features are fingerprint features in the query molecule, but not found or appearing too infrequently in the training set.

1. Molecular_Weight out of range. Value: 435.45. Training min, max, mean, SD: 68.074, 434.63, 171.13, 85.06.
2. Unknown FCFP_2 feature: 436915834: [*]\C=C\1/S[*][*]C1=[*]
3. Unknown FCFP_2 feature: -1986158408: [*]N1[*][*]SC1=O

Feature Contribution

Top features for positive contribution

Fingerprint	Bit/Smiles	Feature Structure	Score
FCFP_2	332760439	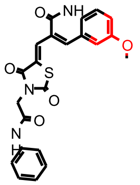 [*]O[c](:[cH]:[*]):[cH]:[*]	0.672

FCFP_2	1	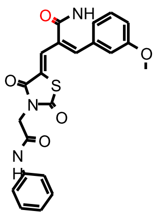 [*]=O	0.511
FCFP_2	451847724	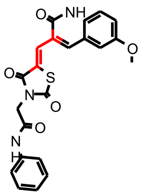 [*]C(=CC(=[*])[*])[*]	0.225

Top Features for negative contribution

Fingerprint	Bit/Smiles	Feature Structure	Score
FCFP_2	203677720	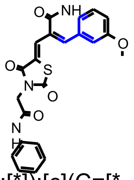 [*][c](:[*]):[c](C=[*])):[cH]:[*]	-0.406
FCFP_2	565998553	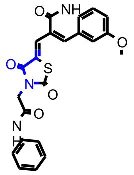 [*]N1[*][*]C(=[*])C1=O	-0.348
FCFP_2	1872154524	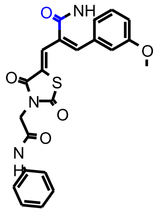 [*]C(=O)[*]	-0.307

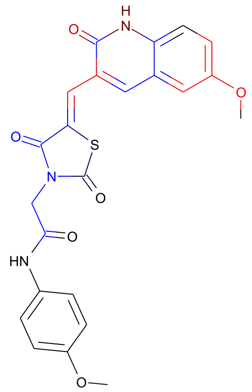

$C_{23}H_{19}N_3O_6S$
Molecular Weight: 465.47846
ALogP: 2.134
Rotatable Bonds: 6
Acceptors: 7
Donors: 2

Model Prediction

Prediction: 0.00383
Unit: g/kg_body_weight
Mahalanobis Distance: 9.95
Mahalanobis Distance p-value: 6.67e-006

Mahalanobis Distance: The Mahalanobis distance (MD) is a generalization of the Euclidean distance that accounts for correlations among the X properties. It is calculated as the distance to the center of the training data. The larger the MD, the less trustworthy the prediction.
Mahalanobis Distance p-value: The p-value gives the fraction of training data with an MD greater than or equal to the one for the given sample, assuming normally distributed data. The smaller the p-value, the less trustworthy the prediciton. For highly non-normal X properties (e.g., fingerprints), the MD p-value is wildly inaccurate.

Structural Similar Compounds

Name	PENICILLIN VK	OCHRATOXIN	SULFISOOXAZOLE
Structure	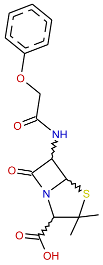	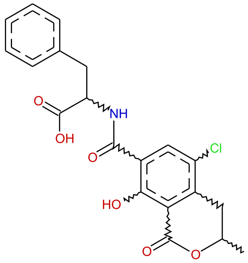	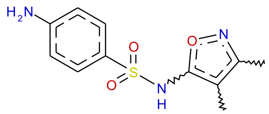
Actual Endpoint (-log C)	2.54455	6.28396	2.82494
Predicted Endpoint (-log C)	3.9702	5.12358	3.0705
Distance	0.700	0.731	0.948
Reference	NCI/NTP TR-336	NCI/NTP TR-358	NCI/NTP TR-138

Model Applicability

Unknown features are fingerprint features in the query molecule, but not found or appearing too infrequently in the training set.

1. Molecular_Weight out of range. Value: 465.48. Training min, max, mean, SD: 68.074, 434.63, 171.13, 85.06.
2. Num_H_Acceptors out of range. Value: 7. Training min, max, mean, SD: 0, 6, 1.6146, 1.644.
3. Molecular_PolarSurfaceArea out of range. Value: 139.33. Training min, max, mean, SD: 0, 138.03, 28.978, 32.1.
4. Unknown FCFP_2 feature: 436915834: [*]\C=C\1/S[*][*]C1=[*]
5. Unknown FCFP_2 feature: -1986158408: [*]N1[*][*]SC1=O

Feature Contribution

Top features for positive contribution

Fingerprint	Bit/Smiles	Feature Structure	Score
FCFP_2	332760439	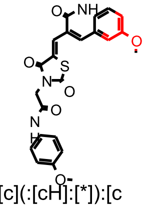 [*]O[c](:[cH]:[*]):[cH]:[*]	0.672

FCFP_2	1	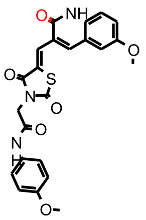 [*]=O	0.511
FCFP_2	451847724	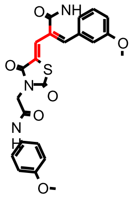 [*]C(=CC(=[*])[*])[*]	0.225

Top Features for negative contribution

**Fingerprint**	**Bit/Smiles**	**Feature Structure**	**Score**
FCFP_2	203677720	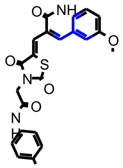 [*][c](:[*]):[c](C=[*]):[cH]:[*]	-0.406
FCFP_2	565998553	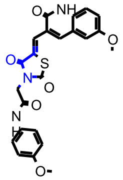 [*]N1[*][*]C(=[*])C1= O	-0.348
FCFP_2	1872154524	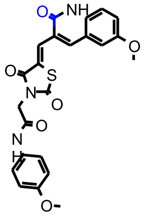 [*]C(=O)[*]	-0.307

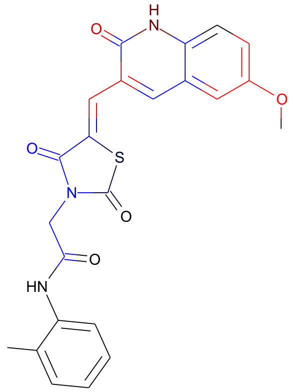

$C_{23}H_{19}N_3O_5S$

Molecular Weight: 449.47906

ALogP: 2.637

Rotatable Bonds: 5

Acceptors: 6

Donors: 2

Model Prediction

Prediction: 0.0074

Unit: g/kg_body_weight

Mahalanobis Distance: 10.5

Mahalanobis Distance p-value: 1.31e-006

Mahalanobis Distance: The Mahalanobis distance (MD) is a generalization of the Euclidean distance that accounts for correlations among the X properties. It is calculated as the distance to the center of the training data. The larger the MD, the less trustworthy the prediction.
Mahalanobis Distance p-value: The p-value gives the fraction of training data with an MD greater than or equal to the one for the given sample, assuming normally distributed data. The smaller the p-value, the less trustworthy the prediciton. For highly non-normal X properties (e.g., fingerprints), the MD p-value is wildly inaccurate.

Structural Similar Compounds

Name	OCHRATOXIN	PENICILLIN VK	SULFISOOXAZOLE
Structure	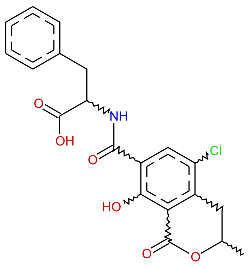	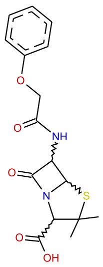	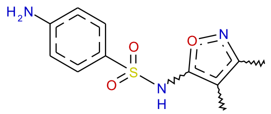
Actual Endpoint (-log C)	6.28396	2.54455	2.82494
Predicted Endpoint (-log C)	5.12358	3.9702	3.0705
Distance	0.658	0.679	0.837
Reference	NCI/NTP TR-358	NCI/NTP TR-336	NCI/NTP TR-138

Model Applicability

Unknown features are fingerprint features in the query molecule, but not found or appearing too infrequently in the training set.

1. Molecular_Weight out of range. Value: 449.48. Training min, max, mean, SD: 68.074, 434.63, 171.13, 85.06.
2. Unknown FCFP_2 feature: 436915834: [*]\C=C\1/S[*][*]C1=[*]
3. Unknown FCFP_2 feature: -1986158408: [*]N1[*][*]SC1=O

Feature Contribution

Top features for positive contribution

Fingerprint	Bit/Smiles	Feature Structure	Score
FCFP_2	332760439	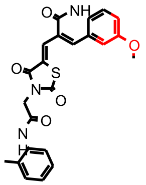 [*]O[c](:[cH]:[*]):[cH]:[*]	0.672

FCFP_2	1	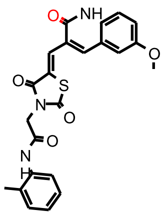 [*]=O	0.511
FCFP_2	451847724	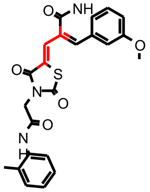 [*]C(=CC(=[*])[*])[*]	0.225
**Top Features for negative contribution**			
**Fingerprint**	**Bit/Smiles**	**Feature Structure**	**Score**
FCFP_2	203677720	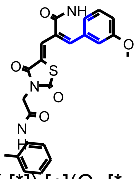 [*][c](:[*]):[c](C=[*]):[cH]:[*]	-0.406
FCFP_2	565998553	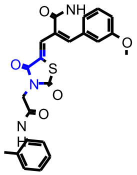 [*]N1[*][*]C(=[*])C1= O	-0.348
FCFP_2	1872154524	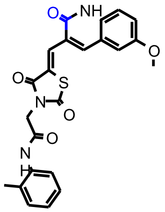 [*]C(=O)[*]	-0.307

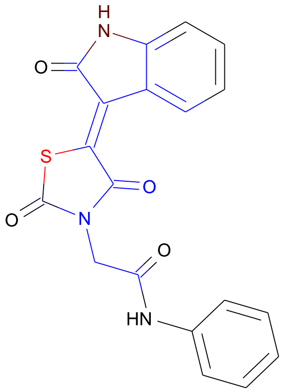

$C_{19}H_{13}N_3O_4S$
Molecular Weight: 379.38922
ALogP: 1.699
Rotatable Bonds: 3
Acceptors: 5
Donors: 2

Model Prediction

Prediction: 0.18
Unit: g/kg_body_weight
Mahalanobis Distance: 7.65
Mahalanobis Distance p-value: 0.00516

Mahalanobis Distance: The Mahalanobis distance (MD) is a generalization of the Euclidean distance that accounts for correlations among the X properties. It is calculated as the distance to the center of the training data. The larger the MD, the less trustworthy the prediction.
Mahalanobis Distance p-value: The p-value gives the fraction of training data with an MD greater than or equal to the one for the given sample, assuming normally distributed data. The smaller the p-value, the less trustworthy the prediciton. For highly non-normal X properties (e.g., fingerprints), the MD p-value is wildly inaccurate.

Structural Similar Compounds

Name	SULFISOOXAZOLE	PENICILLIN VK	OCHRATOXIN
Structure	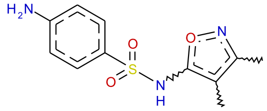	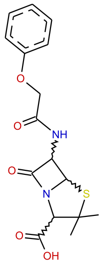	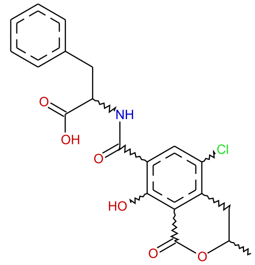
Actual Endpoint (-log C)	2.82494	2.54455	6.28396
Predicted Endpoint (-log C)	3.0705	3.9702	5.12358
Distance	0.596	0.627	0.706
Reference	NCI/NTP TR-138	NCI/NTP TR-336	NCI/NTP TR-358

Model Applicability

Unknown features are fingerprint features in the query molecule, but not found or appearing too infrequently in the training set.

1. All properties and OPS components are within expected ranges.
2. Unknown FCFP_2 feature: -1678275541: [*]C(=C1C(=[*])[*][*]:[c]1:[*])[*]
3. Unknown FCFP_2 feature: 436915834: [*]\C=C\1/S[*][*]C1=[*]
4. Unknown FCFP_2 feature: -1986158408: [*]N1[*][*]SC1=O

Feature Contribution

Top features for positive contribution			
**Fingerprint**	**Bit/Smiles**	**Feature Structure**	**Score**
FCFP_2	1	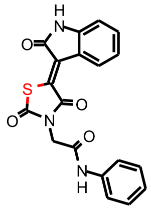 [*]=O	0.511

FCFP_2	3	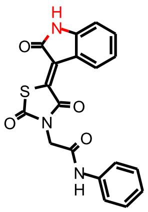 [*]N[*]	0.104
**Top Features for negative contribution**			
**Fingerprint**	**Bit/Smiles**	**Feature Structure**	**Score**
FCFP_2	203677720	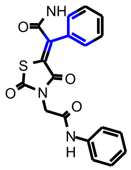 [*][c](:[*]):[c](C=[*]):[cH]:[*]	-0.406
FCFP_2	565998553	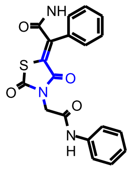 [*]N1[*][*]C(=[*])C1= O	-0.348
FCFP_2	1872154524	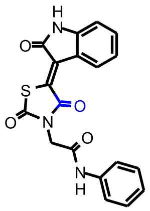 [*]C(=O)[*]	-0.307

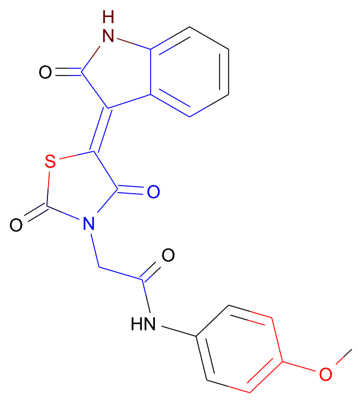

$C_{20}H_{15}N_3O_5S$

Molecular Weight: 409.4152

ALogP: 1.683

Rotatable Bonds: 4

Acceptors: 6

Donors: 2

Model Prediction

Prediction: 0.0142

Unit: g/kg_body_weight

Mahalanobis Distance: 9.94

Mahalanobis Distance p-value: 6.8e-006

Mahalanobis Distance: The Mahalanobis distance (MD) is a generalization of the Euclidean distance that accounts for correlations among the X properties. It is calculated as the distance to the center of the training data. The larger the MD, the less trustworthy the prediction.

Mahalanobis Distance p-value: The p-value gives the fraction of training data with an MD greater than or equal to the one for the given sample, assuming normally distributed data. The smaller the p-value, the less trustworthy the prediciton. For highly non-normal X properties (e.g., fingerprints), the MD p-value is wildly inaccurate.

Structural Similar Compounds

Name	PENICILLIN VK	OCHRATOXIN	SULFISOOXAZOLE
Structure	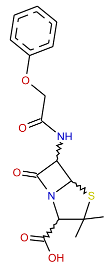	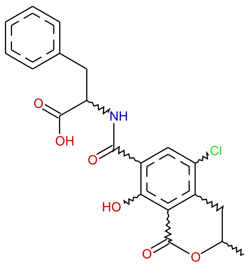	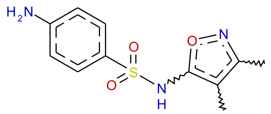
Actual Endpoint (-log C)	2.54455	6.28396	2.82494
Predicted Endpoint (-log C)	3.9702	5.12358	3.0705
Distance	0.583	0.685	0.737
Reference	NCI/NTP TR-336	NCI/NTP TR-358	NCI/NTP TR-138

Model Applicability

Unknown features are fingerprint features in the query molecule, but not found or appearing too infrequently in the training set.

1. All properties and OPS components are within expected ranges.
2. Unknown FCFP_2 feature: -1678275541: [*]C(=C1C(=[*])[*][*]:[c]1:[*])[*]
3. Unknown FCFP_2 feature: 436915834: [*]\C=C\1/S[*][*]C1=[*]
4. Unknown FCFP_2 feature: -1986158408: [*]N1[*][*]SC1=O

Feature Contribution

Top features for positive contribution

Fingerprint	Bit/Smiles	Feature Structure	Score
FCFP_2	332760439	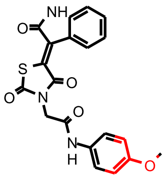 [*]O[c](:[cH]:[*]):[cH]:[*]	0.672

FCFP_2	1	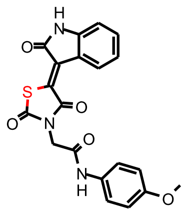 [*]=O	0.511
FCFP_2	3	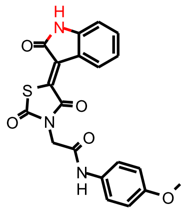 [*]N[*]	0.104
**Top Features for negative contribution**			
**Fingerprint**	**Bit/Smiles**	**Feature Structure**	**Score**
FCFP_2	203677720	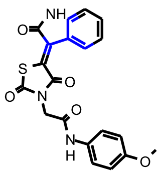 [*][c](:[*]):[c](C=[*]):[cH]:[*]	-0.406
FCFP_2	565998553	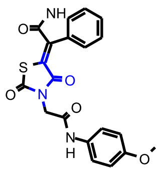 [*]N1[*][*]C(=[*])C1= O	-0.348
FCFP_2	1872154524	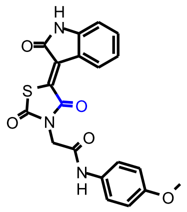 [*]C(=O)[*]	-0.307

Sorafenib

TOPKAT_Rat_Maximum_Tolerated_Dose_Gavage

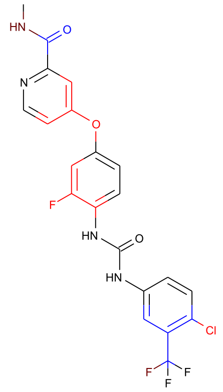

$C_{21}H_{15}ClF_4N_4O_3$
Molecular Weight: 482.81541
ALogP: 4.381
Rotatable Bonds: 6
Acceptors: 4
Donors: 3

Model Prediction

Prediction: 0.000823
Unit: g/kg_body_weight
Mahalanobis Distance: 12.2
Mahalanobis Distance p-value: 4.95e-009

Mahalanobis Distance: The Mahalanobis distance (MD) is a generalization of the Euclidean distance that accounts for correlations among the X properties. It is calculated as the distance to the center of the training data. The larger the MD, the less trustworthy the prediction.
Mahalanobis Distance p-value: The p-value gives the fraction of training data with an MD greater than or equal to the one for the given sample, assuming normally distributed data. The smaller the p-value, the less trustworthy the prediciton. For highly non-normal X properties (e.g., fingerprints), the MD p-value is wildly inaccurate.

Structural Similar Compounds

Name	OCHRATOXIN	SULFISOOXAZOLE	PENICILLIN VK
Structure	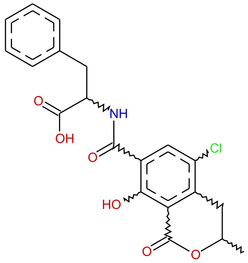	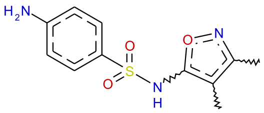	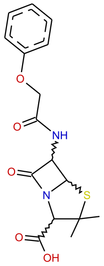
Actual Endpoint (-log C)	6.28396	2.82494	2.54455
Predicted Endpoint (-log C)	5.12358	3.0705	3.9702
Distance	0.776	1.031	1.182
Reference	NCI/NTP TR-358	NCI/NTP TR-138	NCI/NTP TR-336

Model Applicability

Unknown features are fingerprint features in the query molecule, but not found or appearing too infrequently in the training set.

1. Molecular_Weight out of range. Value: 482.82. Training min, max, mean, SD: 68.074, 434.63, 171.13, 85.06.
2. Num_AromaticRings out of range. Value: 3. Training min, max, mean, SD: 0, 2, 0.5625, 0.693.
3. OPS PC5 out of range. Value: -3.5956. Training min, max, SD, explained variance: -3.4, 4.1587, 1.489, 0.0686.
4. OPS PC7 out of range. Value: -3.867. Training min, max, SD, explained variance: -2.8003, 2.9332, 1.16, 0.0416.
5. Unknown FCFP_2 feature: 136686699: [*]NC
6. Unknown FCFP_2 feature: 1499521844: [*]NC(=O)N[*]
7. Unknown FCFP_2 feature: -1029533685: [*]:[c](:[*])C(F)(F)F

Feature Contribution

Top features for positive contribution			
**Fingerprint**	**Bit/Smiles**	**Feature Structure**	**Score**

FCFP_2	332760439	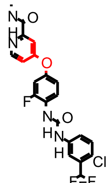 [*]O[c](:[cH]:[*]):[c H]:[*]	0.672
FCFP_2	32	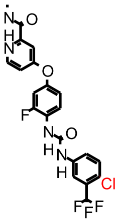 [*]Cl	0.526
FCFP_2	1	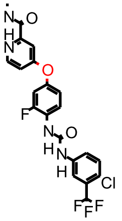 [*]=O	0.511

Top Features for negative contribution

Fingerprint	Bit/Smiles	Feature Structure	Score
FCFP_2	203677720	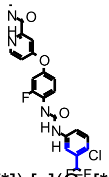 [*][c](:[*]):[c](C=[*]):[cH]:[*]	-0.406
FCFP_2	1872154524	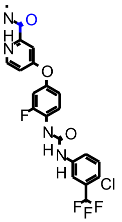 [*]C(=O)[*]	-0.307

FCFP_2	0	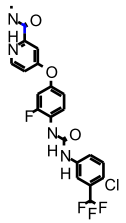<br>[*]C(=[*])[*]	-0.29

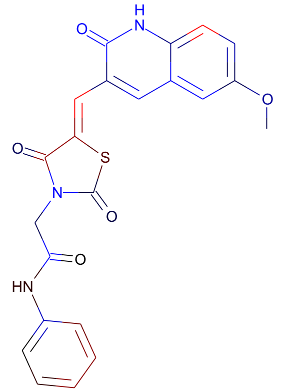

$C_{22}H_{17}N_3O_5S$
Molecular Weight: 435.45248
ALogP: 2.15
Rotatable Bonds: 5
Acceptors: 6
Donors: 2

Model Prediction

Prediction: 0.899
Unit: g/kg_body_weight
Mahalanobis Distance: 23.7
Mahalanobis Distance p-value: 2.54e-024

Mahalanobis Distance: The Mahalanobis distance (MD) is a generalization of the Euclidean distance that accounts for correlations among the X properties. It is calculated as the distance to the center of the training data. The larger the MD, the less trustworthy the prediction.
Mahalanobis Distance p-value: The p-value gives the fraction of training data with an MD greater than or equal to the one for the given sample, assuming normally distributed data. The smaller the p-value, the less trustworthy the prediciton. For highly non-normal X properties (e.g., fingerprints), the MD p-value is wildly inaccurate.

Structural Similar Compounds

Name	PIRETANIDE	ETHYL-bis-COUMACETATE	OCHRATOXIN A
Structure	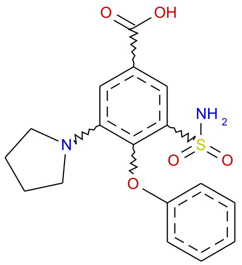	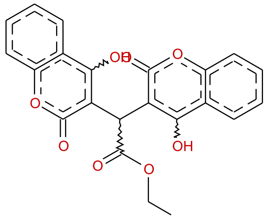	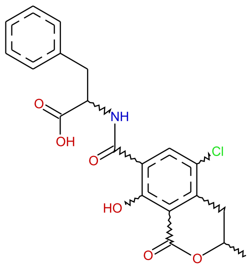
Actual Endpoint (-log C)	1.811	2.687	4.305
Predicted Endpoint (-log C)	1.83976	2.7054	3.03558
Distance	0.525	0.580	0.620
Reference	DRFUD4 2;393;77	FEPRA7 10;303;51	FCTXAV 6;479;68

Model Applicability

Unknown features are fingerprint features in the query molecule, but not found or appearing too infrequently in the training set.

1. All properties and OPS components are within expected ranges.
2. Unknown ECFP_2 feature: 2131425032: [*]\C=C(\C=[*])/C(=[*])[*]
3. Unknown ECFP_2 feature: 1000552169: [*]\C=C\1/S[*][*]C1=[*]
4. Unknown FCFP_6 feature: 16: [*][c](:[*]):[*]
5. Unknown FCFP_6 feature: 1618154665: [*][c](:[*]):[cH]:[cH]:[*]
6. Unknown FCFP_6 feature: 451371068: [*]C(=C[c](:[*]):[*])[*]

Feature Contribution

Top features for positive contribution			
Fingerprint	Bit/Smiles	Feature Structure	Score
ECFP_6	642810091	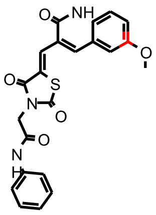 [*][c](:[*]):[*]	0.281

ECFP_6	-1897341097	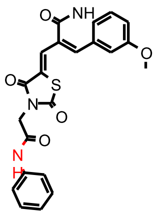<br>[*]N[*]	0.216
ECFP_6	1571214559	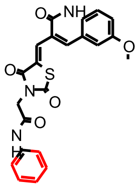<br>[*]1:[cH]:[cH]:[cH]:[cH]:[cH]:1	0.19
**Top Features for negative contribution**			
**Fingerprint**	**Bit/Smiles**	**Feature Structure**	**Score**
ECFP_6	2106656448	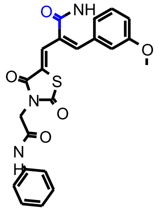<br>[*]C(=O)[*]	-0.352
ECFP_6	-176455838	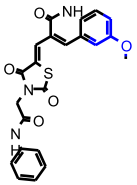<br>[*]O[c](:[cH]:[*]):[c H]:[*]	-0.257
FCFP_6	1676877079	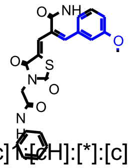<br>[*]O[c]1:[cH]:[*]:[c] ([*]):[c](C=[*]):[cH]:1	-0.254

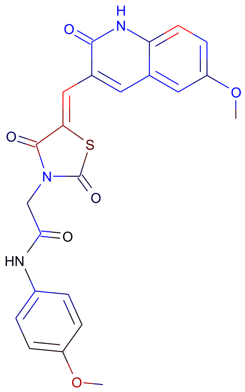

$C_{23}H_{19}N_3O_6S$
Molecular Weight: 465.47846
ALogP: 2.134
Rotatable Bonds: 6
Acceptors: 7
Donors: 2

Model Prediction

Prediction: 1.32
Unit: g/kg_body_weight
Mahalanobis Distance: 23.6
Mahalanobis Distance p-value: 1.41e-023

Mahalanobis Distance: The Mahalanobis distance (MD) is a generalization of the Euclidean distance that accounts for correlations among the X properties. It is calculated as the distance to the center of the training data. The larger the MD, the less trustworthy the prediction.
Mahalanobis Distance p-value: The p-value gives the fraction of training data with an MD greater than or equal to the one for the given sample, assuming normally distributed data. The smaller the p-value, the less trustworthy the prediciton. For highly non-normal X properties (e.g., fingerprints), the MD p-value is wildly inaccurate.

Structural Similar Compounds

Name	ETHYL-bis-COUMACETATE	PIRETANIDE	FEBANTEL
Structure	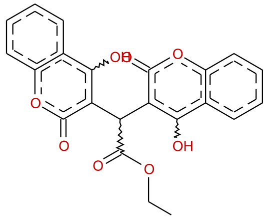	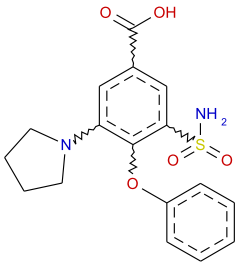	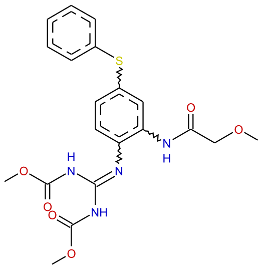
Actual Endpoint (-log C)	2.687	1.811	1.624
Predicted Endpoint (-log C)	2.7054	1.83976	2.37098
Distance	0.567	0.620	0.676
Reference	FEPRA7 10;303;51	DRFUD4 2;393;77	ARZNAD 28;2193;78

Model Applicability

Unknown features are fingerprint features in the query molecule, but not found or appearing too infrequently in the training set.

1. All properties and OPS components are within expected ranges.
2. Unknown ECFP_2 feature: 2131425032: [*]\C=C(\C=[*])/C(=[*])[*]
3. Unknown ECFP_2 feature: 1000552169: [*]\C=C\1/S[*][*]C1=[*]
4. Unknown FCFP_6 feature: 16: [*][c](:[*]):[*]
5. Unknown FCFP_6 feature: 1618154665: [*][c](:[*]):[cH]:[cH]:[*]
6. Unknown FCFP_6 feature: 451371068: [*]C(=C[c](:[*]):[*])[*]

Feature Contribution

Top features for positive contribution			
Fingerprint	Bit/Smiles	Feature Structure	Score
ECFP_6	642810091	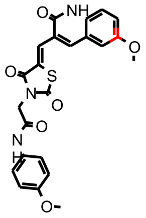 [*][c](:[*]):[*]	0.281

ECFP_6	-1897341097	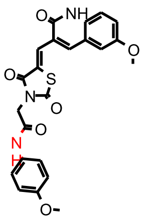<br>[*]N[*]	0.216
FCFP_6	436915834	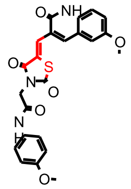<br>[*]\C=C\1/S[*][*]C1=[<br>*]	0.184

Top Features for negative contribution

Fingerprint	Bit/Smiles	Feature Structure	Score
ECFP_6	2106656448	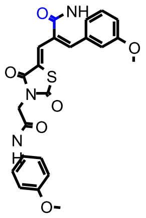<br>[*]C(=O)[*]	-0.352
ECFP_6	-176455838	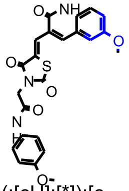<br>[*]O[c](:[cH]:[*]):[c<br>H]:[*]	-0.257
FCFP_6	1676877079	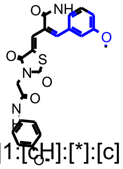<br>[*]O[c]1:[cH]:[*]:[c]<br>([*]):[c](C=[*]):[cH<br>]:1	-0.254

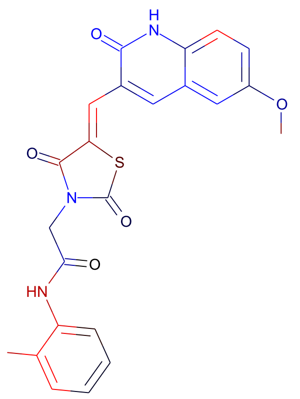

$C_{23}H_{19}N_3O_5S$
Molecular Weight: 449.47906
ALogP: 2.637
Rotatable Bonds: 5
Acceptors: 6
Donors: 2

Model Prediction

Prediction: 0.405
Unit: g/kg_body_weight
Mahalanobis Distance: 23.6
Mahalanobis Distance p-value: 1.42e-023

Mahalanobis Distance: The Mahalanobis distance (MD) is a generalization of the Euclidean distance that accounts for correlations among the X properties. It is calculated as the distance to the center of the training data. The larger the MD, the less trustworthy the prediction.
Mahalanobis Distance p-value: The p-value gives the fraction of training data with an MD greater than or equal to the one for the given sample, assuming normally distributed data. The smaller the p-value, the less trustworthy the prediciton. For highly non-normal X properties (e.g., fingerprints), the MD p-value is wildly inaccurate.

Structural Similar Compounds

Name	PIRETANIDE	ETHYL-bis-COUMACETATE	OCHRATOXIN A
Structure	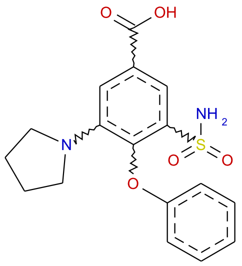	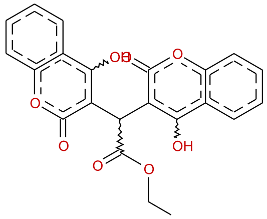	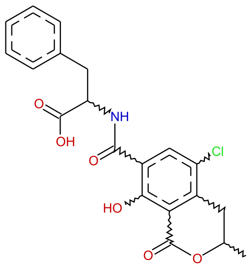
Actual Endpoint (-log C)	1.811	2.687	4.305
Predicted Endpoint (-log C)	1.83976	2.7054	3.03558
Distance	0.555	0.582	0.612
Reference	DRFUD4 2;393;77	FEPRA7 10;303;51	FCTXAV 6;479;68

Model Applicability

Unknown features are fingerprint features in the query molecule, but not found or appearing too infrequently in the training set.

1. All properties and OPS components are within expected ranges.
2. Unknown ECFP_2 feature: 2131425032: [*]\C=C(\C=[*])/C(=[*])[*]
3. Unknown ECFP_2 feature: 1000552169: [*]\C=C\1/S[*][*]C1=[*]
4. Unknown FCFP_6 feature: 16: [*][c](:[*]):[*]
5. Unknown FCFP_6 feature: 1618154665: [*][c](:[*]):[cH]:[cH]:[*]
6. Unknown FCFP_6 feature: 451371068: [*]C(=C[c](:[*]):[*])[*]

Feature Contribution

Top features for positive contribution			
Fingerprint	Bit/Smiles	Feature Structure	Score
ECFP_6	642810091	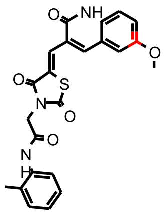 [*][c](:[*]):[*]	0.281

ECFP_6	2147419938	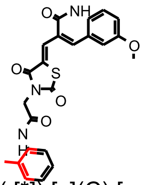 [*][c](:[*]):[c](C):[cH]:[*]	0.263
ECFP_6	-1897341097	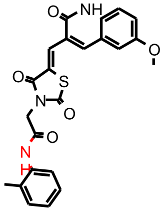 [*]N[*]	0.216
**Top Features for negative contribution**			
**Fingerprint**	**Bit/Smiles**	**Feature Structure**	**Score**
ECFP_6	2106656448	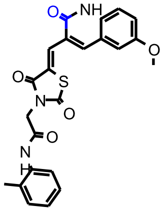 [*]C(=O)[*]	-0.352
ECFP_6	-176455838	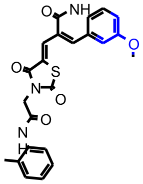 [*]O[c](:[cH]:[*]):[cH]:[*]	-0.257
FCFP_6	1676877079	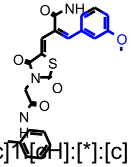 [*]O[c]1:[cH]:[*]:[c]([*]):[c](C=[*]):[cH]:1	-0.254

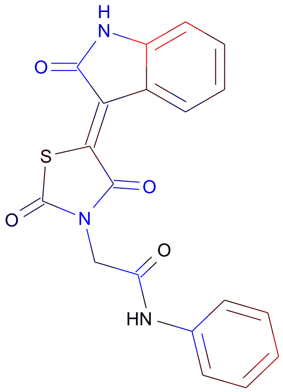

$C_{19}H_{13}N_{3}O_{4}S$
Molecular Weight: 379.38922
ALogP: 1.699
Rotatable Bonds: 3
Acceptors: 5
Donors: 2

Structural Similar Compounds

Name	PIROXICAM	PIRETANIDE	1H-1;4-BENZODIAZEPINE-1-CARBOXAMIDE; 2;3-DIHYDRO-N-METHYL-7-NITRO-2-OXO-5-PHENYL-
Structure	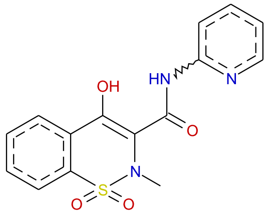	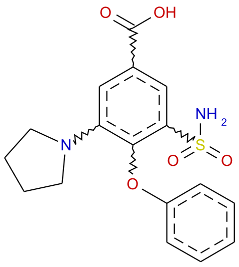	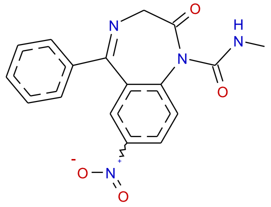
Actual Endpoint (-log C)	3.186	1.811	2.171
Predicted Endpoint (-log C)	2.63418	1.83976	2.64752
Distance	0.534	0.548	0.574
Reference	ARZNAD 28;1714;78	DRFUD4 2;393;77	TAKHAA 29;153;70

Model Prediction

Prediction: 1.4
Unit: g/kg_body_weight
Mahalanobis Distance: 21.3
Mahalanobis Distance p-value: 2.64e-013

Mahalanobis Distance: The Mahalanobis distance (MD) is a generalization of the Euclidean distance that accounts for correlations among the X properties. It is calculated as the distance to the center of the training data. The larger the MD, the less trustworthy the prediction.
Mahalanobis Distance p-value: The p-value gives the fraction of training data with an MD greater than or equal to the one for the given sample, assuming normally distributed data. The smaller the p-value, the less trustworthy the prediciton. For highly non-normal X properties (e.g., fingerprints), the MD p-value is wildly inaccurate.

Model Applicability

Unknown features are fingerprint features in the query molecule, but not found or appearing too infrequently in the training set.

1. All properties and OPS components are within expected ranges.
2. Unknown FCFP_6 feature: 16: [*][c](:[*]):[*]
3. Unknown FCFP_6 feature: 1618154665: [*][c](:[*]):[cH]:[cH]:[*]
4. Unknown FCFP_6 feature: -1678275541: [*]C(=C1C(=[*])[*][*]:[c]1:[*])[*]

Feature Contribution

Top features for positive contribution

Fingerprint	Bit/Smiles	Feature Structure	Score
ECFP_6	642810091	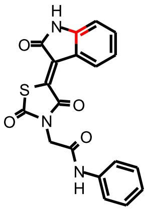 [*][c](:[*]):[*]	0.281

ECFP_6	-1897341097	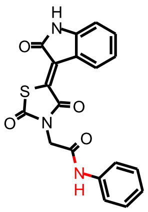 [*]N[*]	0.216
ECFP_6	1571214559	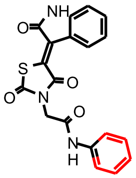 [*]1:[cH]:[cH]:[cH]:[cH]:[cH]:1	0.19

Top Features for negative contribution

Fingerprint	Bit/Smiles	Feature Structure	Score
ECFP_6	2106656448	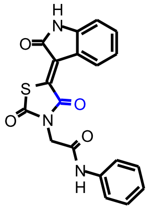 [*]C(=O)[*]	-0.352
FCFP_6	566058135	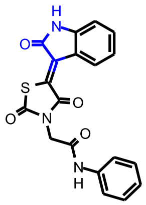 [*]NC(=O)C(=[*])[*]	-0.216
ECFP_6	670515721	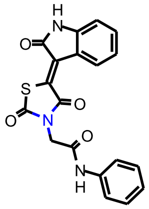 [*]N([*])[*]	-0.108

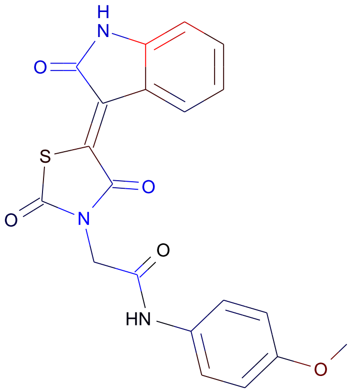

$C_{20}H_{15}N_3O_5S$

Molecular Weight: 409.4152

ALogP: 1.683

Rotatable Bonds: 4

Acceptors: 6

Donors: 2

Model Prediction

Prediction: 1.21

Unit: g/kg_body_weight

Mahalanobis Distance: 21.6

Mahalanobis Distance p-value: 1.24e-014

Mahalanobis Distance: The Mahalanobis distance (MD) is a generalization of the Euclidean distance that accounts for correlations among the X properties. It is calculated as the distance to the center of the training data. The larger the MD, the less trustworthy the prediction.
Mahalanobis Distance p-value: The p-value gives the fraction of training data with an MD greater than or equal to the one for the given sample, assuming normally distributed data. The smaller the p-value, the less trustworthy the prediciton. For highly non-normal X properties (e.g., fingerprints), the MD p-value is wildly inaccurate.

Structural Similar Compounds

Name	PIRETANIDE	ETHYL-bis-COUMACETATE	PIROXICAM
Structure	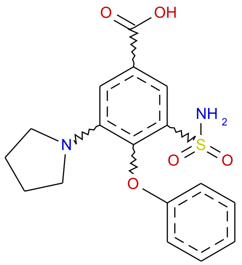	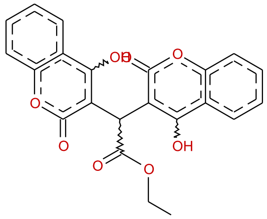	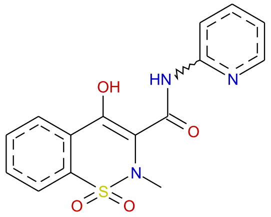
Actual Endpoint (-log C)	1.811	2.687	3.186
Predicted Endpoint (-log C)	1.83976	2.7054	2.63418
Distance	0.514	0.586	0.614
Reference	DRFUD4 2;393;77	FEPRA7 10;303;51	ARZNAD 28;1714;78

Model Applicability

Unknown features are fingerprint features in the query molecule, but not found or appearing too infrequently in the training set.

1. All properties and OPS components are within expected ranges.
2. Unknown FCFP_6 feature: 16: [*][c](:[*]):[*]
3. Unknown FCFP_6 feature: 1618154665: [*][c](:[*]):[cH]:[cH]:[*]
4. Unknown FCFP_6 feature: -1678275541: [*]C(=C1C(=[*])[*][*]:[c]1:[*])[*]

Feature Contribution

Top features for positive contribution

Fingerprint	Bit/Smiles	Feature Structure	Score
ECFP_6	642810091	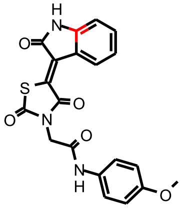 [*][c](:[*]):[*]	0.281

ECFP_6	-1897341097	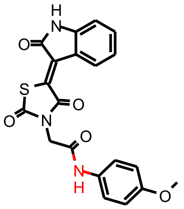 [*]N[*]	0.216
FCFP_6	436915834	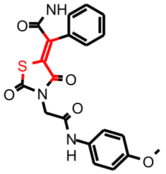 [*]\C=C\1/S[*][*]C1=[*]	0.184

Top Features for negative contribution

Fingerprint	Bit/Smiles	Feature Structure	Score
ECFP_6	2106656448	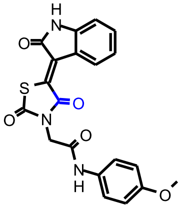 [*]C(=O)[*]	-0.352
ECFP_6	-176455838	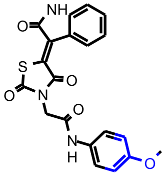 [*]O[c](:[cH]:[*]):[cH]:[*]	-0.257
FCFP_6	566058135	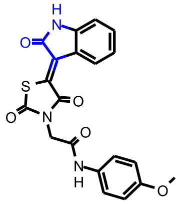 [*]NC(=O)C(=[*])[*]	-0.216

Sorafenib

TOPKAT_Rat_Oral_LD50

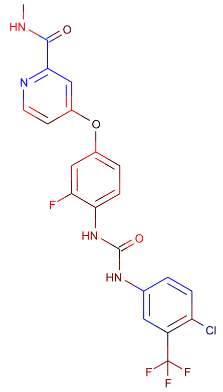

$C_{21}H_{15}ClF_4N_4O_3$

Molecular Weight: 482.81541

ALogP: 4.381

Rotatable Bonds: 6

Acceptors: 4

Donors: 3

Structural Similar Compounds

Name	PHOSPHORAMIDOTHIOIC ACID; ACETIMIDOYL-; O;O-bis-(p-CHLOROPHENYL)ESTER	FLUBENDAZOLE	OXYCLOZANIDE
Structure	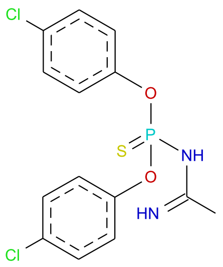	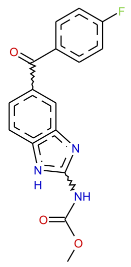	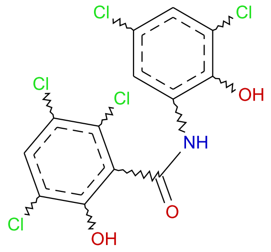
Actual Endpoint (-log C)	5.006	2.088	2.604
Predicted Endpoint (-log C)	3.23989	2.69288	2.94104
Distance	0.720	0.728	0.732
Reference	FMCHA2 -;C149;89	YRTMA6 9;11;78	NATUAS 210;744;66

Model Prediction

Prediction: 0.89

Unit: g/kg_body_weight

Mahalanobis Distance: 21.1

Mahalanobis Distance p-value: 1.49e-012

Mahalanobis Distance: The Mahalanobis distance (MD) is a generalization of the Euclidean distance that accounts for correlations among the X properties. It is calculated as the distance to the center of the training data. The larger the MD, the less trustworthy the prediction.
Mahalanobis Distance p-value: The p-value gives the fraction of training data with an MD greater than or equal to the one for the given sample, assuming normally distributed data. The smaller the p-value, the less trustworthy the prediciton. For highly non-normal X properties (e.g., fingerprints), the MD p-value is wildly inaccurate.

Model Applicability

Unknown features are fingerprint features in the query molecule, but not found or appearing too infrequently in the training set.

1. All properties and OPS components are within expected ranges.
2. Unknown FCFP_6 feature: 16: [*][c](:[*]):[*]
3. Unknown FCFP_6 feature: 1618154665: [*][c](:[*]):[cH]:[cH]:[*]
4. Unknown FCFP_6 feature: 1747237384: [*][c](:[*]):n:[cH]:[*]
5. Unknown FCFP_6 feature: 136686699: [*]NC
6. Unknown FCFP_6 feature: 71476542: [*]:[c](:[*])Cl

Feature Contribution

Top features for positive contribution

Fingerprint	Bit/Smiles	Feature Structure	Score

FCFP_6	71953198	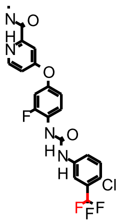 [*]C([*])([*])F	0.392
ECFP_6	-1046436026	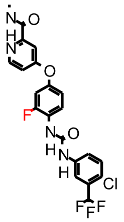 [*]F	0.349
ECFP_6	642810091	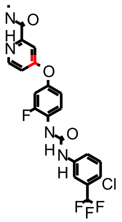 [*][c](:[*]):[*]	0.281
**Top Features for negative contribution**			
**Fingerprint**	**Bit/Smiles**	**Feature Structure**	**Score**
ECFP_6	226796801	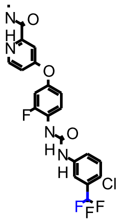 [*]C([*])([*])F	-0.32
ECFP_6	-817402818	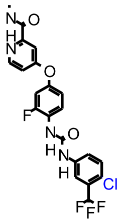 [*]Cl	-0.263

ECFP_6	-176455838	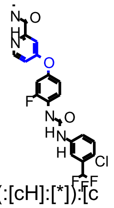 [*]O[c](:[cH]:[*]):[cH]:[*]	-0.257
